# A novel approach to metabolic profiling in case models of *MECP2*-related disorders

**DOI:** 10.1007/s11011-025-01546-5

**Published:** 2025-02-13

**Authors:** Jessica A. Cooley Coleman, Bridgette A. Moffitt, William C. Bridges, Kelly Jones, Melanie May, Cindy Skinner, Michael J. Friez, Steven A. Skinner, Charles E. Schwartz, Luigi Boccuto

**Affiliations:** 1 Greenwood Genetic Center, Greenwood, SC 29646 USA; 2 School of Nursing, College of Behavioral, Social and Health Sciences, Clemson University, Clemson, SC 29634 USA; 3 School of Mathematical and Statistical Sciences, Clemson University, Clemson, SC 29634 USA

**Keywords:** *MECP2*, Rett syndrome, *MECP2* duplication syndrome, *MECP2*-related disorders, *IRAK1*, Trofenatide

## Abstract

Genetic abnormalities of the *MECP2* gene cause several conditions grouped under the umbrella term of *MECP2*-related disorders and characterized by a variety of phenotypes. We applied a functional approach to identify metabolic profiles in two patients with Rett syndrome (RTT) and one patient with *MECP2* duplication syndrome (MRXSL). Such an approach is based on the Phenotype Mammalian Microarray (PM-M) technology, which is designed to assess the cellular production of energy in the presence of different compounds generating distinct metabolic environments. The findings in the three case models were compared versus 50 controls. Although the small number of samples prevented most results from reaching significant *p*-values when adjusted with the Benjamini-Hochberg correction, some interesting trends emerged. Some compounds indicated metabolic trends shared by the two conditions, like increased energy production in the presence of energy sources such as pectin, adenosine, and pyruvic acid, or decreased metabolic response to certain hormones. Other compounds showed opposite trends for the two disorders, like interleukin-1 beta (IL-1 beta), which caused decreased energy production in the RTT group but increased energy production in the patient with MRXSL. The response to IL-1 beta also offers valuable insights into the pathogenic mechanism and potential therapeutic approaches. The metabolic profiling of *MECP2*-related disorders bears a remarkable translational potential since it may be helpful to investigate the molecular abnormalities underlying the phenotypical variety in this spectrum of conditions, develop biomarkers for the identification of ideal candidates for treatments like the recently approved trofenatide, and identify potential targets for the development of novel therapeutic approaches.

**Supplementary Information:**

The online version contains supplementary material available at 10.1007/s11011-025-01546-5.

## Introduction

The *Methyl-CpG Binding Protein 2* gene (*MECP2*, MIM *300005) maps on chromosome Xq28 and encodes the MECP2 protein, a transcriptional regulator capable of both repression and activation of numerous genes (Yasui et al. 2007; Chahrour et al. 2008), resulting in modulation of a vast number of targeted pathways, including among others the synthesis and degradation of cytoskeletal components, vesicular transport elements, ribosomal subunits and mRNA processing machinery (Pascual-Alonso et al. 2023). *MECP2*-related disorders include classic Rett syndrome (RTT) (MIM 312750), atypical or variant RTT (MIM 312750), severe neonatal encephalopathy (MIM 300673), X-linked intellectual disability (MIM 300055), and *MECP2* duplication syndrome or X-Linked intellectual disability syndrome, Lubs type MRXSL (MIM 300260) (Online Mendelian Inheritance in Man, OMIM^®^ n.d.; Vidal et al. 2019; Collins and Neul 2022). There is a vast clinical variability with disorders associated with *MECP2*, often depending on the specific variant and pattern of X inactivation for females (Villard et al. 2000). Classic RTT is a severe neurodevelopmental disorder that typically only affects females but has been seen in cases of males with Klinefelter syndrome or mosaic variants in *MECP2* (Leonard et al. 2001; Collins and Neul 2022). The prevalence ranges from 1 in 10,000 to 1 in 23,000 live female births (Kaur and Christodoulou 2001). Patients with RTT have an apparently normal development up to about 18 months of age, then undergo a period of regression (Neul et al. 2010). In addition to regression, RTT has four main clinical criteria: loss of purposeful hand movements, loss of acquired spoken language, abnormal gait, and stereotypic or repetitive hand movements. Supportive criteria can include bruxism and/or breathing disturbances while awake, sleep impairment, abnormal muscle tone, peripheral vasomotor disturbances, fits of screaming or inappropriate laughter, scoliosis or kyphosis, cold and small hands and feet, diminished response to pain, intense eye communication, and growth retardation (Kaur and Christodoulou 2001; Neul et al. 2010; Leonard et al. 2017).

*MECP2* duplication syndrome (MIM #300260), also known as Lubs-type X-linked syndromic intellectual developmental disorder (MRXSL), is characterized by developmental delay, severe intellectual disability, seizures, poor speech, autistic features, extreme progressive spasticity, gastrointestinal issues, and recurrent infections in males (Pascual-Alonso et al. 2021). MRXSL mainly affects males, with females having a less severe phenotype, including anxiety, autistic features, and mild intellectual impairment. MRXSL has a 100% penetrance within males. The exact prevalence is unknown; however, several studies have reported a prevalence of 1% in males with moderate to severe intellectual disabilities (Van Esch 2008). Most patients with a *MECP2* duplication also have an *IRAK1* duplication due to the close proximity of the two genes (Sugitate et al. 2022), and the full duplication of both genes may be necessary for the correct diagnosis of MRXSL (Pascual-Alonso et al. 2020). *IRAK1* (Interleukin 1 Receptor-Associated Kinase 1) encodes a serine/threonine kinase of the proinflammatory cytokine interleukin-1 receptor (IL-1R) and is thought to potentially play a role in the inflammatory responses seen in patients with MRXSL (Bauer et al. 2015). Given each patient has a different size duplication, other nearby genes, such as *L1CAM*, are often included in a patient’s duplication. The roles that many of these other genes play are unclear; however, the duplicated region in common for most patients includes *MECP2* and *IRAK1* (Allison et al. 2024).

As a transcription regulator, MECP2 binds to CpG islands within the promoter of genes to mediate either repression or activation and interacts with protein corepressors and coactivators (Chahrour et al. 2008). Genes repressed by MECP2 binding had higher methylation of CpG sites in their promotor regions than genes activated by MECP2 (Chahrour et al. 2008). Studies using *Mecp2*-Tg mice (having a gain of *Mecp2*, reminiscent of *MECP2* duplication syndrome) had significantly more activation, while *Mecp2*-null mice (reminiscent of Rett syndrome) had more gene repression. Additionally, most of the genes activated in *Mecp2*-Tg mice were the same genes that were repressed in *Mecp2*-null mice (Chahrour et al. 2008). These results indicate that the molecular mechanism for RTT is loss of function due to loss of transcriptional activation of downstream targets, while the mechanism for *MECP2* duplication syndrome is *MECP2* gain of function resulting in increased expression of the same downstream targets. Other mouse models may better emphasize the complexities of each disorder. For example, mouse models overexpressing *Mecp2*, including the *Mecp2*-Tg mice, do not completely model what is seen at the genomic level in human patients with MRXSL, that of a tandem duplication that usually also includes *IRAK1*. A recent study utilized a CRISPR/Cas9 fusion proximity-based approach to generate an *Irak1-Mecp2* tandem duplication mouse model, termed ‘*Mecp2 Dup*’ (Maino et al. 2024). Maino et al. assessed the relative expression levels of certain dysregulated genes reported in other *MECP2* duplication models and found that these previously reported trends were in agreement in their new *Mecp2 Dup* mouse model. Numerous other mouse models have also been developed for RTT, including ones that harbor each of the eight most common *MECP2* pathogenic variants, including two RTT variants in the current study, Thr158Met and Arg168Ter (Wegener et al. 2014; Brown et al. 2016).

In addition to being a regulator of transcription, MECP2 is involved in chromatin scaffolding, DNA methylation maintenance, RNA splicing, and microRNA processing (Horvath and Monteggia 2018). MECP2 is also involved in numerous pathways, including MAPK signaling, ERBB signaling, inflammation, sterol biosynthesis, cholesterol metabolism, cytoskeleton formation, and apoptosis (Ehrhart et al. 2016). Cell morphology, synapsis function, and neuron development, growth, and maintenance depend on a complex regulatory network involving MECP2. Due to the complex homeostasis of the numerous pathways regulated by MECP2, the molecular and clinical effects of *MECP2* loss or duplication may be remarkably variable and may include overlapping features as well as different ones. Exploring the metabolic profiles associated with RTT and MRXSL, the two conditions associated with *MECP2* loss and duplication, respectively, may help us understand the mechanisms underlying the pathogenesis of *MECP2*-related disorders.

In light of the recent approval by the FDA of clinical trials employing the drug trofinetide for the treatment of RTT (Harris 2023; Hudu et al. 2023; Neul et al. 2023), we thought that the identification of potential metabolic biomarkers might be instrumental in identifying ideal candidates for treatments and providing precise outcome measures for clinical trials. Moreover, thorough metabolic profiling of individuals with *MECP2*-related disorders may reveal novel molecular targets that might suggest new potential drugs or potential pharmacogenomic dynamics influencing the response to trofinetide.

In this study, we report the metabolic profiles of one patient with MRXSL and two patients with RTT using Biolog Phenotype Microarray plates. Comparing the metabolic profiles of gain of function versus loss of function of the same gene may give further insight into pathogenic mechanisms, identification of biomarkers, and potential molecular targets and/or candidate drugs.

## Materials and methods

### Participants

Two female individuals with RTT (ages 12 and 32 years at the time of sample collection), one male individual with MRXSL (age 5 years at the time of sample collection), and fifty controls were utilized in this study. The controls consisted of 26 females and 24 males, ranging in age from one to ten years, at the time of sample collection. Participants were recruited at the Greenwood Genetic Center (GGC) in Greenwood, South Carolina. This study was performed in line with the principles of the Declaration of Helsinki. Approval was granted by the Self Regional Healthcare Institutional Review Board (Pro00056079, IRB Study #24). Informed consent for research studies was obtained from the parents or legal guardians of the participants. Peripheral blood was collected from each participant, and lymphoblastoid cell lines (LCLs) were created from blood samples using Epstein-Barr virus (EBV) transformation. LCLs were cultured for the metabolic arrays and tested only after they reached at least 55% viability.

### Clinical diagnostic testing

The Greenwood Genetic Center Molecular Diagnostic Laboratory received peripheral blood from all three patients. DNA was isolated using the FlexiGene DNA Kit (Qiagen). For the two individuals with RTT, PCR was performed to amplify the *MECP2* gene (NM_004992.3) following standard procedures. PCR products were Sanger sequenced using BigDye Terminator v3.1 Cycle Sequencing Kit and then run on an Applied Biosystems 3730xl DNA Analyzer (Thermo Fisher Scientific). For the individual with MRXSL, multiplex ligation-dependent probe amplification (MLPA) was performed using the SALSA MLPA P015C kit (MRC-Holland, Amsterdam, The Netherlands) according to the manufacturer’s instructions. Clinical testing was performed and reported years prior to the research studies; therefore, images of the Sanger electropherograms are not currently available.

### Biolog metabolic arrays

**Fig. 1:**
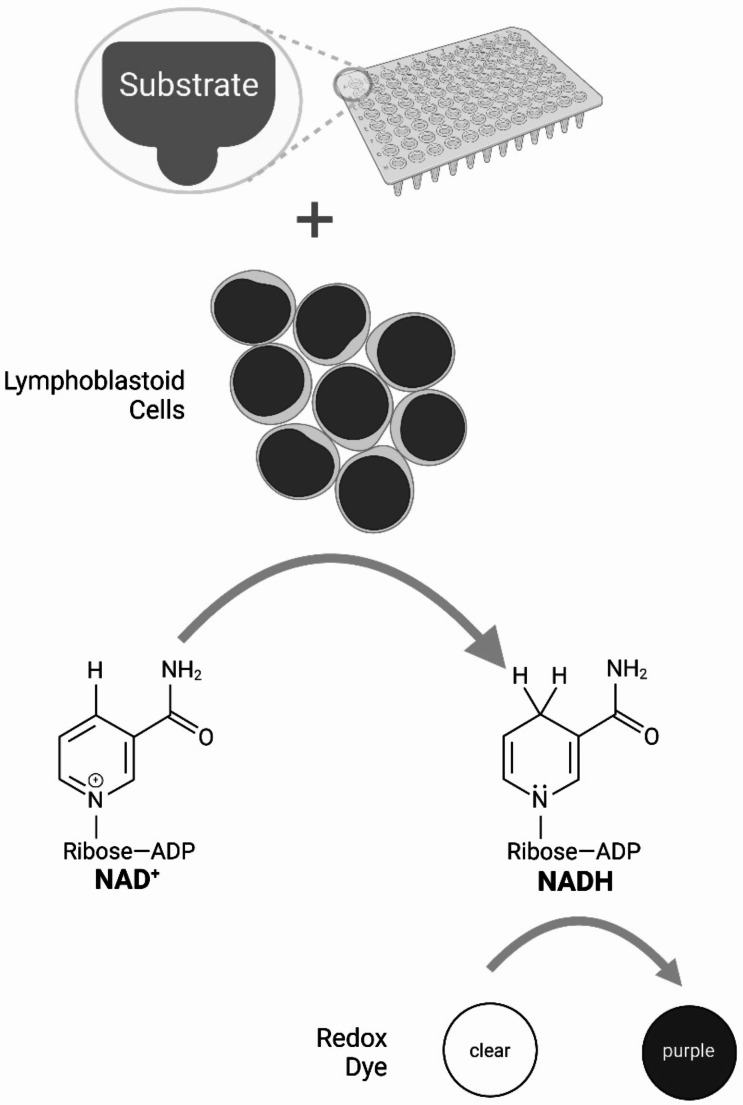
Schematic for the method of the Biolog Phenotype Mammalian MicroArray (PM-M) plates. Each plate is pre-loaded with carbon and nitrogen substrates, ions, or stimulatory or inhibitory compounds. Lymphoblastoid cells of patients or controls are added to the plate along with redox dye. Upon NADH production by the cells metabolizing the compound, redox dye is reduced, resulting in a color change in which the density directly correlates to the amount of NADH produced

Phenotype Mammalian MicroArray (PM-M) plates by Biolog (Hayward, CA, USA) were used to assess the metabolic phenotypes of RTT cells compared to MRXSL cells. These plates measure the cellular production of NADH (nicotinamide adenine dinucleotide, reduced form) in the presence of carbon energy sources (PM-M1 plate), amino acids and dipeptides (PM-M2 to M4), ions (PM-M5), hormones and metabolic effectors (PM-M6 to M8). Each well of the PM-M plate contains a different compound, some of which include multiple concentration points. Upon NADH production, redox dye within each well is reduced, resulting in a color change to purple, of which the density directly correlates to the amount of NADH produced (Fig. 1). The optical density is measured using the Biolog OmniLog automated incubator-reader.

Each plate was incubated with 20,000 viable cells per cell in a volume of 50 µL, using the modified Biolog IF-M1 medium. The modified medium was prepared by adding 1.1 mL of 100× penicillin/streptomycin solution, 0.16 mL of 200 mM Glutamine (final concentration 0.3 mM), and 275 µL of α-D-glucose (final concentration 5.5 mM) to 100 mL of Biolog IF-M1. The cells were incubated for 48 h at 37 °C in 5% CO_2_, after which 10 µL of Biolog Redox Dye Mix MB was added to each well. The plates were again incubated at the same temperature for 24 h, and the optical density was calculated by the Omnilog system every 15 min during this incubation. The area under the curve (AUC) was calculated from the resulting kinetic curves of the NADH production. After this incubation, the plates were also analyzed utilizing a microplate reader with readings at 590 (the highest absorbance peak of the redox dye) and 750 nm (for a measure of background noise), and the relative absorbance (A_590−750_) was calculated per well.

### Data processing and statistical analysis of Phenotype Mammalian MicroArray (PM-M) Data

Absorbance and AUC readings from the PM-M plates were transformed into a logarithmic scale. For the purpose of the analysis, the null hypothesis was that each group (the patients with Rett / loss of function versus the patient with the *MECP2* duplication) has a separate and unique PM-M profile. The data was analyzed using a custom R package (version 3.6) and the opm R package (available at R-Forge.r-project.org). The values generated for our patients were compared individually (MRXSL, Arg168Ter, or Thr158Met) and as a disorder category (RTT or MRXSL) to the average values of 50 control individuals. Additionally, a comparison of RTT to MRXSL was performed. The non-parametric Mann–Whitney two-sided test was used to calculate *p*-values. *P*-values were deemed statistically significant at < 0.05. To control for multiple testing, the Benjamini-Hochberg correction (R method: p.adjust) was applied with a false discovery rate (FDR) set to q < 0.05. Due to the small sample size, alternative statistical methods were subsequently applied. Patient values for each compound tested on the PM-M plates were graphed on box-and-whisker plots. Using the inter-ocular trauma test, compounds were deemed of statistical interest when patient values fell above or below the box-and-whisker plots’ outer limits.

## Results

### Clinical presentation of patients

This study investigates one patient with MRXSL and two diagnosed with RTT who have loss-of-function pathogenic variants of *MECP2*.

Patient 1 (P1) is a 30-year-old Caucasian male diagnosed with MRXSL at 14 years of age. He has severe intellectual disability and remains nonverbal. He had severe psychomotor delay from birth and hypotonia, but had no growth delay. Upon physical examination, he was noted to have an asymmetric skull with brachycephaly, a high-arched palate, and a tented upper lip with excessive drooling. By 14 months of age, he was able to sit but not able to stand or walk. By age 3 years and 9 months, he was able to commando crawl but was still unable to stand or walk. At age 9 years, he began having myoclonic and atonic seizures occurring daily and was prescribed Depakote and Topamax, as well as Diastat for any prolonged seizures. He was prone to recurrent respiratory infections, particularly pneumonia, and had issues with gastroesophageal reflux. Multiplex-ligation-dependent probe amplification (MLPA) revealed a duplication encompassing the *MECP2* and *L1CAM* genes (Supplemental Fig. 1).

Patient 2 (P2) is a 56-year-old Caucasian female who was diagnosed with Rett syndrome at 32 years of age. Limited clinical information was noted when she was referred to GGC for diagnostic testing, which included profound intellectual disability and “other specified cerebral degenerations in childhood”. Prior testing showed an apparently balanced karyotype with a pericentric inversion of the X chromosome (46,X, inv(X)(p22.3q26). Sanger sequencing of the *MECP2* gene revealed a heterozygous nonsense c.502 C > T variant, leading to the substitution of the arginine in residue 168 with a premature stop codon (p.Arg168Ter). This variant is pathogenic per the American College of Medical Genetics and Genomics (ACMG) guidelines and has been reported in multiple individuals with Rett syndrome in the Human Gene Mutation Database (HGMD) and RettBase (Krishnaraj et al. 2017).

Patient 3 (P3) is a 27-year-old Caucasian female who was diagnosed with Rett syndrome at five years of age. She met early developmental milestones, including rolling at 4.5 months, sitting at six months, and walking independently at 18 months, but then regressed in her development. By 18 months, she had at least four words but then stopped speaking abruptly. She had repetitive hand movements and would either keep her hands in her mouth or at her midline. She had corrective surgery for strabismus at around two years of age and heel cord surgery at five years of age. She had a history of absence and atonic seizures for which she was prescribed Depakote. She has microcephaly, hypotonia, and poor balance. Sanger sequencing of the *MECP2* gene revealed a heterozygous missense variant, c.473 C > T, causing a p.Thr158Met substitution. This variant is pathogenic per ACMG guidelines and is noted as a common and recurrent pathogenic variant in individuals with Rett syndrome in HGMD and RettBase (Krishnaraj et al. 2017).

### Metabolic profiling of in vitro models of *MECP2* alterations

Due to our small sample size of patients, no wells for the MRXSL cell line reached statistical significance when analyzed against controls. Initially, 264 wells (39.3%) were significantly different for the two patients with RTT compared to controls; however, the majority of these wells lost their significant *p*-value when adjusted with the Benjamini-Hochberg correction. Ninety-five wells (14.1%), which are compounds on the PM-M8 plate containing hormones and metabolic effectors, retained a significant *p*-value. Data analysis via our custom R package showed that the cells with *MECP2* loss-of-function (LOF) variants produced a lower metabolic response than controls in every well on plate PM-M8 except for well G10 (TNF-alpha), a trend largely confirmed by the box-and-whisker plots. When comparing the individual RTT pathogenic variant (either p.Arg168Ter or p.Thr158Met) against controls, no wells were statistically significant. Also, when comparing MRXSL to the two RTT patients, no wells were statistically significant (see supplemental files), likely due to the low sample number. Additionally, when comparing the male MRXSL patient to only male controls, no wells were statistically significant. However, when comparing the two female patients with RTT to female controls, several wells in plate PM-M3, M4, and M8 achieved statistical significance. Many of the significant wells contained amino acids with important roles in the central nervous system: for example, 12/30 (40%) wells containing glycine, which functions as neurotransmitter in both inhibitory and excitatory synapses, and 11/27 (40.7%) wells containing tyrosine, the chemical precursor of the neurotransmitter dopamine, showed reduced NADH production in the RTT samples. Moreover, results from PM-M8 indicate a reduced energy production in the presence of any tested concentration of TNF-alpha and interferon gamma, strengthening the hypothesis of a disruption of the immune system. All the data from the analysis for each group are available in the Supplemental Files.

**Fig. 2:**
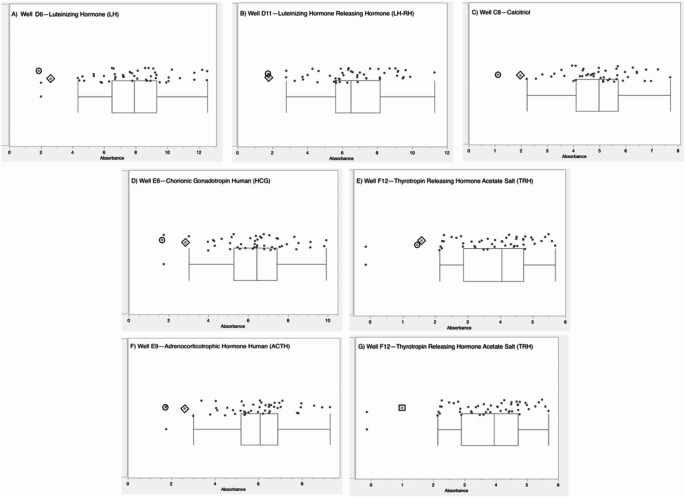
Compounds with notable differences in metabolic activity and energy production after analysis of box-and-whisker plots using the inter-ocular trauma test for each well in PM-M8, where the circle enclosed * represents the female LOF patient with the nonsense variant, the diamond enclosed * represents the female LOF patient with the missense variant, the square enclosed * represents the male patient with the *MECP2* duplication, and the dots represent the controls. *P*-values < 0.11. (**A**) D06—Luteinizing Hormone (LH) for LOF patients. (**B**) well D11—Luteinizing Hormone Releasing Hormone (LH-RH) for LOF patients. (**C**) well C08—Calcitriol (1a,25-dihydroxyvitaminD3) for LOF patients. (**D**) well E06—Chorionic Gonadotropin Human (HCG) for LOF patients. (**E**) well F12—Thyrotropin Releasing Hormone Acetate Salt (TRH) for LOF patients. (**F**) well E09—Adrenocorticotrophic Hormone Human (ACTH) for LOF patients. (**G**) well F12—Thyrotropin Releasing Hormone Acetate Salt (TRH) for the patient with the *MECP2* duplication

**Fig. 3:**
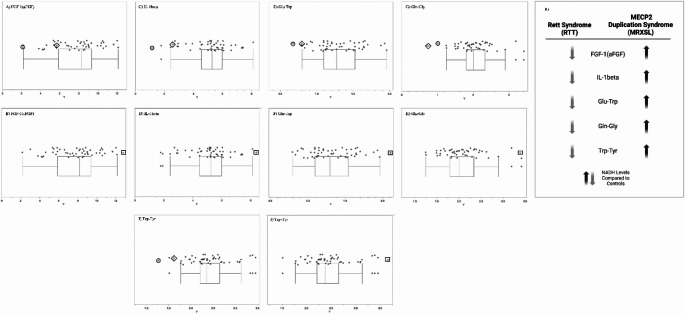
Compounds where patient groups exhibited opposite trends in metabolic activity and energy production after analysis of box-and-whisker plots using the inter-ocular trauma test, where the circle enclosed * represents the female LOF patient with the nonsense variant, the diamond enclosed * represents the female LOF patient with the missense variant, the square enclosed * represents the male patient with the *MECP2* duplication, and the dots represent the controls. *P*-values < 0.11. Plate PM-M7, well F03—FGF-1 (aFGF) for (**A**) LOF patients and (**B**) the patient with the *MECP2* duplication. Plate PM-M7, well G01—IL-1beta for (**C**) LOF patients and (**D**) the patient with the *MECP2* duplication. Plate PM-M2, well H02—Glu-Trp for (**E**) LOF patients and (**F**) the patient with the *MECP2* duplication. Plate PM-M2, well H07—Gln-Gly for (**G**) LOF patients and (**H**) the patient with the *MECP2* duplication. Plate PM-M4, well F05—Trp-Tyr for (**I**) LOF patients and (**J**) the patient with the *MECP2* duplication. **K**) Simplified representation of the compounds that exhibited opposite trends between the two patient groups

The complete list of wells with box-and-whisker plots deemed to have notable visual differences between patients and controls using the inter-ocular trauma test can be found in Table 1, and the individual plots for each well are available in the Supplemental Files. Patient 1 with the *MECP2* duplication showed 25 compounds that produced lower NADH levels and 66 compounds that produced higher NADH levels than controls. In the two patients with *MECP2* LOF variants, 64 compounds produced lower NADH levels, and only five produced higher NADH levels than controls. Pectin, adenosine, pyruvic acid, Ile-Gln, and Ser-Gln all had higher NADH levels for both groups of patients (*MECP2* duplication and *MECP2* LOF) as compared to controls. Several compounds shared a lower NADH level for both groups as compared to controls (Fig. 2, A to G). Only five compounds were found in opposing directions between the two groups of patients: FGF-1(aFGF), IL-1beta, Glu-Trp, Gln-Gly, and Trp-Tyr (Fig. 3). These five compounds yielded higher NADH than controls in the patient with the *MECP2* duplication but lower NADH than controls in the two patients with LOF variants.

Overall, the patient with a *MECP2* duplication showed an increase in the utilization of main energy sources (see Table 1), including carbohydrates (PM-M1) and dipeptides (PM-M2, PM-M3, and PM-M4), using the box-and-whisker inter-ocular test. This patient had a decrease in utilization of ions (PM-M5), hormones, and metabolic effectors (PM-M6, PM-M7, and PM-M8). The PM-M5 plate contains ionic compounds in sets of four wells with increasing concentration. Although a few wells on this plate had a visual difference in our patient, generally it was only the most concentrated replicate of each compound that differed the most from controls. This patient also showed a decreased metabolic response to several hormones and metabolic effectors (Table 1; Fig. 2G), suggesting a reduced ability for the patient’s cells to adjust to specific hormone signals.

Conversely, the patients with *MECP2* loss of function variants overall showed a decrease in their utilization of compounds across all plates, with the exception of plate PM-M1 (Table 1). Only four carbohydrates on PM-M1 differed from controls, and all had an increase in utilization in the two patients. The utilization of dipeptides decreased in the LOF patients (opposite of the increase seen in the patient with the *MECP2* duplication). All notably trending wells containing hormones and metabolic effectors also induced a lower metabolic response (Table 1; Fig. 2A-F), suggesting a decreased ability for LOF patient cells to adjust to specific hormone signals.

**Table 1:**
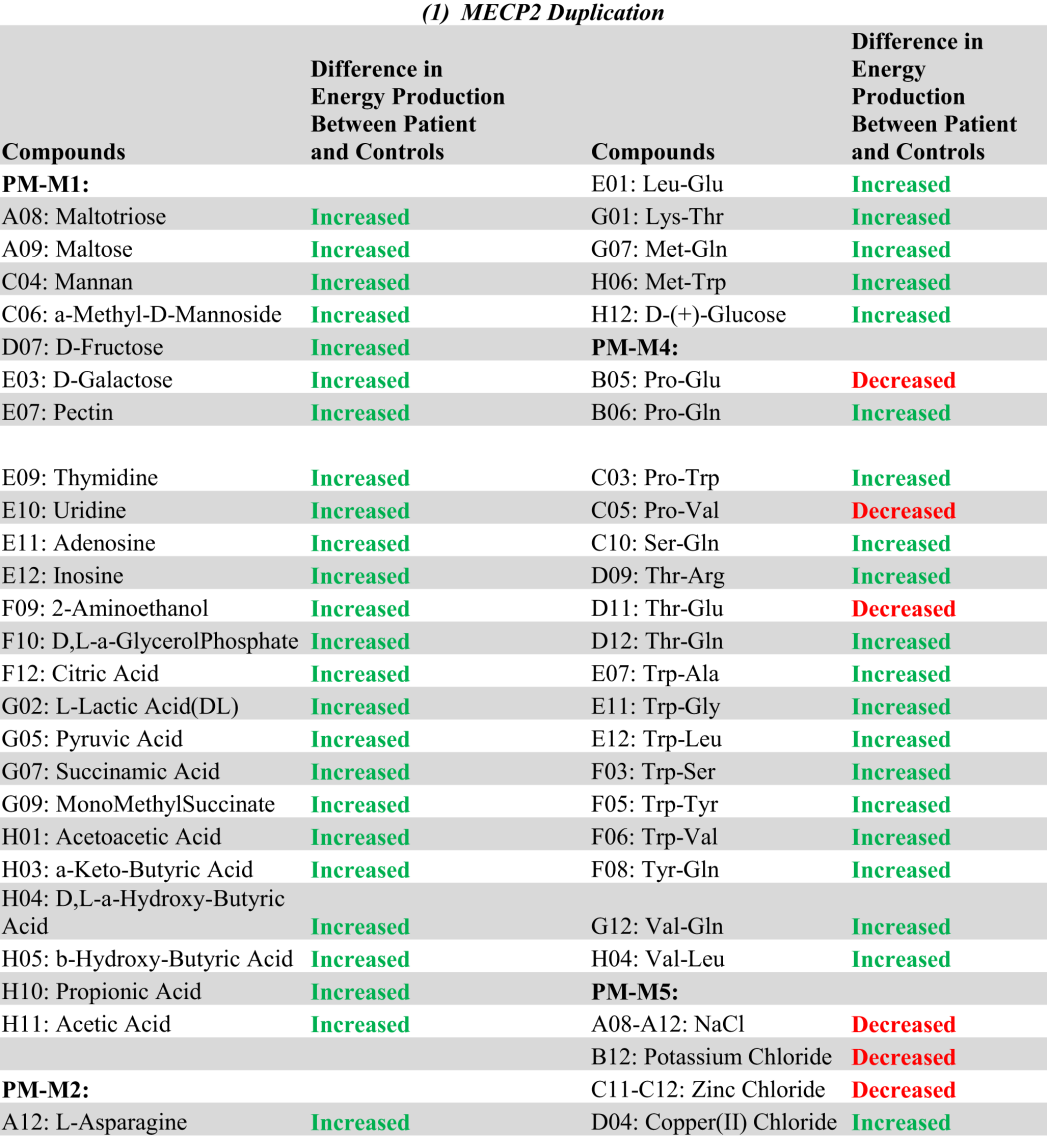

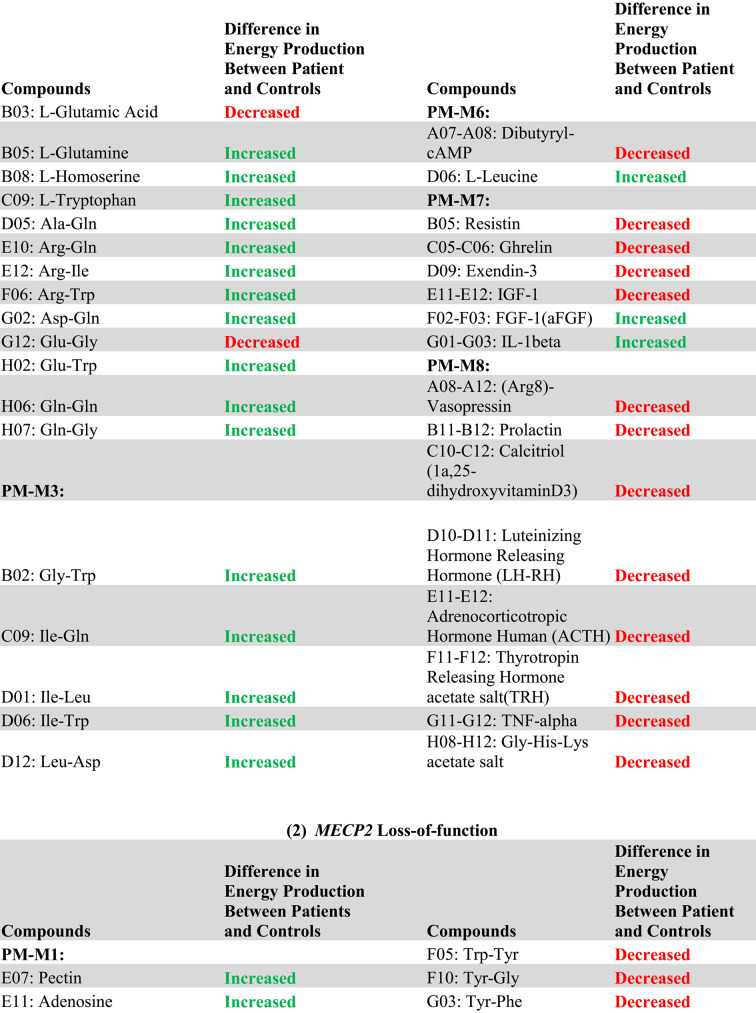

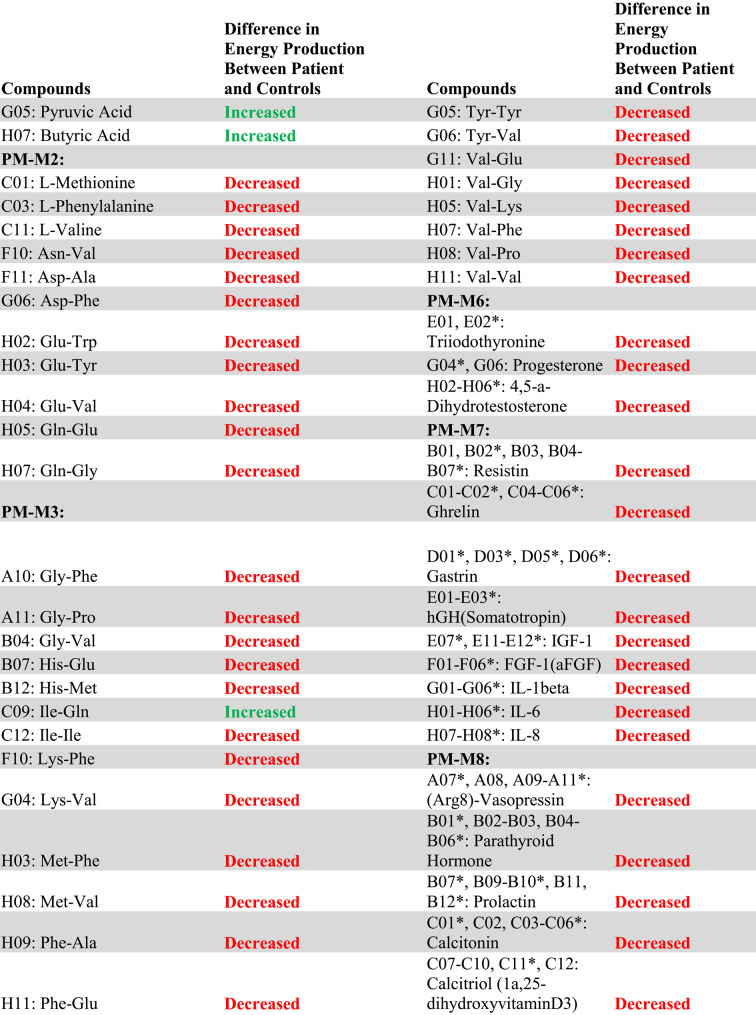

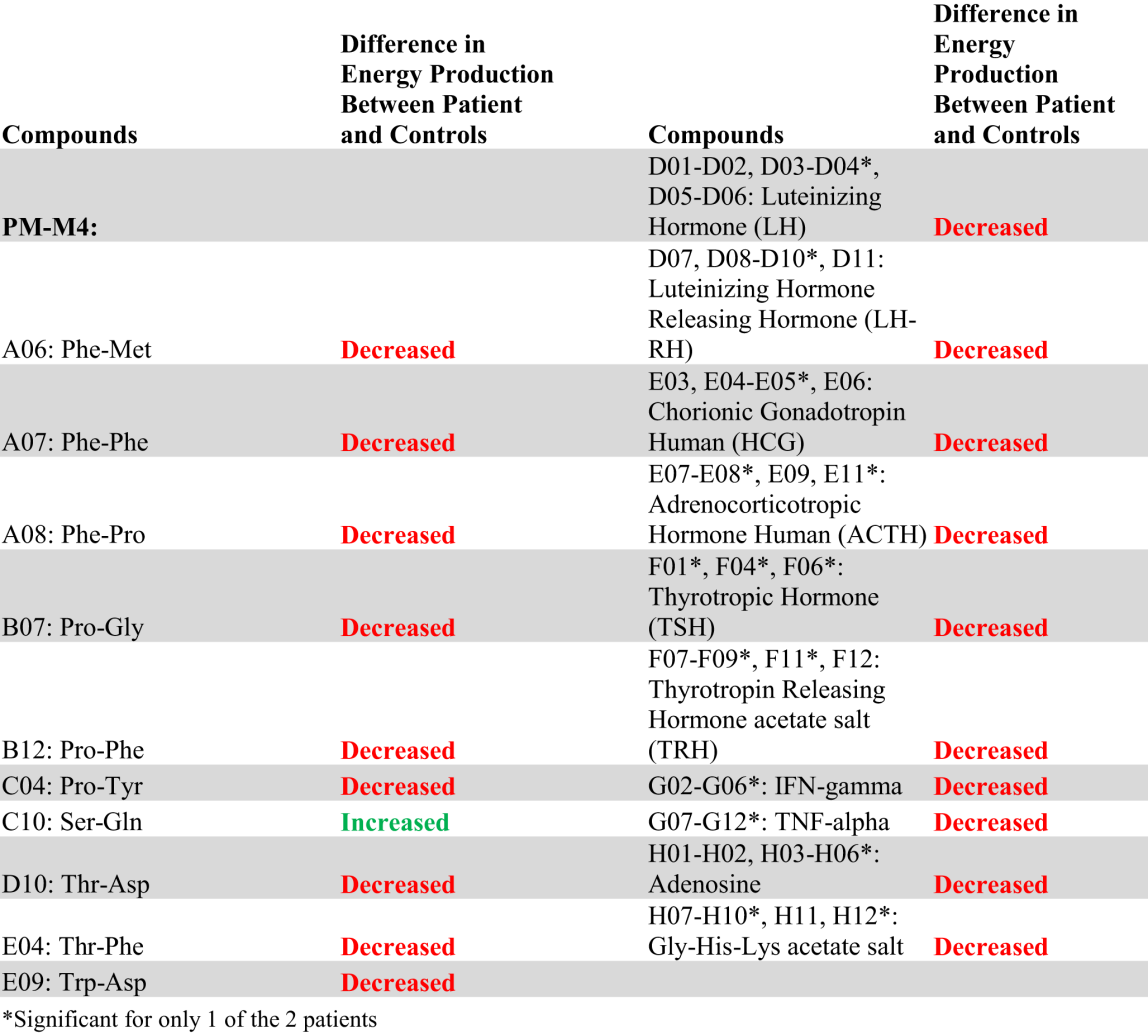
Compounds with a notable visual difference of metabolic activity and energy production by analysis of box-and-whisker plots using the inter-ocular trauma test in (a) the patient with a *MECP2* duplication and (b) the two patients with *MECP2* loss of function variants when compared to a cohort of controls. Green “increased” refers to a higher NADH production in the patient(s) compared to controls, while red “decreased” refers to a lower NADH product in the patient(s) compared to controls. The identifiers of the Biolog plates containing the compounds are indicated in bold

## Discussion

Our study is a “Proof of Concept” that metabolic profiling might highlight common trends at the molecular level that may guide biomarker identification and therapy development for various disorders. We assessed the metabolic profiles of two patients with Rett syndrome (RTT), a neurodevelopmental disorder caused by loss-of-function variants in the *MECP2* gene, versus one patient with *MECP2* duplication syndrome (MRXSL), a disorder caused by gain-of-function duplication of the *MECP2* gene. Correlations between the genetic alterations and the metabolic and clinical phenotypes are based on the premise that the mRNA levels of *MECP2* are decreased in patients with RTT and increased in subjects with MRXSL, as compared to controls. This assumption has been largely validated in the scientific literature, proving that loss-of-function *MECP2* variants are responsible for the disruption of the gene’s function and/or expression in RTT (Gold et al. 2024), while *MECP2* expression is increased in MRXSL LCLs regardless of rearrangement type compared to unaffected control LCLs (Pehlivan et al. 2024). Moreover, restoration of normal *MECP2* mRNA levels is a typical outcome measure for genetic therapy approaches in both RTT (Palmieri et al. 2023) and MRXSL (Rizvi et al. 2024), indicating how disruption of *MECP2* expression is a key pathogenic phenomenon in these disorders. The major metabolic signatures emerging from our study are a generalized reduction of energy production in cells from the two individuals with *MECP2* LOF variants and a large increase in the subject with *MECP2* duplication. Although the two profiles are not determined by the same wells, it is possible to identify key compounds that may be considered candidate biomarkers. For example, all major carbon-based energy sources (PM-M1 plate) cause higher NADH levels with *MECP2* duplication, while only four compounds show the same trend in the cells with *MECP2* loss of function. It is plausible to infer that the lack of *MECP2* fails to exert a deleterious effect on the major metabolic pathways regulating energy production, probably because of various compensatory mechanisms, while the overexpression of the same protein activates a series of downstream pathways requiring an increase of cellular energetic demand. When compared against female controls, the samples from the two female patients with *MECP2* loss of function showed significant reduction in the production of NADH in the presence of several amino acids, revealing interesting clusters involving glycine, a common neurotransmitter in the vertebrate central nervous system, with inhibitory effects in glycinergic synapses in spinal cord and brain stem and excitatory effects (Legendre 2001). Glycine also serves in addition to D-serine as an essential co-agonist of glutamate at NMDAR subtypes of ionotropic glutamate receptors (Eulenburg et al. 2005). Another significant finding emerging from this comparison indicates lower NADH production in the presence of tyrosine, which is used as the chemical precursor of dopamine, an important neurotransmitter associated with the regulation of voluntary movement and a broad array of behavioral processes such as mood, reward, addiction, and stress (Chinta and Andersen 2005). These findings suggest a potentially reduced availability of key molecules in the central nervous system of female individuals with *MECP2* loss of function. The eventual variables that may perturb pathways regulated by MECP2 must be taken into account: assessment of larger cohorts should decrease the risk of unrelated genetic variants biasing the correlation of the metabolic profiles with the various *MECP2*-related disorders.

Another major finding is the reduced response to metabolic effectors in both groups as compared to controls. With the exception of leucine, FGF-1(aFGF), and IL-1beta, associated with higher NADH levels in the sample with *MECP2* duplication, exposure to several hormones and cytokines induced a lower production of energy in both groups, indicating both loss or gain *MECP2* results in a noticeable disruption of the capacity of the cells to adjust to hormonal or immune signals.

Of particular interest is the profile emerging from the response to cytokines: while both groups generated lower NADH than controls in the presence of TNF-alpha and the cells with *MECP2* loss of function showed the same trend with any other cytokine on the Biolog arrays (IL-2, IL-6, IFN-gamma), the response to IL-1beta followed opposite trends in the two groups. Patient 1 harbors a duplication encompassing a region on chromosome X, including the *L1CAM* gene through the *MECP2* gene. *AVPR2*,* ARHGAP4*,* NAA10*,* RENBP*,* HCFC1*,* TMEM187*, and *IRAK1* can be found within this region between the *L1CAM* and *MECP2* genes. *AVPR2*,* NAA10*, and *HCFC1* are associated with disorders inherited in an X-linked recessive fashion, while *ARHGAP4*,* RENBP*,* TMEM187* and *IRAK1* do not have associated syndromes per OMIM. Metabolic profiling of Patient 1 showed an increased response to IL-1beta, the cytokine that binds with IL-1R.

**Fig. 4:**
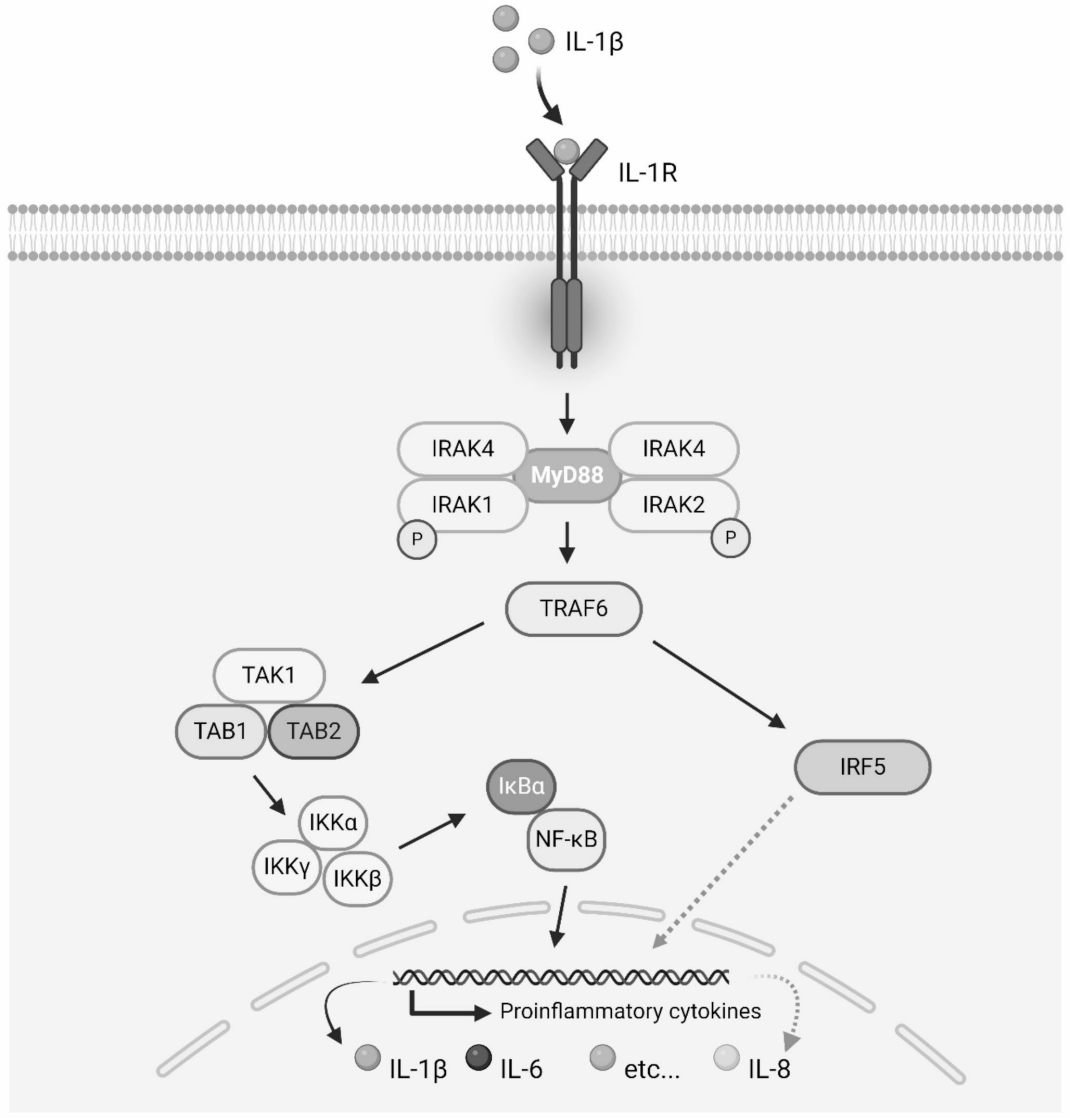
Portion of the canonical NF-κB signaling pathway containing IRAK1 and inflammatory cytokines discussed in this study (IL-1beta, IL-6, and IL-8). Upon binding of IL-1 ligands to the interleukin-1 receptor (IL-1R), inflammatory signaling is activated. MyD88 complexes with IRAK4, which induces the phosphorylation of IRAK1. This triggers further complexing, association, activation, or phosphorylation steps that lead to gene transcription in the nucleus, resulting in the production of inflammatory cytokines such as IL-1beta, IL-6, and IL-8

IRAK1 plays an important role in mediating the responses following the binding of IL-1beta to its receptor. When IL-1beta binds to IL-1R, myeloid differentiation primary response 88 (MyD88) protein is recruited inside the cell membrane. MyD88 then complexes with IRAK4, which induces the phosphorylation of IRAK1. The pathway continues for several additional steps until gene transcription is signaled to induce the creation of more inflammatory cytokines (including IL-6 and IL-8) (Fig. 4), thus leading to a state of autoinflammation in the body (Kanehisa and Goto 2000; Jain et al. 2014; Kanehisa 2019; Gottschalk et al. 2023; Kanehisa et al. 2023).

**Fig. 5:**
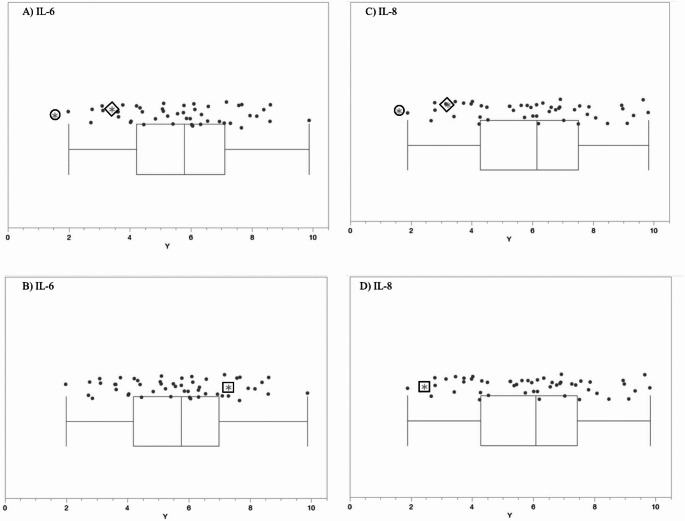
IL-6 and IL-8 box-and-whisker plots using the inter-ocular trauma test, where the circle enclosed * represents the female LOF patient with the nonsense variant, the diamond enclosed * represents the female LOF patient with the missense variant, the square enclosed * represents the male patient with the *MECP2* duplication, and the dots represent the controls. *P*-values < 0.11. Well H04 – IL-6 for (**A**) LOF patients, and (**B**) the patient with the *MECP2* duplication. Well H07 – IL-8 for (**C**) LOF patients, and (**D**) the patient with the *MECP2* duplication

In a recent publication, the production of IL-6 and IL-8 was measured after stimulating fibroblasts, peripheral blood mononuclear cells (PBMCs), and whole blood of patients with MRXSL and healthy controls with IL-1beta. There was no significant difference between patients and controls, showing that *MECP2/IRAK1* duplication does not lead to higher levels of inflammatory cytokines upon stimulation (Gottschalk et al. 2023). The authors suggest that the inflammation seen in patients with MRXSL might either be driven by an infection rather than an autoimmune process, that increased cytokine production might be different in other tissues such as lung epithelia, or that the underlying cause for patients’ inflammation may be due to the duplication of *MECP2* and not *IRAK1.* In the ‘*Mecp2 Dup*’ mouse model developed by Maino et al., IL-6 and IL-8 were not elevated when the mice were infected with influenza H1N1; however, an abnormal immune response was noted as compared to wildtype mice, which included elevation of other proinflammatory cytokines (IFNγ, IL-2, IL-4, IL-13, IL-20, and TNFα among others) (Maino et al. 2024). The lymphoblast cells of our patient with MRXSL showed an increased response to IL-1beta compared to the 50 controls; however, there was no increase in response to IL-6 and IL-8, which aligns with the findings from these previous studies. Moreover, our findings suggest a role for *MECP2* disruption in the response to IL-1beta and other cytokines that might be unrelated to IRAK1 because we noted a decreased response to IL-1beta, IL-6, and IL-8 in cells with *MECP2* LOF but intact *IRAK1*. This potential role of *MECP2* LOF (but not duplication) in immune regulation would be consistent with the lack of abnormal levels of IL-6 and/or IL-8 in individuals with MRXSL (Fig. 5). Whether the trend of increased response to IL-1beta relates to the inflammation seen in patients with MRXSL remains unclear. Nevertheless, exposure to IL-1beta generated opposite metabolic responses in our assays– higher NADH levels in the cells with *MECP2* duplication and lower NADH levels in the cells with *MECP2* loss of function: it is not possible to exclude that the supplemental copy of the *IRAK1* gene may determine such difference by enhancing the cell response to IL-1beta. The close connection between immune response and neuronal development has been validated by several studies, such as the work by Pascual-Alonso et al. 2023 where immune-related genes were downregulated in RTT and upregulated in MRXSL (Pascual-Alonso et al. 2023) and the work by Albizzati et al. 2024 where the authors reported the secretion of IL-6 by *Mecp2* KO cortical astrocytes when cultured together with wildtype neurons (reminiscent of the typical heterozygous or mosaic nature of pathogenic variant distribution across tissues of individuals with RTT). These findings suggest a deleterious effect on neurons (i.e., synaptogenesis) from IL-6 response in patients with RTT (Albizzati et al. 2024). A connection between immune activity and neuronal function mediated by *MECP2* has been suggested by Frasca et al. (2024), who noted that IFNγ is able to rescue synaptic defects, as well as motor and cognitive alterations in *Mecp2*-deficient models.

The utilization of amino acids and dipeptides as energy sources is vastly altered in all patients, indicating that abnormal metabolism of these compounds may mediate some of the pathogenic mechanisms underlying the clinical presentation of *MECP2*-associated disorders. This is consistent with recent metabolomic studies on human and mouse models for Rett syndrome (Golubiani et al. 2021; Illescas et al. 2023) and with alterations of amino acid metabolism detected by the same approach in autism spectrum disorder (Cascio et al. 2020).

The phenotypic traits reported in the three patients of this study are common in various neurodevelopmental disorders and fail to highlight peculiar features that could be pathognomonic for either *MECP2* gain or loss. Moreover, MRXSL, RTT, and all other *MECP2*-related disorders lack peculiar metabolic abnormalities or laboratory findings. Therefore, it is not possible to establish clear genotype-phenotype correlations corroborated by our findings. Nevertheless, we can infer that specific metabolic signatures, like the abnormal metabolism of amino acids and the disrupted response to hormones, may be involved in some pathogenic mechanisms and play a role in some *MECP2*-associated features. For example, several wells containing the amino acid glutamic acid (or glutamate) showed reduced NADH production in both RTT and MRXSL samples; glutamate acts as a neurotransmitter in the central nervous system, and alterations in its metabolism may be associated with abnormal development of certain brain areas or seizures. Such metabolic abnormalities indicate that altered MECP2 levels may affect a plethora of pathways and the related pathogenic mechanisms may spread beyond the central nervous system, which is associated with the most characteristic clinical features of *MECP2*-related disorders: abnormal utilization of amino acid or unbalanced immune activities hint towards more systemic issues, as suggested by other studies as well (Cascio et al. 2020; Frasca et al. 2024).

Investigation of the interaction between IL-1R and MECP2 has revealed a very tight relationship in both in vitro and in vivo models, with IL-1R-mediated stimulation leading to an increase of MeCP2 levels and eventually to disruption of dendritic spine morphology, synaptic plasticity and plasticity-related gene expression (Tomasoni et al. 2017; Li Puma et al. 2023). Both studies suggest that the IL-1R inhibitor Anakinra corrects transcriptional changes, restores MeCP2 levels and spine plasticity, and ameliorates cognitive defects: these conclusions are consistent with the trend we observed in cells with *MECP2* duplication, where it is plausible to infer that the extra copies of both *MECP2* and *IRAK1* amplify the response to IL-1beta and exert a synergistic disruptive effect on MECP2 levels, exacerbating the consequent deleterious effects on synapses. For these reasons, we would suggest that Anakinra-based treatments in individuals with MRXSL may ameliorate the symptoms mediated by an abnormal response to IL-1beta and possibly compensate to some extent for synaptic imbalance.

After validation from larger cohorts, metabolic profiling of *MECP2*-related disorders may integrate diagnostic/screening protocols to assess the functional effect of genetic alterations classified as variants of uncertain significance, increasing the diagnostic yield of such protocols and providing more precise characterization of the genetic and metabolic features of children with *MECP2*-related disorders even before the complete onset of the clinical phenotype.

Another potential translational application of the results of this study involves the use of the drug trofinetide, which has recently been approved by the FDA for the treatment of individuals with RTT (Harris 2023; Hudu et al. 2023; Neul et al. 2023). This drug is derived from an endogenous tripeptide of the N-terminal domain of Insulin-like Growth Factor 1 (IGF-1) (Parent et al. 2023) and therefore identification of abnormal cellular responses to IGF-1 – as detected in both our groups of patients – may offer the opportunity of selecting best and/or worst responder to the treatment and monitor the efficacy of the drug during the therapeutic protocol.

This study comes with its limitations. Due to the small number of patients included, statistical significance was not reached by standard methods, prompting further analysis via alternative methods. Therefore, we view this study as a “Proof of Concept” that metabolic profiling can highlight trends in genetic disorders to guide further research or treatment discovery. We caution generalizing the findings of this study to every subject with *MECP2*-related disorders, considering the potential contributions of other genetic and environmental factors, such as X inactivation rate in female patients with RTT or variability in metabolic responses to drugs or inflammation. Additionally, we do not have results on the two patients with RTT for plate PM-M5 due to the lack of specimens. This plate, which contains ions, may have yielded additional significant findings to further contribute to the metabolic profile of patients with RTT. One may also note a drawback of using cultured lymphoblastoid cell lines in that the transformation may erase some of the original characteristics of the cells; however, this may actually be an advantage to our study. By transforming cells, potential confounding factors that may impact other metabolomic studies (i.e., age, diet, drugs. etc.) are erased, allowing all results to be directly linked to the genotype alone. Another important limitation related to the utilization of lymphoblastoid cells is that the expression and metabolic profiles change in different tissues and, therefore, may not reflect faithfully the cellular activities in the central nervous system, which is mostly affected in individuals with *MECP2*-associated disorders. However, some of our most interesting findings are connected to the immune system, to which belong the lymphocytes collected for this study. Moreover, the PM-M technology has been successfully employed in the functional investigation of other neurodevelopmental disorders (Cascio et al. 2020), and it may highlight metabolic abnormalities in blood-derived cells that could be used as biomarkers since these cells are more accessible than neural tissue.

The results of this study should be validated with a larger cohort to confirm the metabolic profiles seen and identify optimal targets for interventions. Other future studies could include molecular quantification of the energy production as well as directly measuring the levels of inflammatory cytokines, such as IL-1beta, IL-6, and IL-8, in individuals with RTT and MRXSL.

## Conclusions

Despite these technical limitations, the results of the study bear remarkable potential for translational applications. The metabolic signatures detected in cells with abnormal MECP2 levels can pave the way to the development of biomarkers for early screening of individuals at risk or presenting with clinical features suggestive of *MECP2*-related disorders. Abnormalities in metabolic response to specific compounds unveil novel pathogenic mechanisms that can possibly explain the phenotypical variability associated with *MECP2* alterations. Finally, the compounds inducing significant metabolic differences could be targeted for the development of novel therapeutic approaches or be used to monitor the efficacy of existing treatments. Metabolic profiling may offer numerous possibilities for a deeper investigation of *MECP2*-related disorders and other neurodevelopmental disorders, as well as for developing more efficient diagnostic/screening protocols and treatment approaches that are more adherent to precision medicine goals.

## Electronic supplementary material

Below is the link to the electronic supplementary material.

Supplementary Material 1

Supplementary Material 2

Supplementary Material 3

Supplementary Material 4

Supplementary Material 5

Supplementary Material 6

Supplementary Material 7

Supplementary Material 8

Supplementary Material 9

Supplementary Material 10

Supplementary Material 11

Supplementary Material 12

Supplementary Material 13

Supplementary Material 14

Supplementary Material 15

Supplementary Material 16

Supplementary Material 17

Supplementary Material 18

Supplementary Material 19

Supplementary Material 20

Supplementary Material 21

Supplementary Material 22

Supplementary Material 23

## Data Availability

The datasets generated during and/or analyzed during the current study are available in the supplementary information.
