# Supplementary figures and images for: A novel approach to metabolic profiling in case models of MECP2-related disorders
Source: Metab Brain Dis. 2025 Feb 13;40(2):124. doi: 10.1007/s11011-025-01546-5 (PMC11825590; doi:10.1007/s11011-025-01546-5)

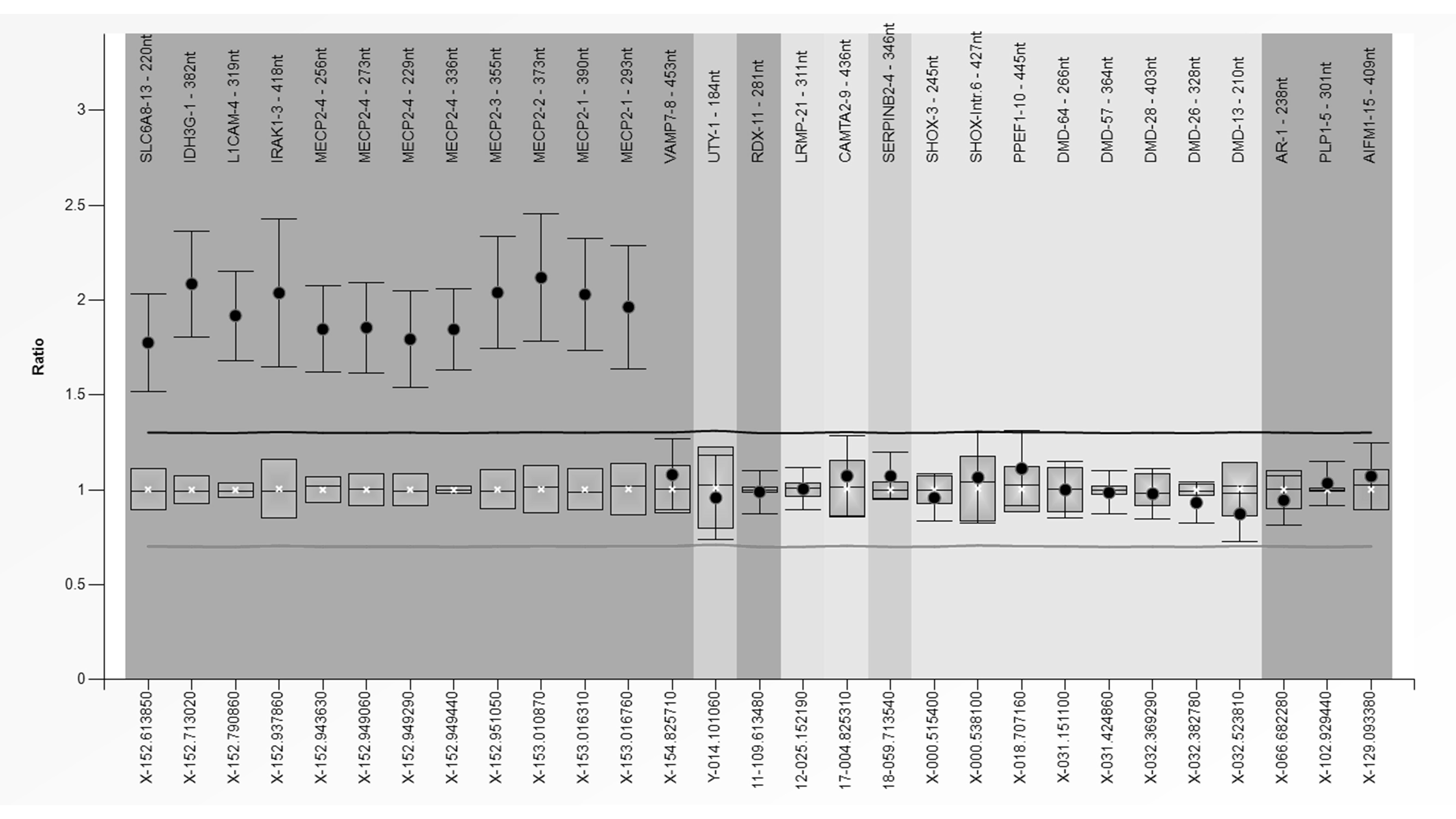

Supplement: Supplementary file 6 — Supplementary Material 6 [file 11011_2025_1546_MOESM6_ESM.jpg]
