# Supplementary material for: A novel approach to metabolic profiling in case models of MECP2-related disorders
Source: Metab Brain Dis. 2025 Feb 13;40(2):124. doi: 10.1007/s11011-025-01546-5 (PMC11825590; doi:10.1007/s11011-025-01546-5)

Graph Builder

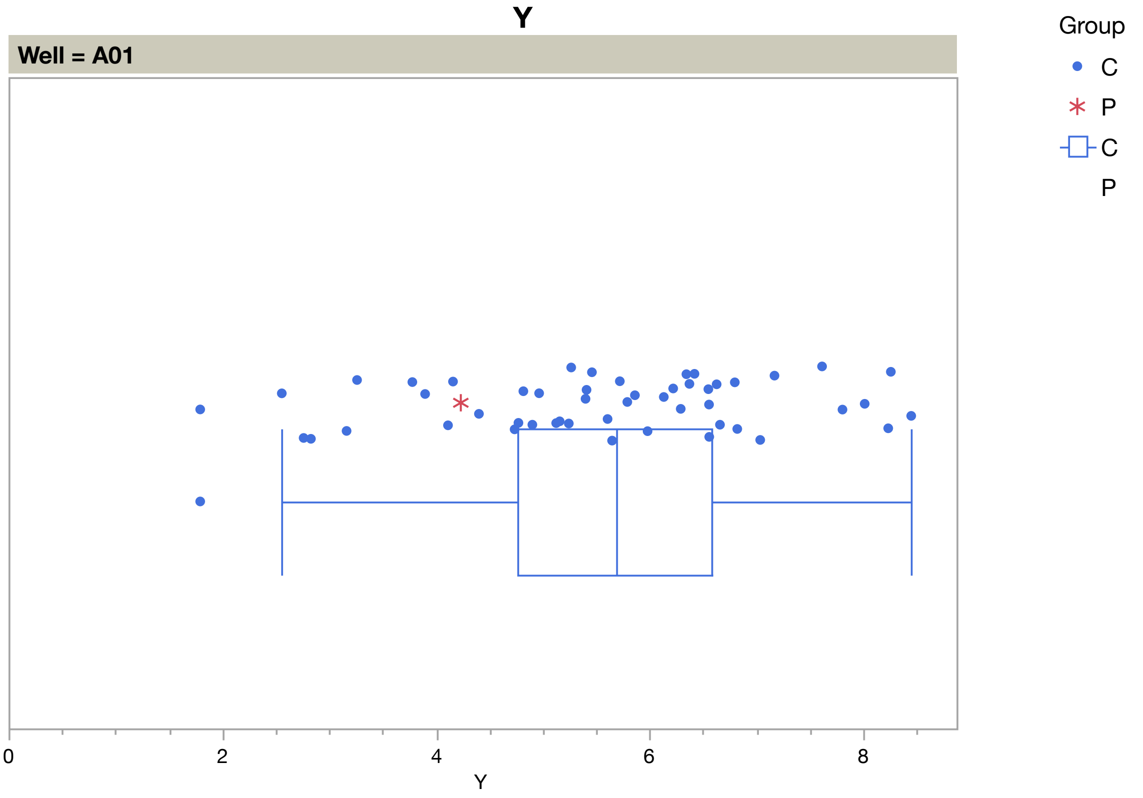

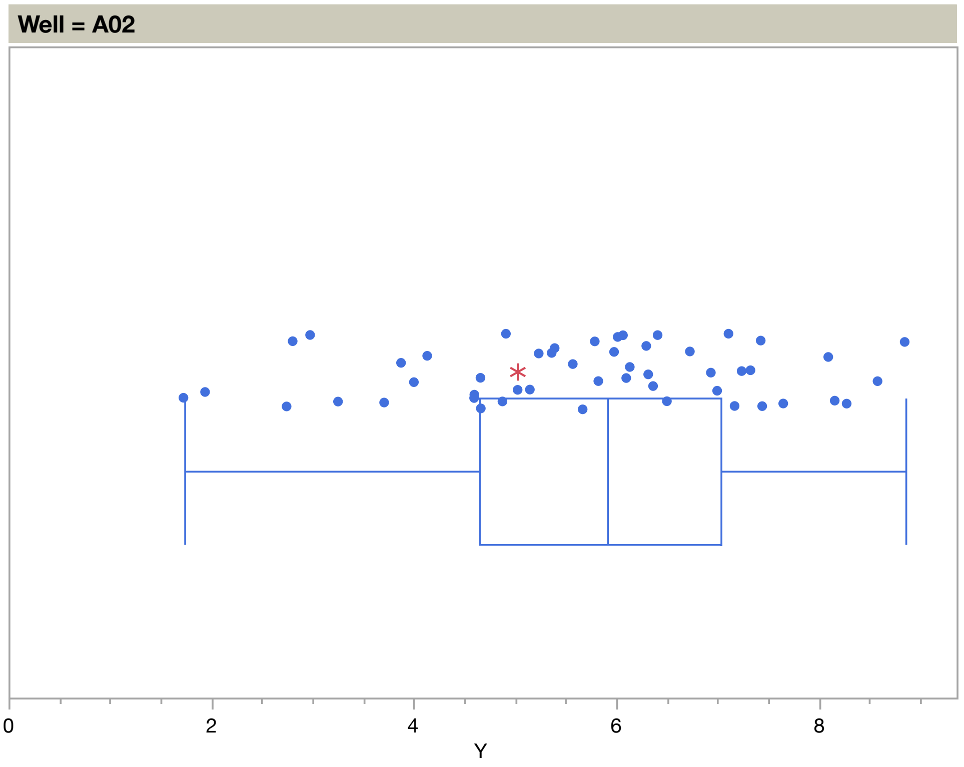

Well = A03

Graph Builder

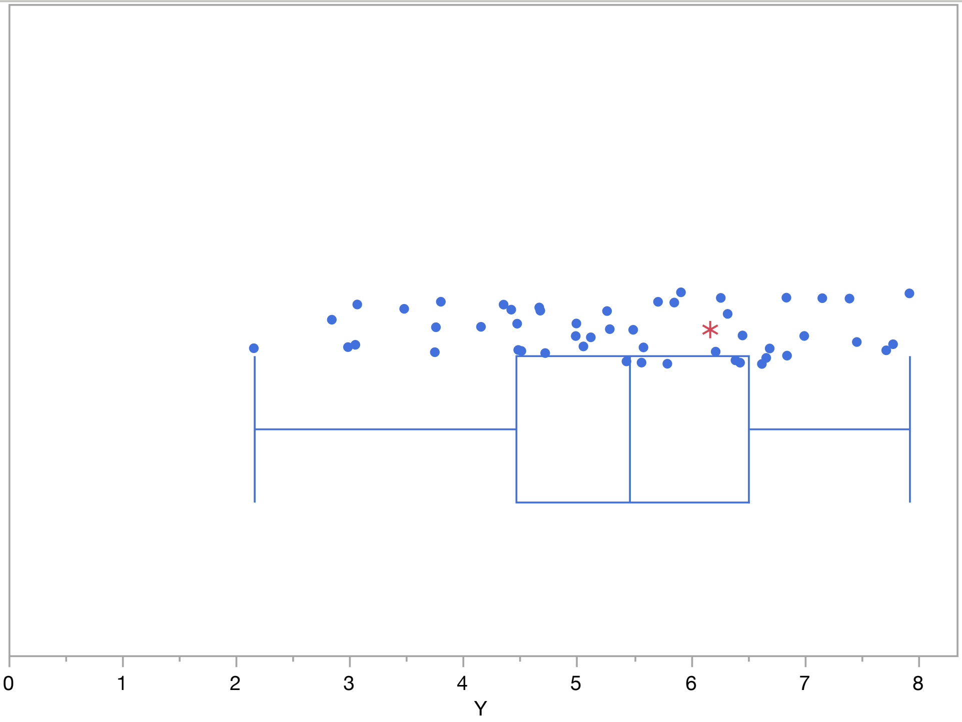

Well = A04

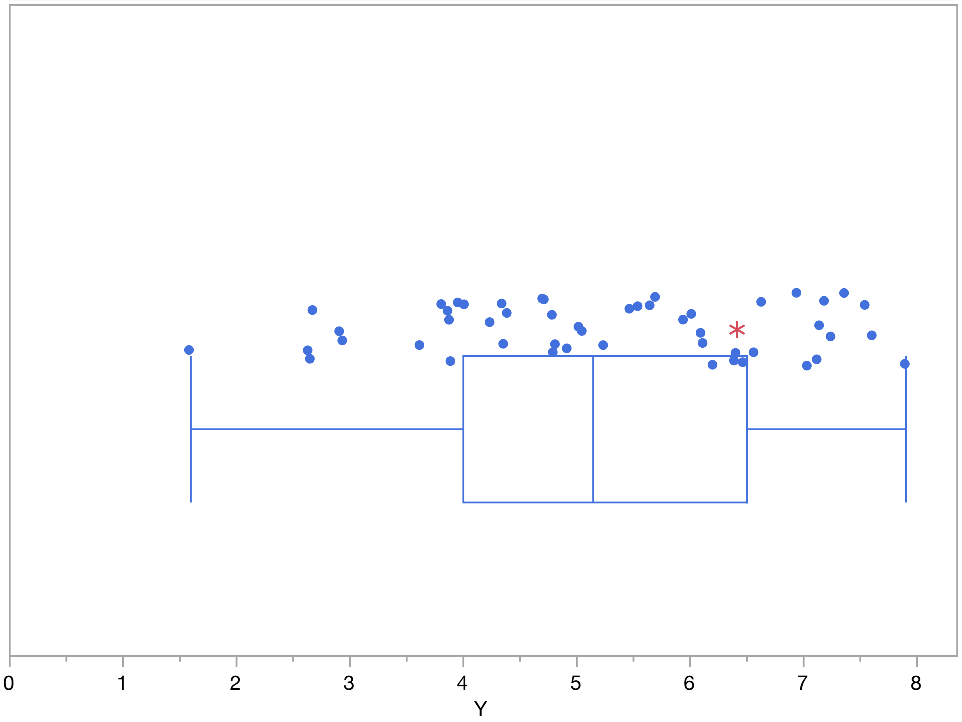

Well = A05

Graph Builder

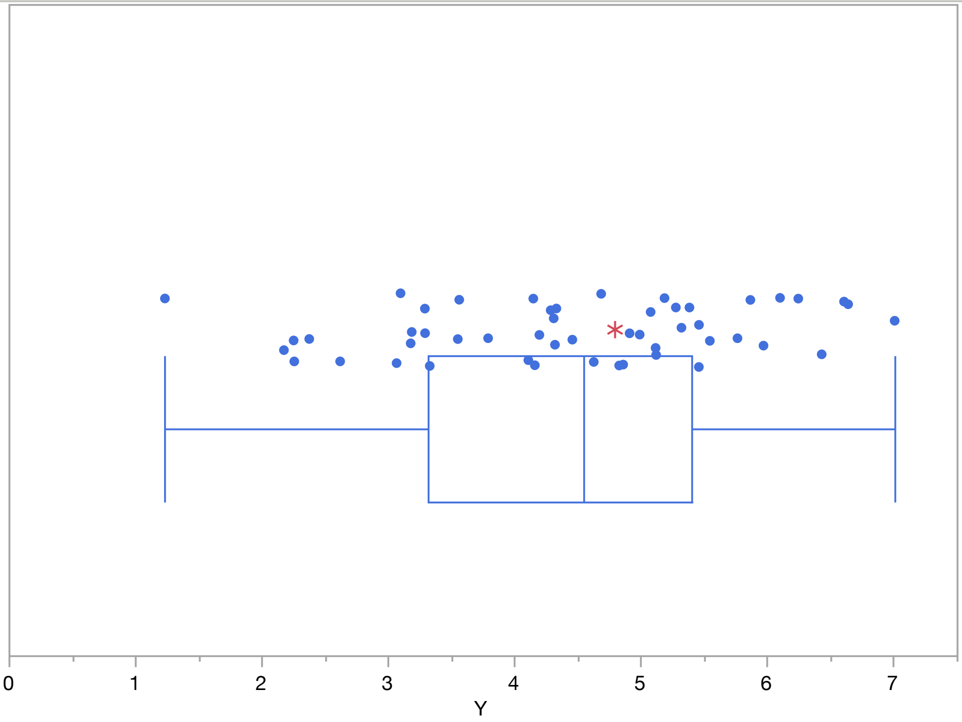

Well = A06

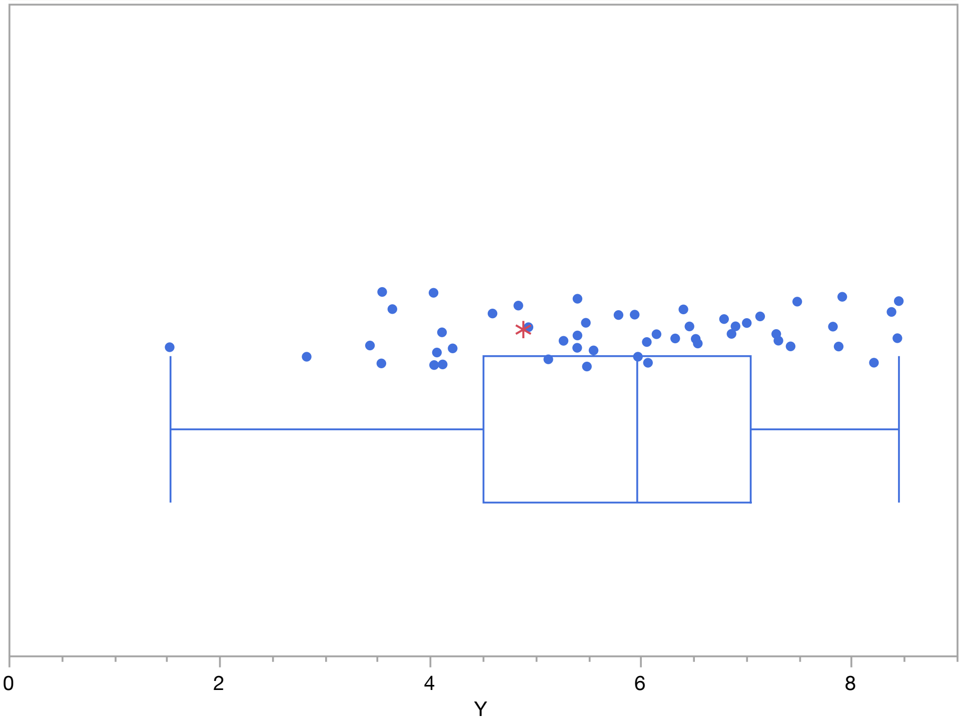

Well = A07

Graph Builder

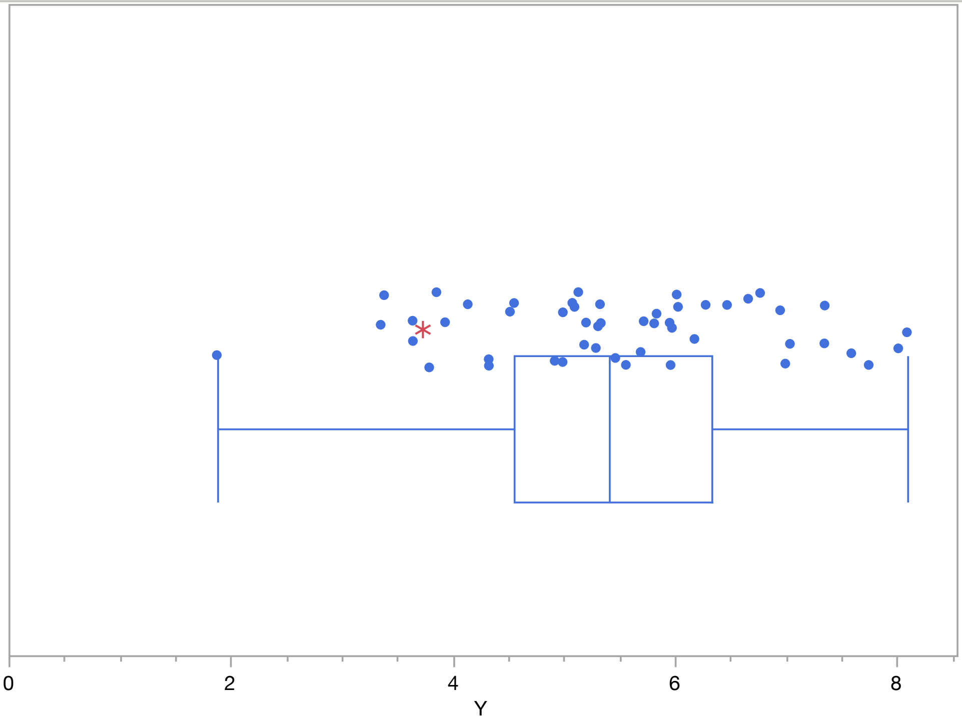

Well = A08

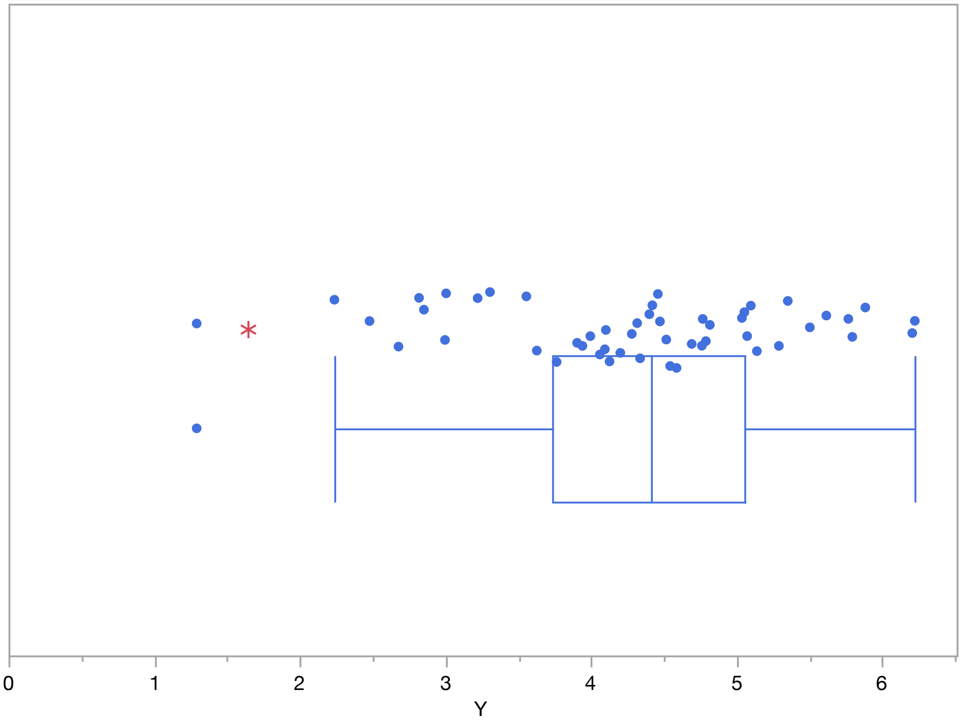

Well = A09

Graph Builder

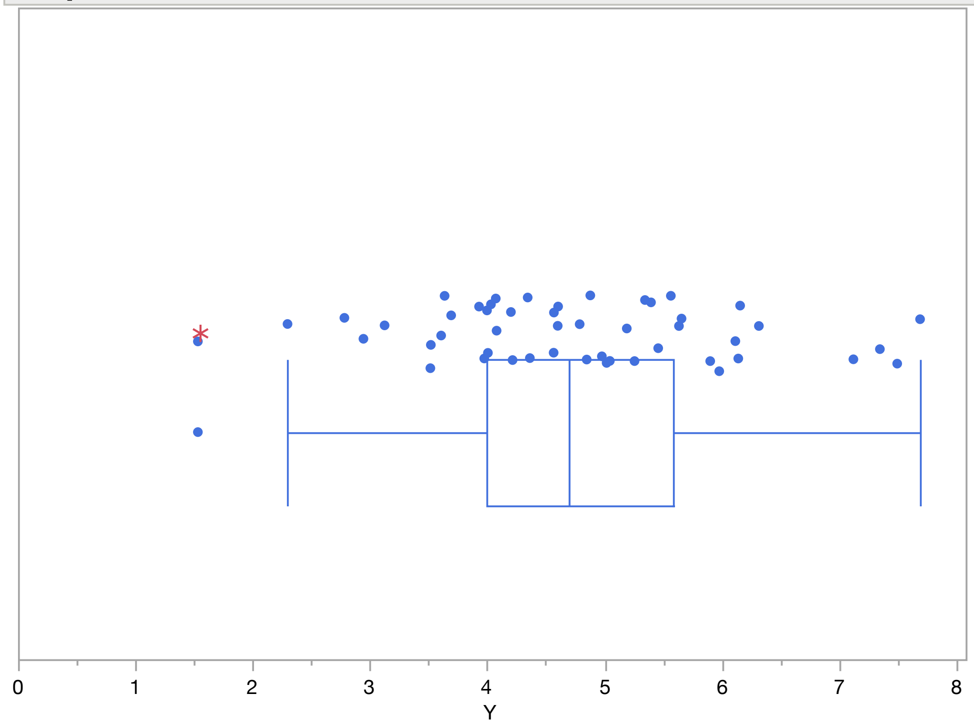

Well = A10

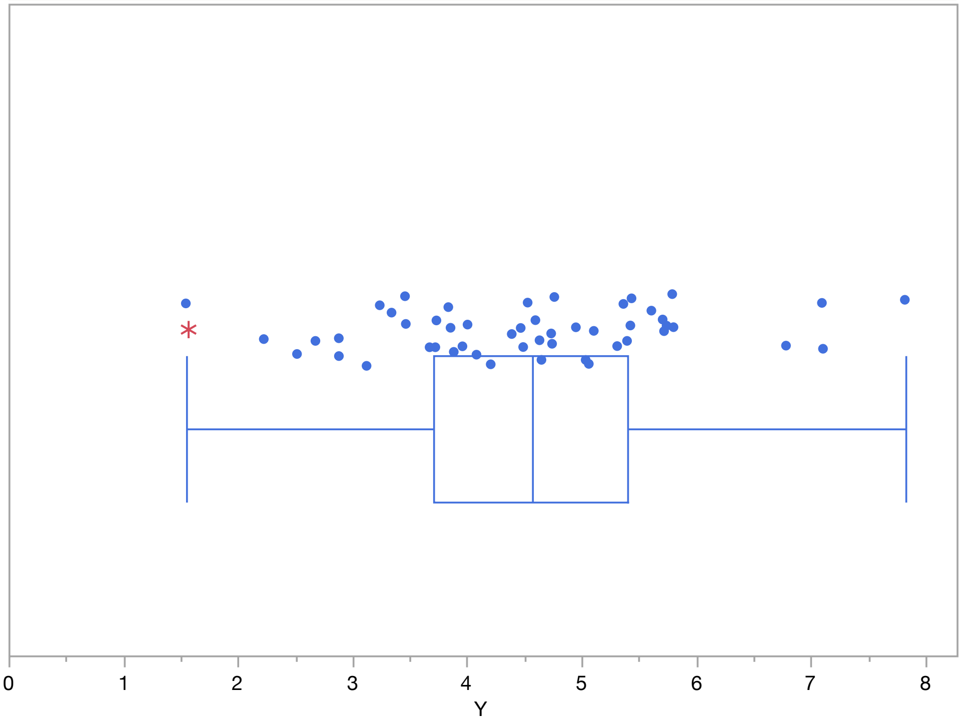

Well = A11

Graph Builder

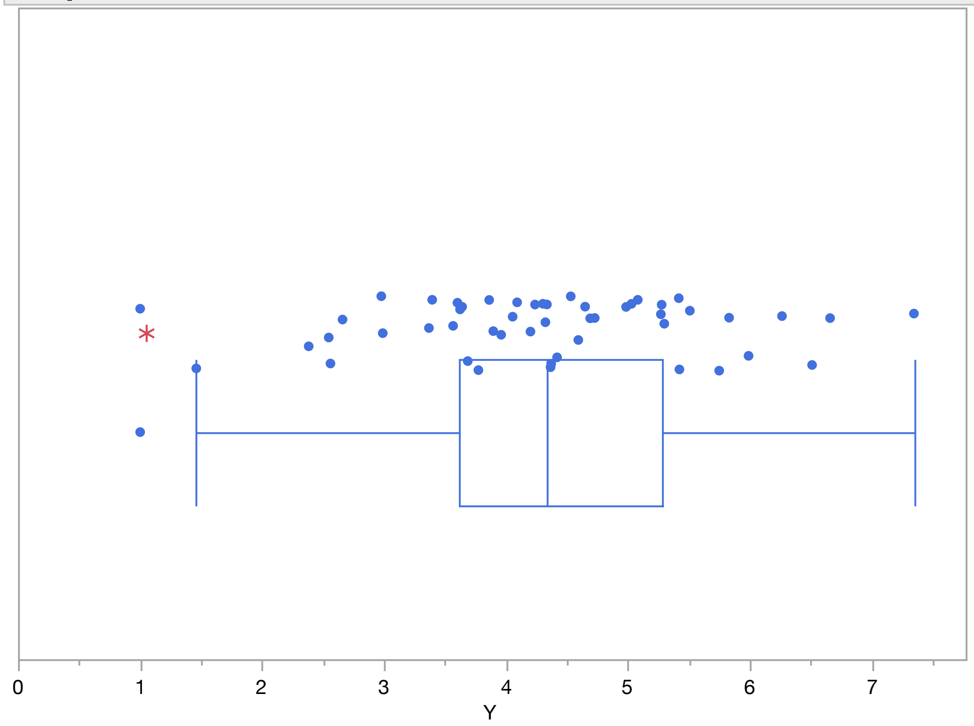

Well = A12

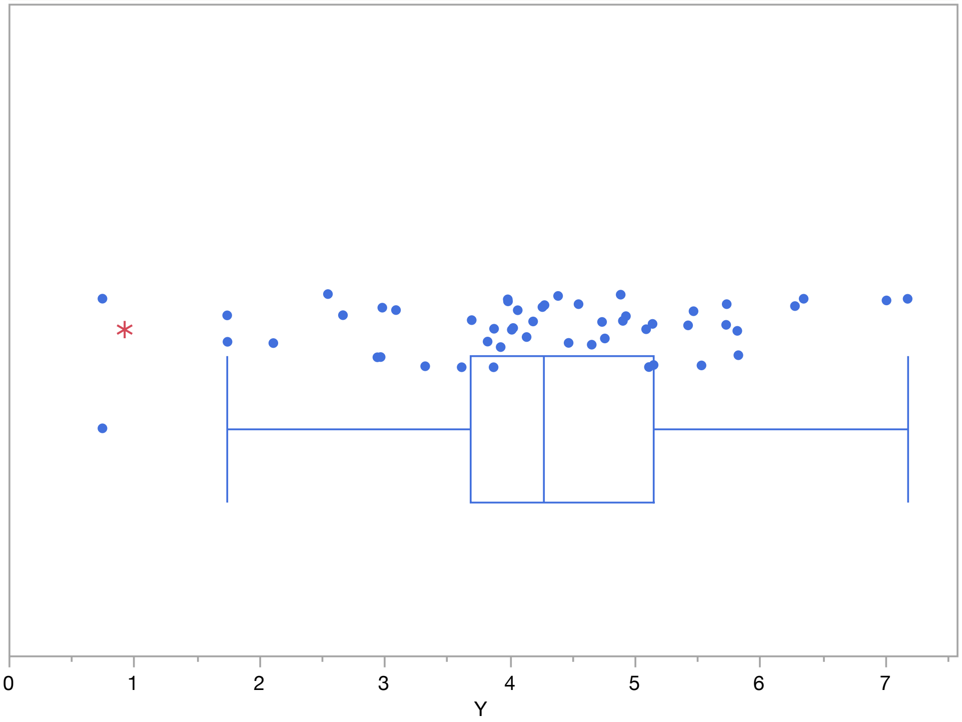

Well = B01

Graph Builder

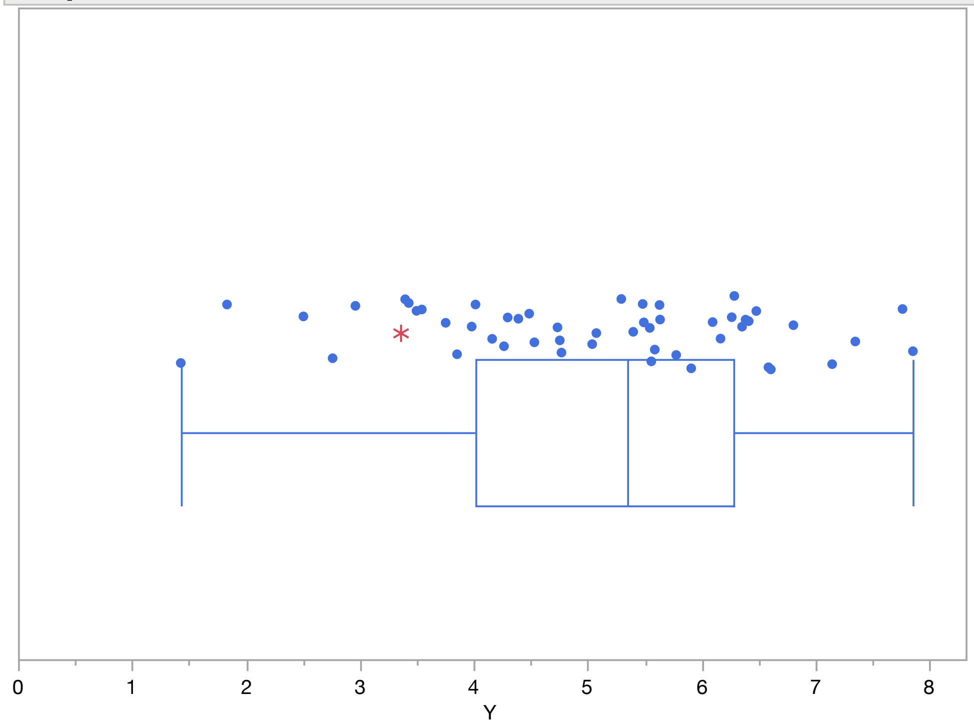

Well = B02

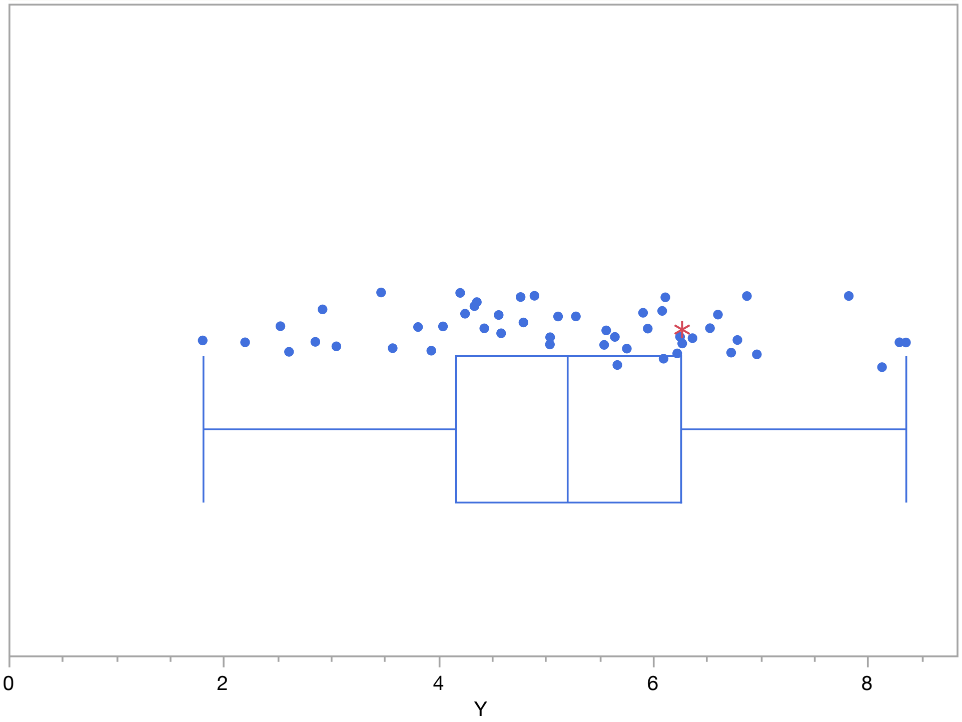

Well = B03

Graph Builder

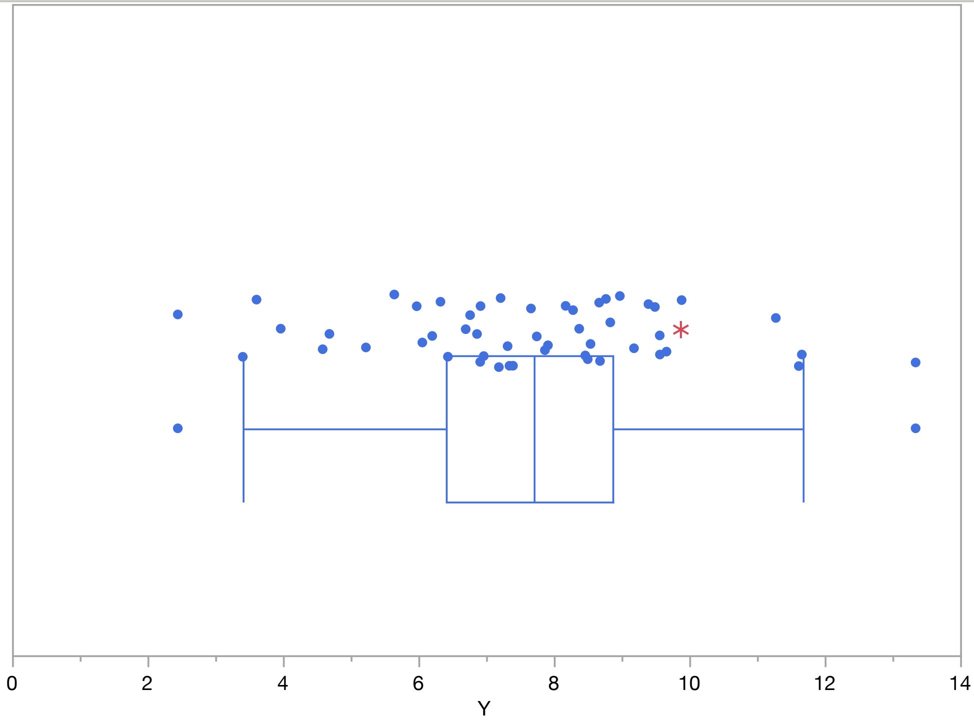

Well = B04

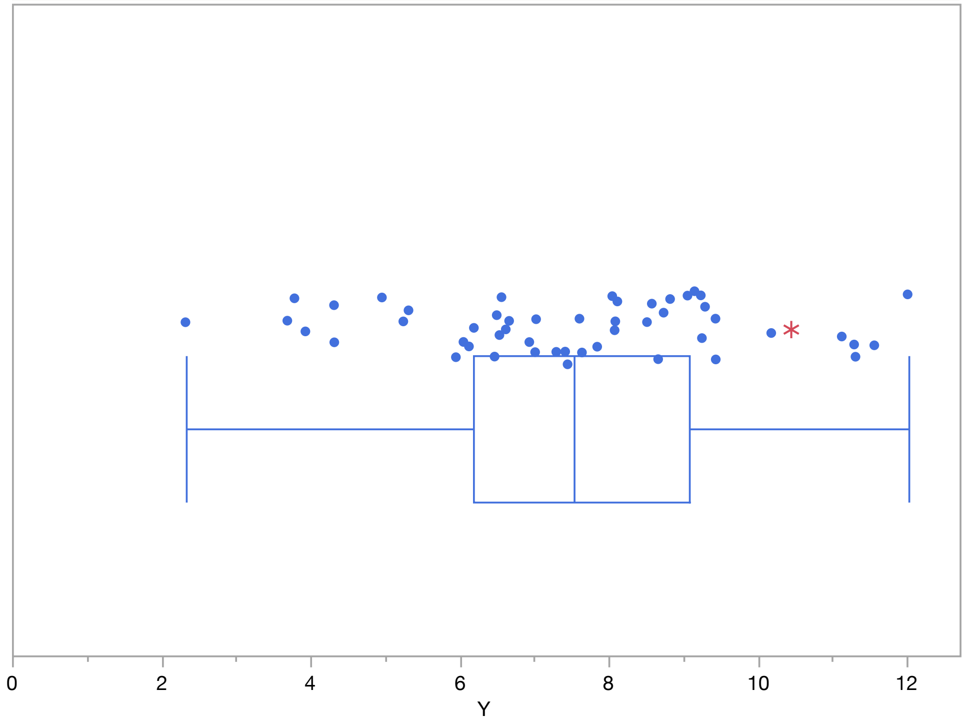

Well = B05

Graph Builder

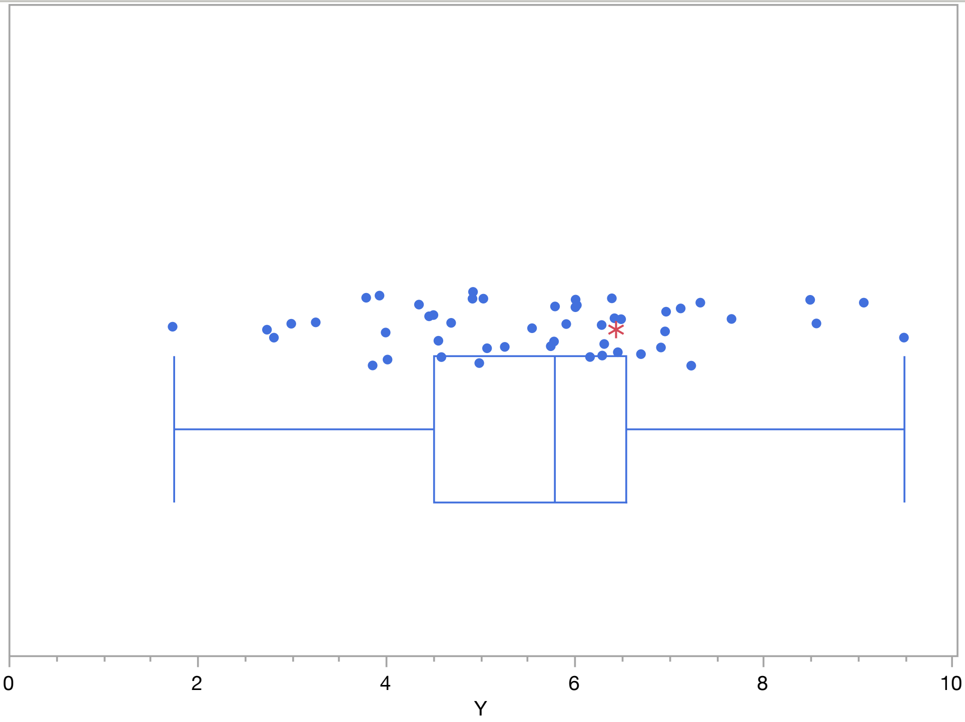

Well = B06

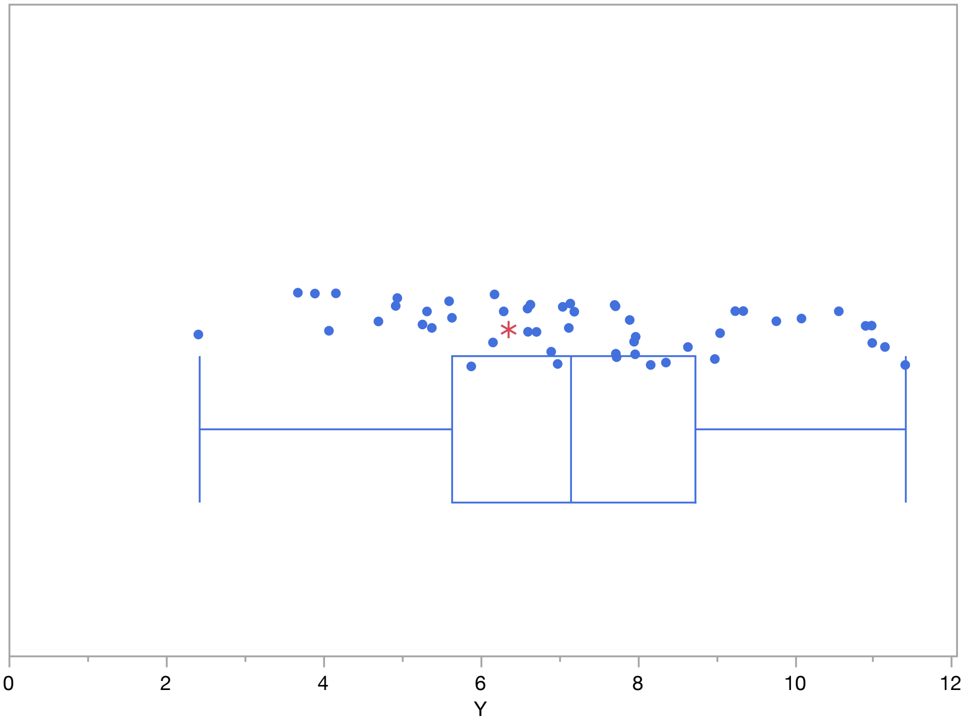

Well = B07

Graph Builder

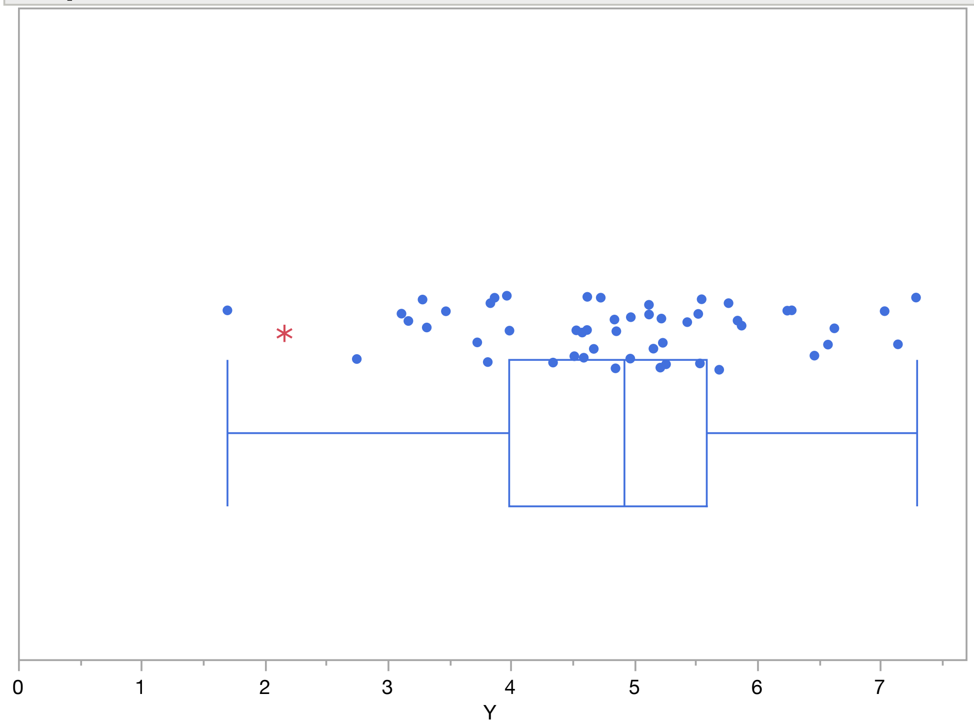

Well = B08

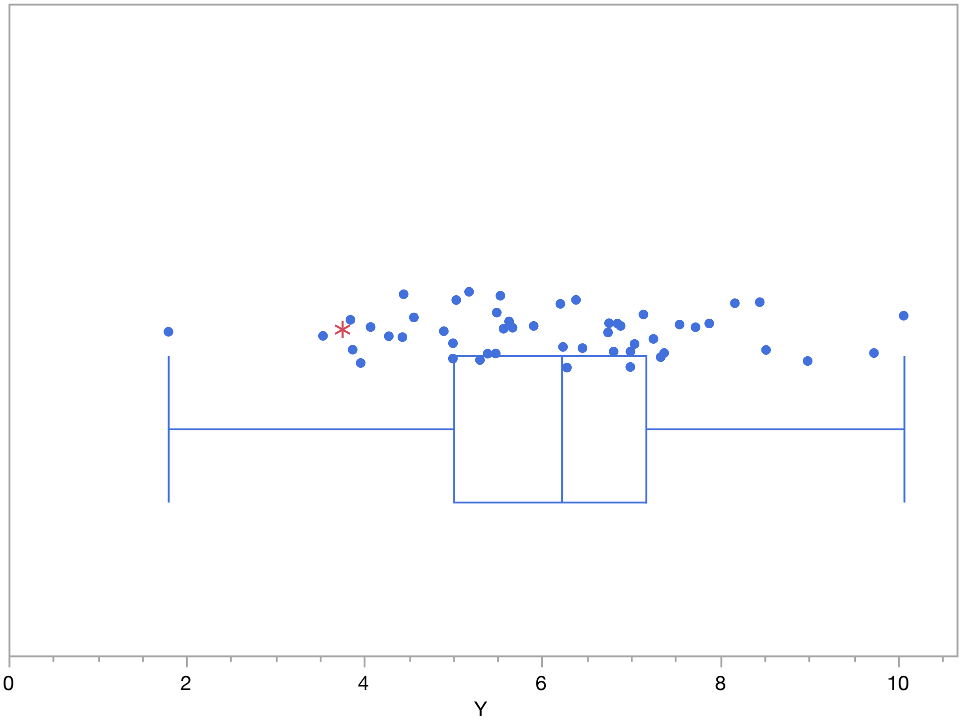

Well = B09

Graph Builder

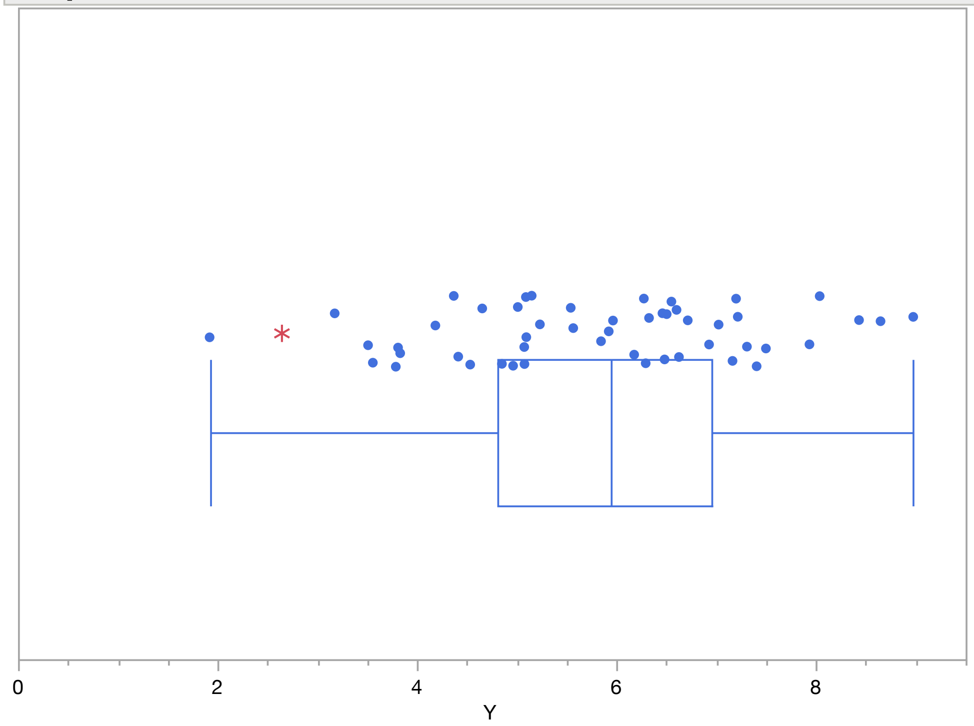

Well = B10

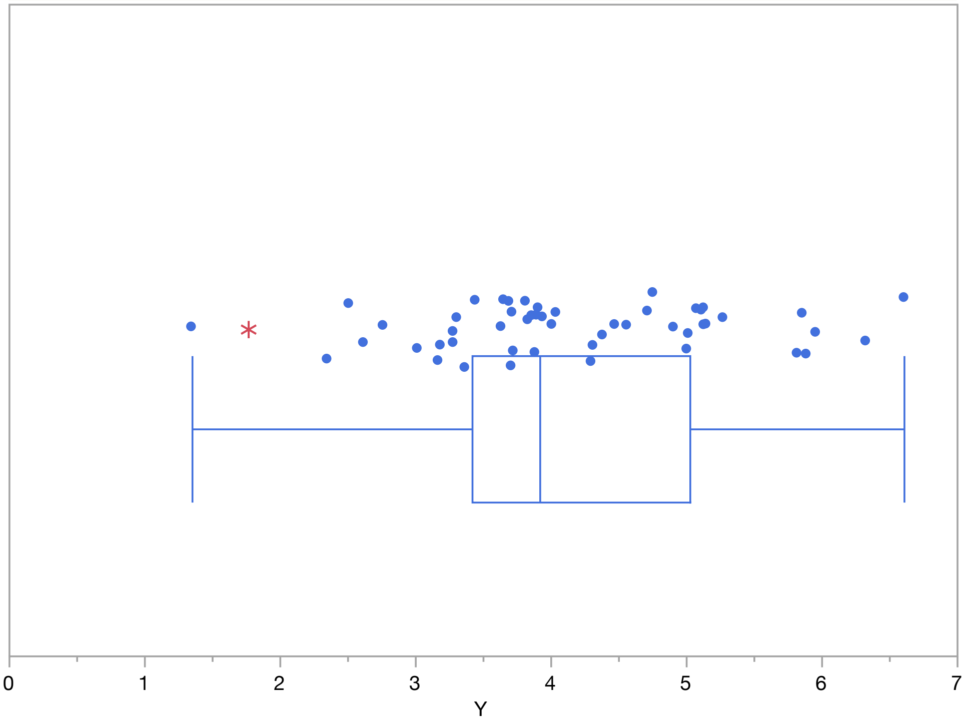

Well = B11

Graph Builder

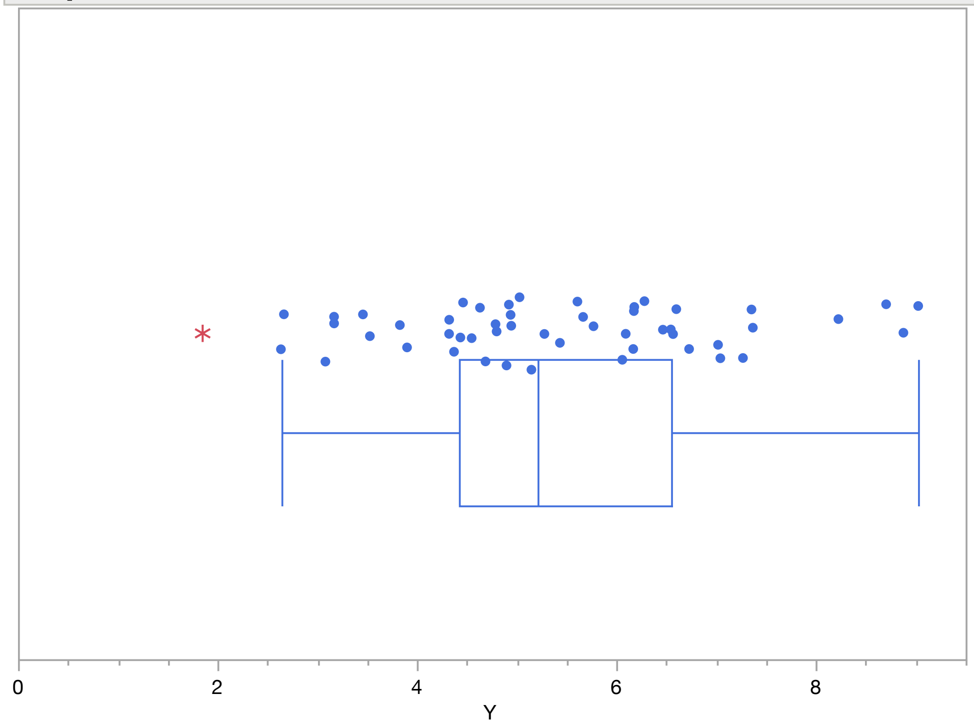

Well = B12

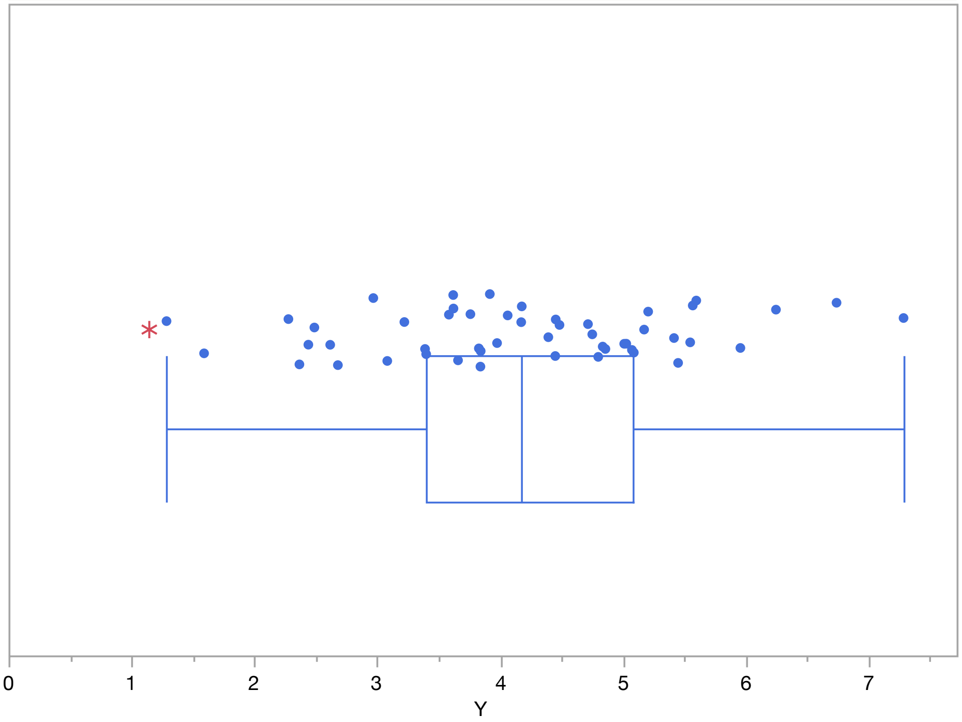

Well = C01

Graph Builder

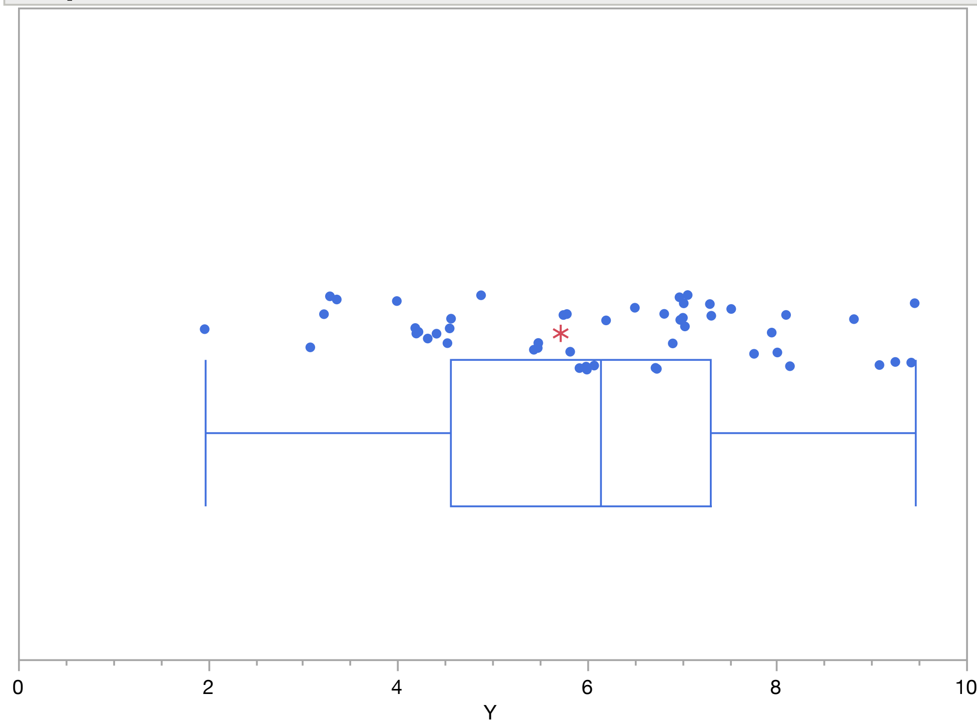

Well = C02

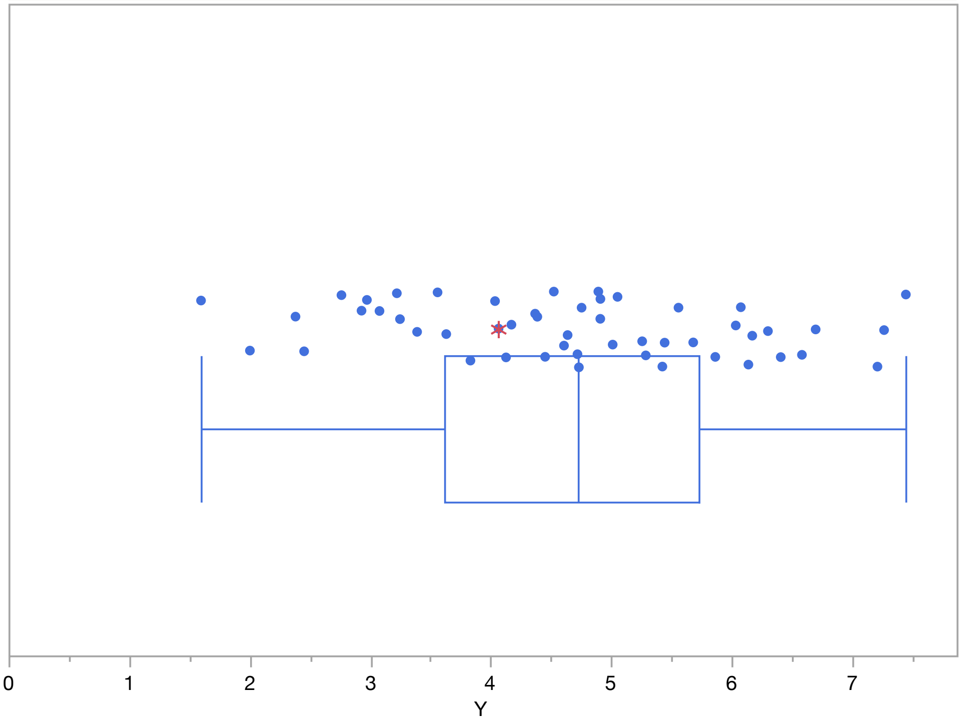

Well = C03

Graph Builder

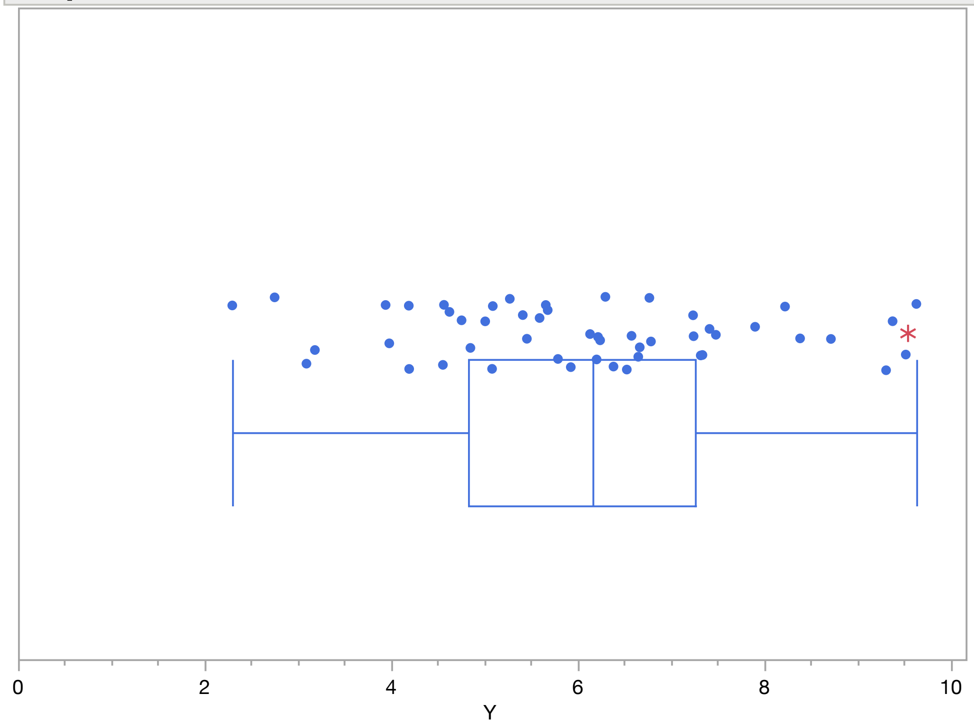

Well = C04

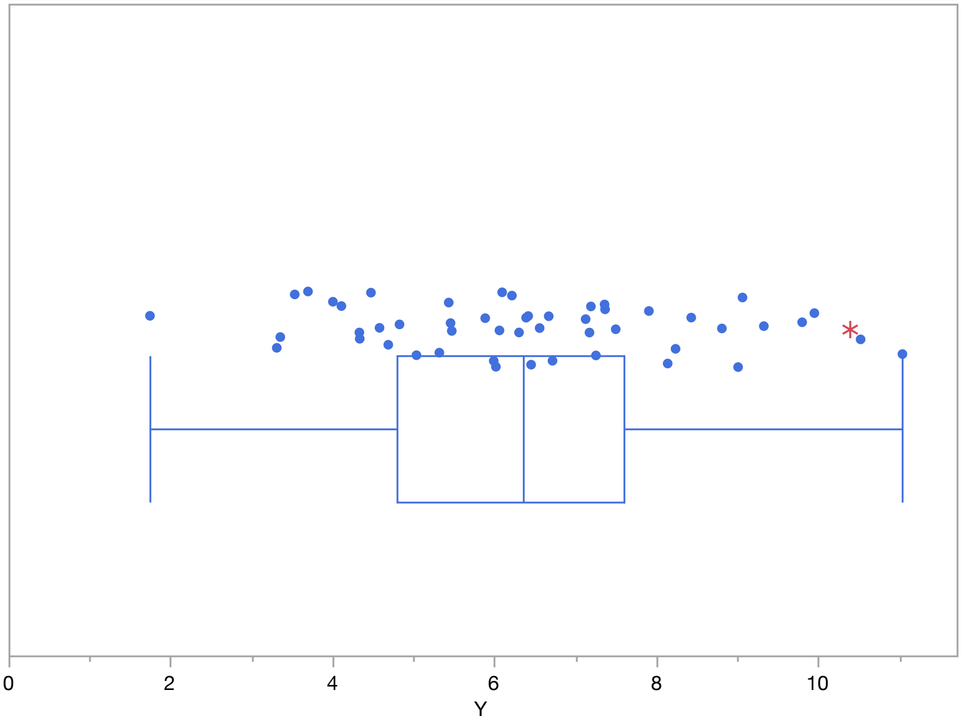

Well = C05

Graph Builder

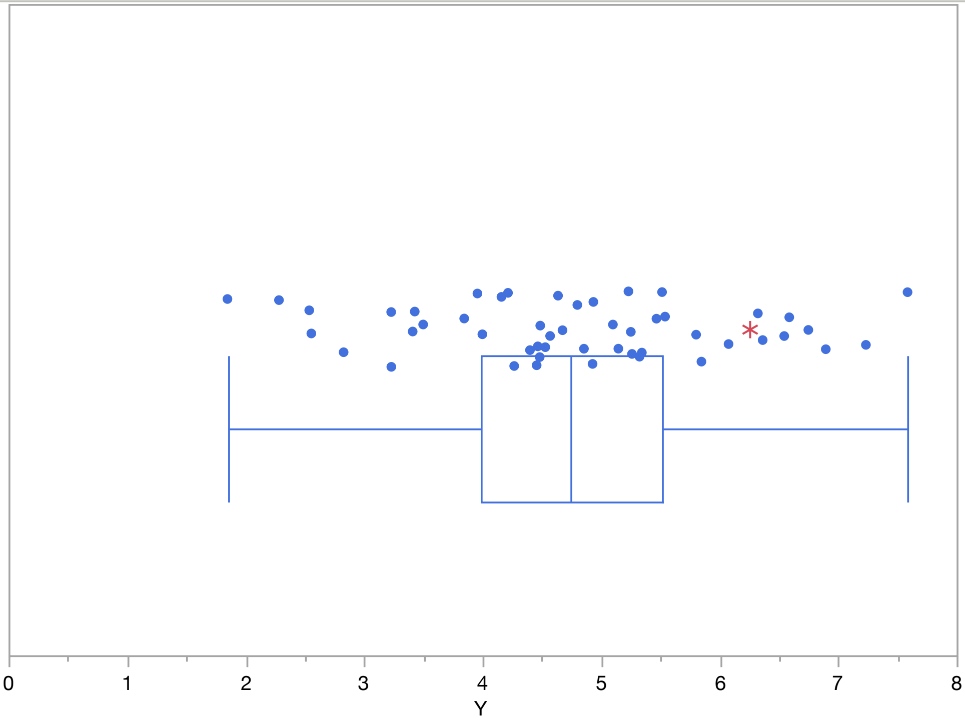

Well = C06

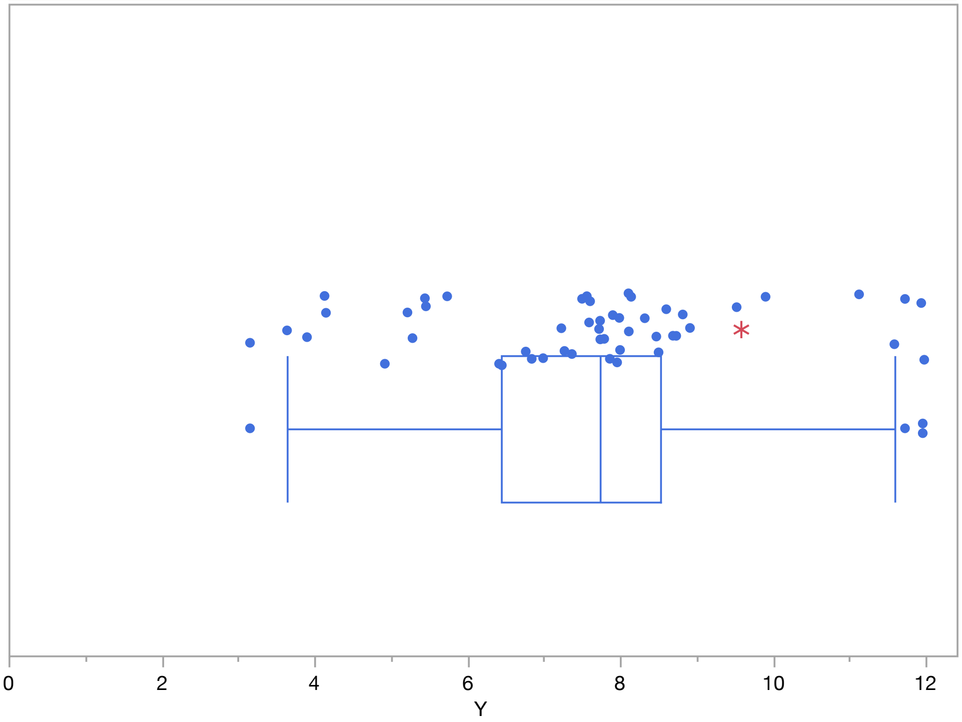

Well = C07

Graph Builder

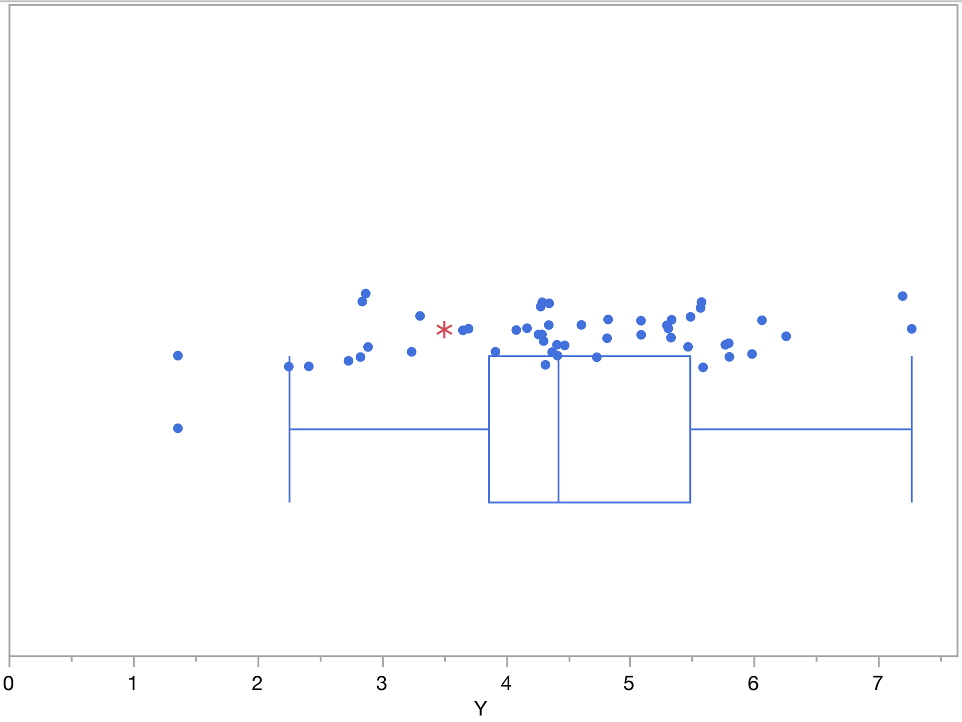

Well = C08

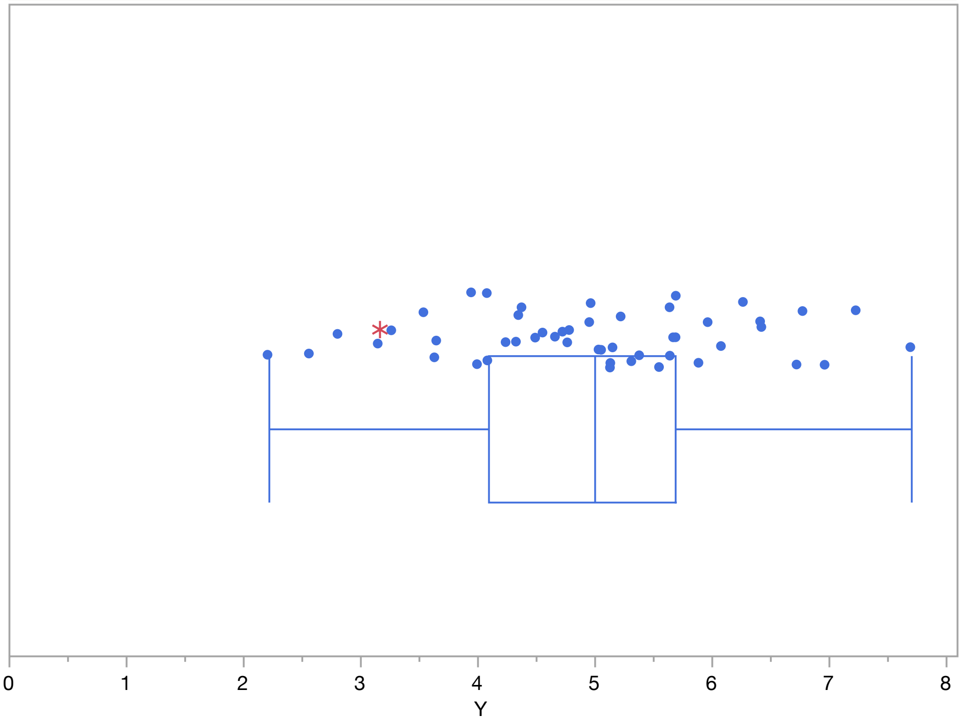

Well = C09

Graph Builder

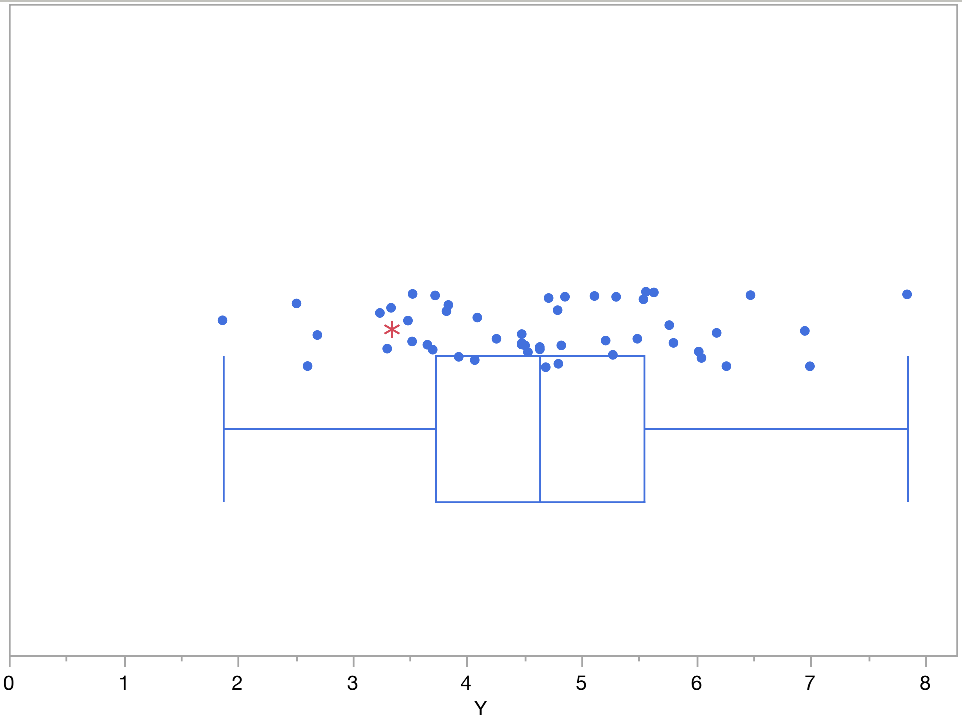

Well = C10

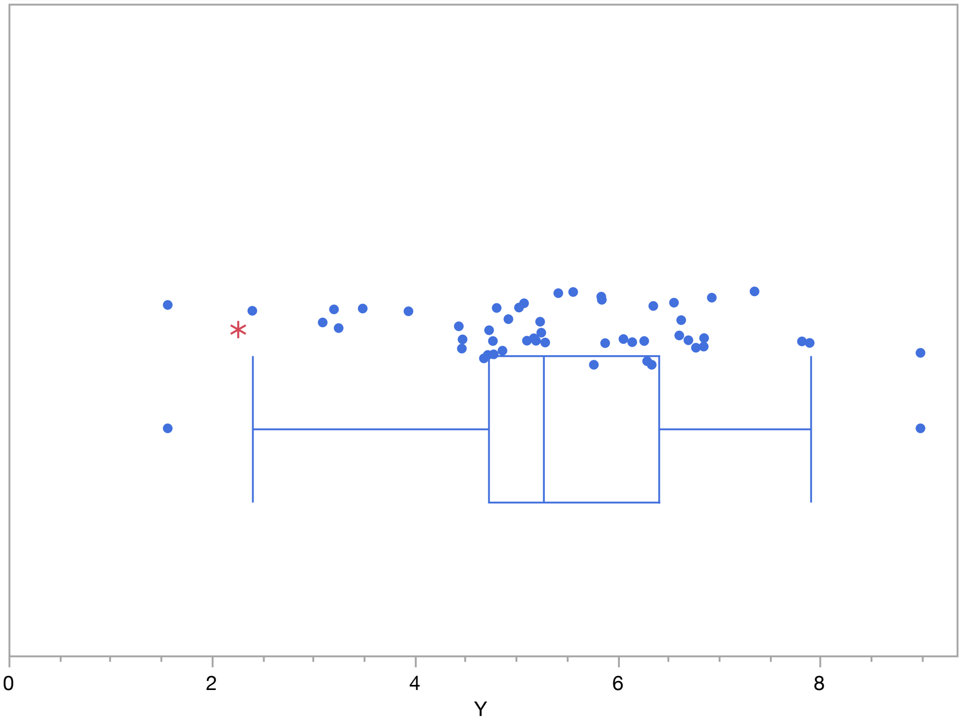

Well = C11

Graph Builder

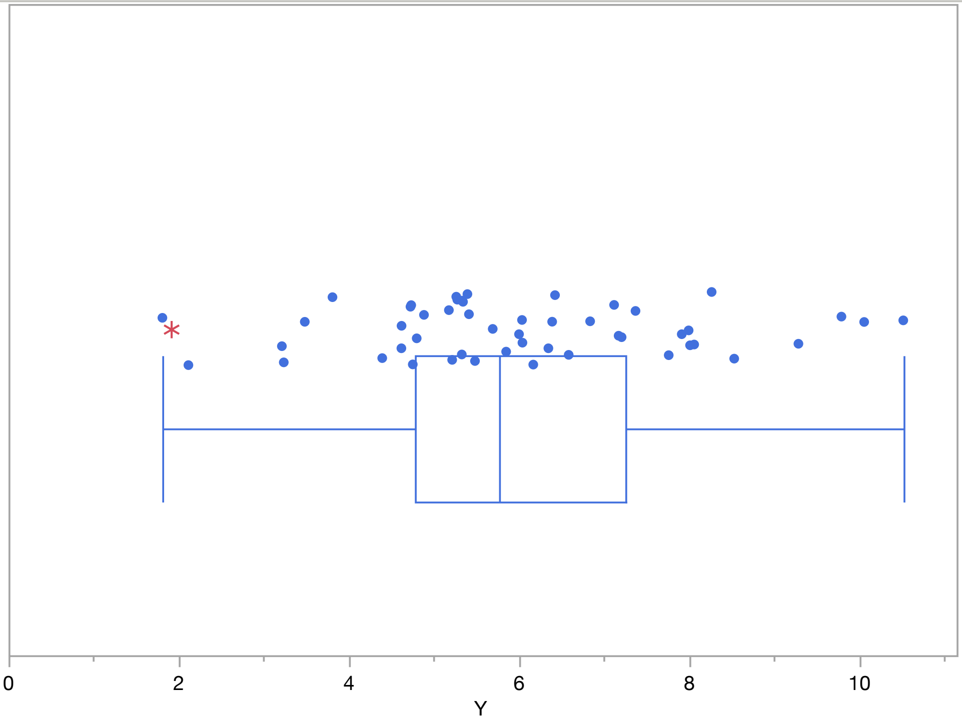

Well = C12

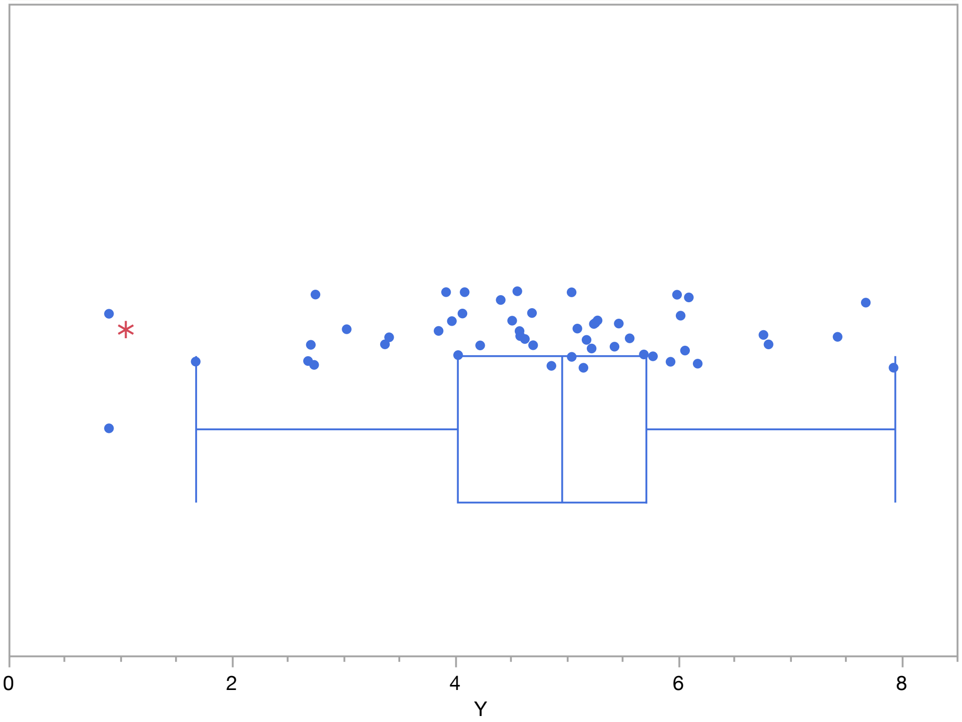

Well = D01

Graph Builder

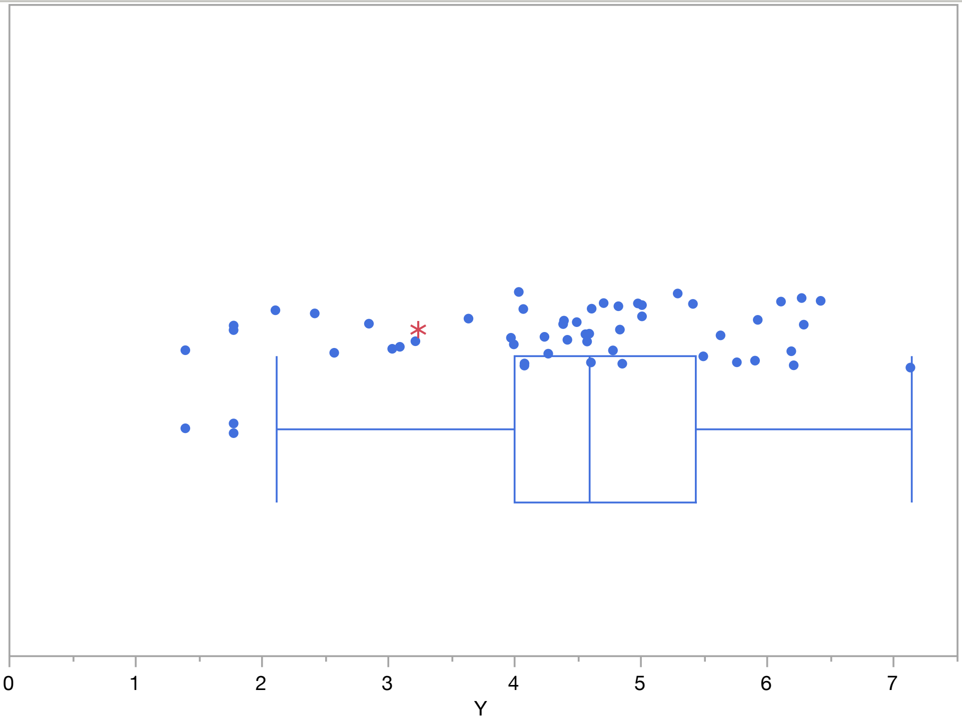

Well = D02

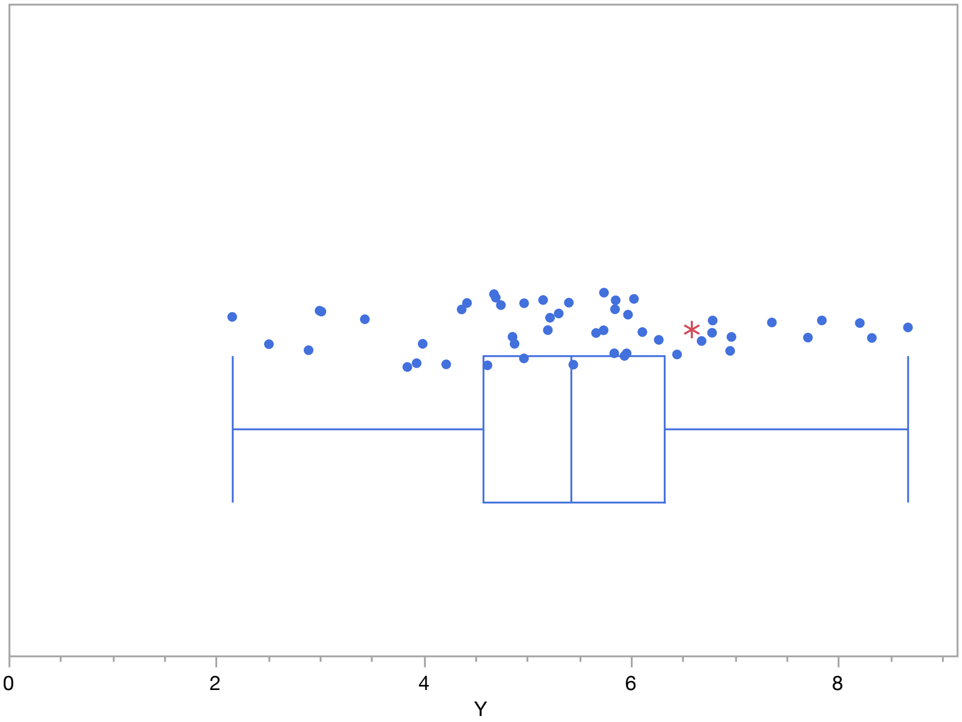

Well = D03

Graph Builder

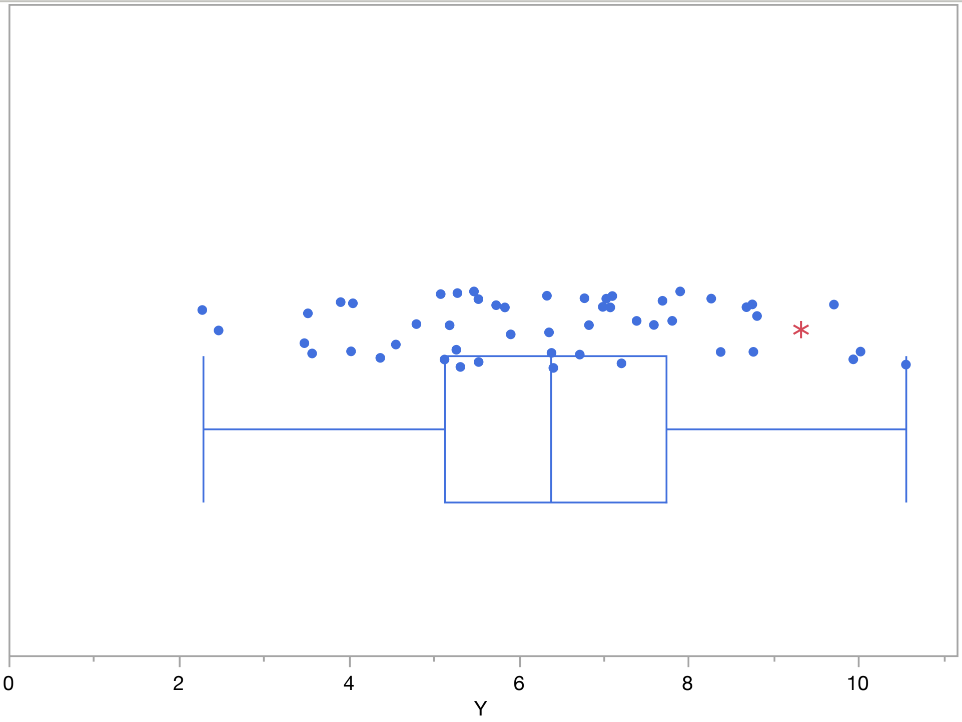

Well = D04

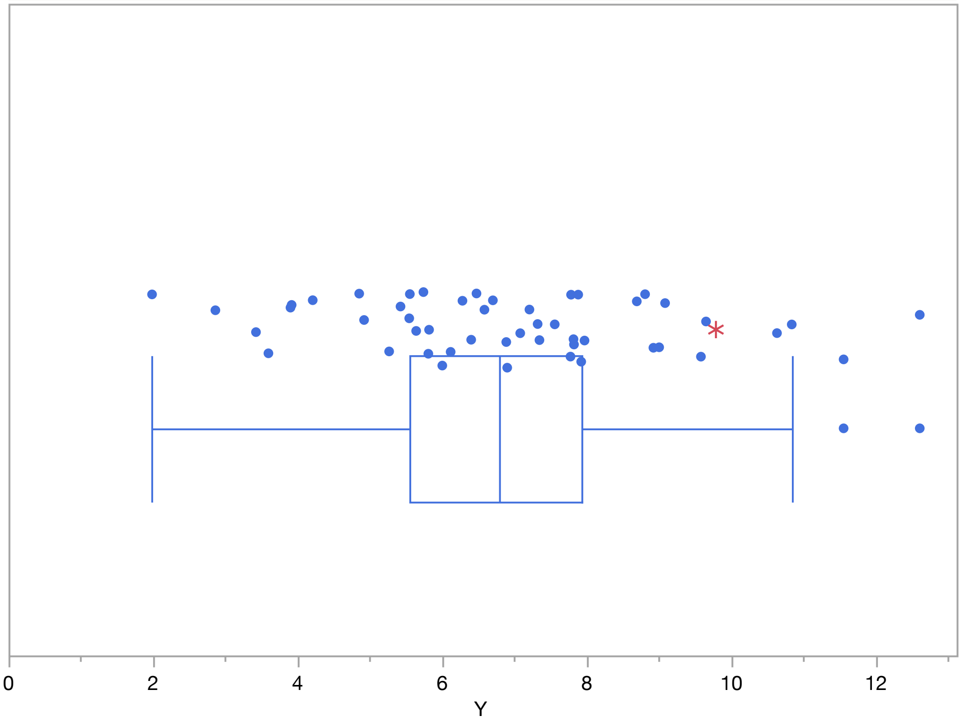

Well = D05

Graph Builder

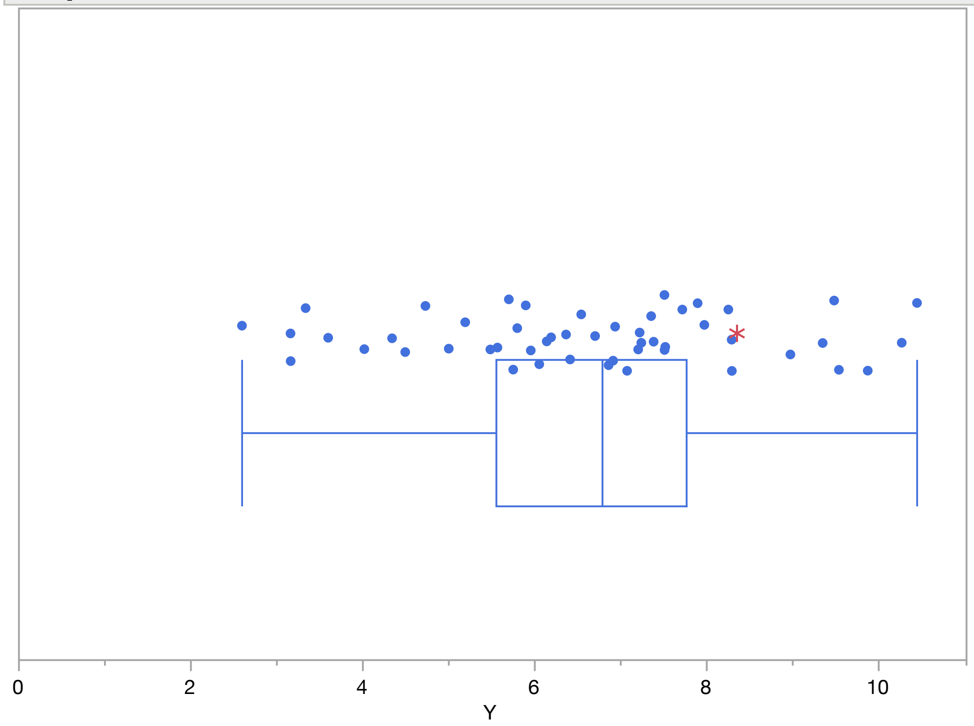

Well = D06

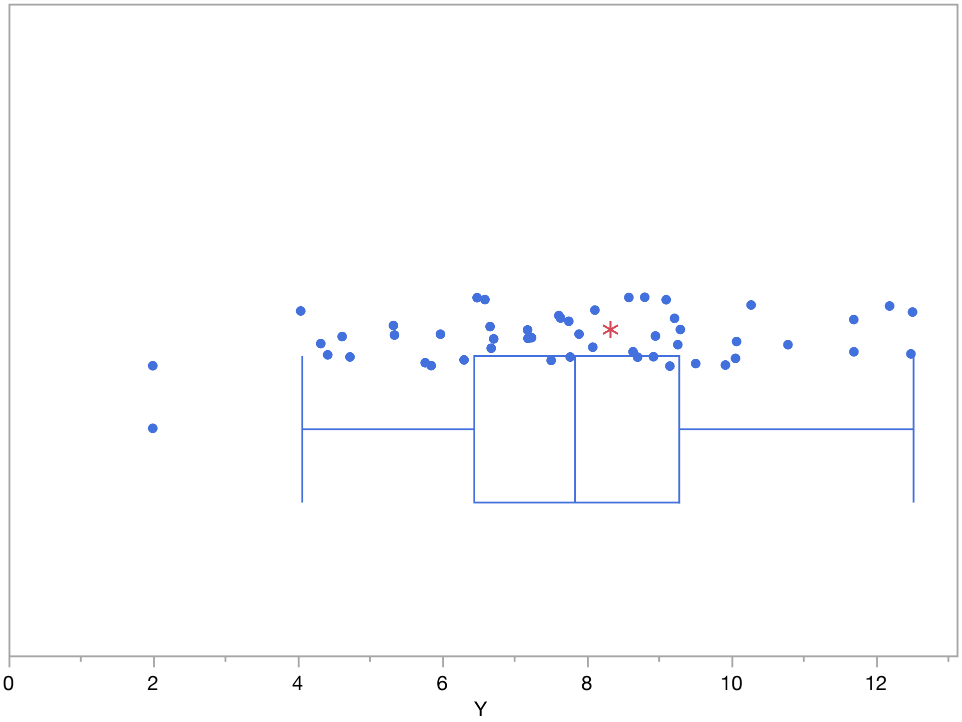

Well = D07

Graph Builder

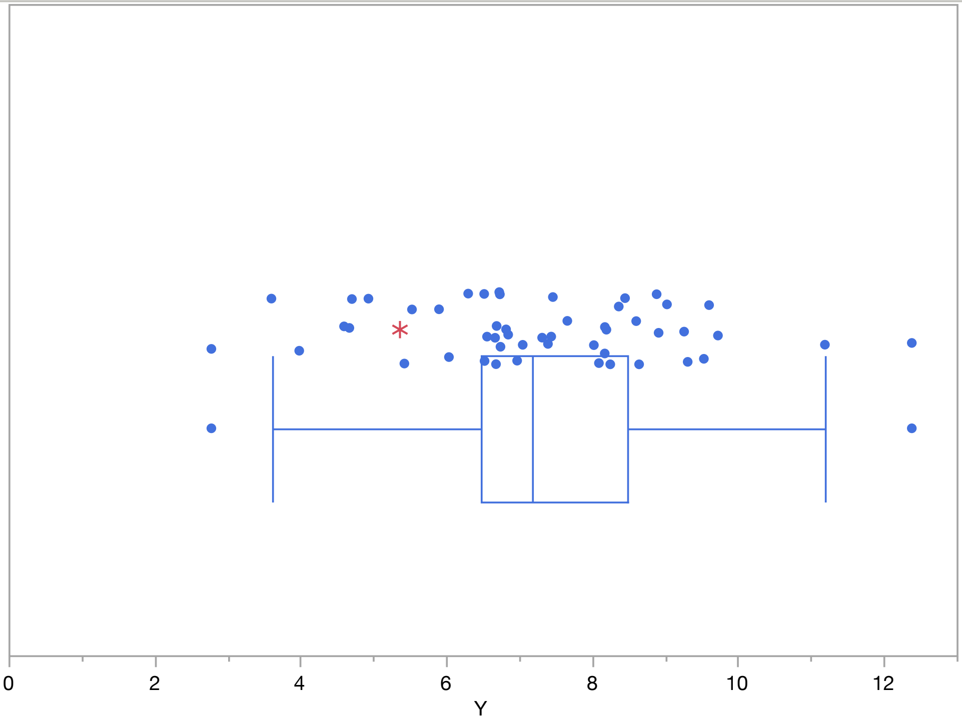

Well = D08

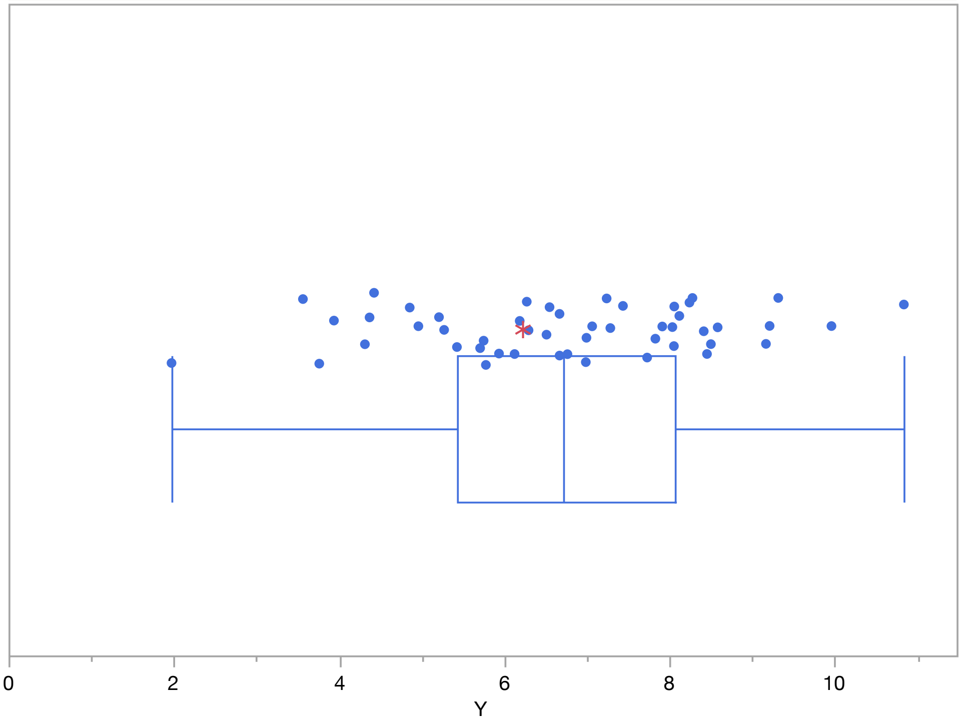

Well = D09

Graph Builder

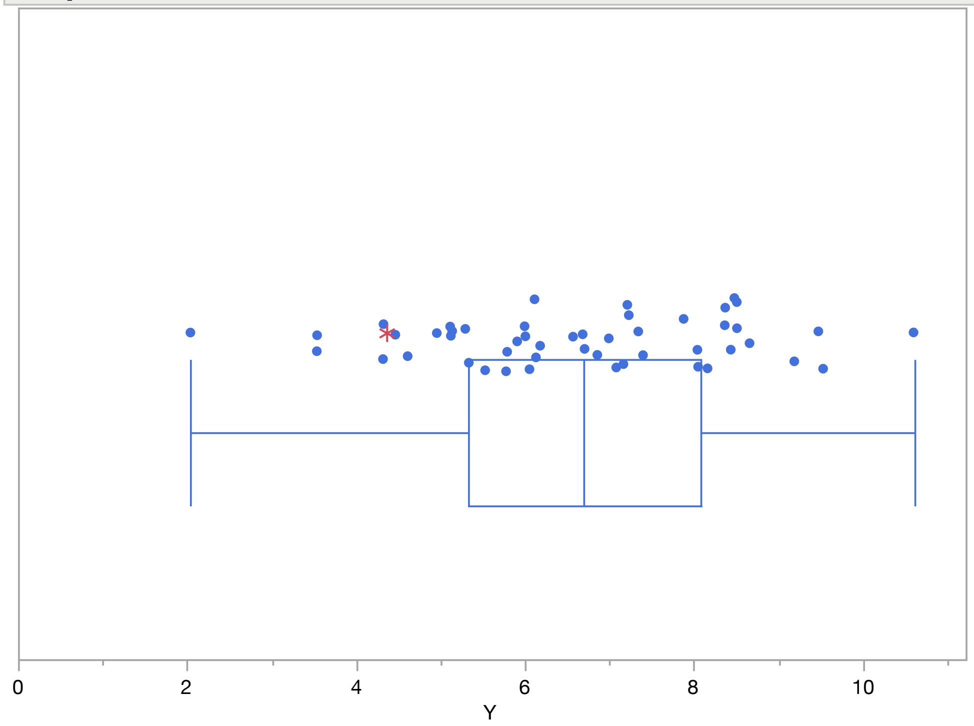

Well = D10

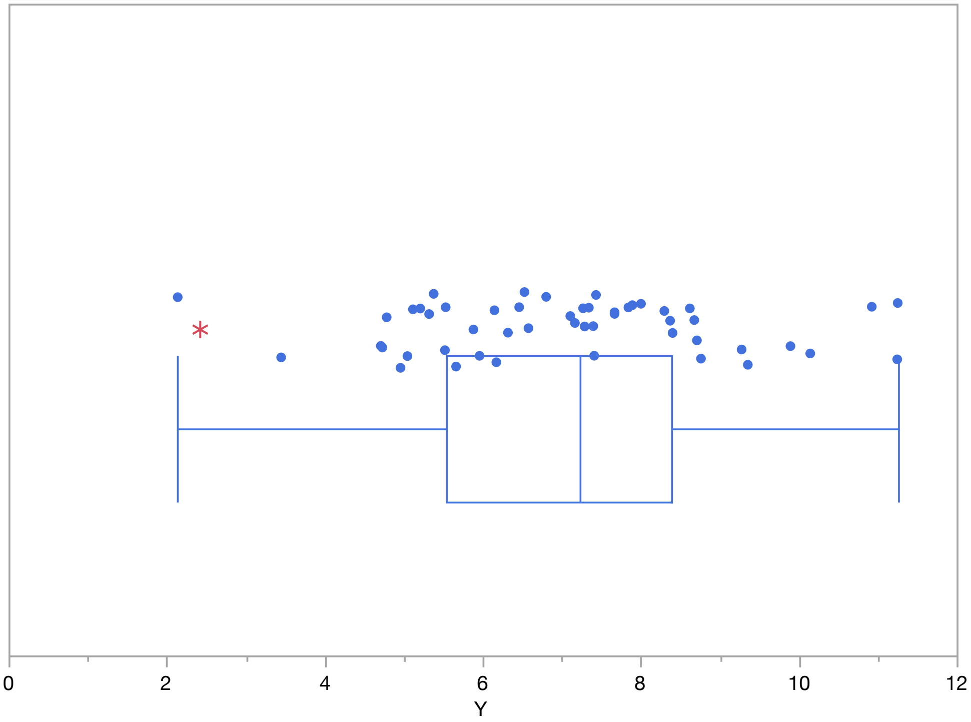

Well = D11

Graph Builder

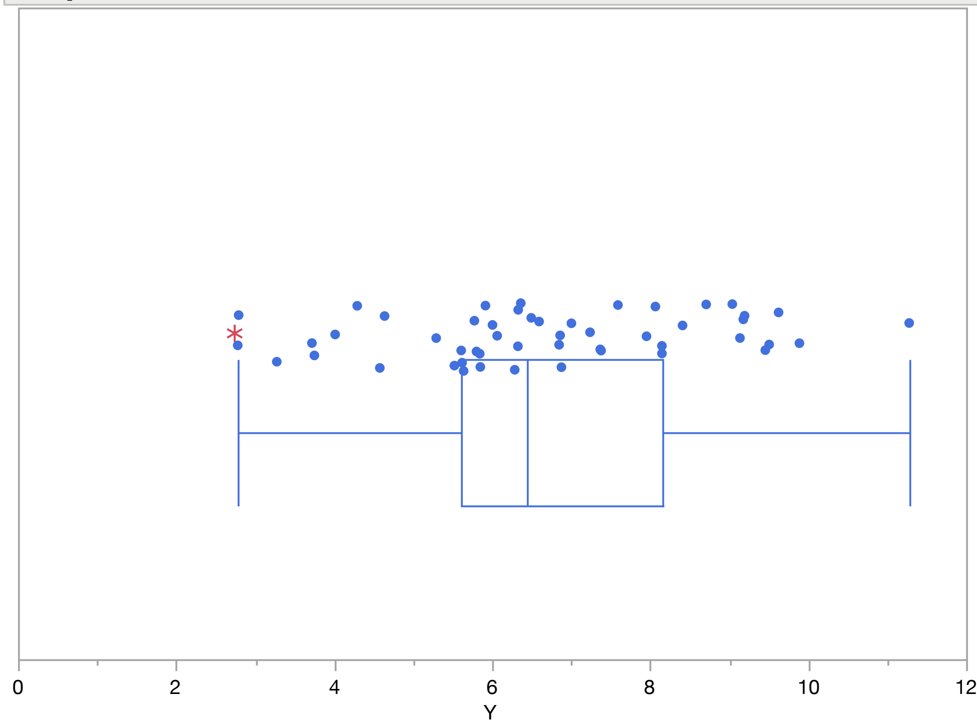

Well = D12

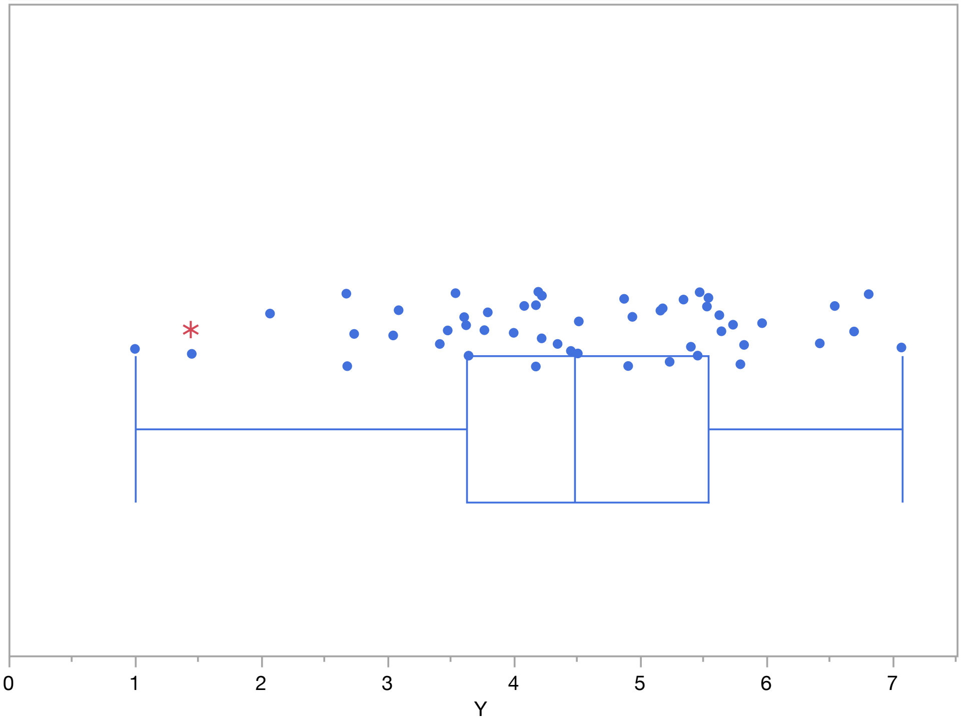

Well = E01

Graph Builder

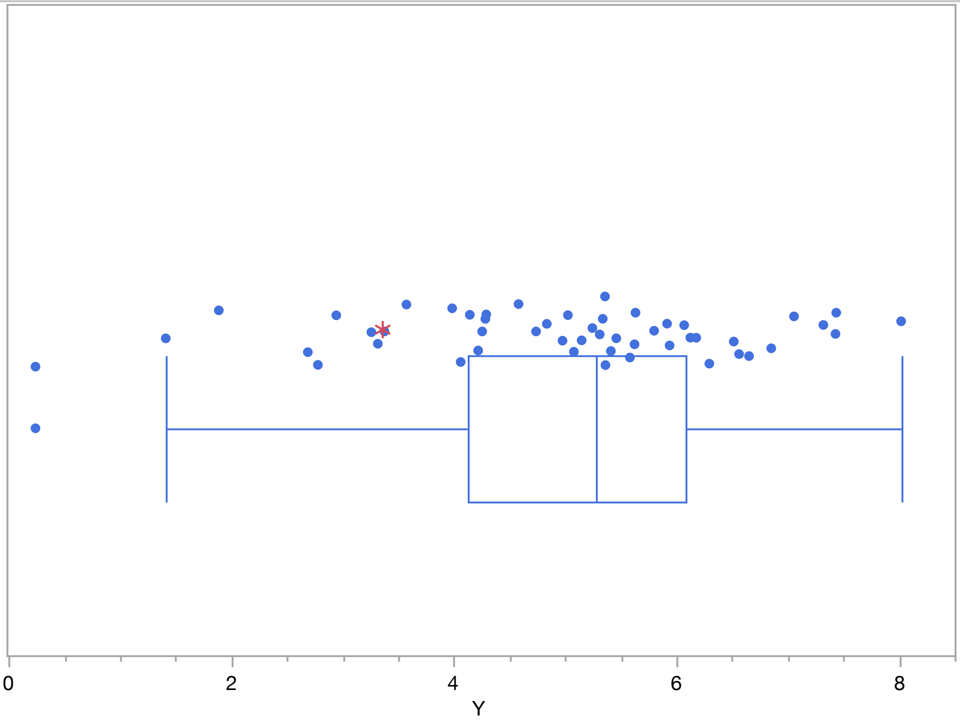

Well = E02

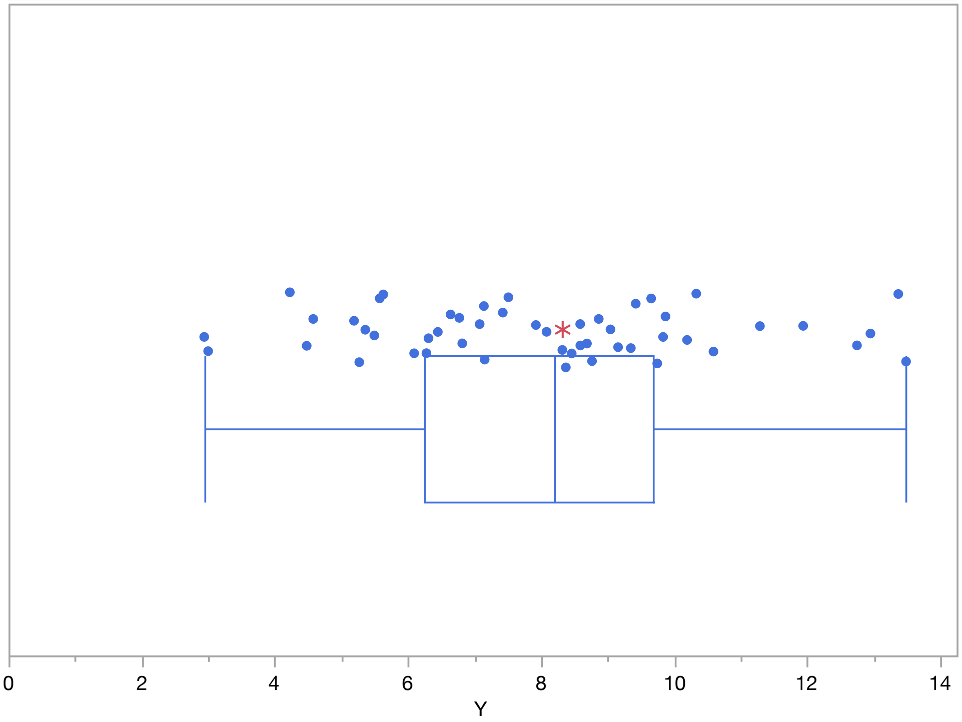

Well = E03

Graph Builder

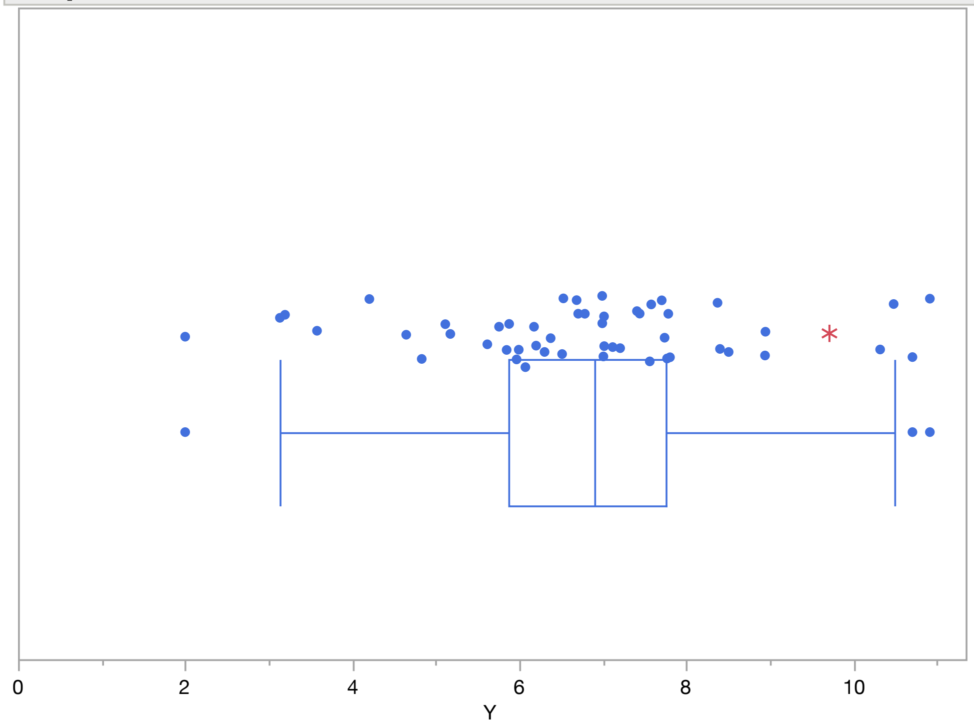

Well = E04

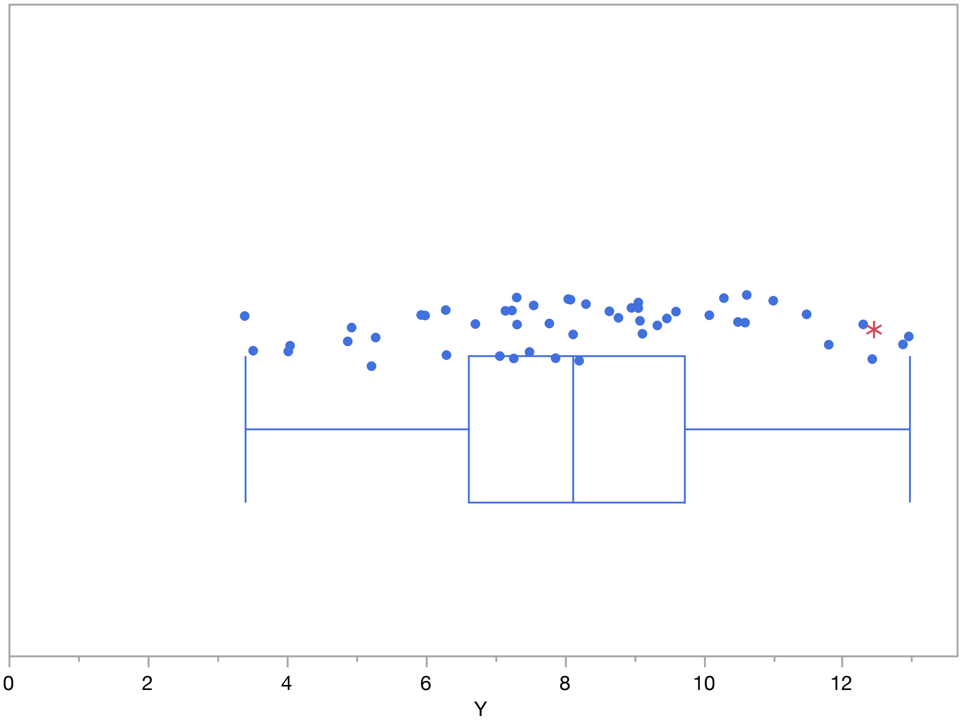

Well = E05

Graph Builder

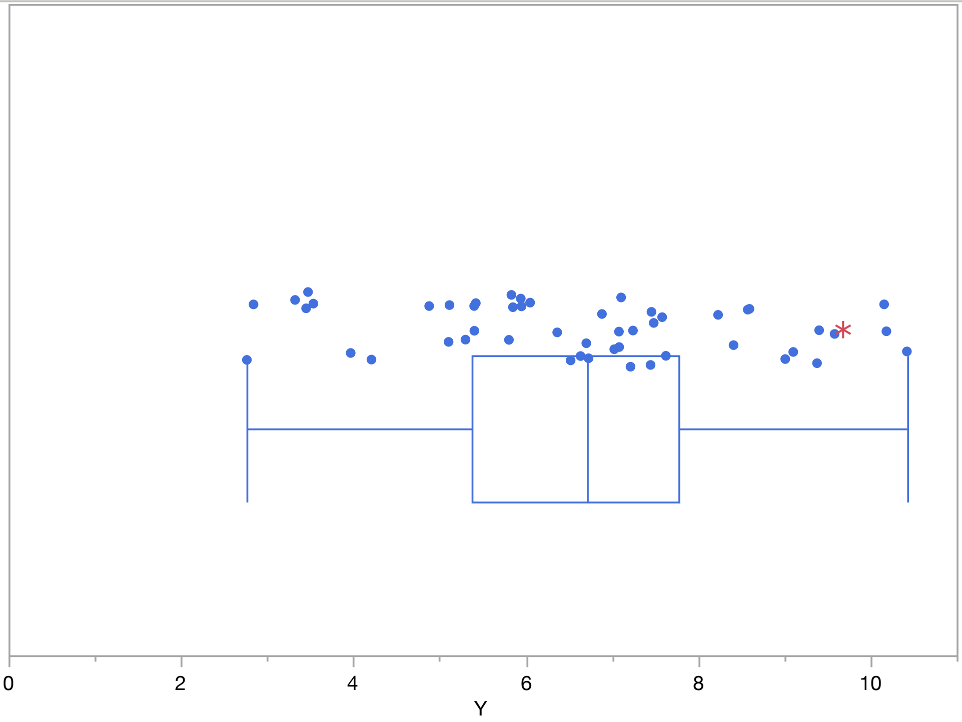

Well = E06

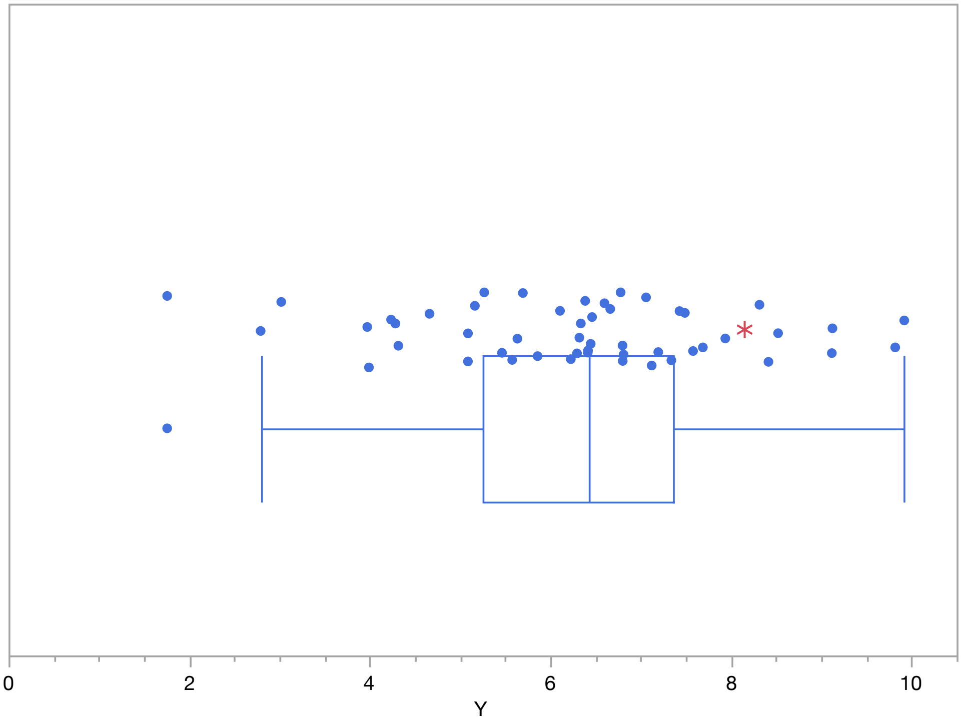

Well = E07

Graph Builder

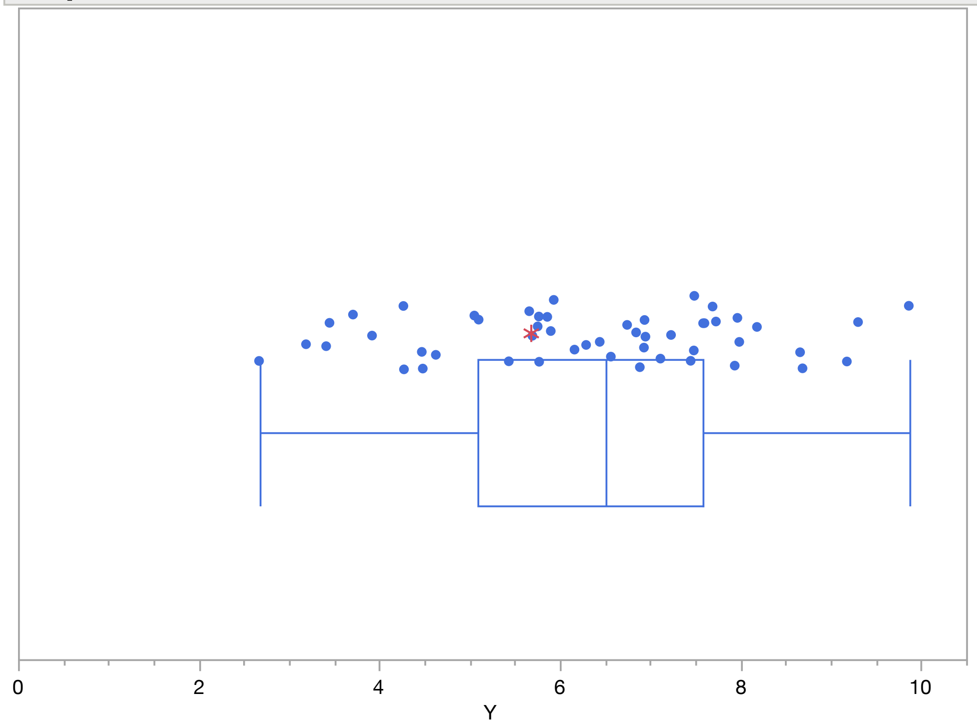

Well = E08

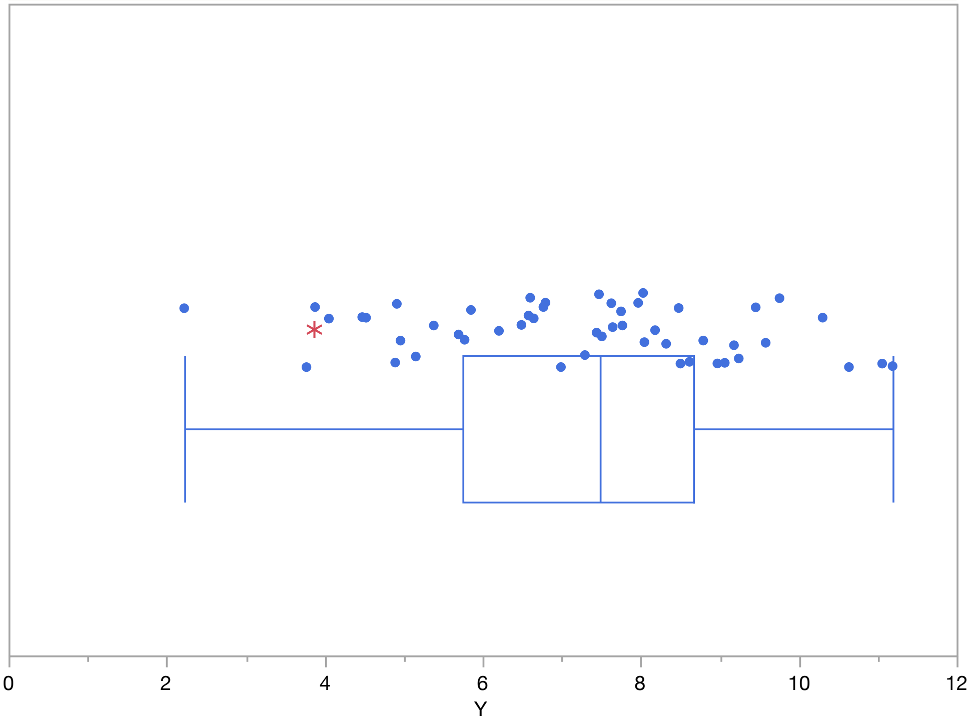

Well = E09

Graph Builder

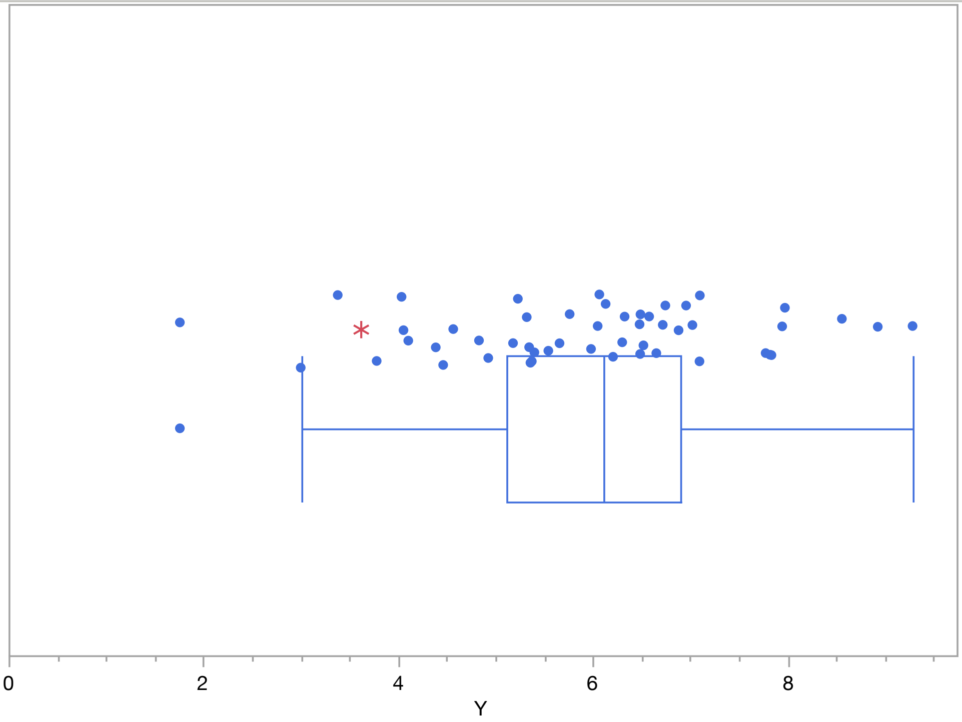

Well = E10

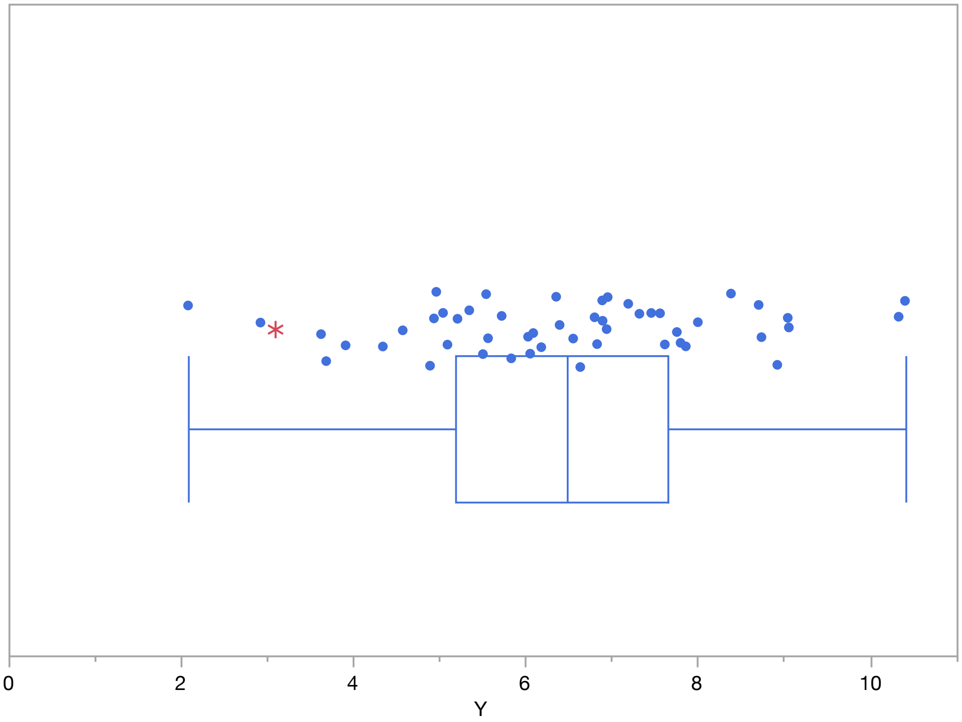

Well = E11

Graph Builder

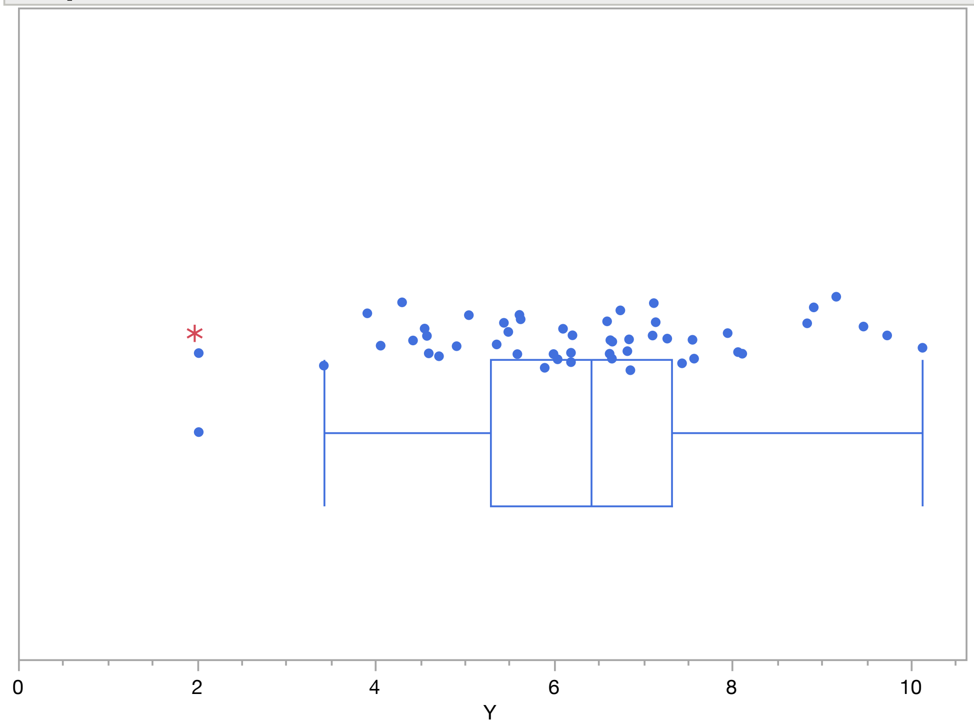

Well = E12

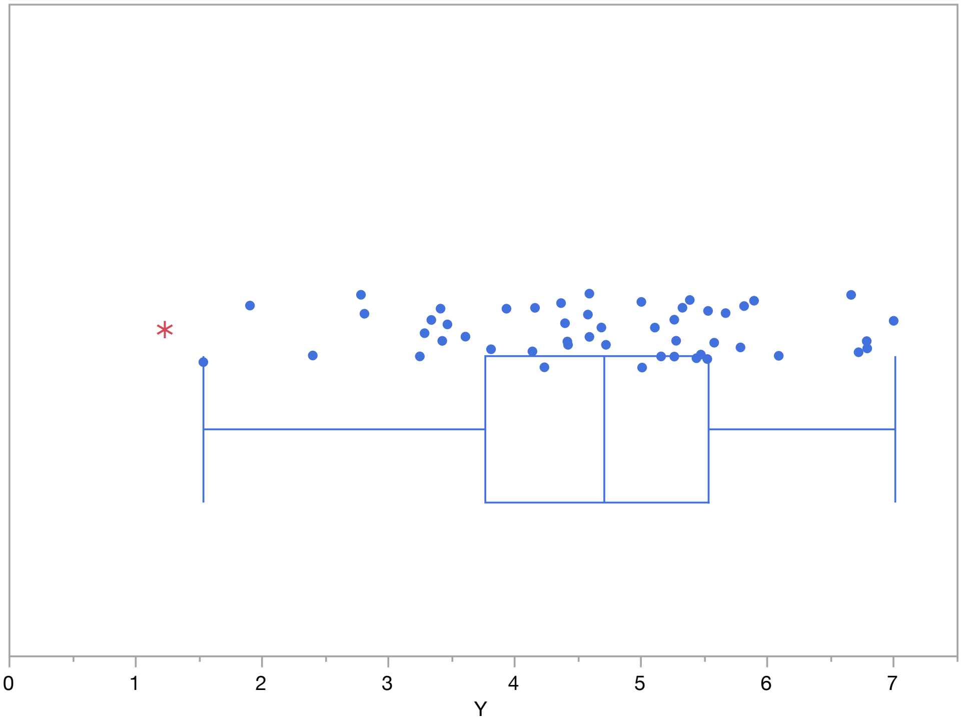

Well = F01

Graph Builder

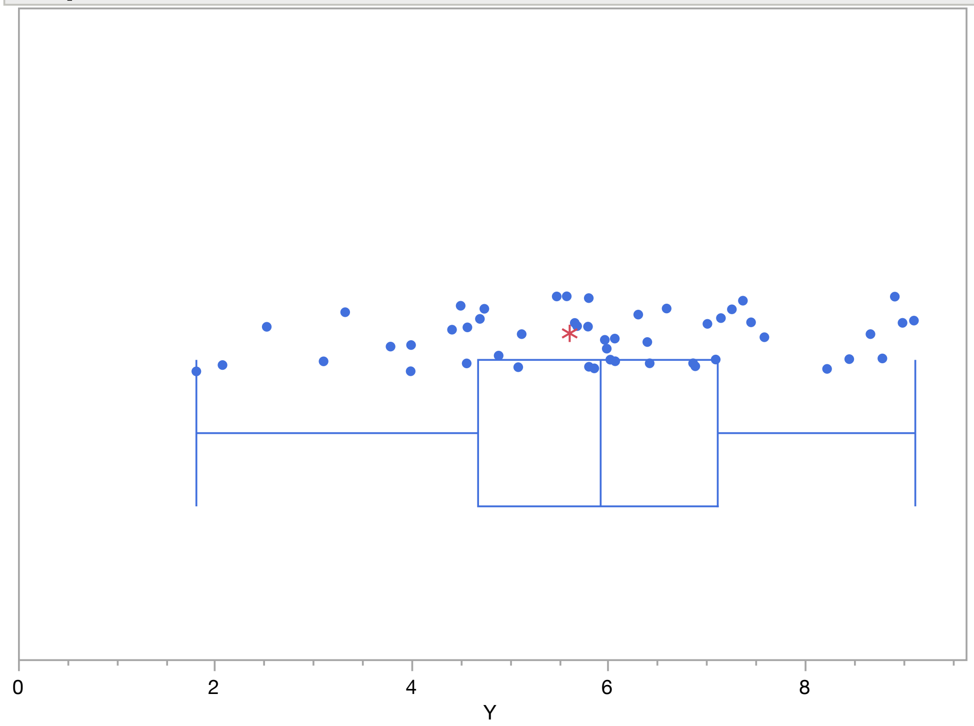

Well = F02

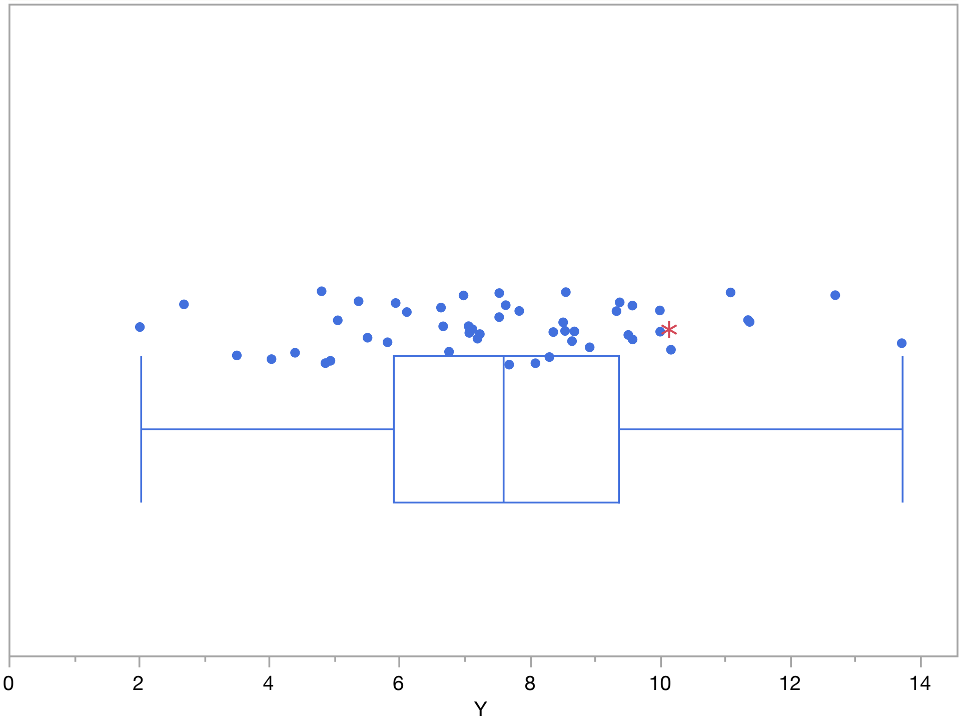

Well = F03

Graph Builder

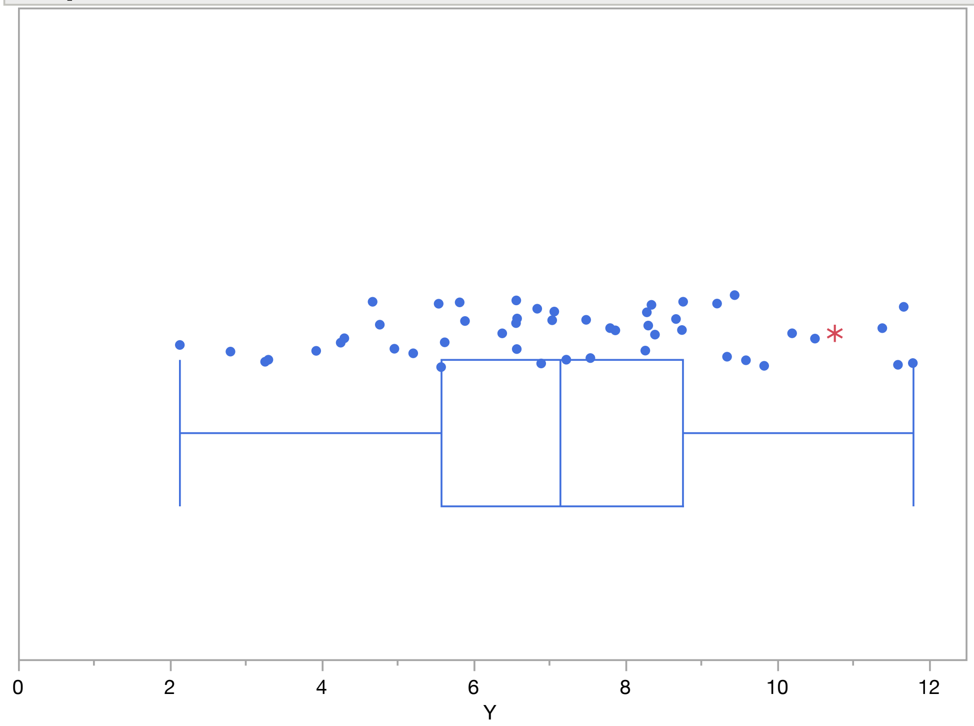

Well = F04

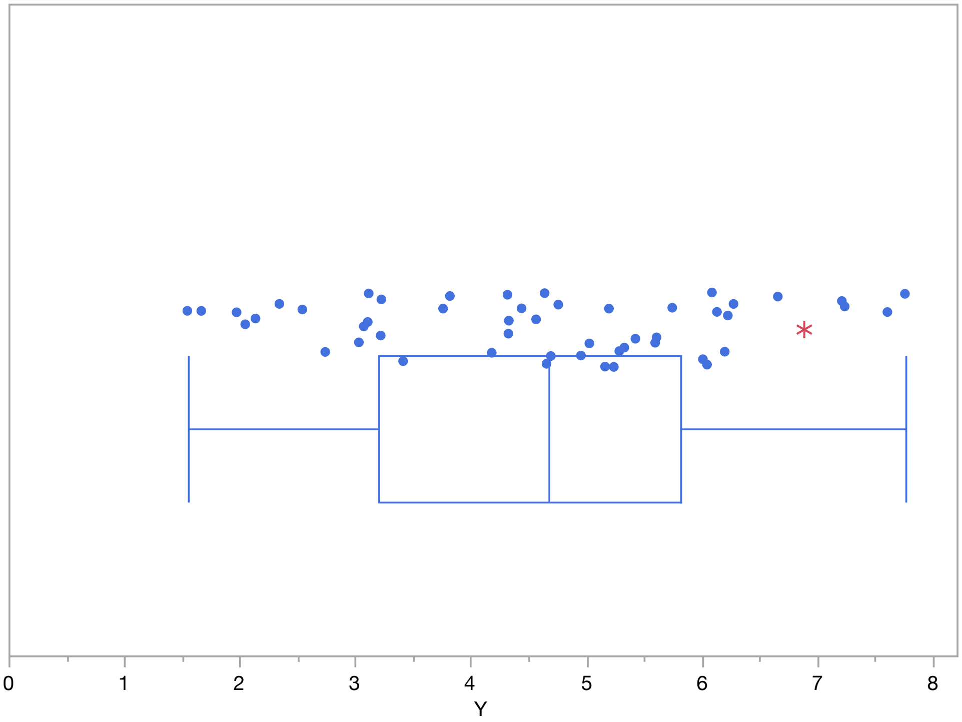

Well = F05

Graph Builder

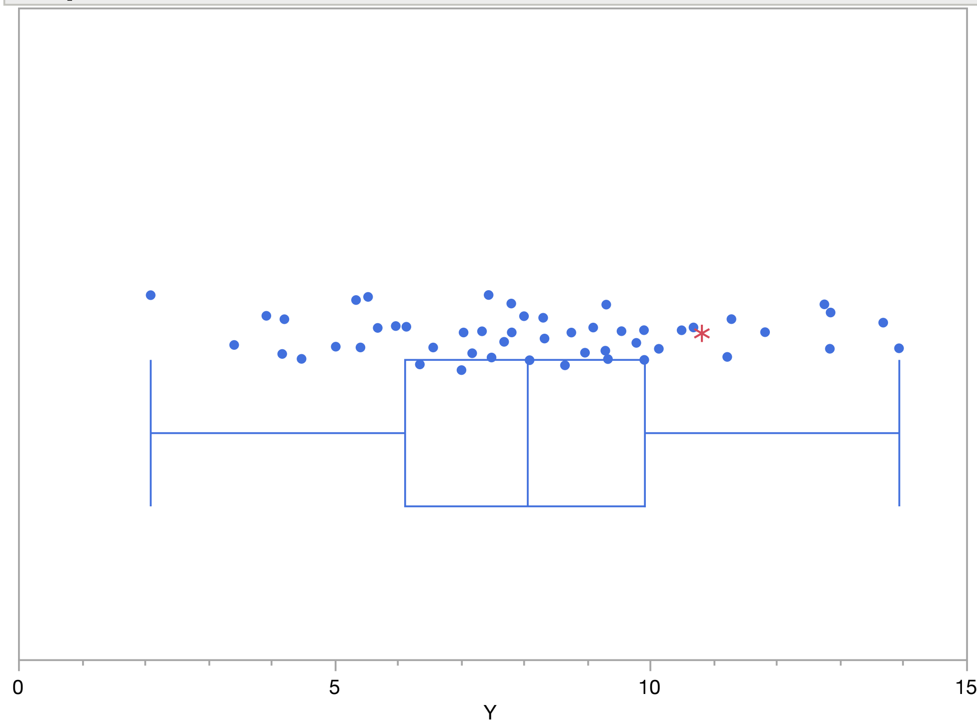

Well = F06

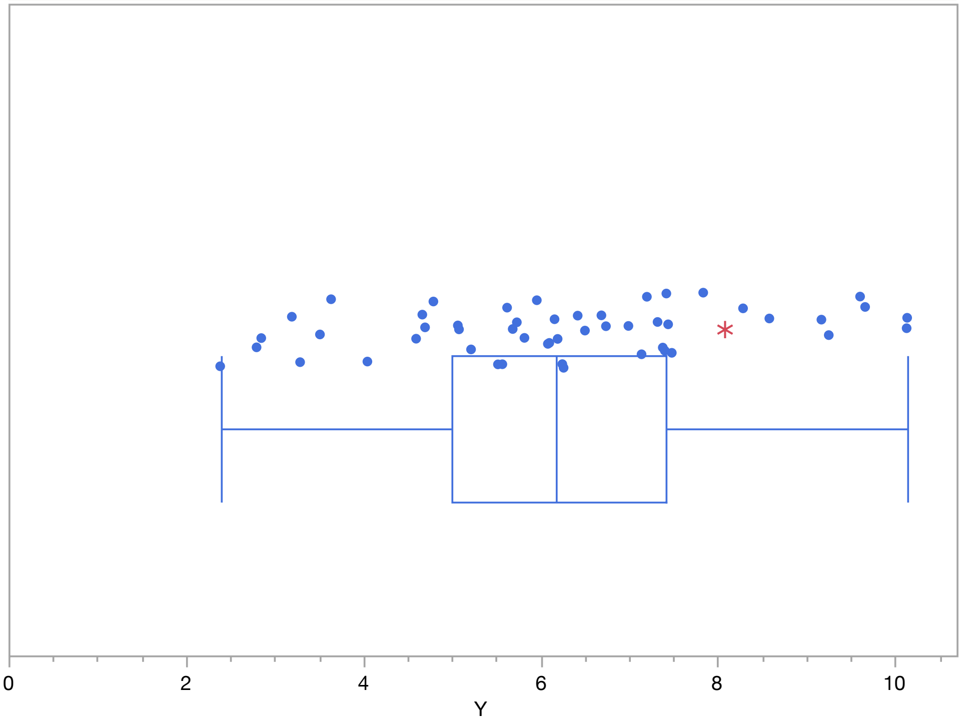

Well = F07

Graph Builder

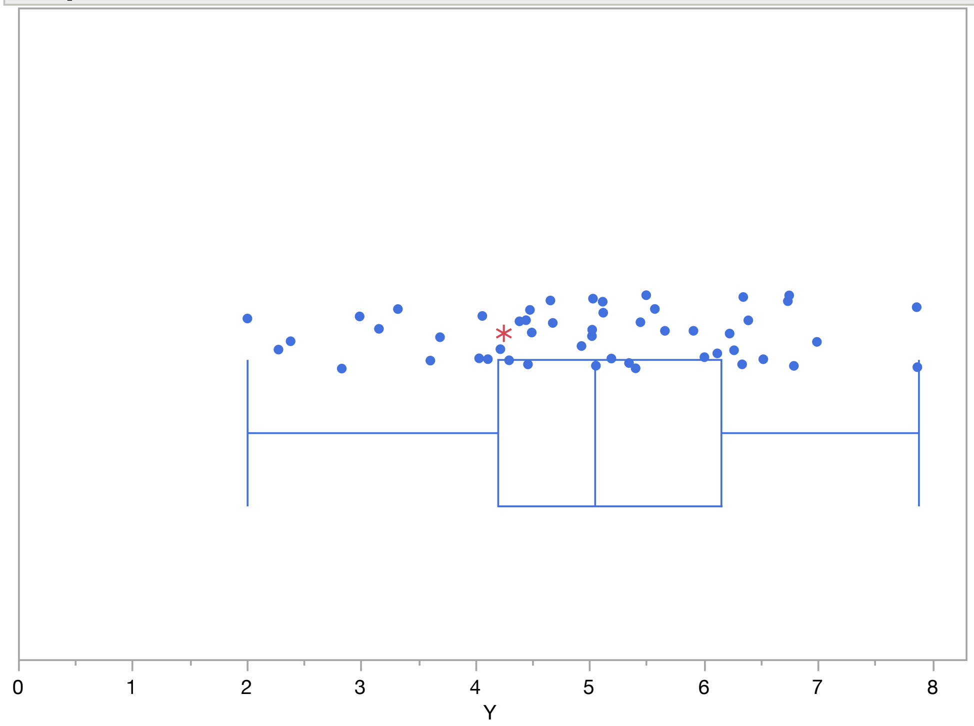

Well = F08

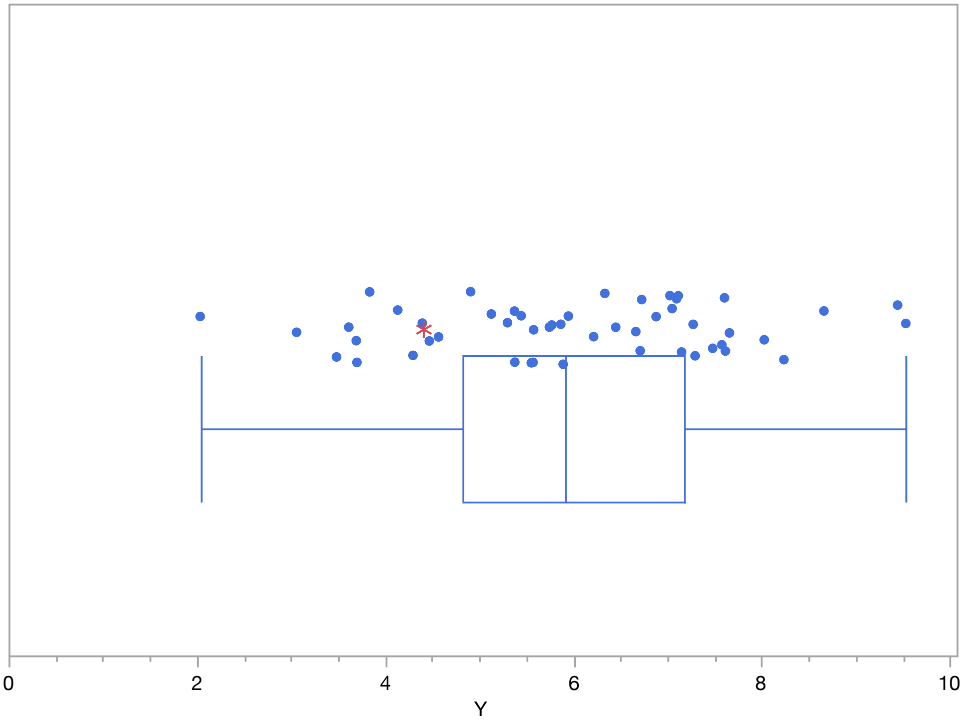

Well = F09

Graph Builder

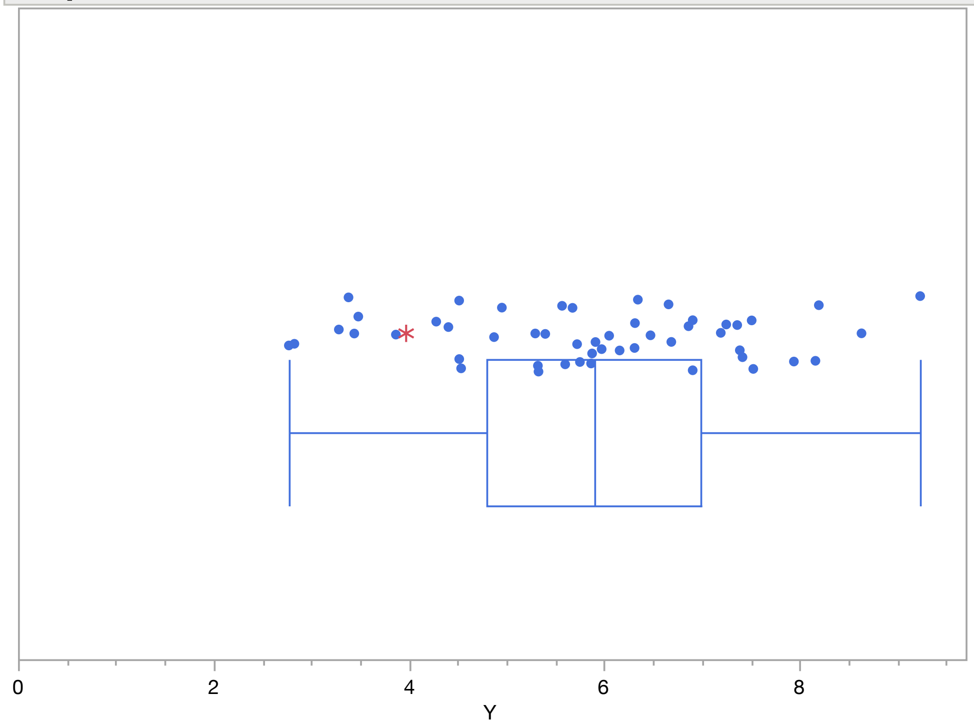

Well = F10

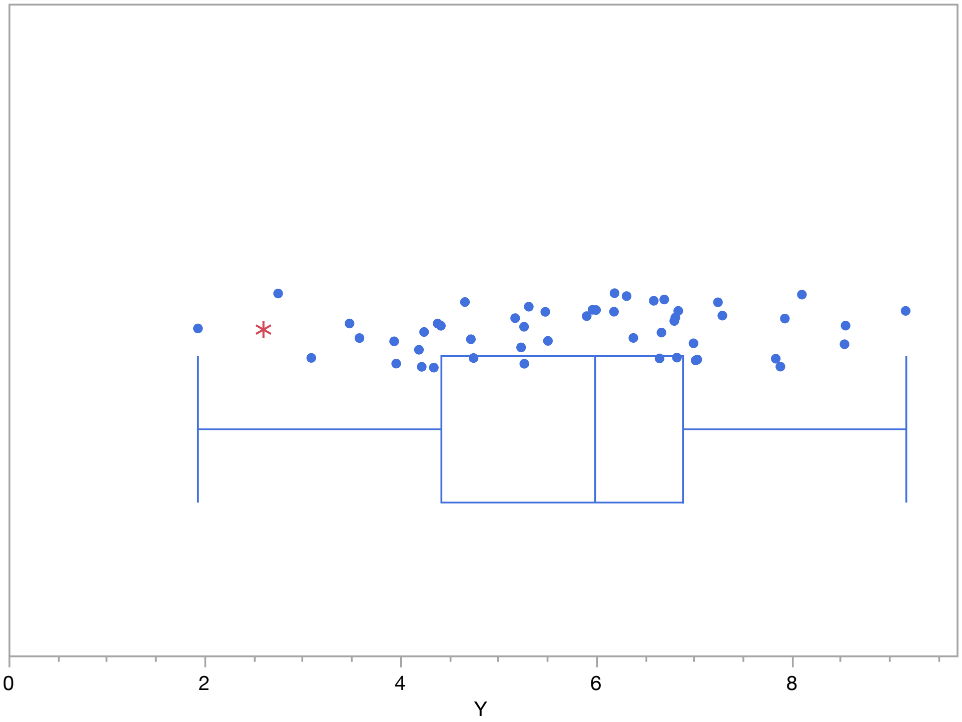

Well = F11

Graph Builder

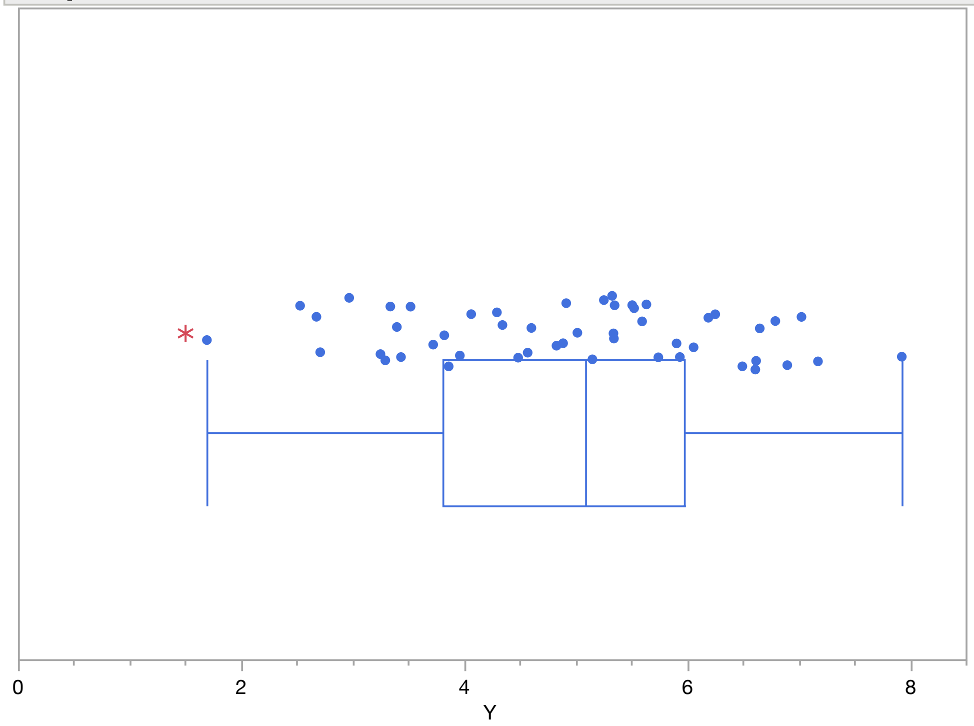

Well = F12

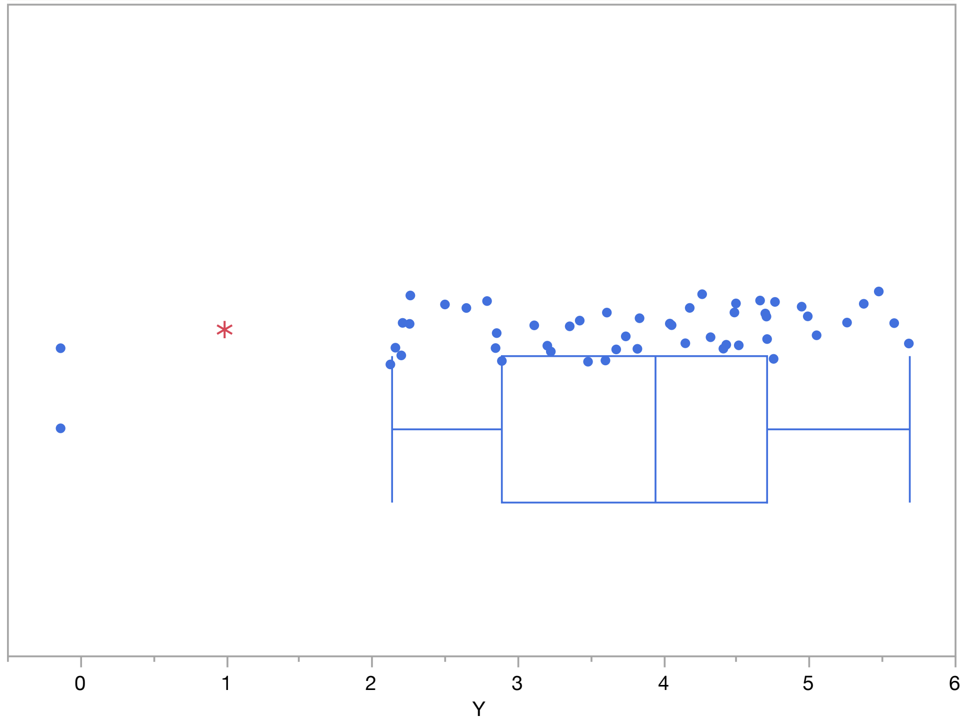

Well = G01

Graph Builder

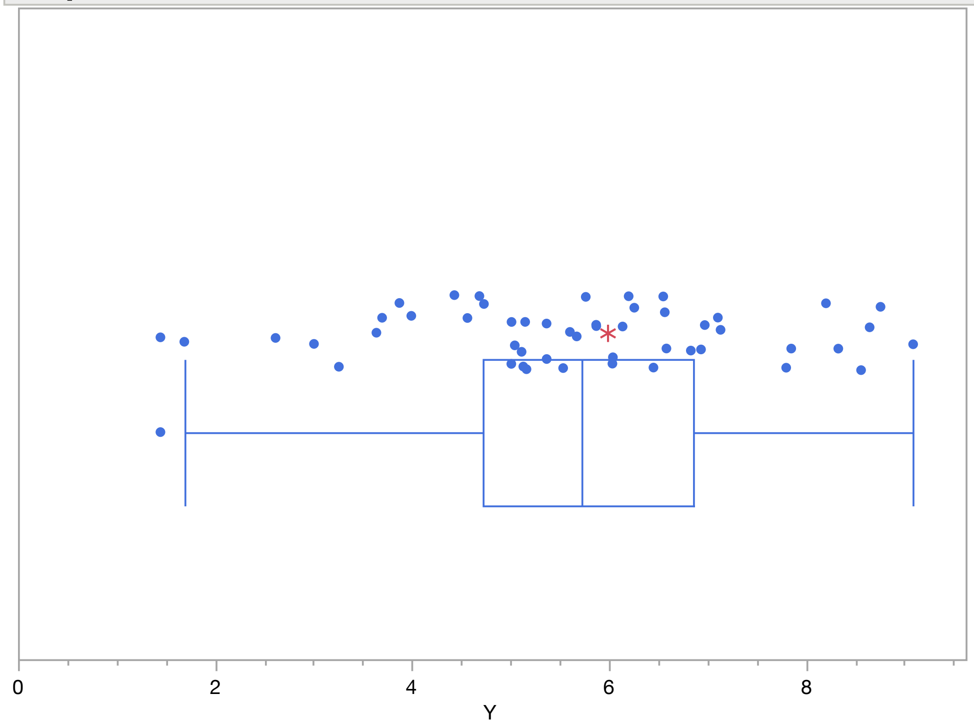

Well = G02

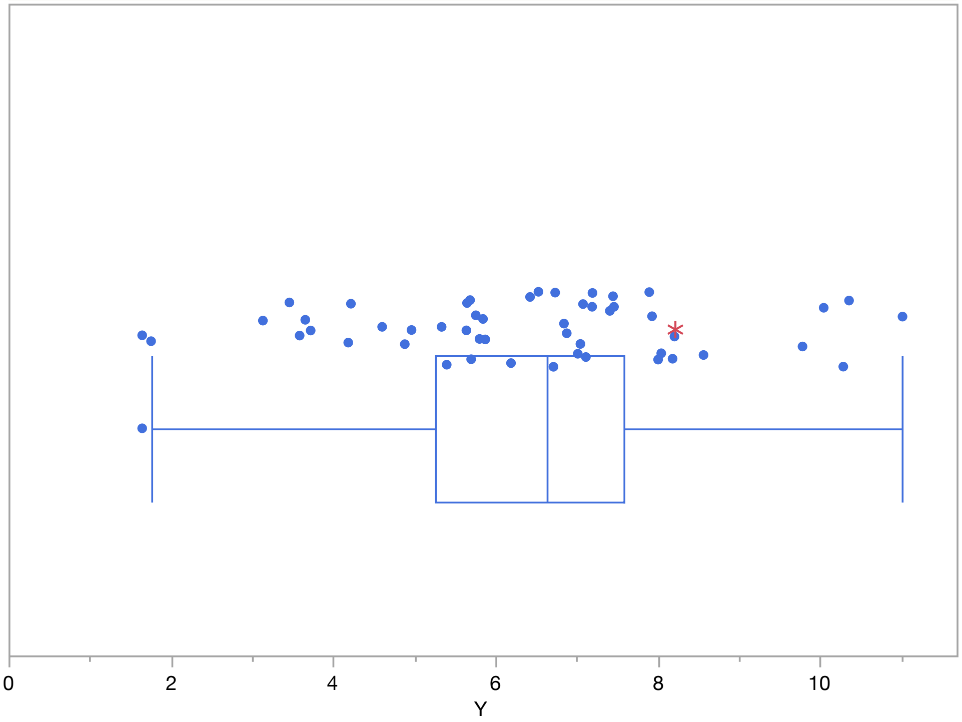

Well = G03

Graph Builder

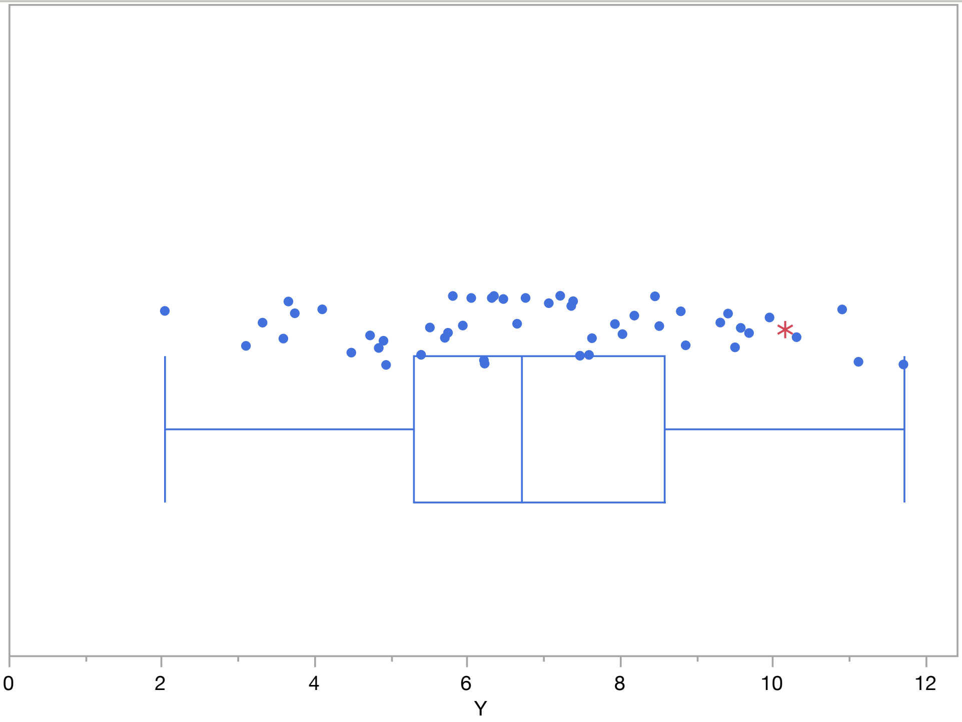

Well = G04

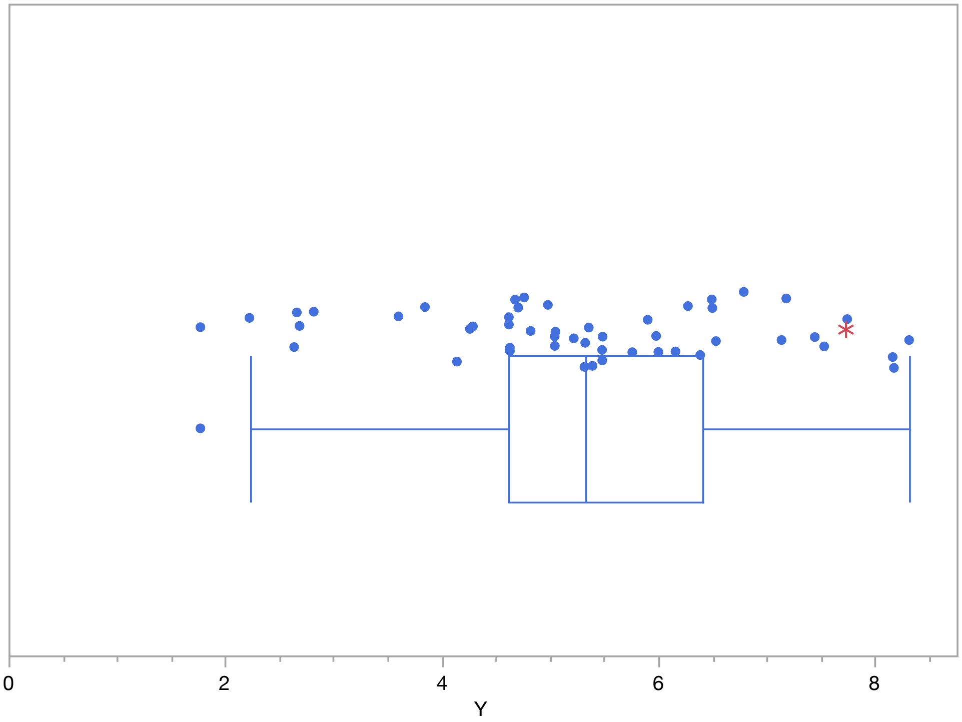

Well = G05

Graph Builder

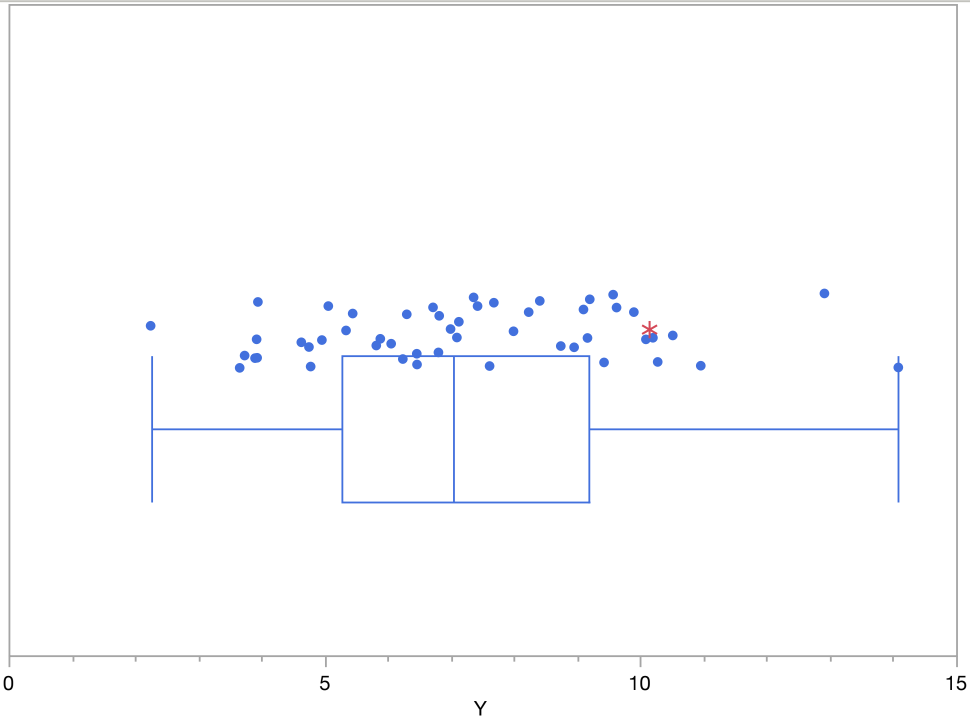

Well = G06

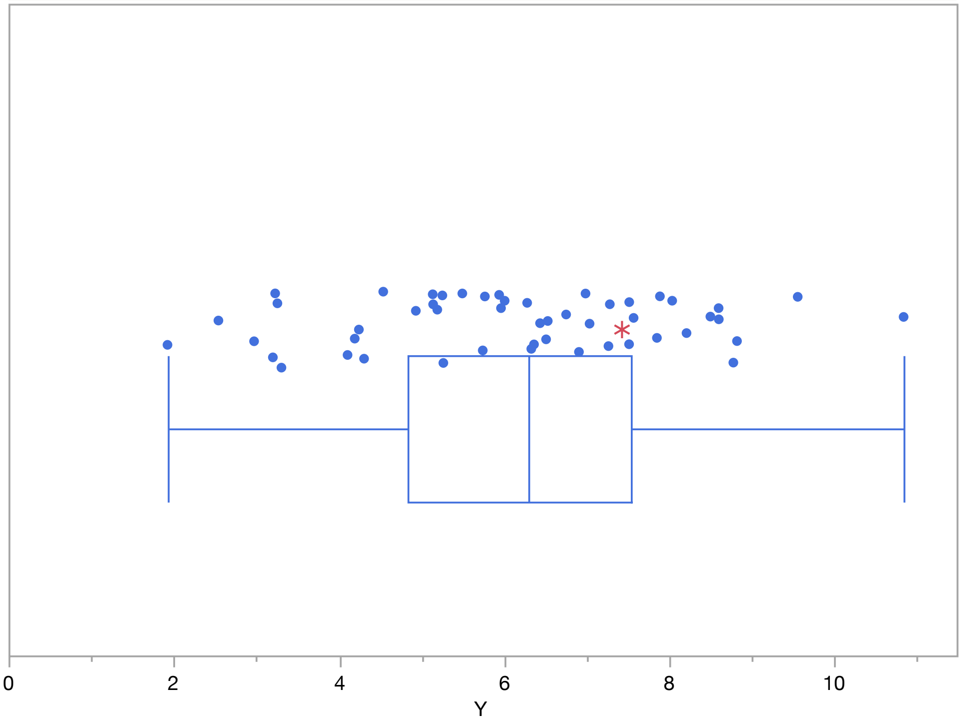

Well = G07

Graph Builder

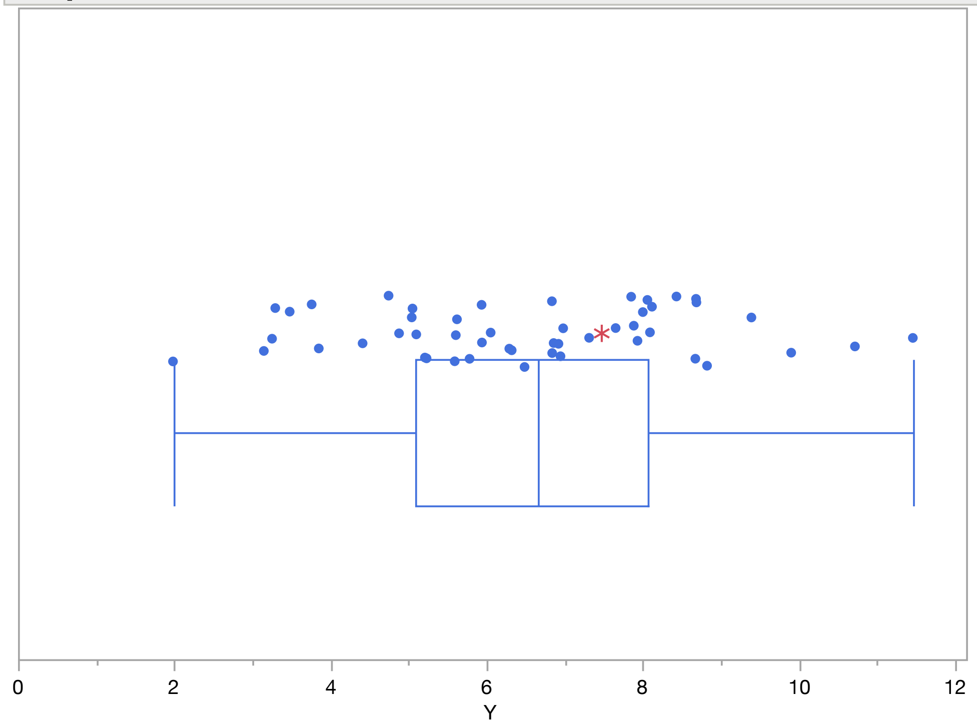

Well = G08

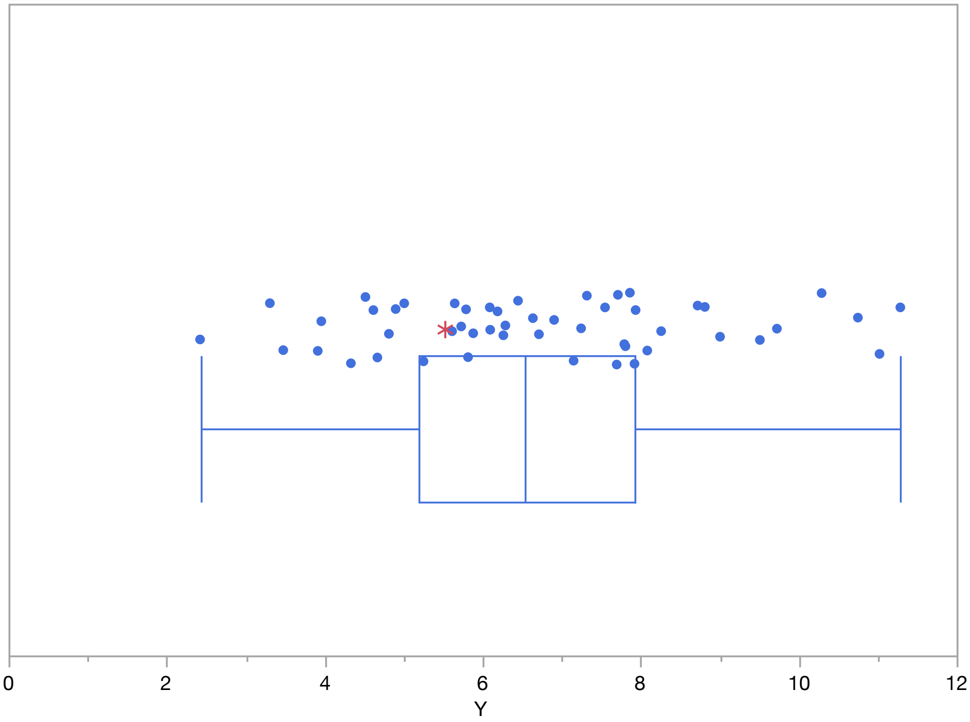

Well = G09

Graph Builder

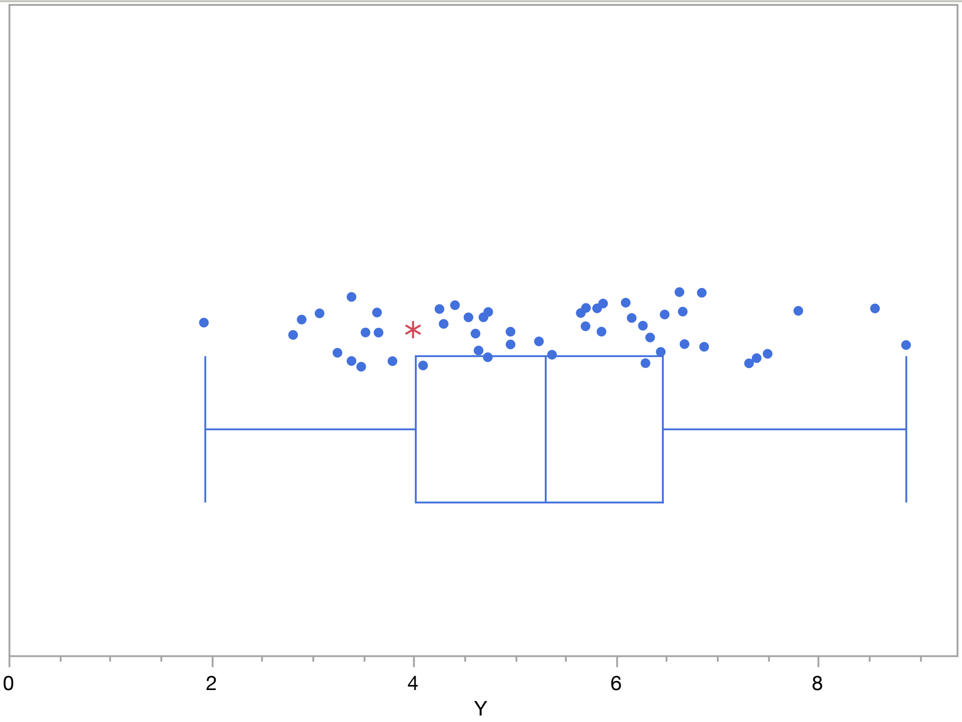

Well = G10

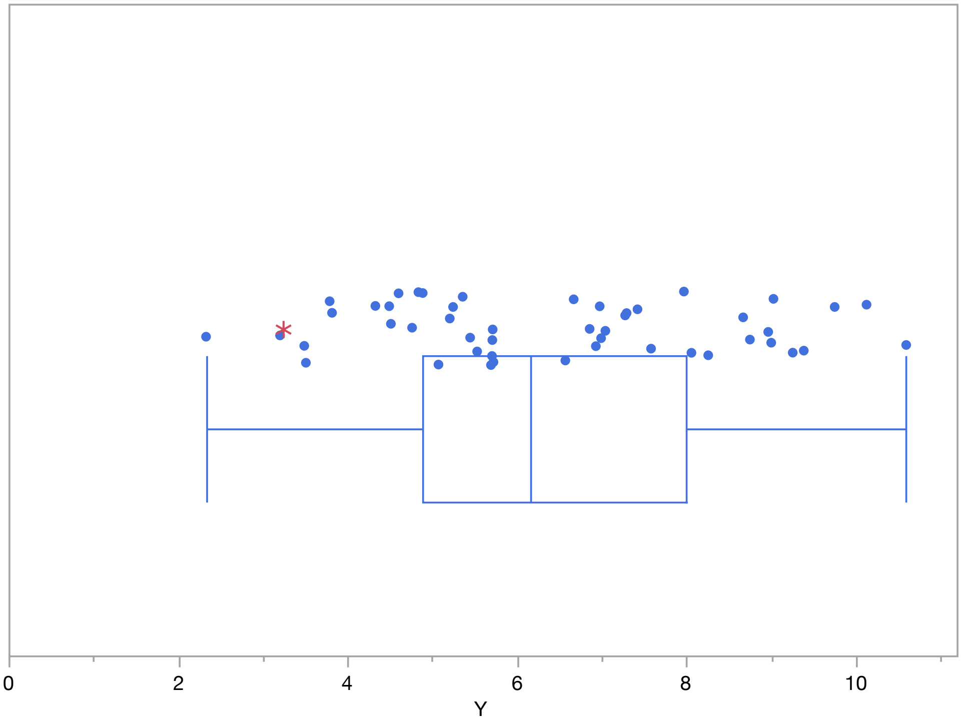

Well = G11

Graph Builder

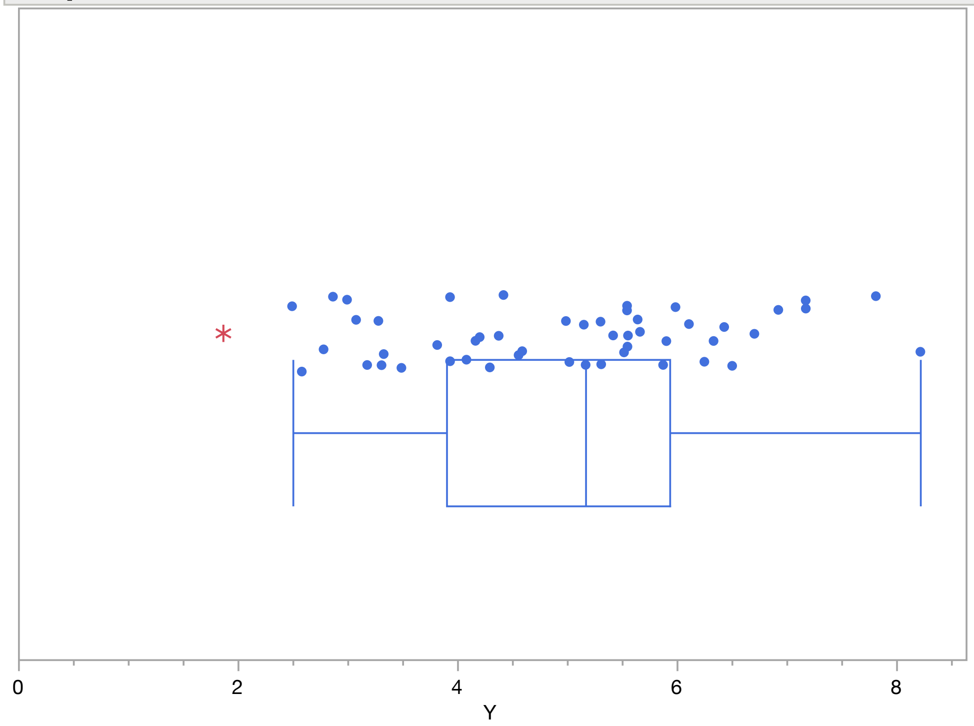

Well = G12

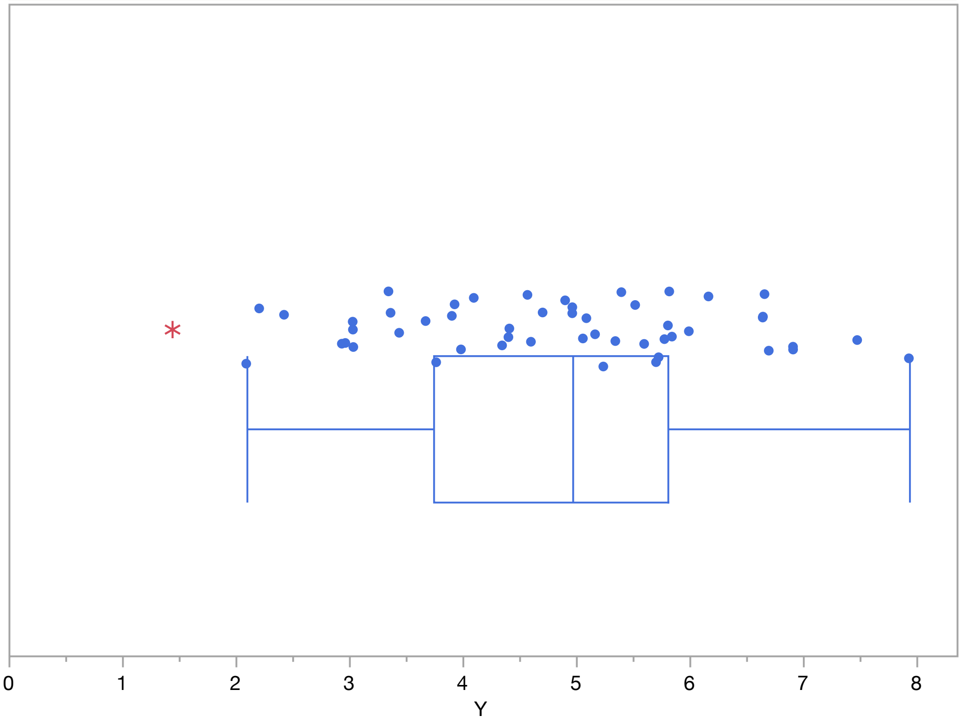

Well = H01

Graph Builder

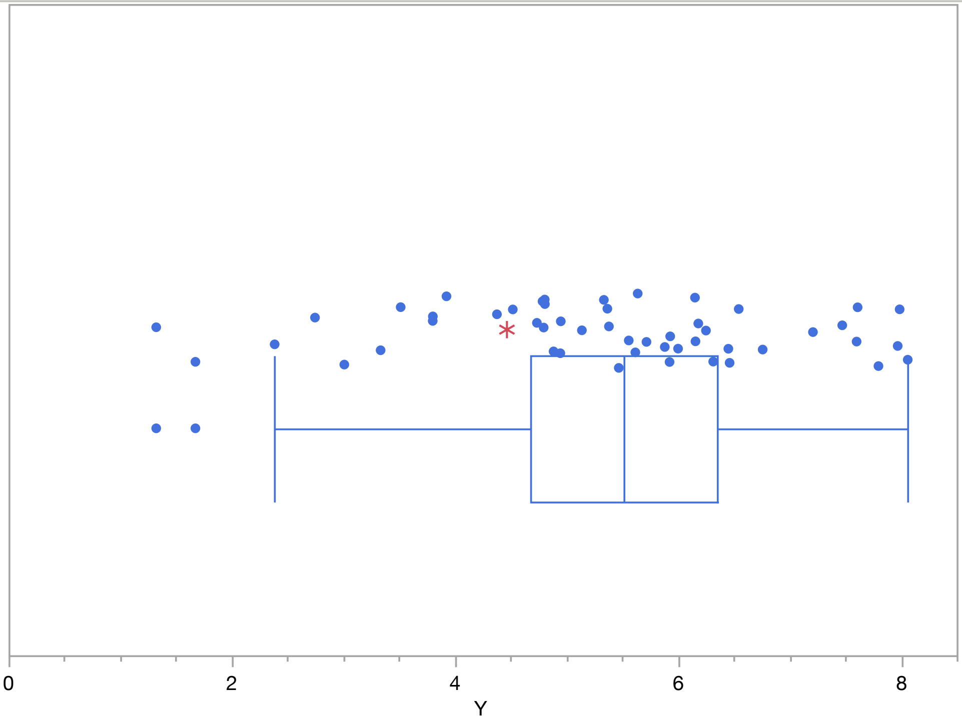

Well = H02

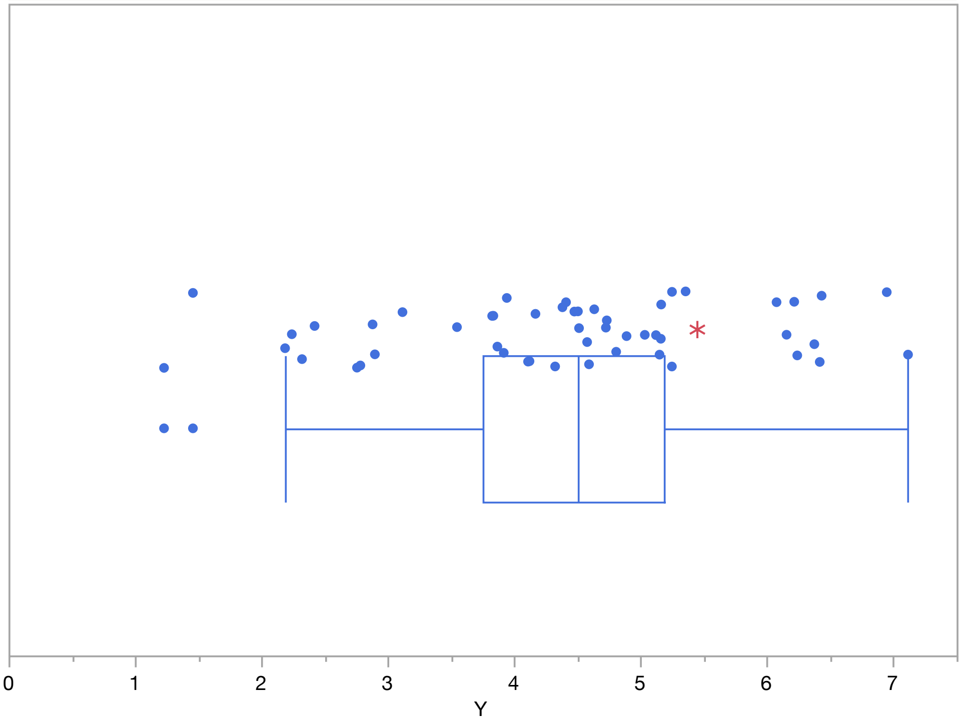

Well = H03

Graph Builder

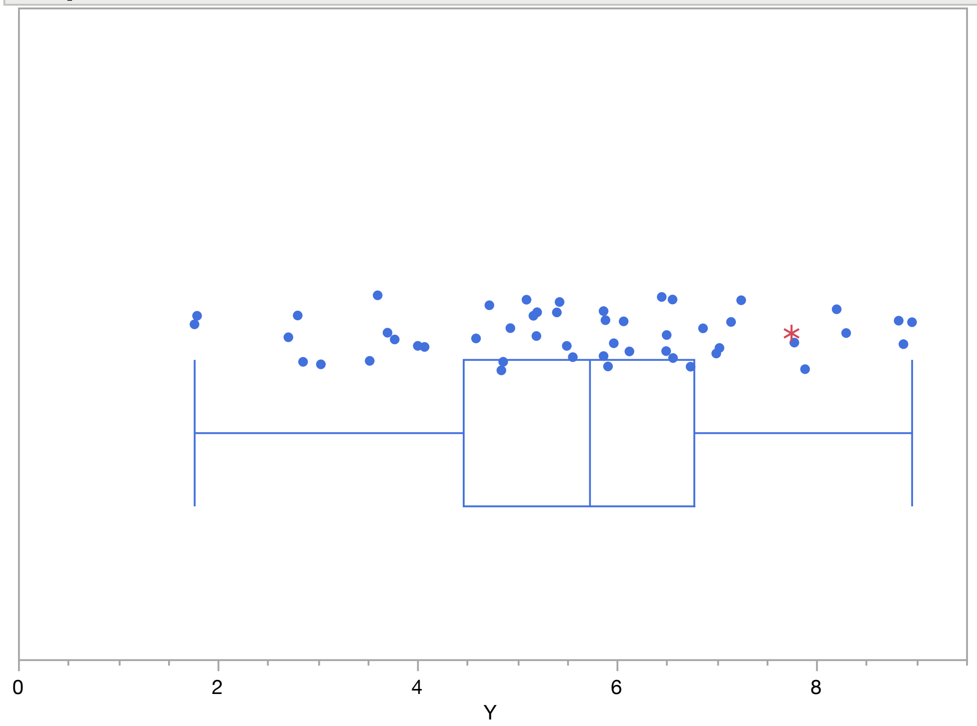

Well = H04

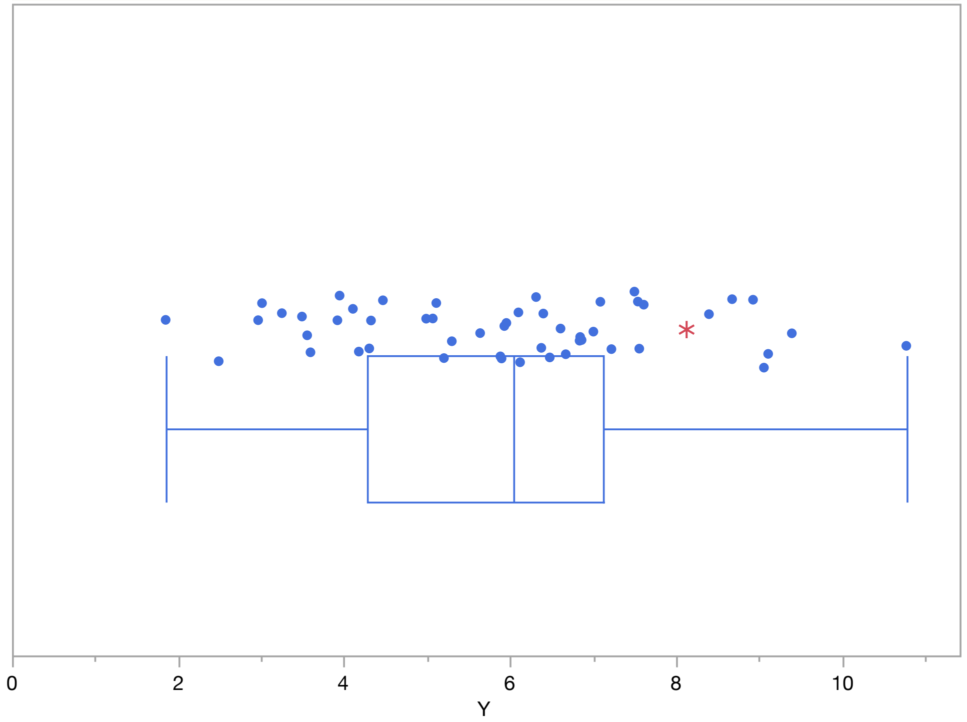

Well = H05

Graph Builder

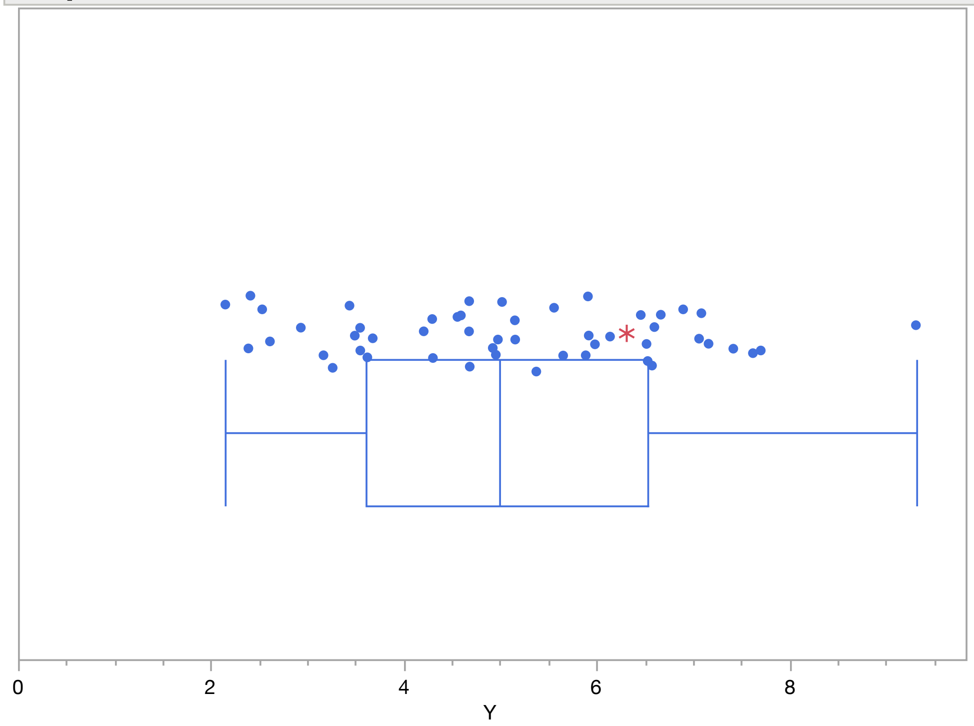

Well = H06

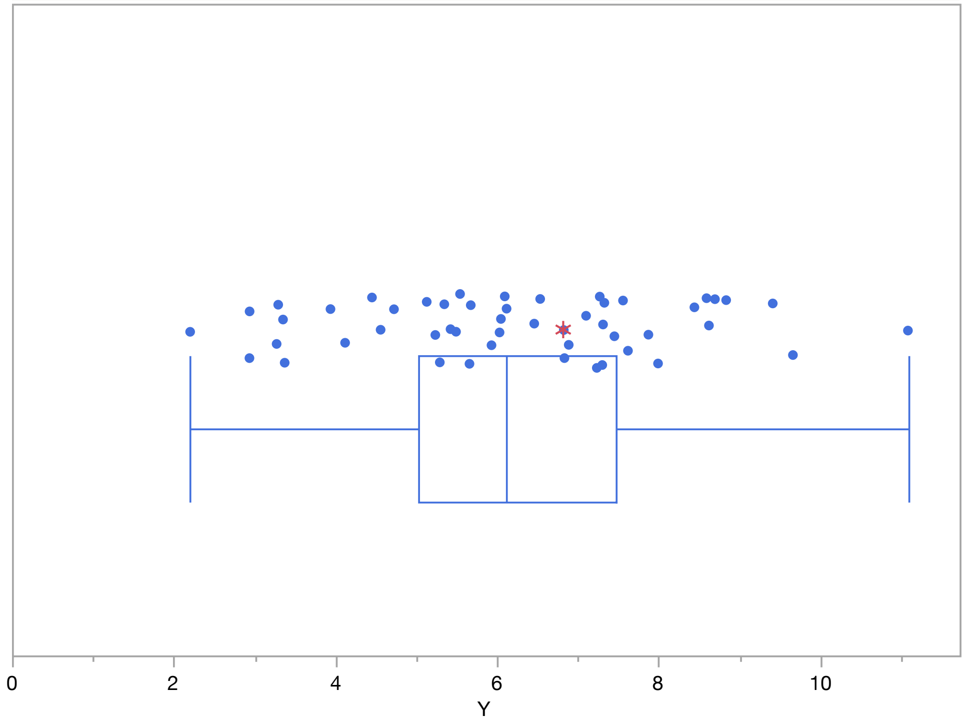

Well = H07

Graph Builder

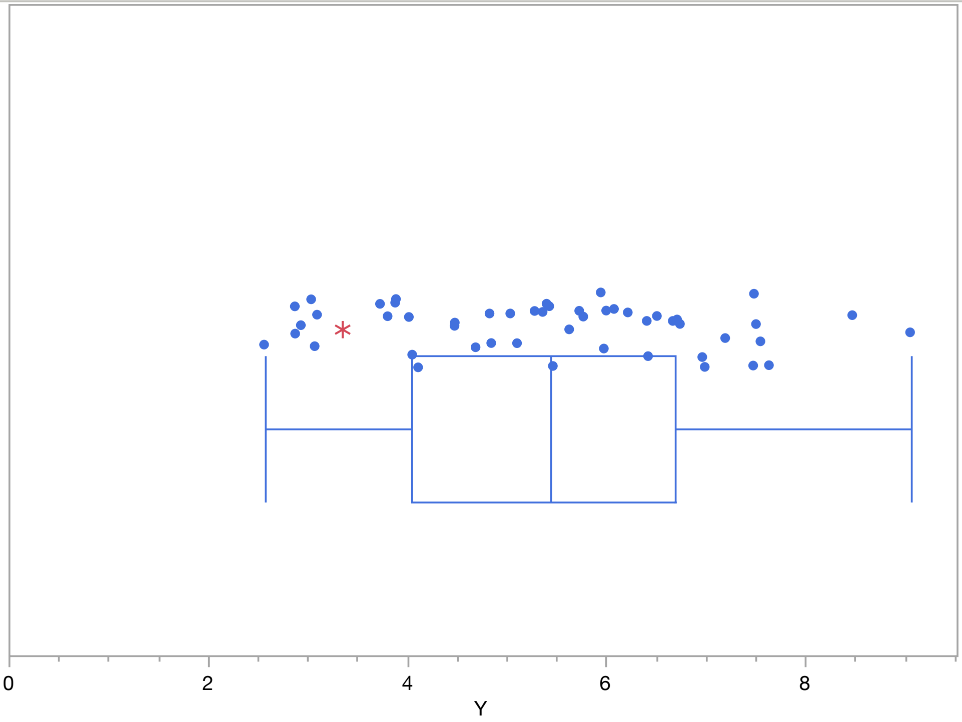

Well = H08

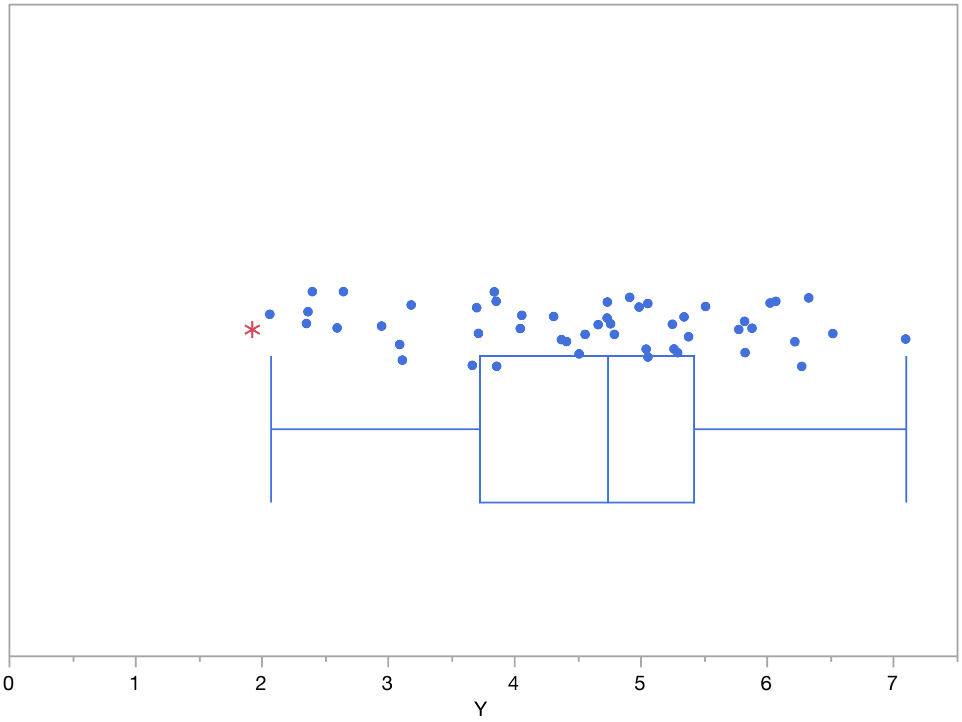

Well = H09

Graph Builder

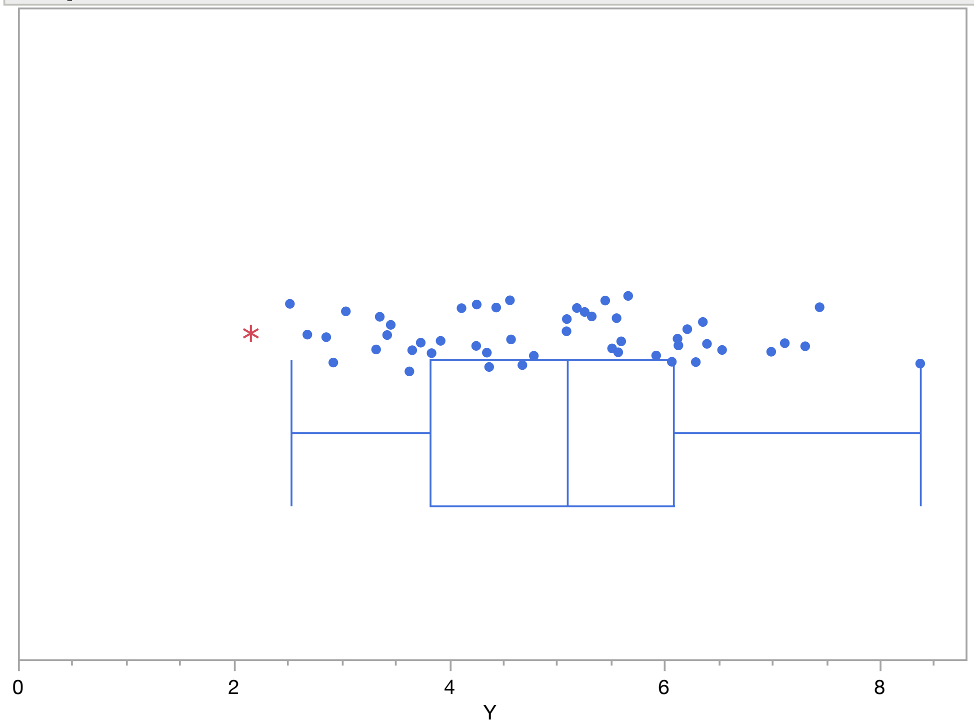

Well = H10

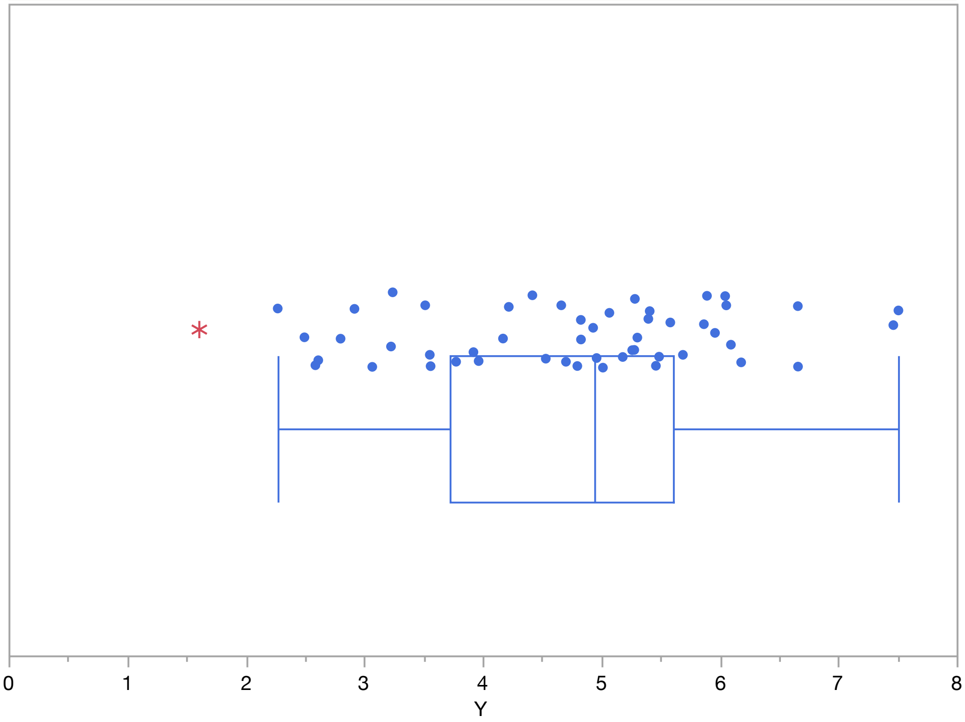

Well = H11

Graph Builder

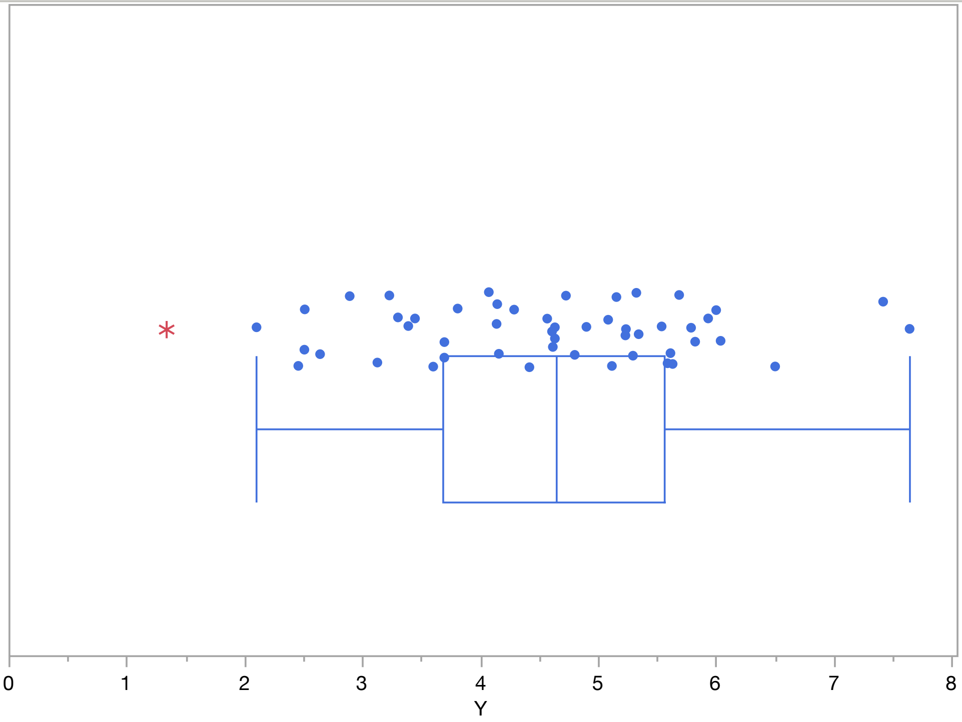

Well = H12

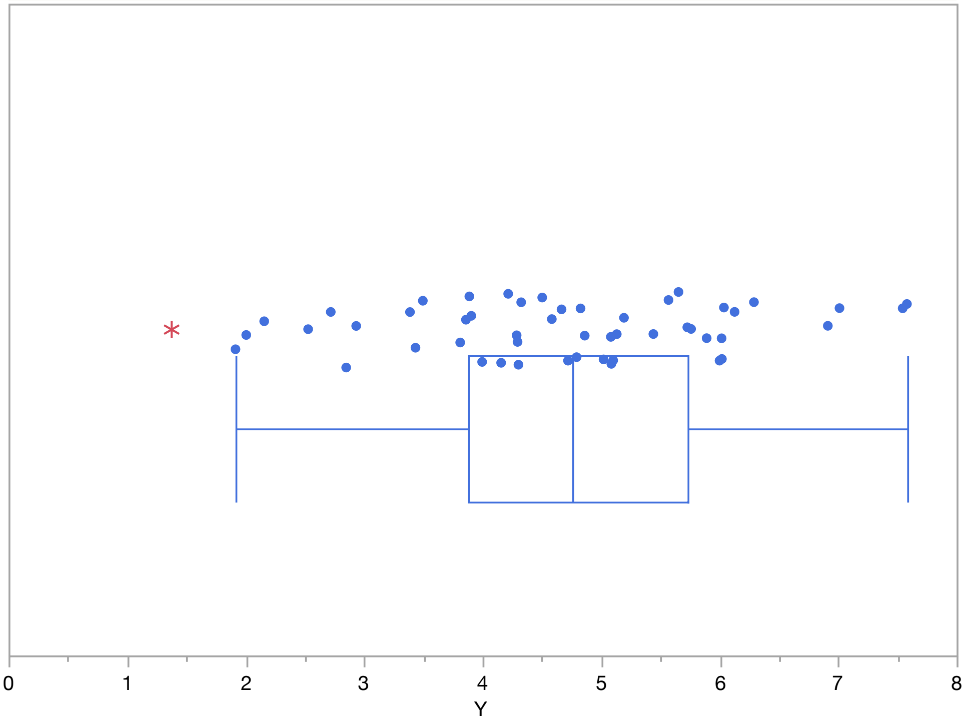

Supplement: Supplementary file 12 — Supplementary Material 12 [file 11011_2025_1546_MOESM12_ESM.pdf]
