# Supplementary material for: A novel approach to metabolic profiling in case models of MECP2-related disorders
Source: Metab Brain Dis. 2025 Feb 13;40(2):124. doi: 10.1007/s11011-025-01546-5 (PMC11825590; doi:10.1007/s11011-025-01546-5)

## Graph Builder

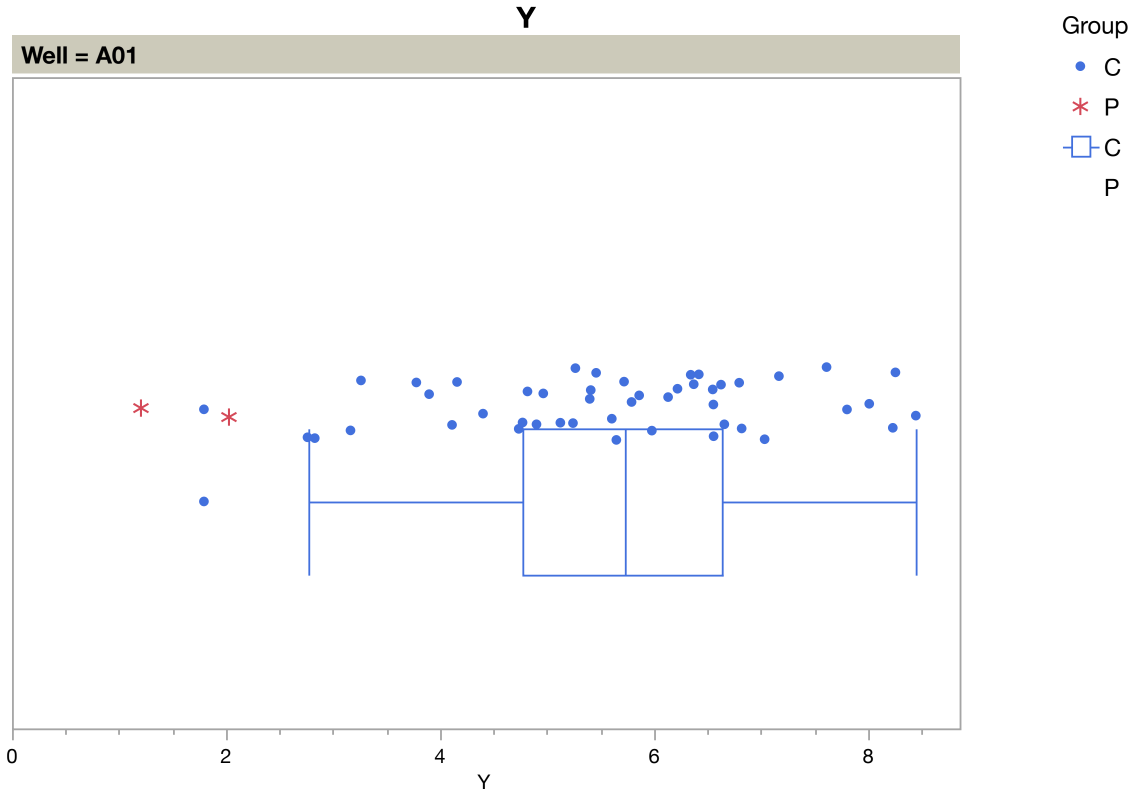


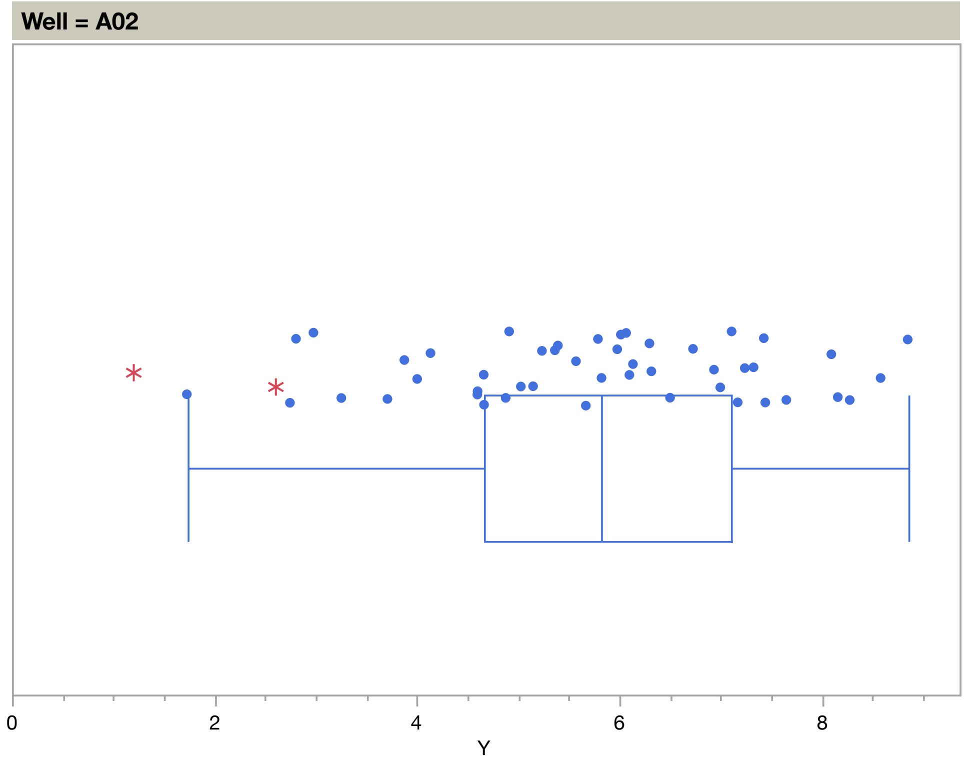


Well = A03

## Graph Builder

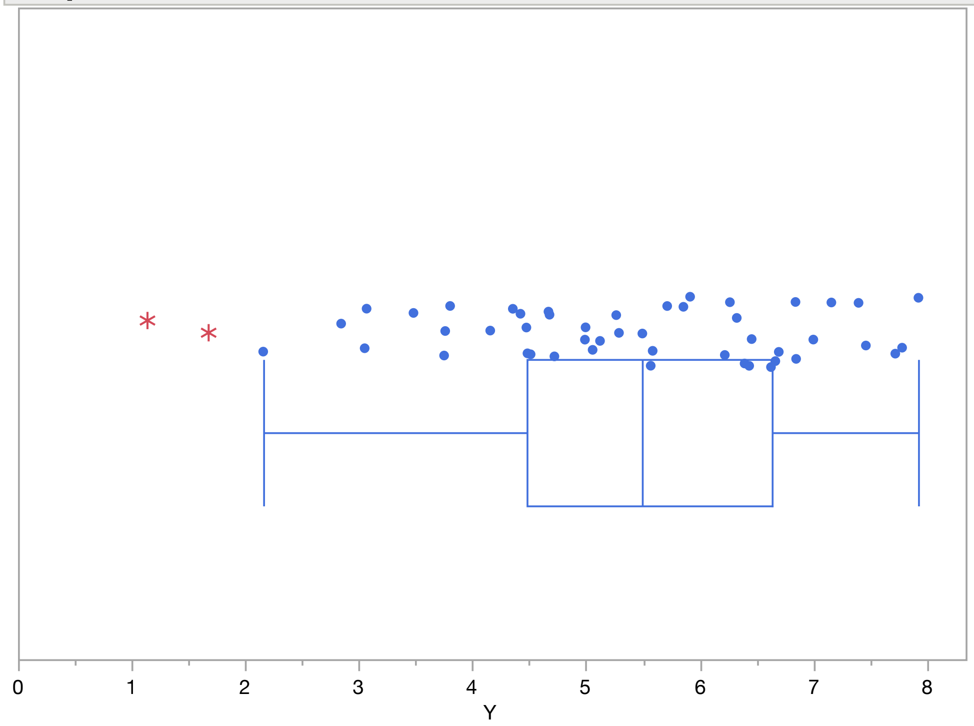


**Well = A04**

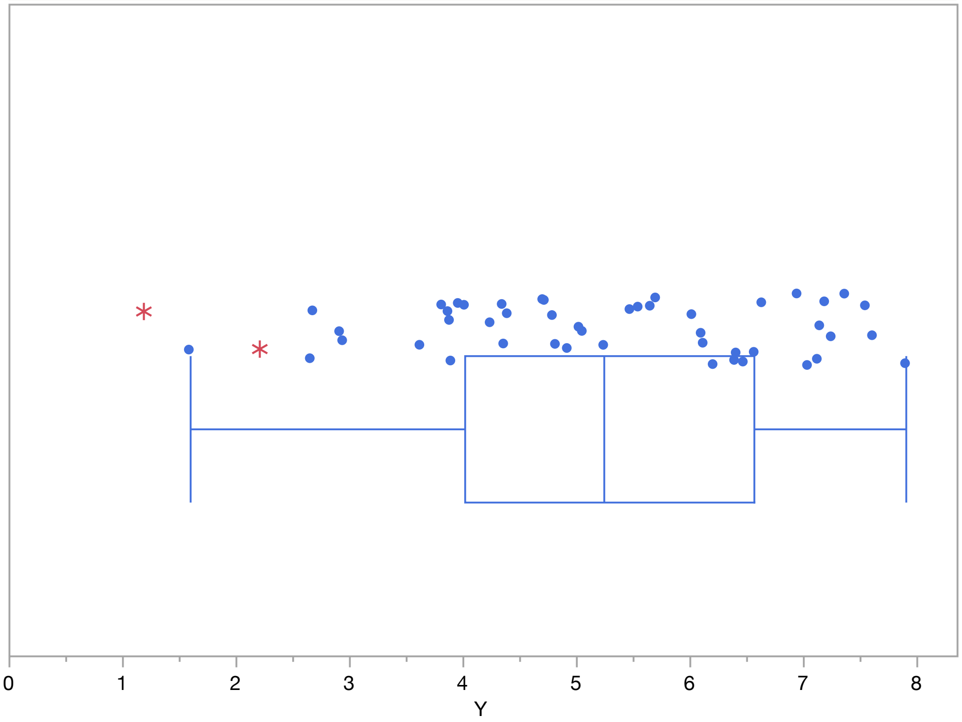


**Well = A05**

## Graph Builder

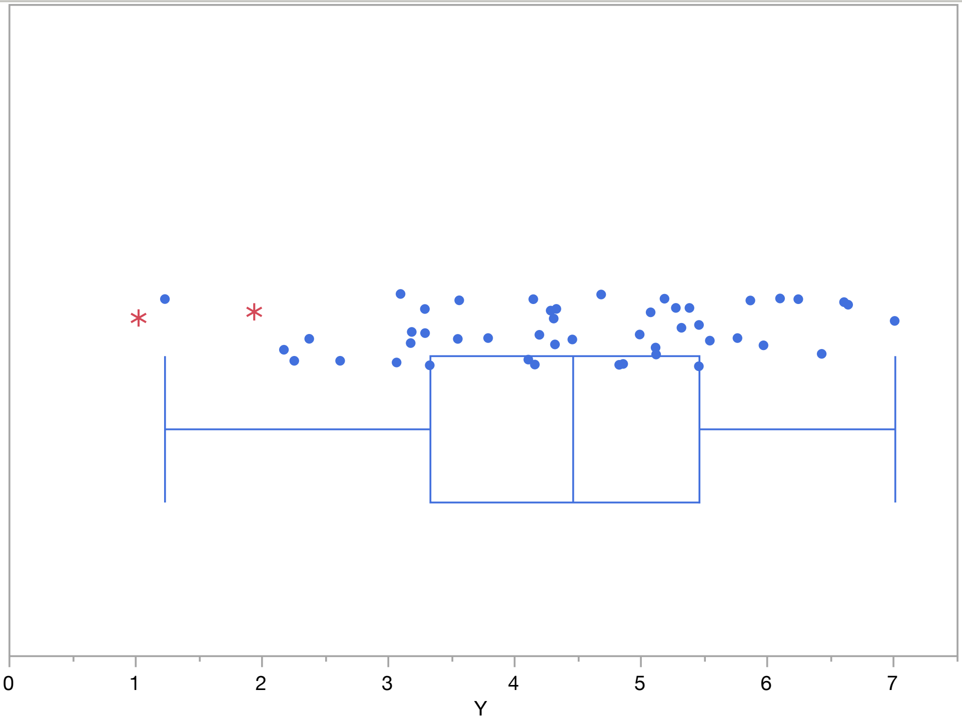


**Well = A06**

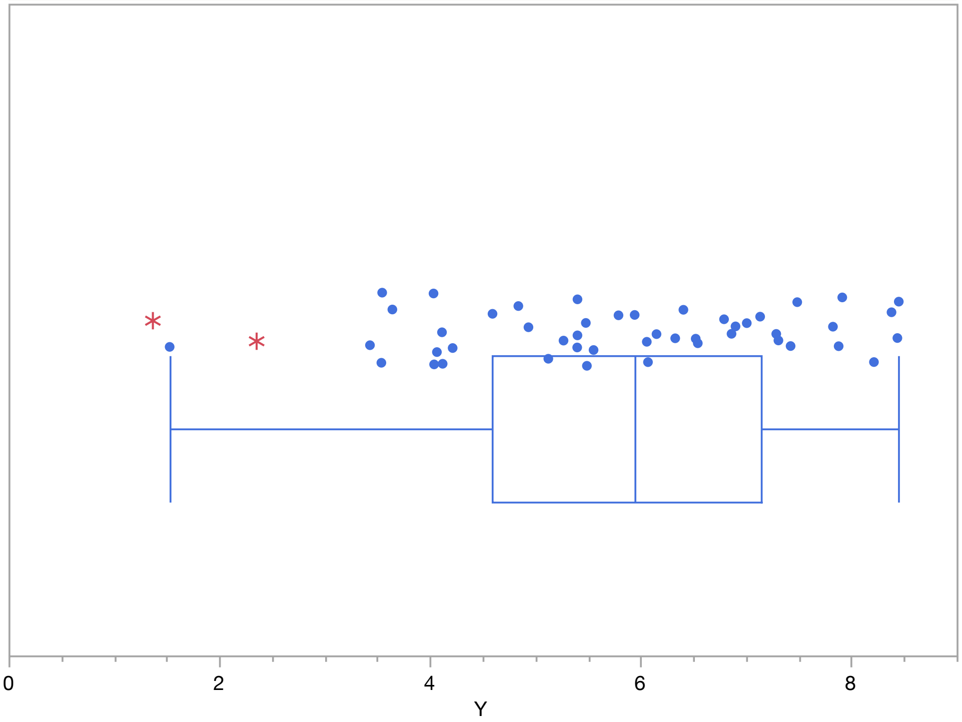


**Well = A07**

## Graph Builder

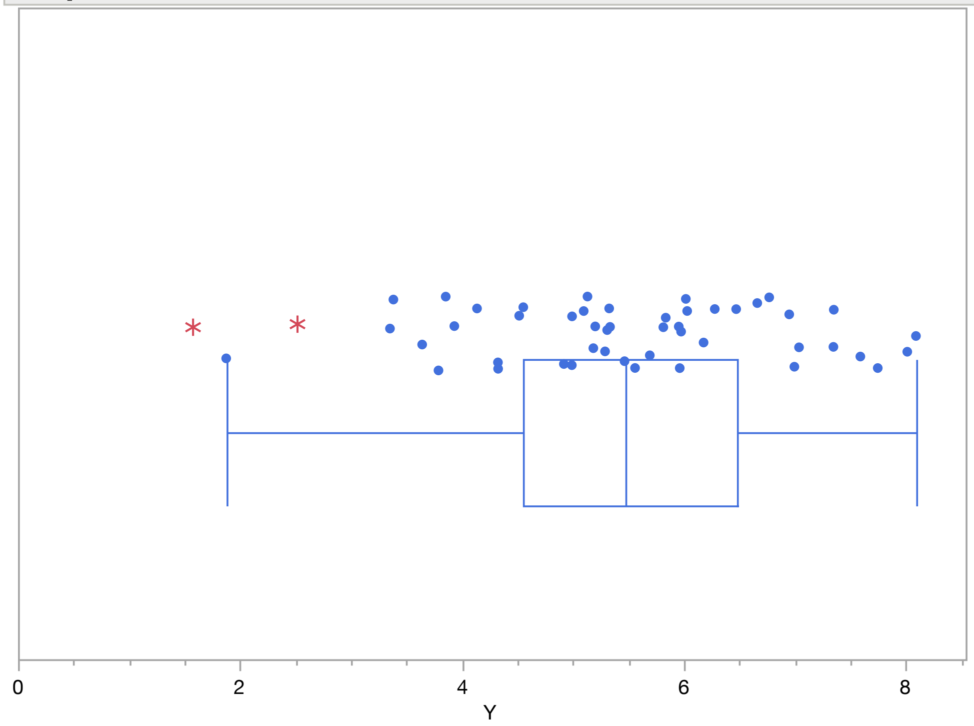


**Well = A08**

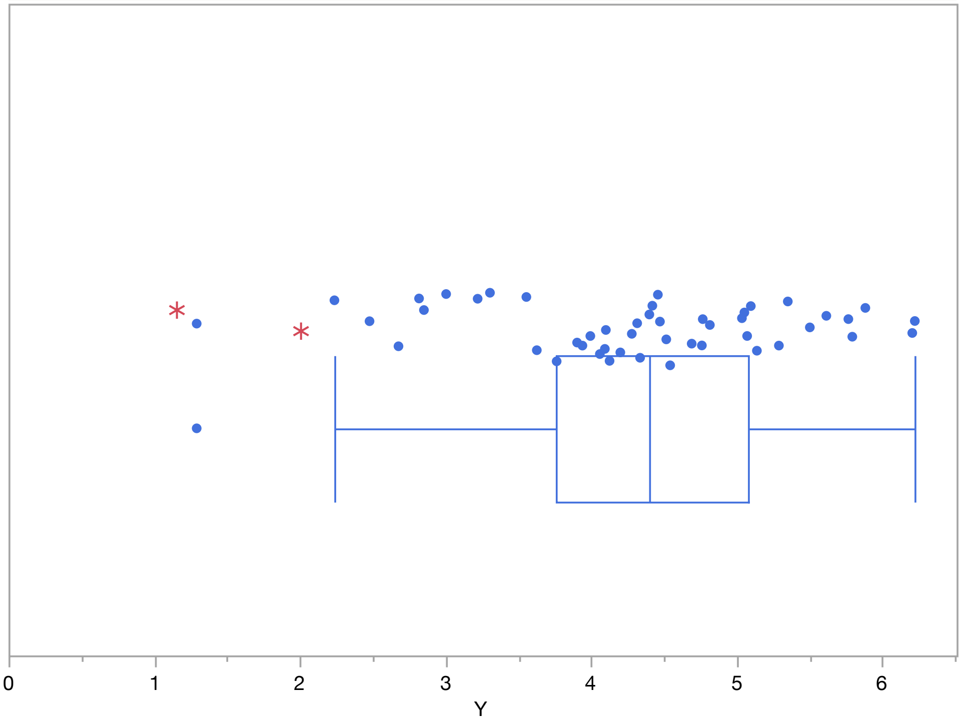


**Well = A09**

## Graph Builder

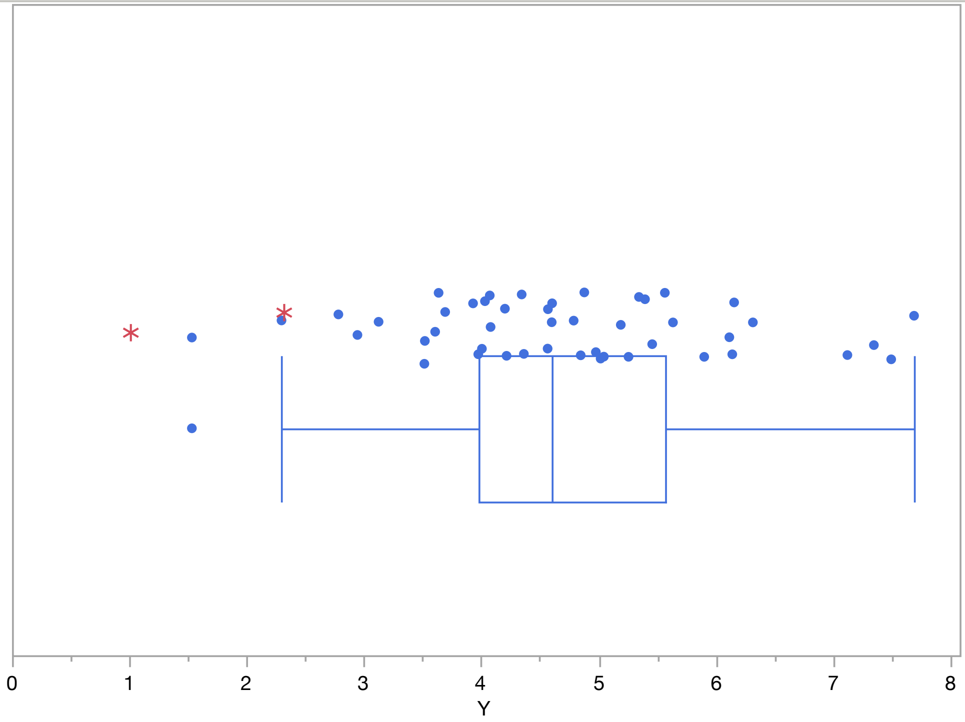


**Well = A10**

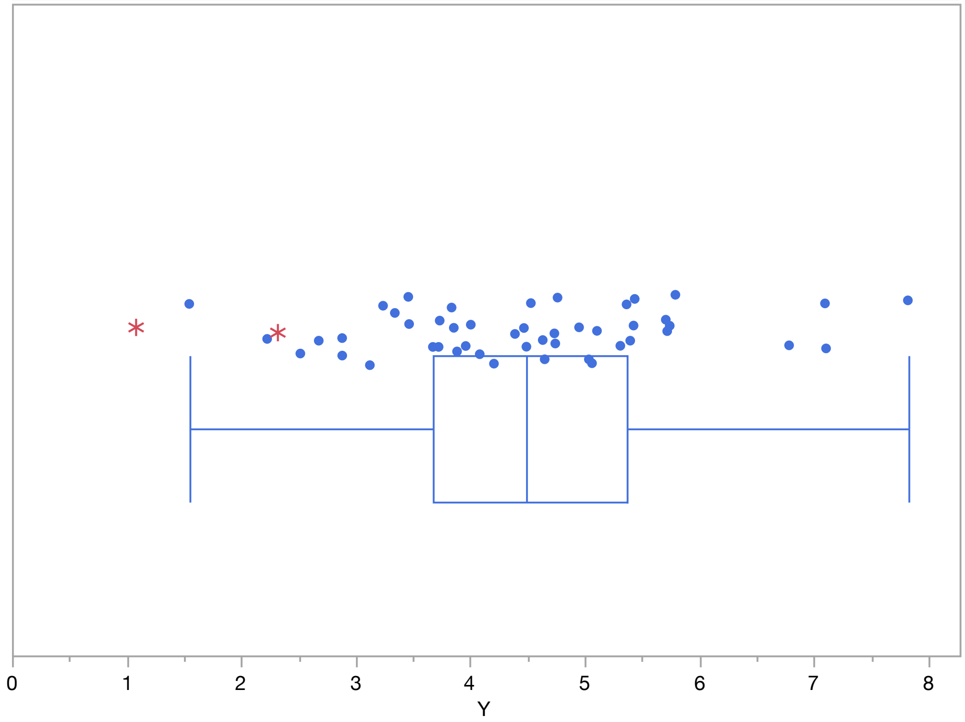


**Well = A11**

## Graph Builder

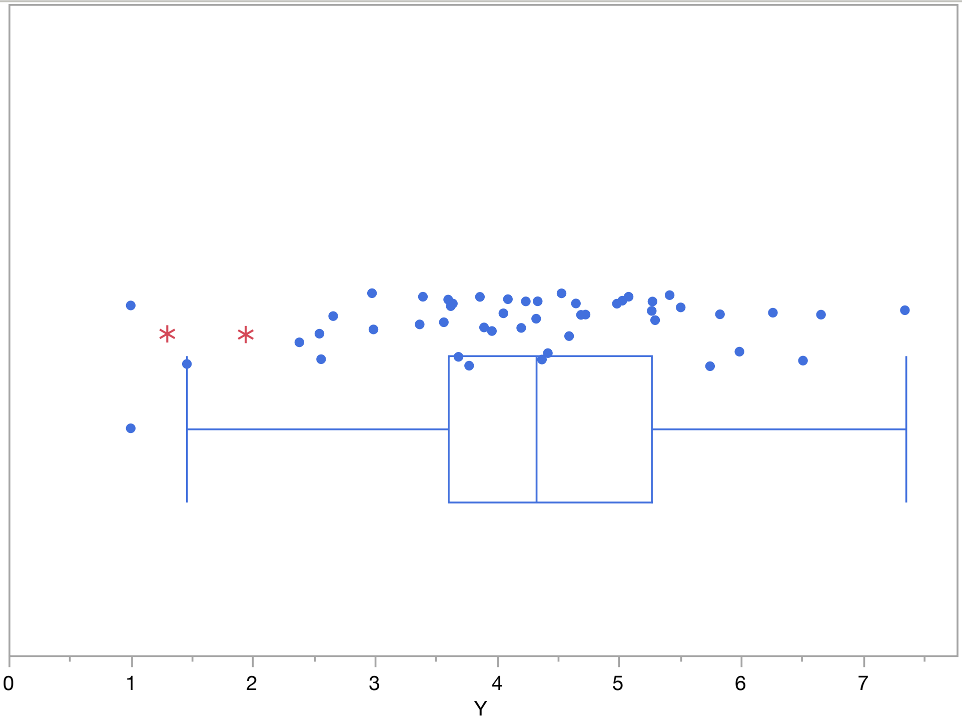


**Well = A12**

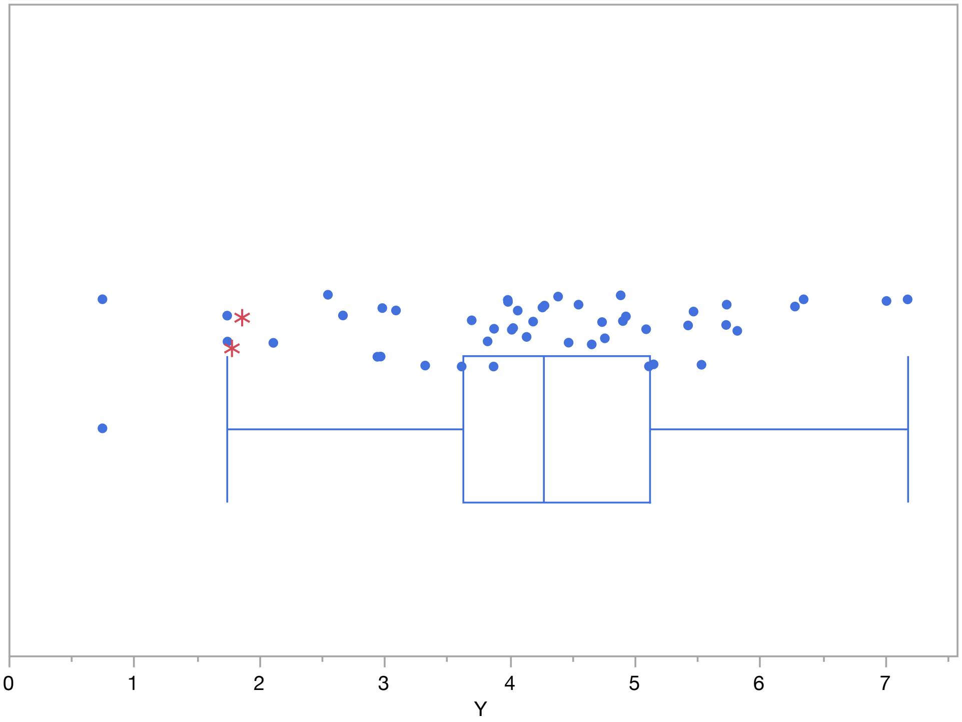


**Well = B01**

## Graph Builder

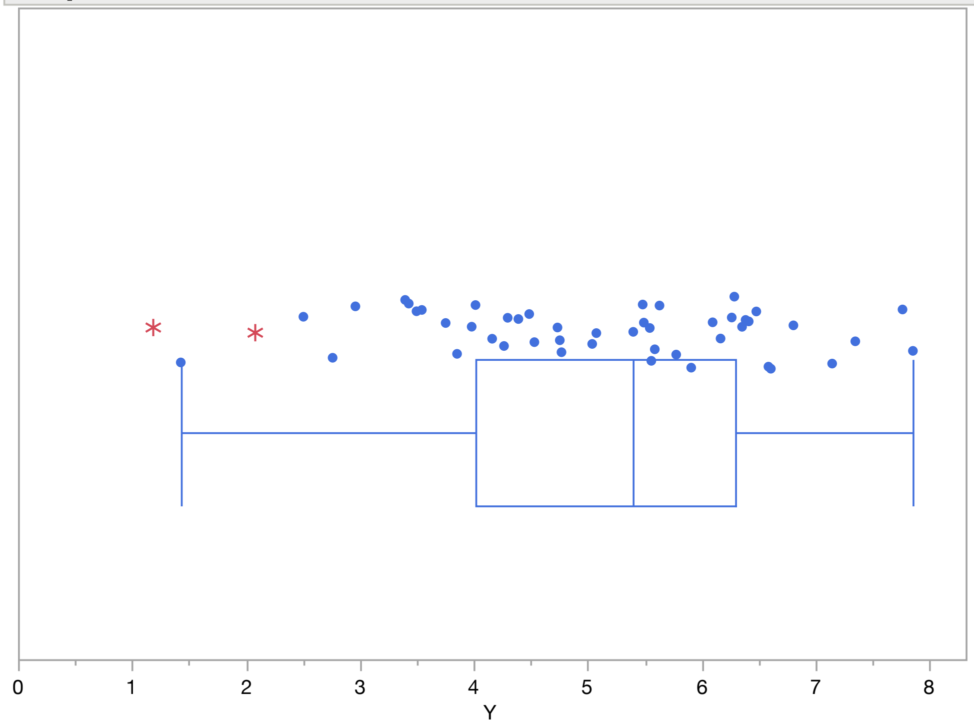


**Well = B02**

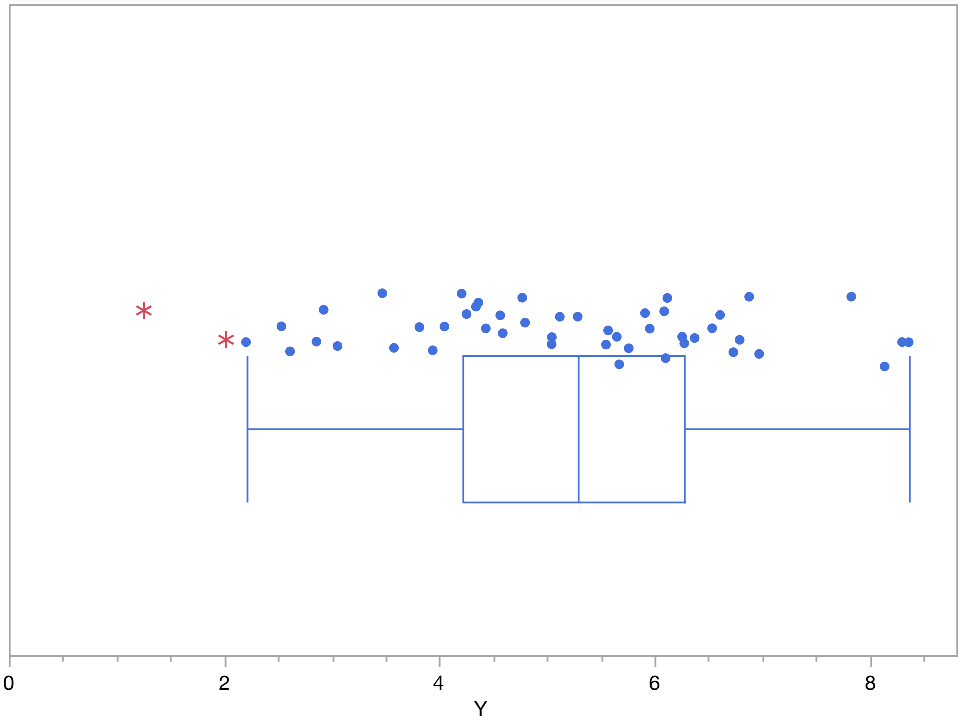


**Well = B03**

## Graph Builder

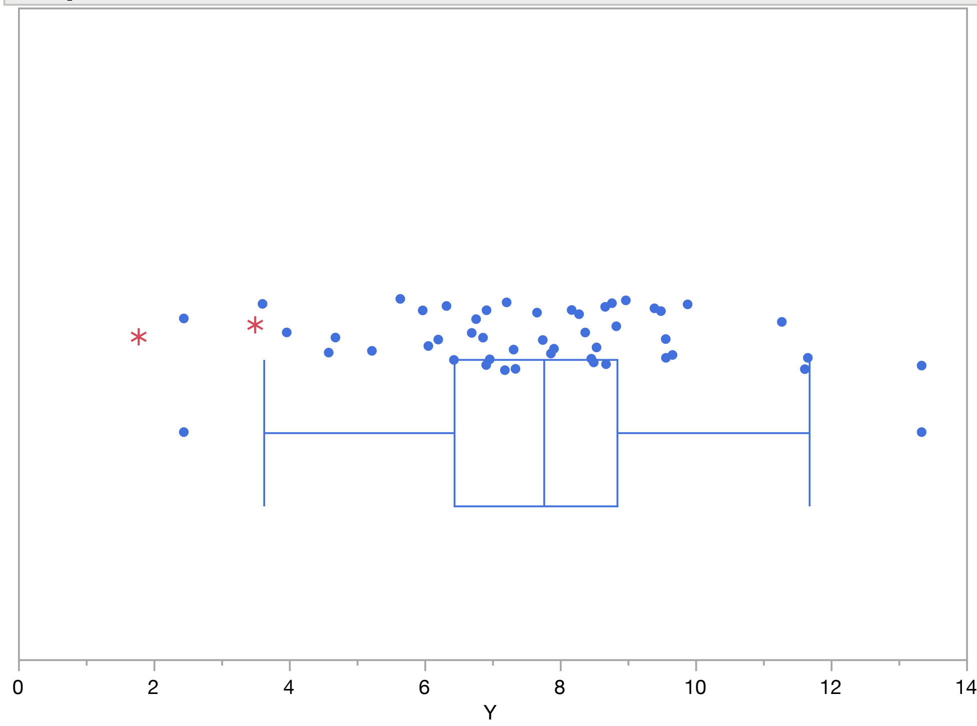


**Well = B04**

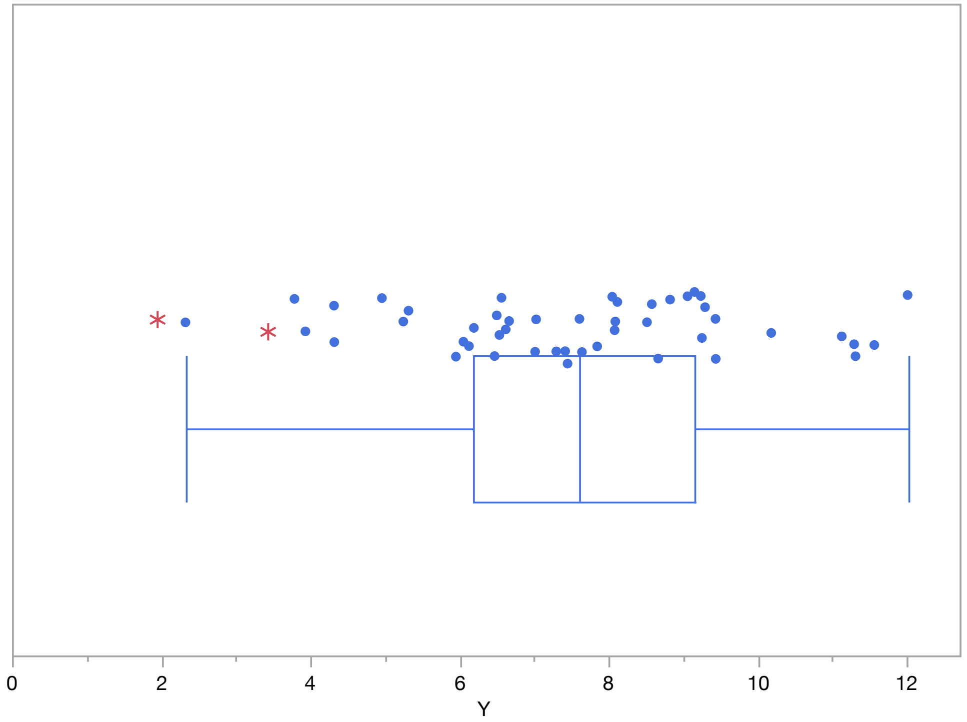


**Well = B05**

## Graph Builder

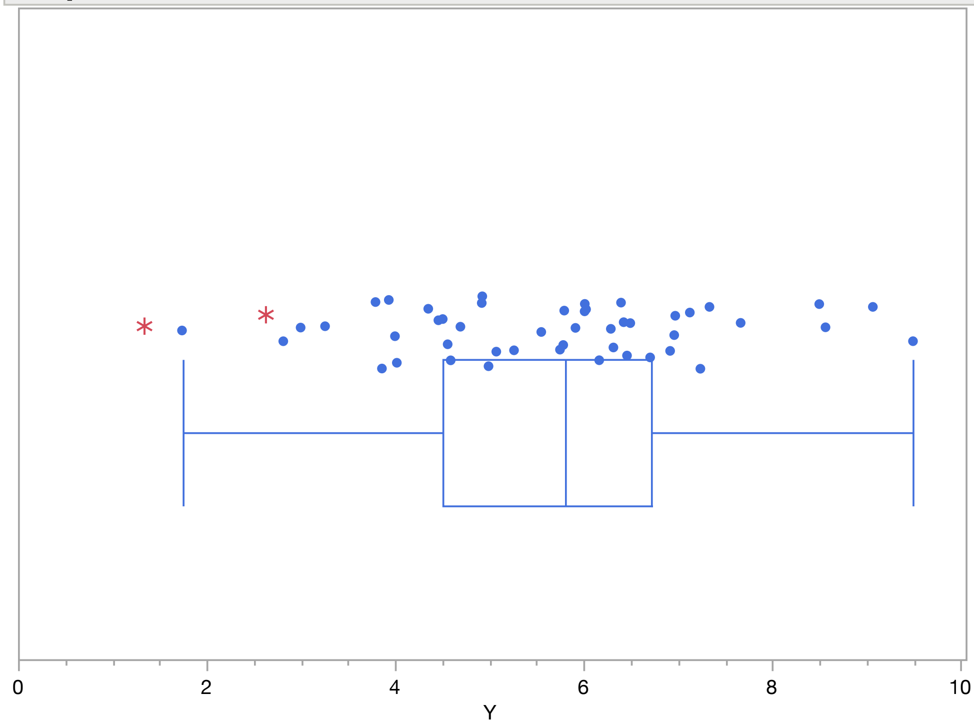


**Well = B06**

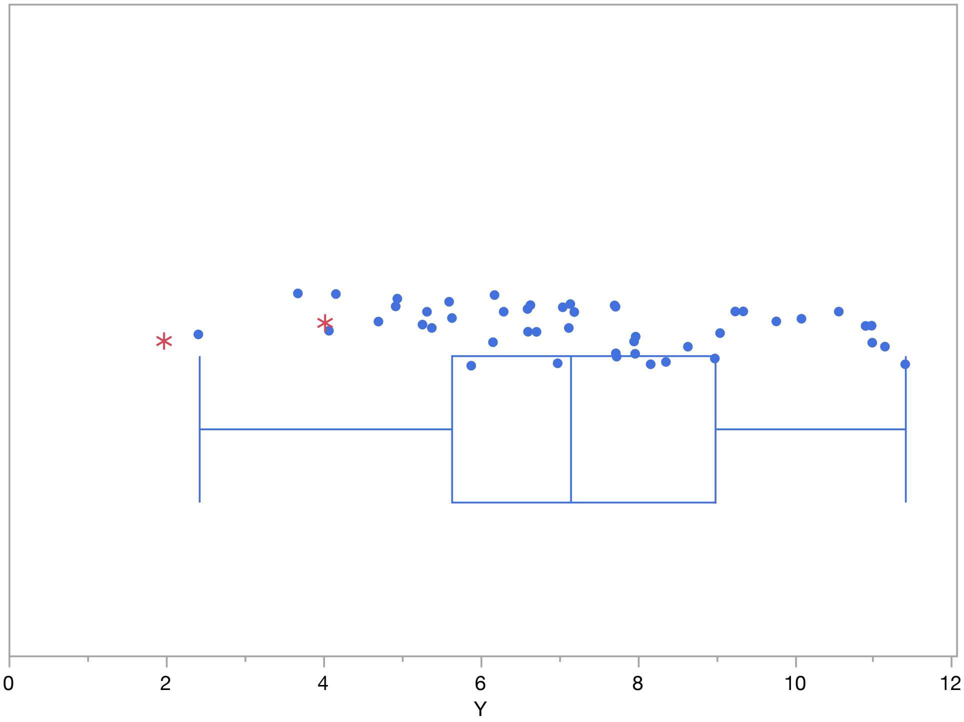


**Well = B07**

## Graph Builder

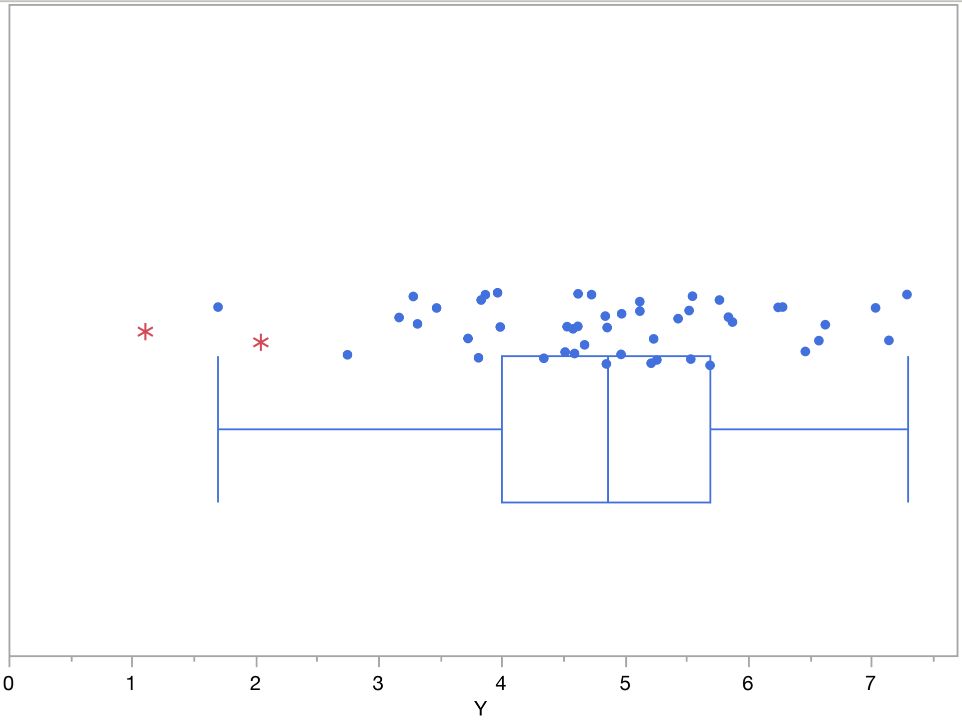


**Well = B08**

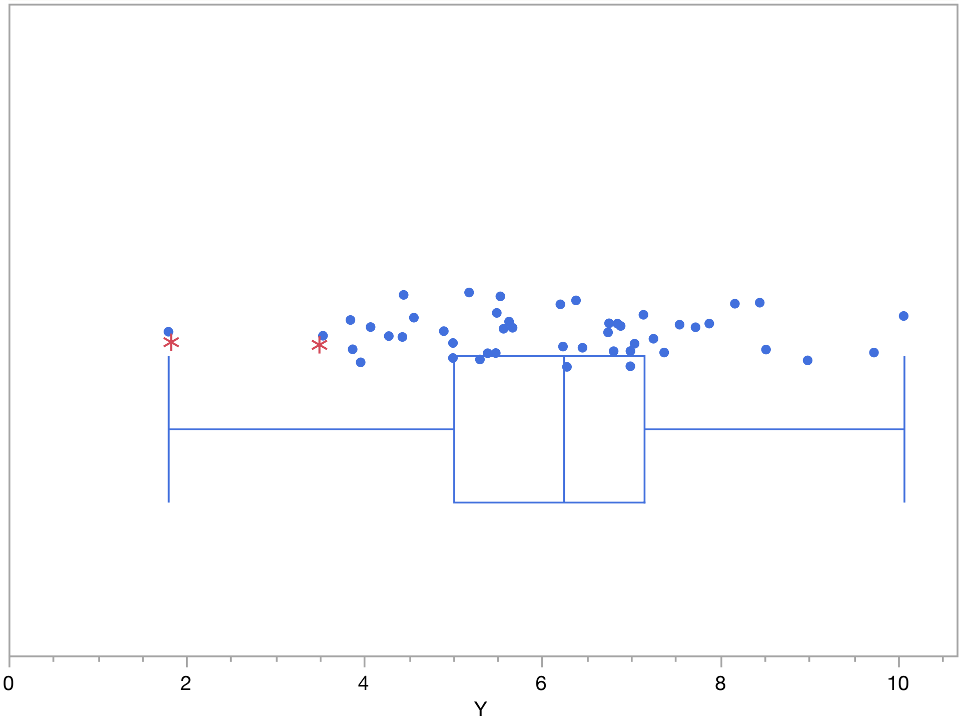


**Well = B09**

## Graph Builder

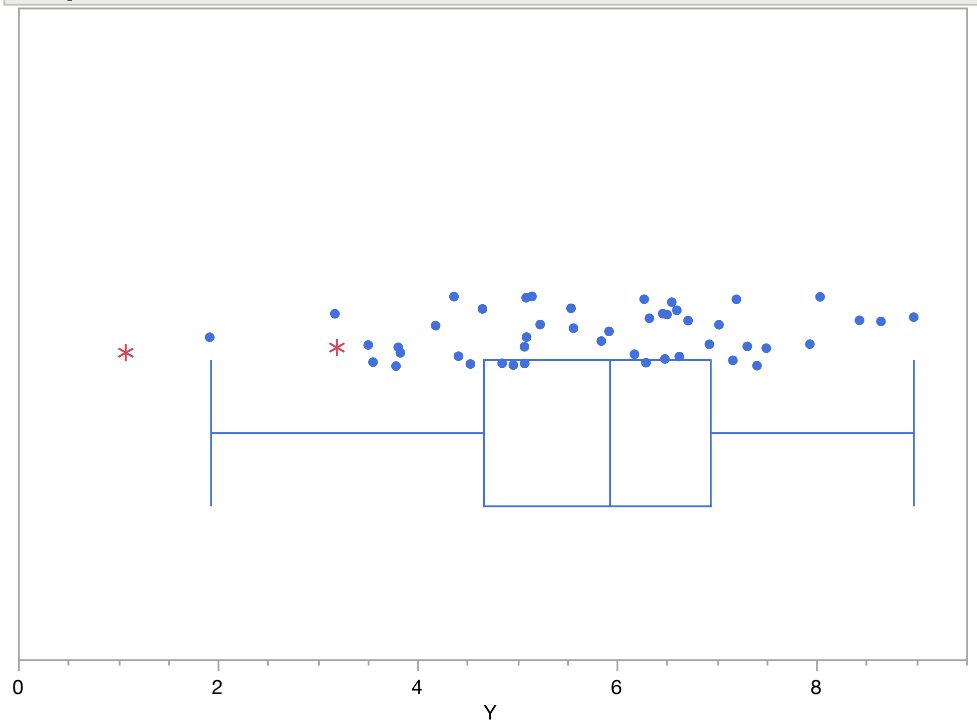


**Well = B10**

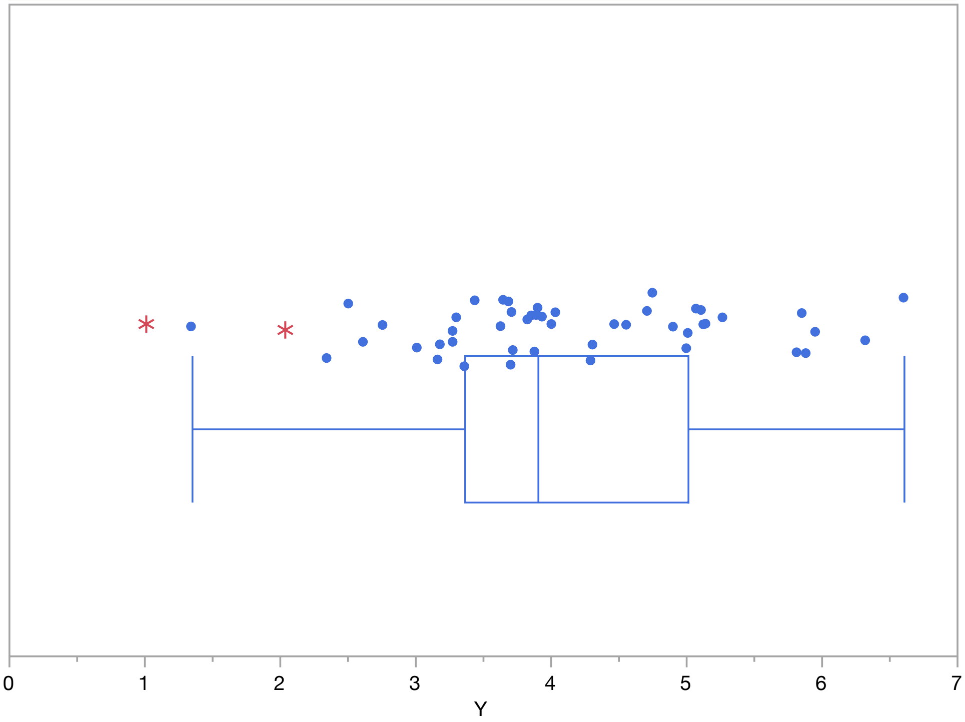


**Well = B11**

## Graph Builder

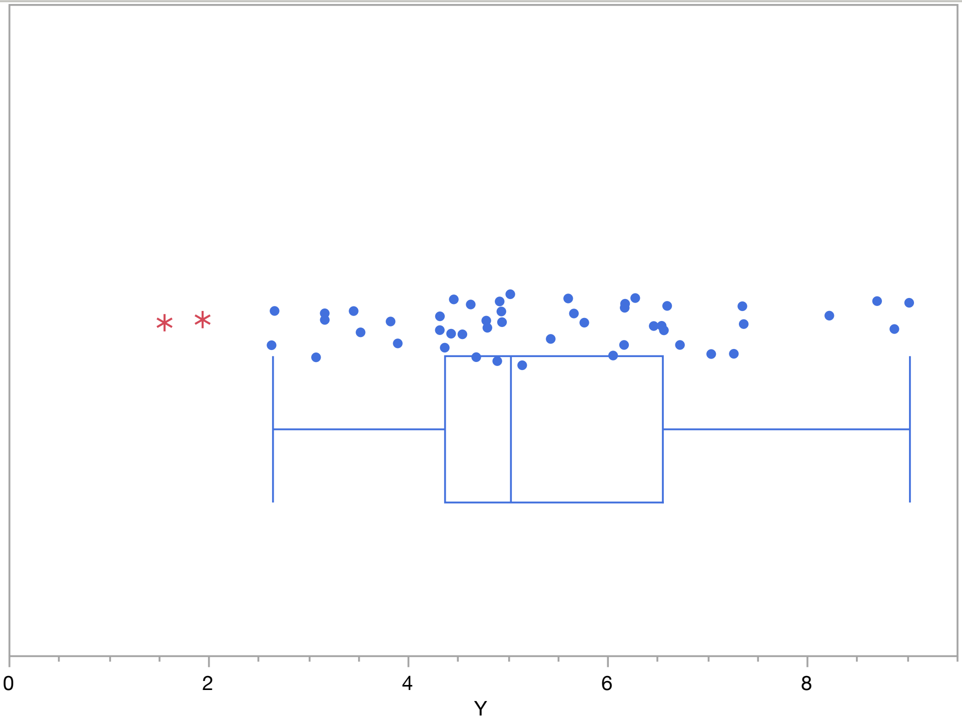


**Well = B12**

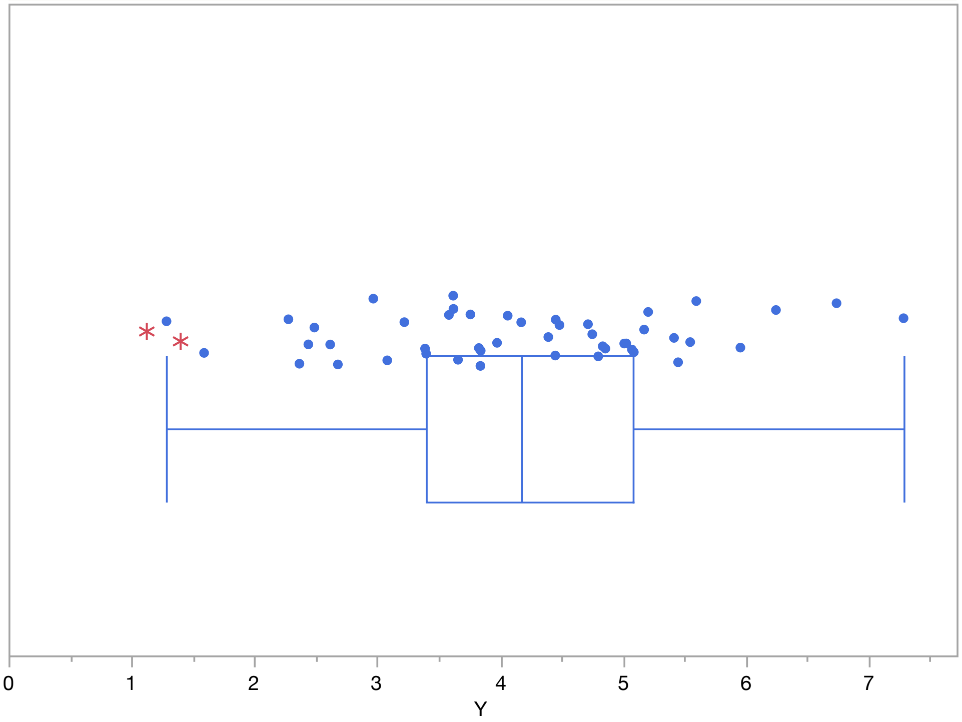


**Well = C01**

## Graph Builder

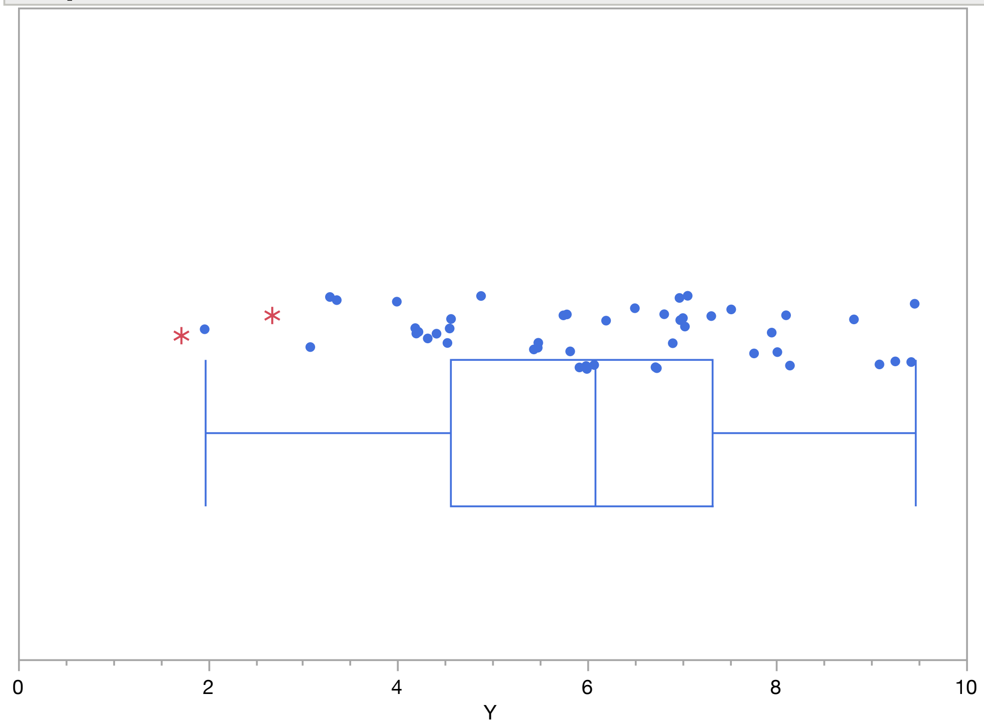


**Well = C02**

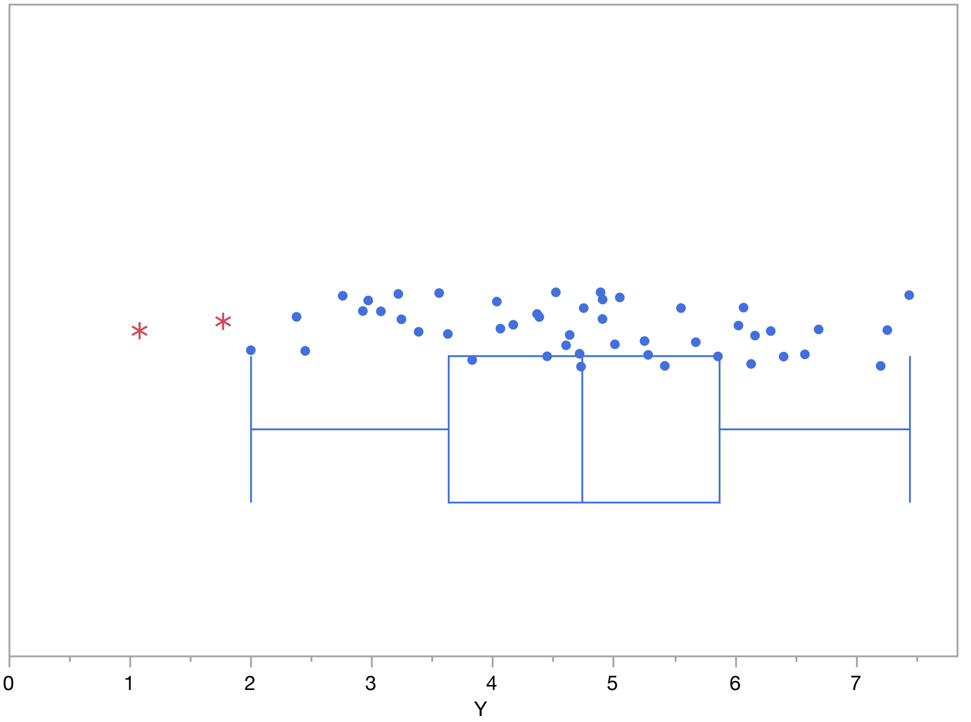


**Well = C03**

## Graph Builder

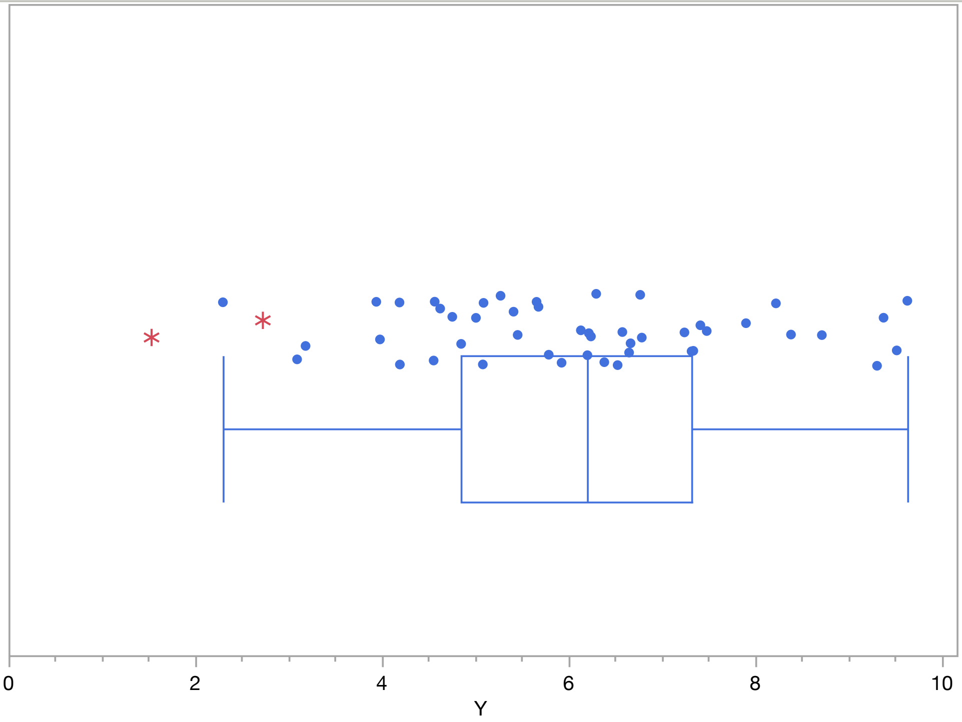


**Well = C04**

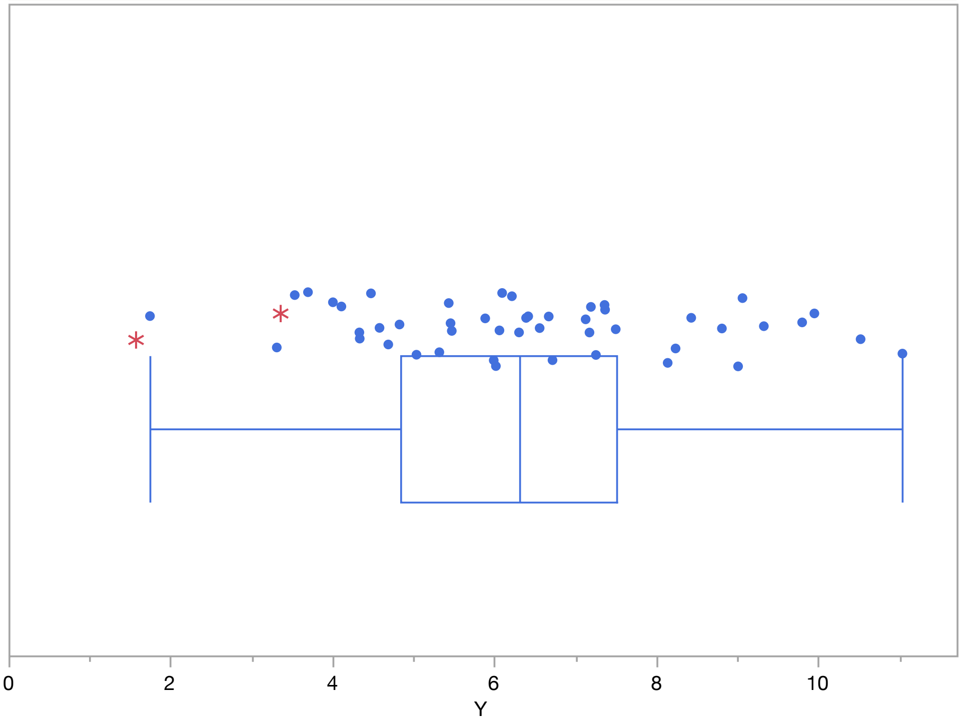


**Well = C05**

## Graph Builder

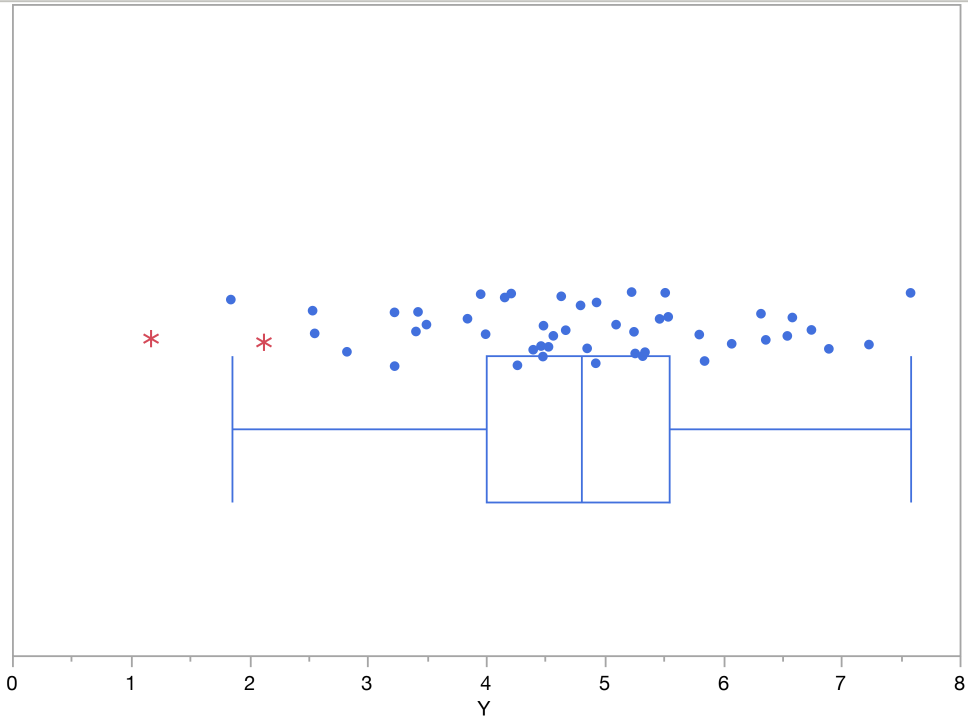


**Well = C06**

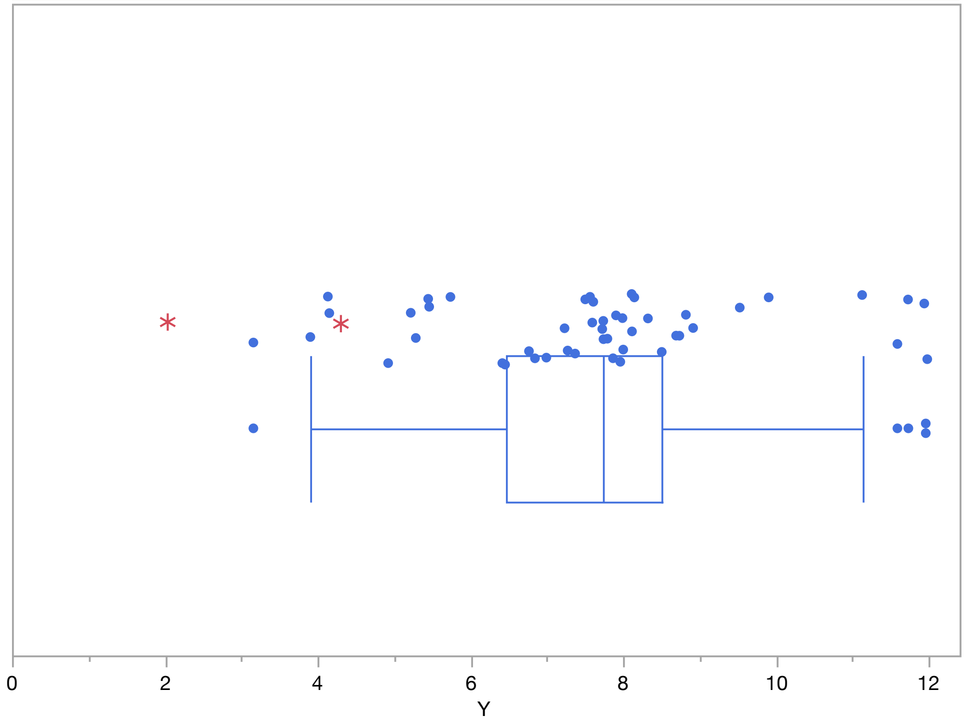


**Well = C07**

## Graph Builder

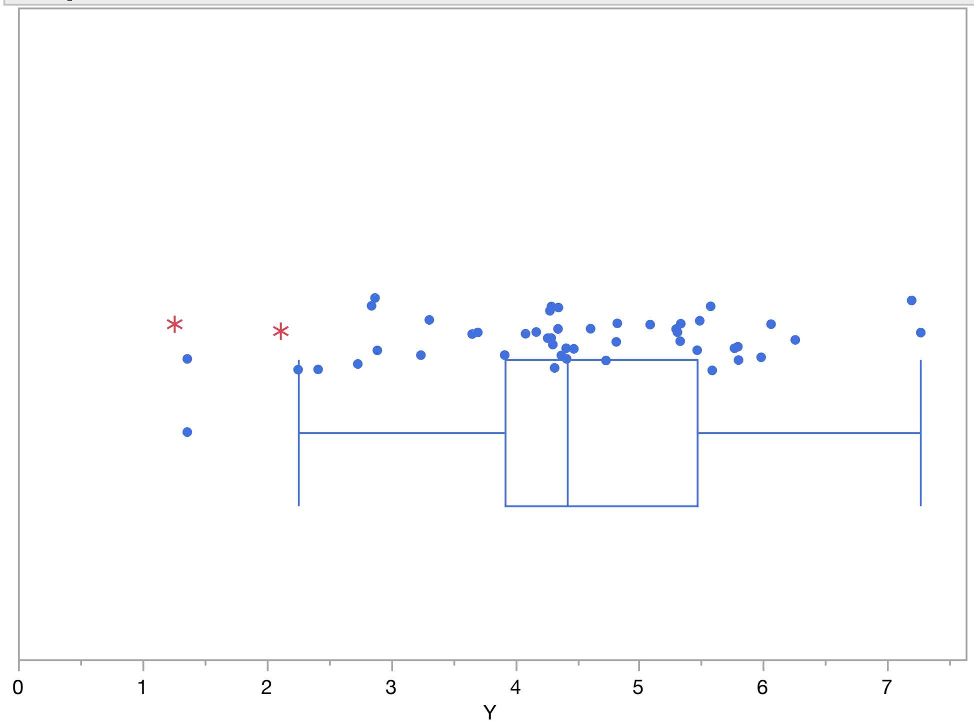


**Well = C08**

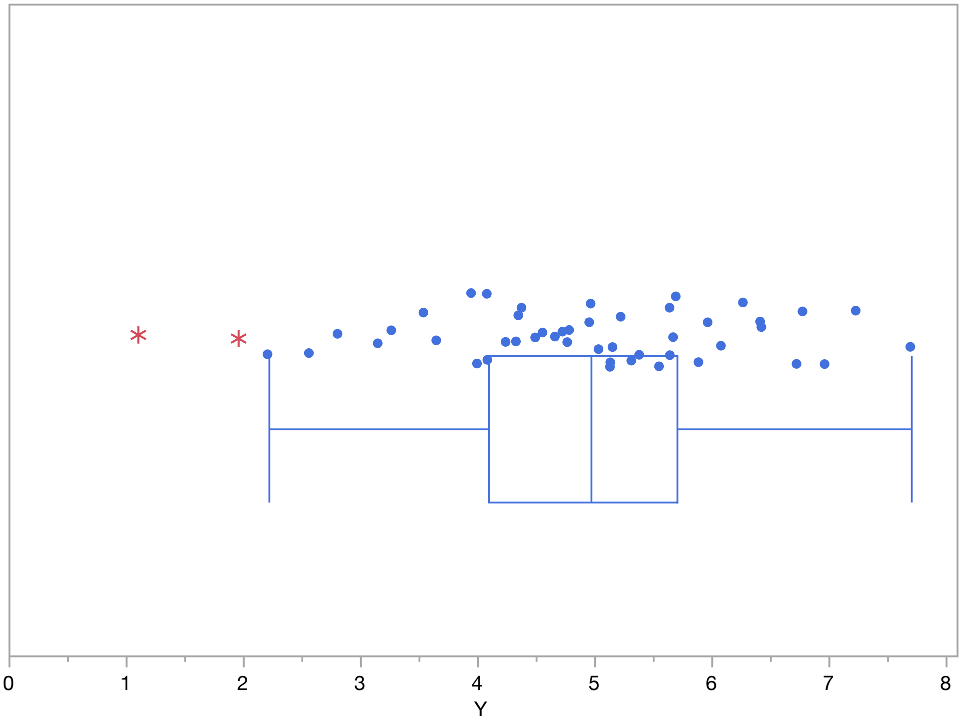


**Well = C09**

## Graph Builder

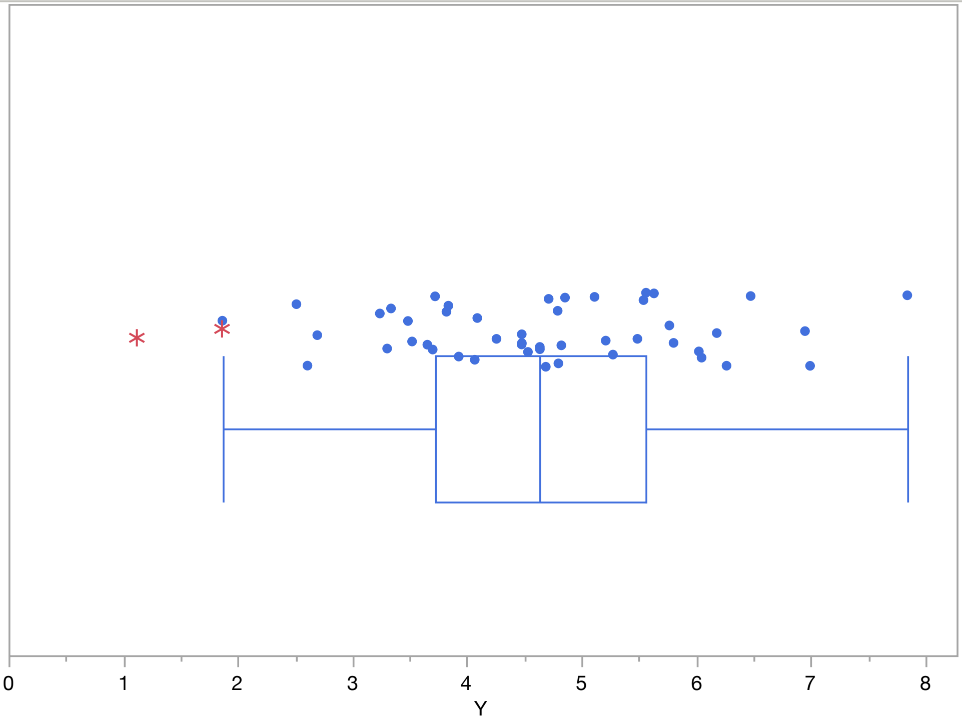


**Well = C10**

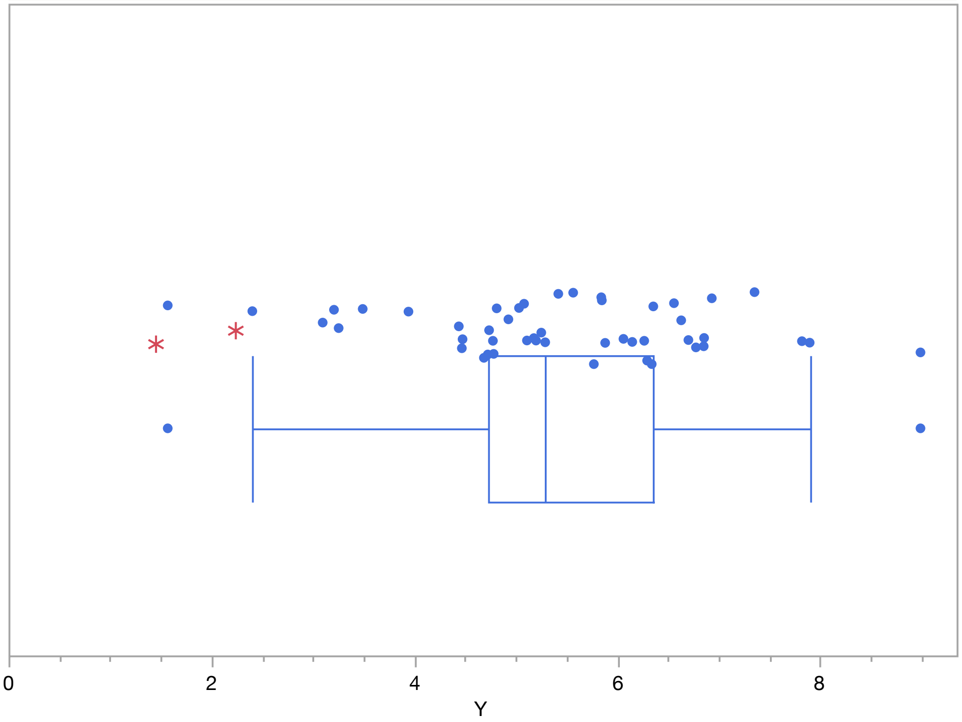


**Well = C11**

## Graph Builder

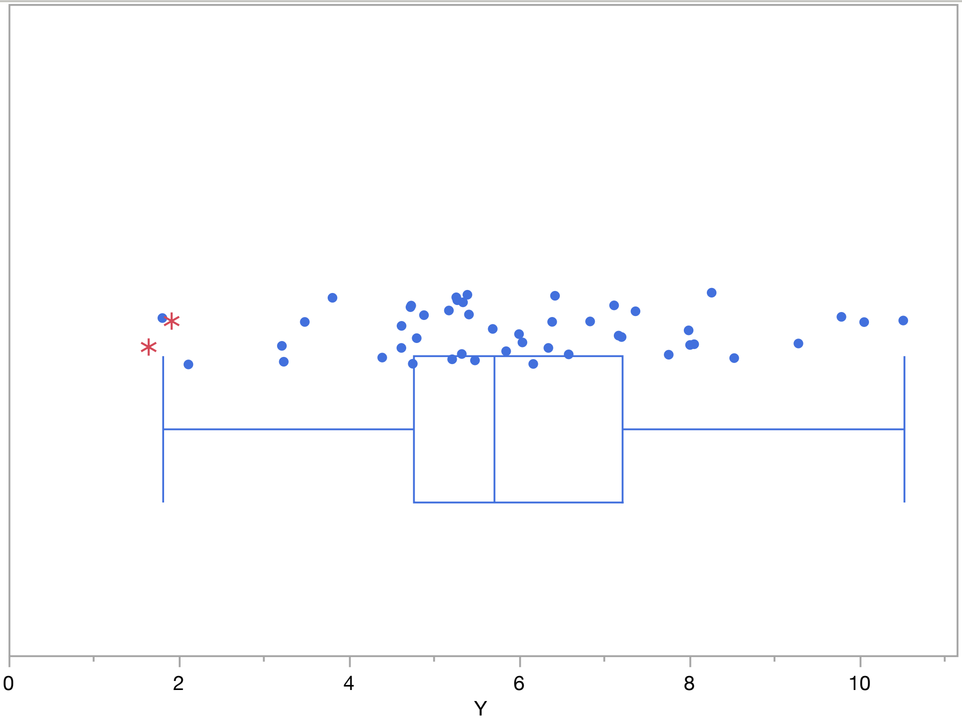


**Well = C12**

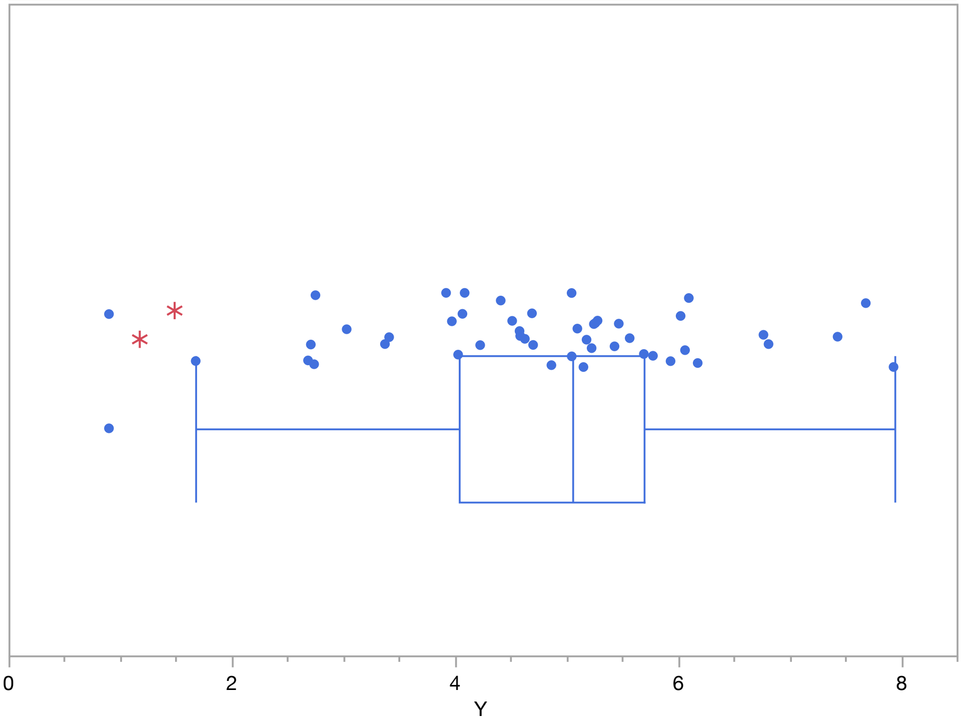


**Well = D01**

## Graph Builder

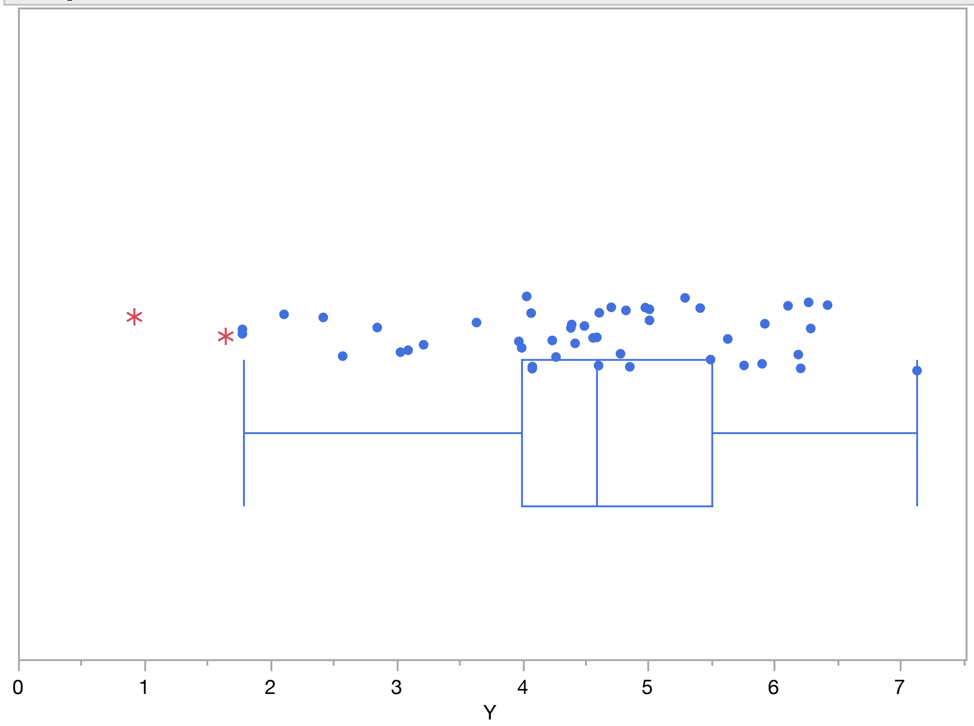


**Well = D02**

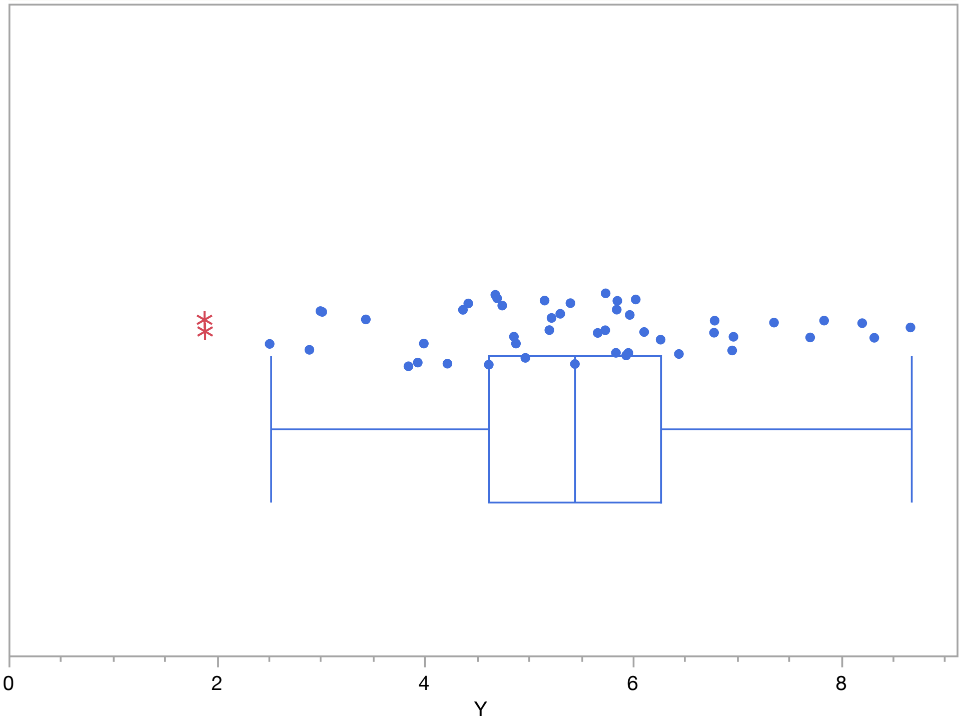


**Well = D03**

## Graph Builder

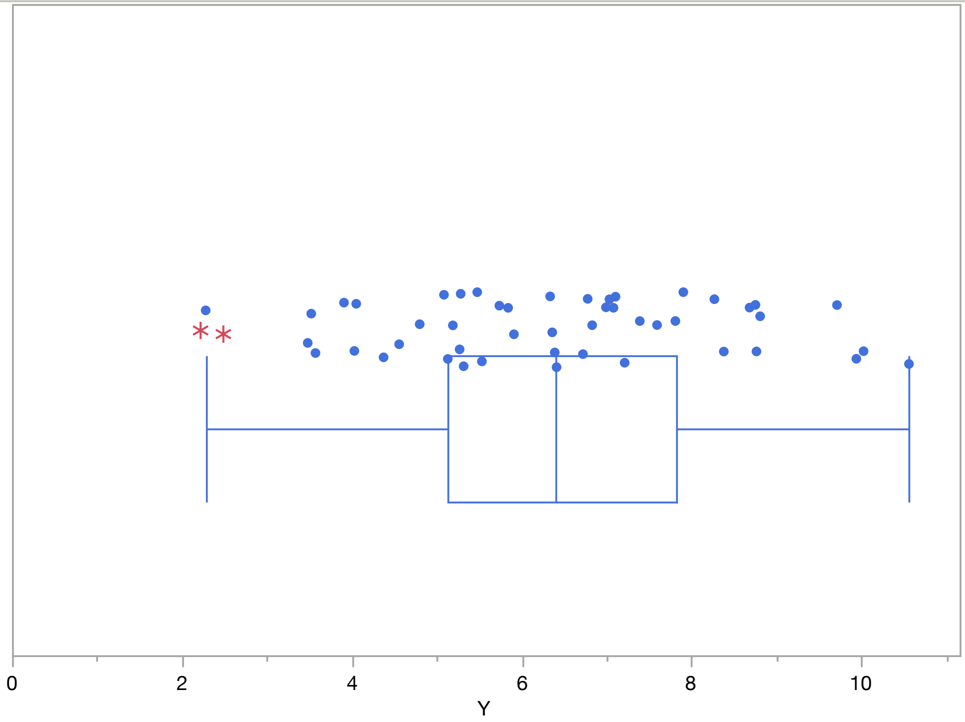


**Well = D04**

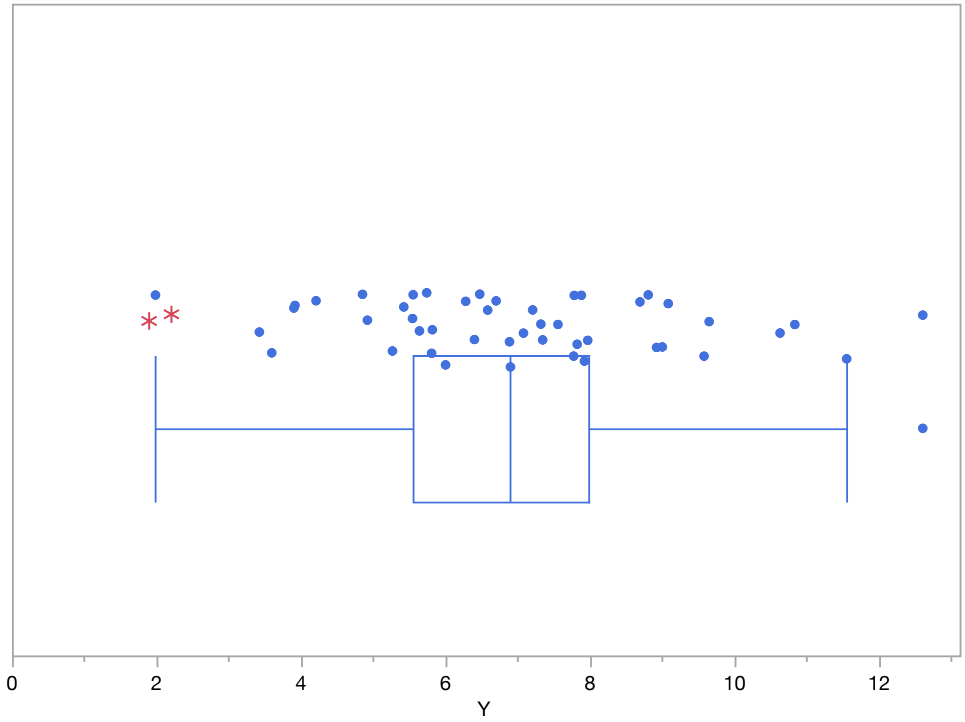


**Well = D05**

## Graph Builder

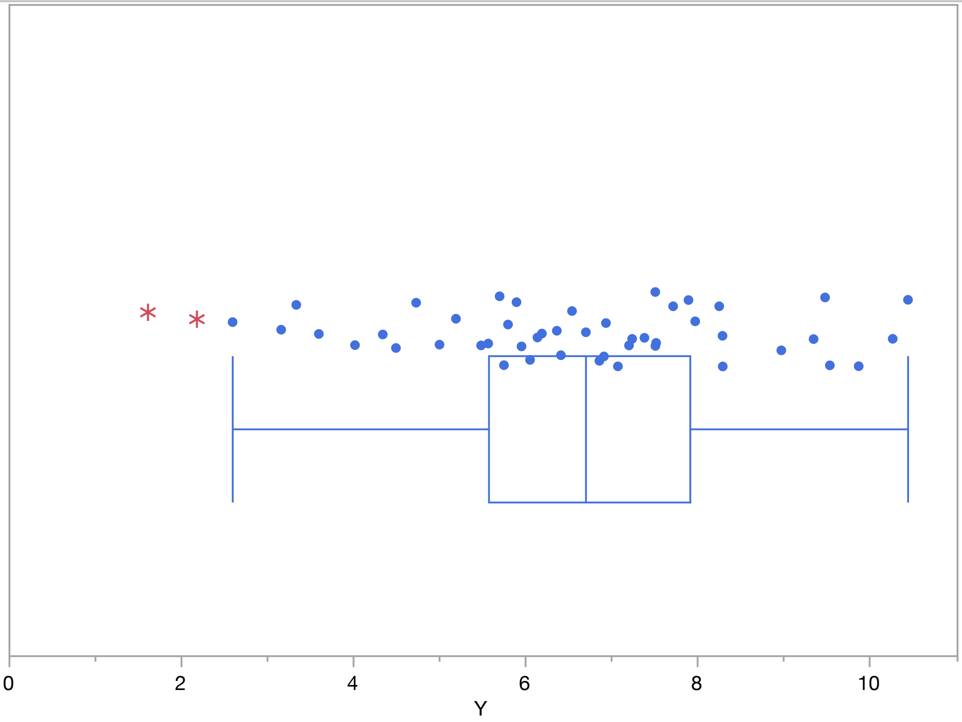


**Well = D06**

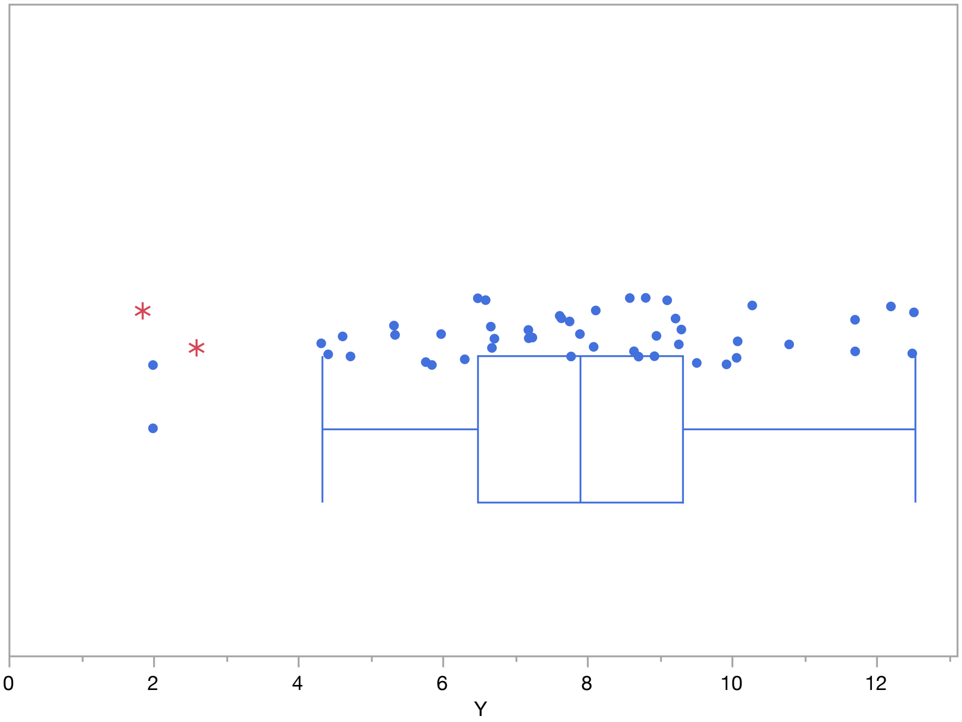


**Well = D07**

## Graph Builder

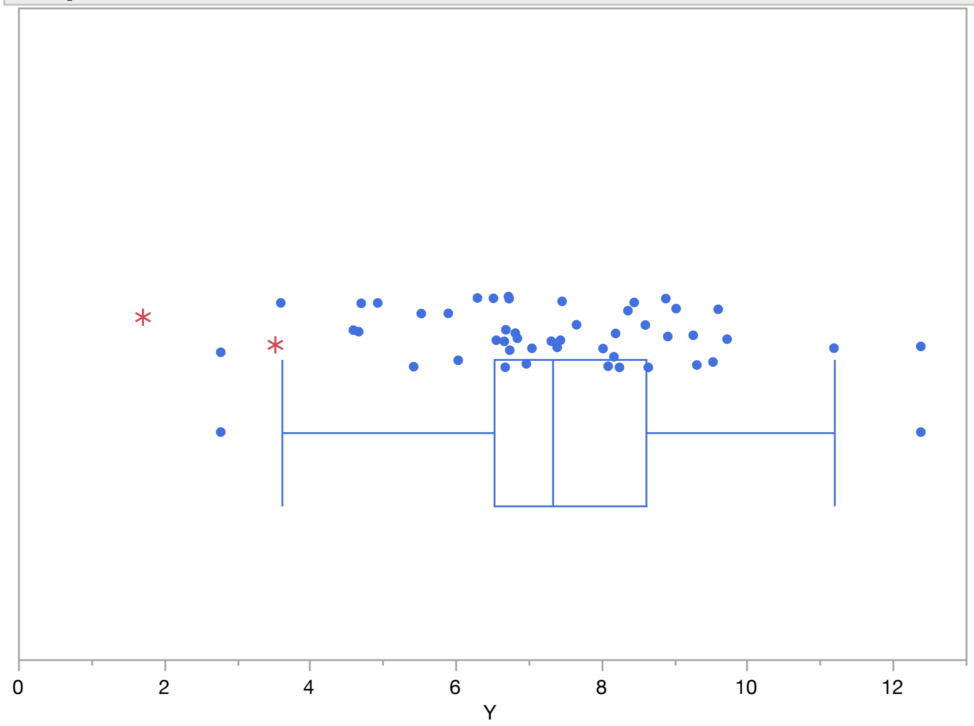


**Well = D08**

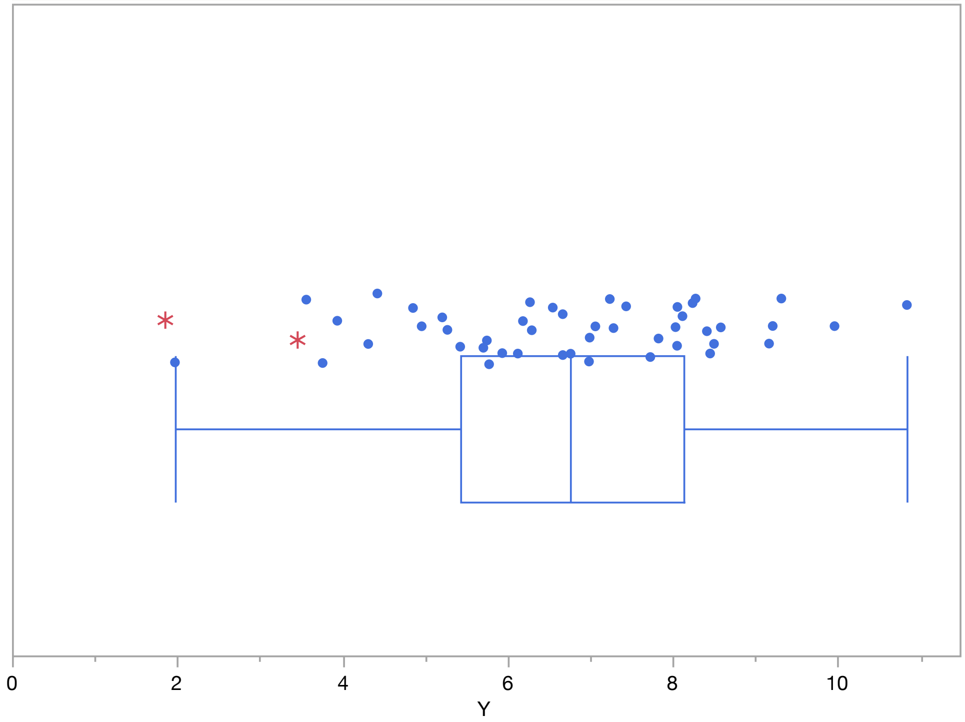


**Well = D09**

## Graph Builder

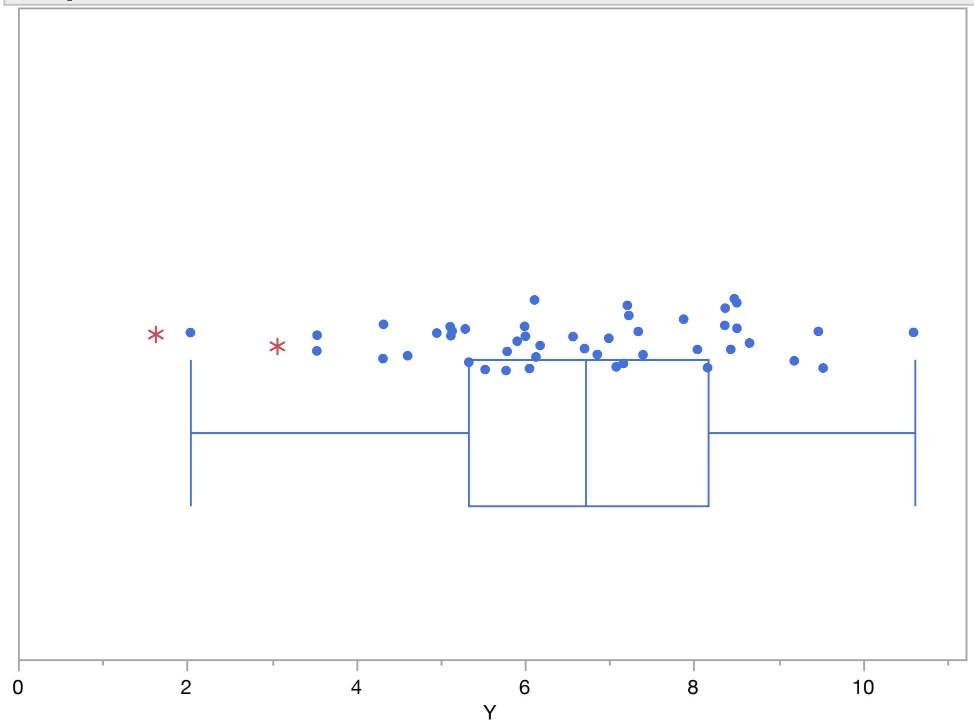


**Well = D10**

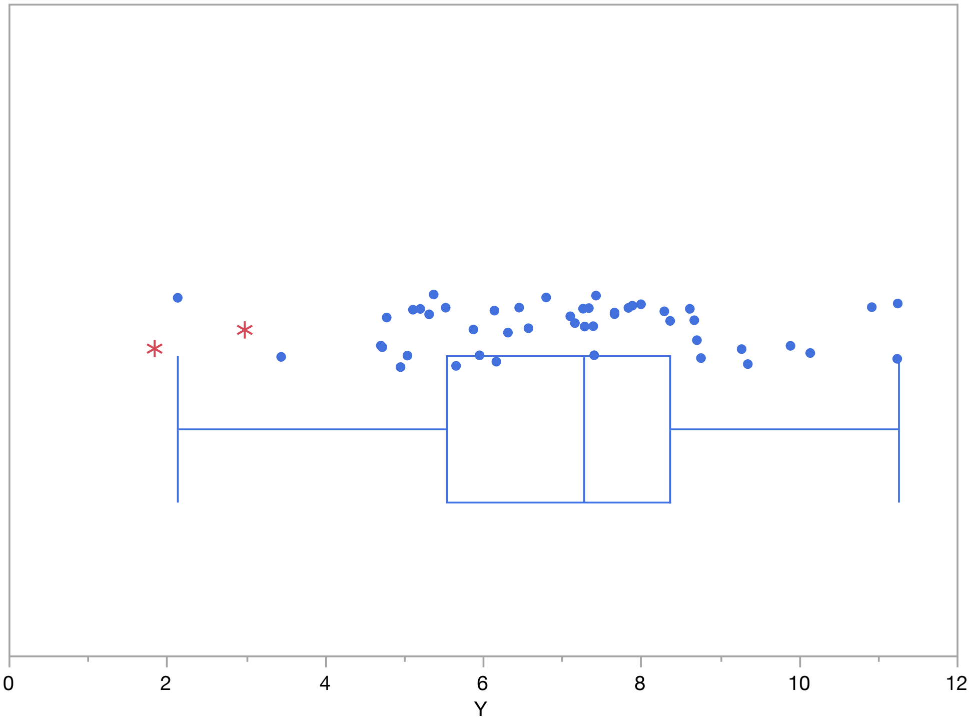


**Well = D11**

## Graph Builder

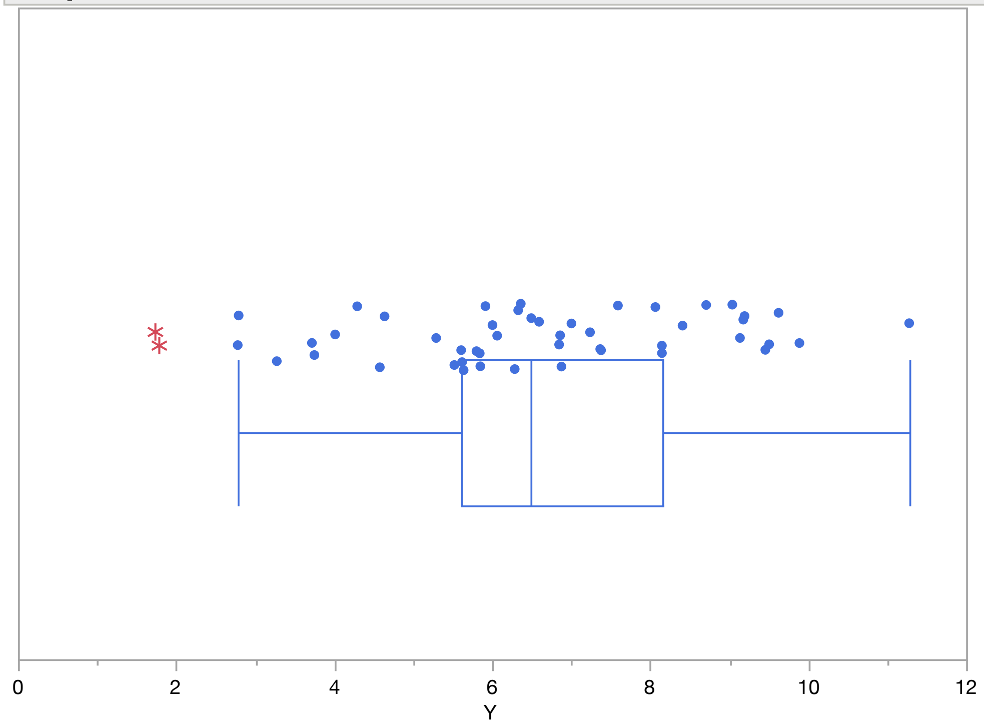


**Well = D12**

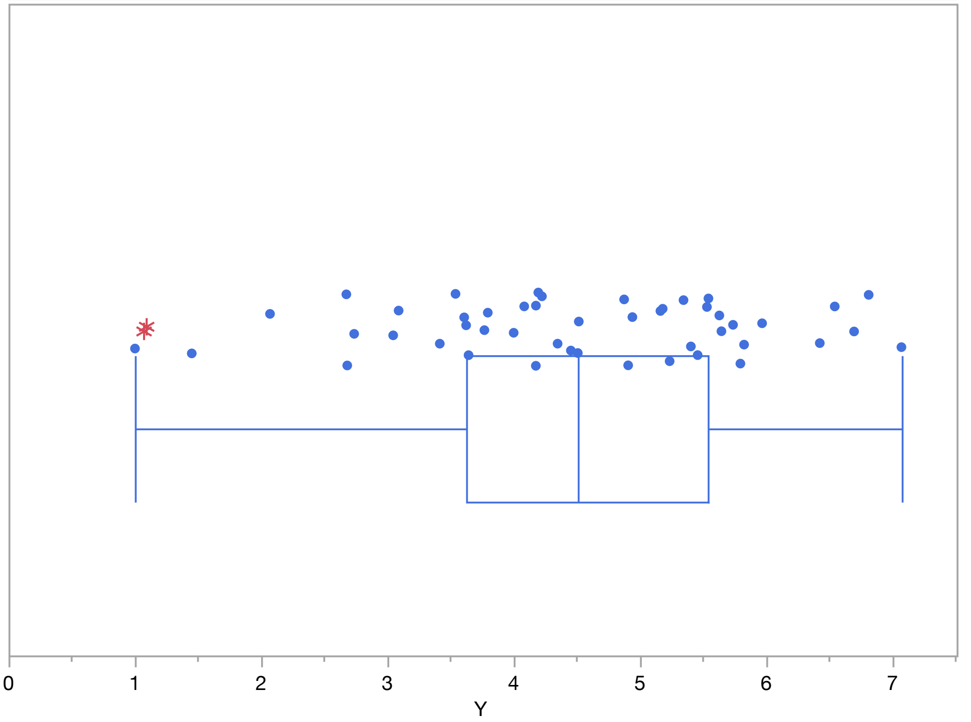


**Well = E01**

## Graph Builder

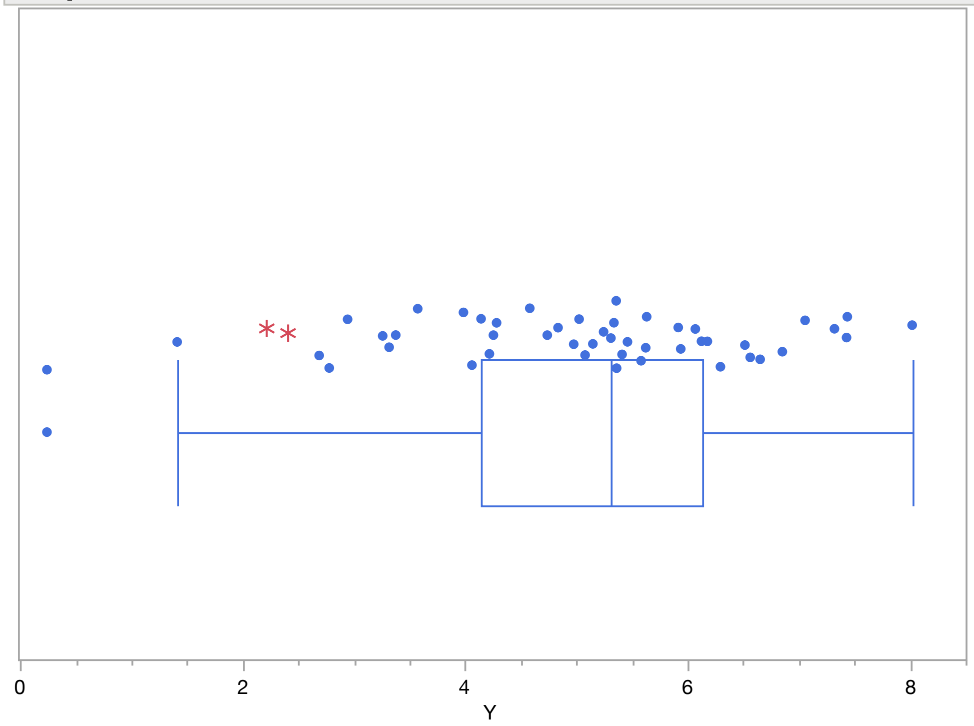


**Well = E02**

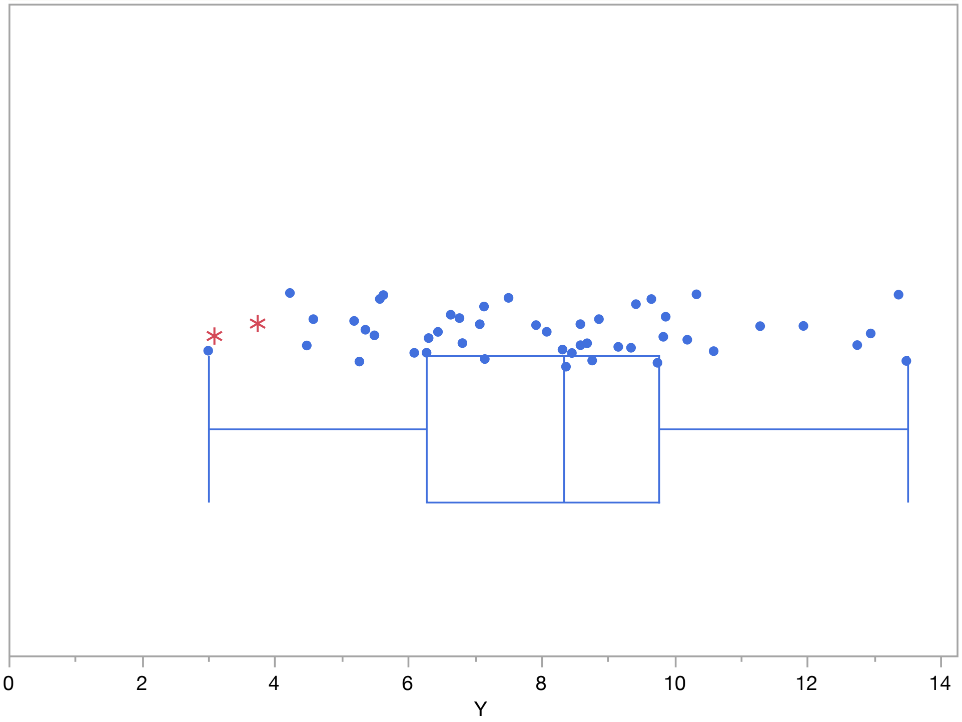


**Well = E03**

## Graph Builder

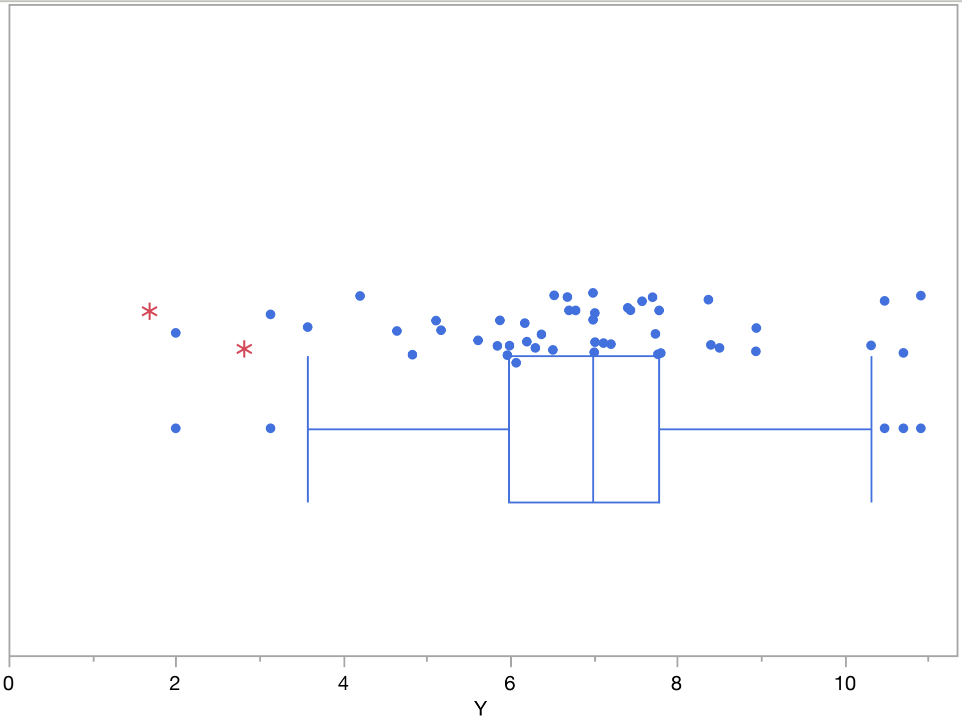


**Well = E04**

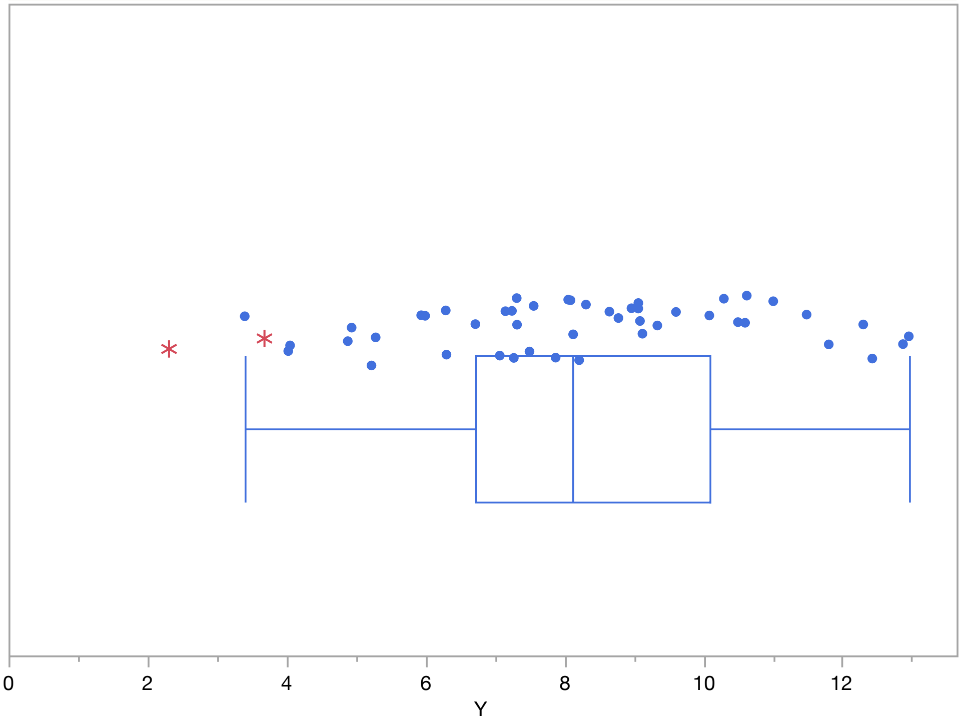


**Well = E05**

## Graph Builder

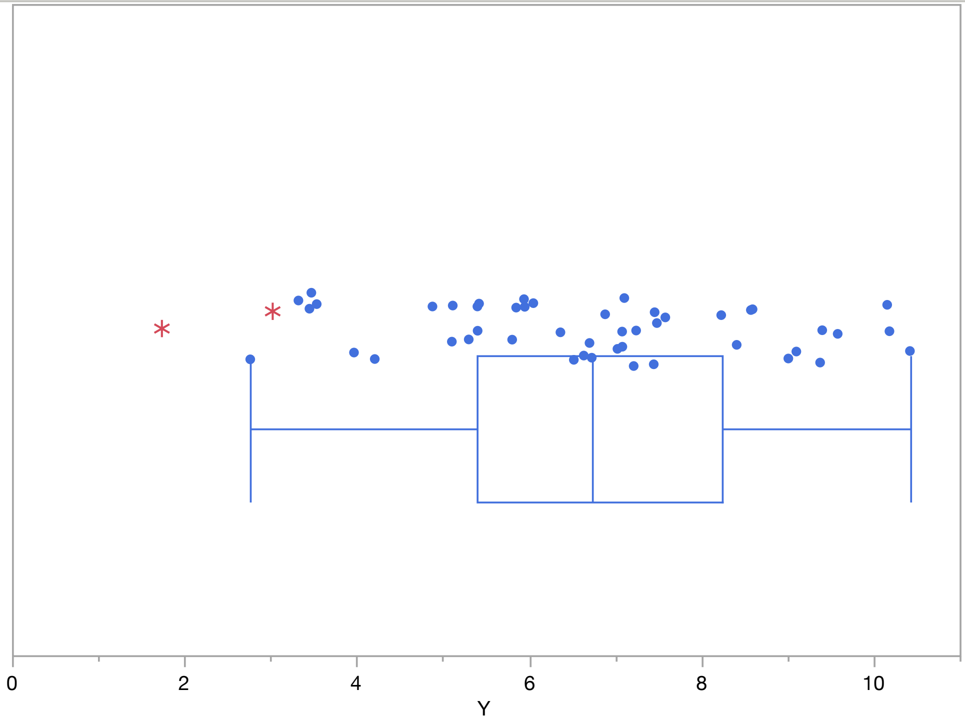


**Well = E06**

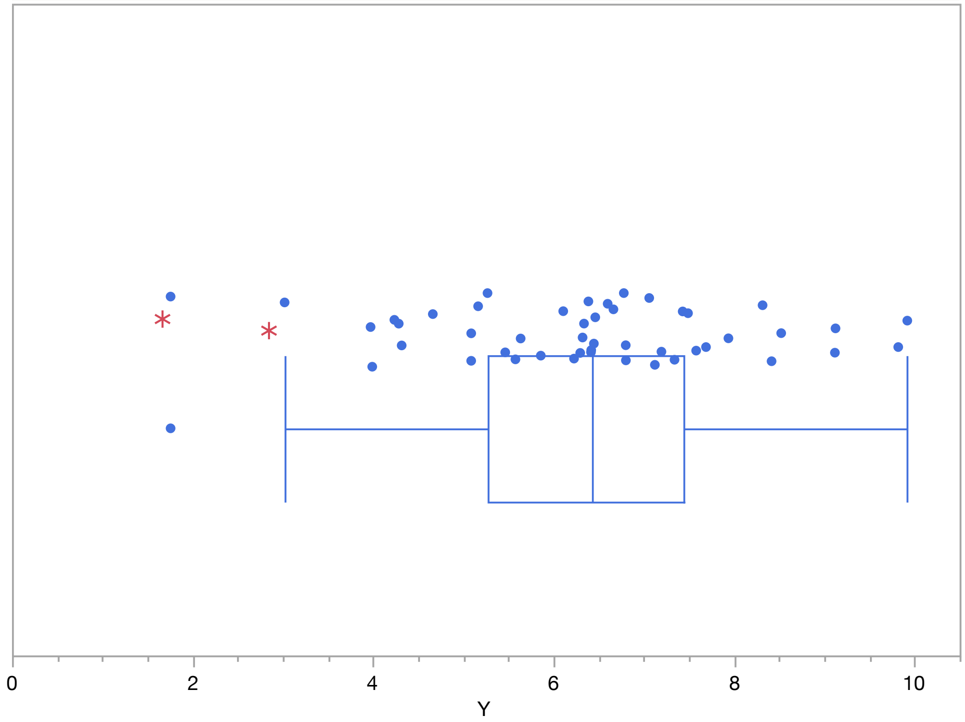


**Well = E07**

## Graph Builder

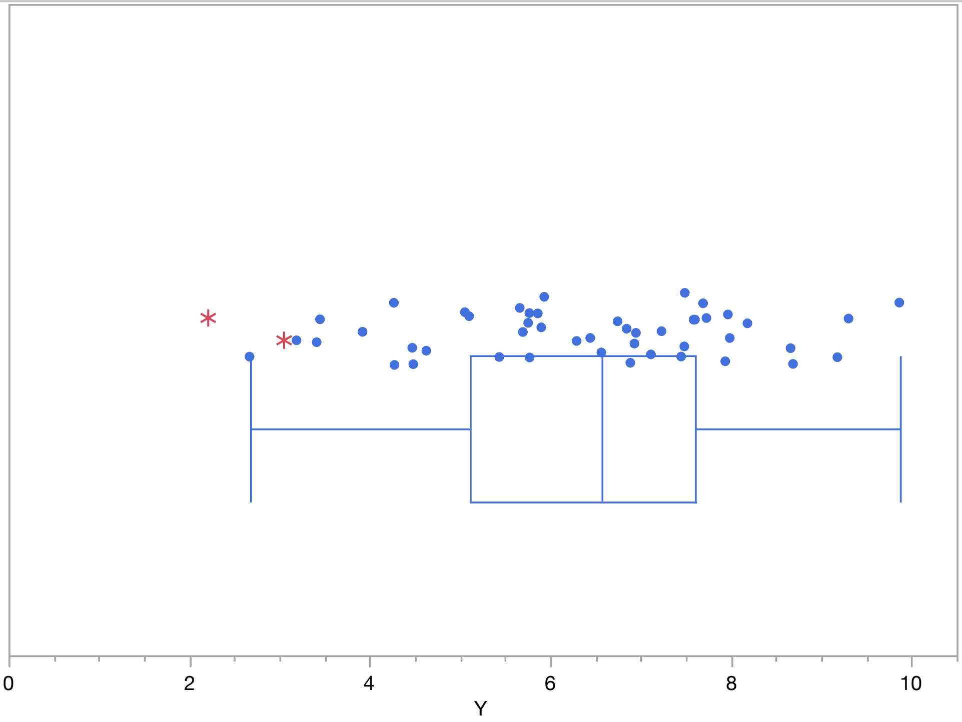


### Well = E08

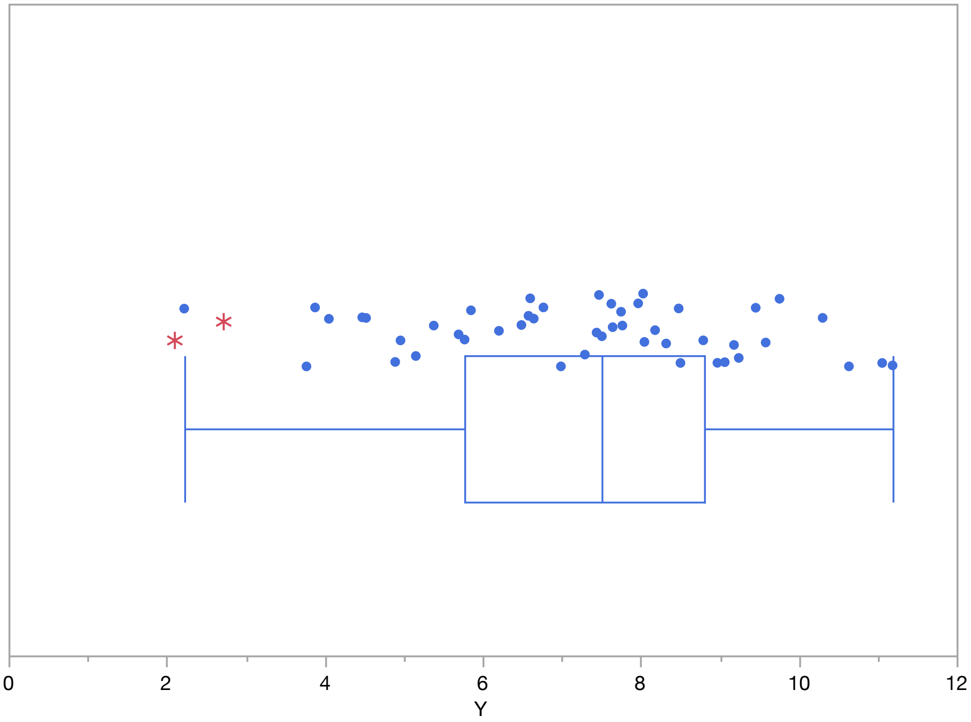


### Well = E09

## Graph Builder

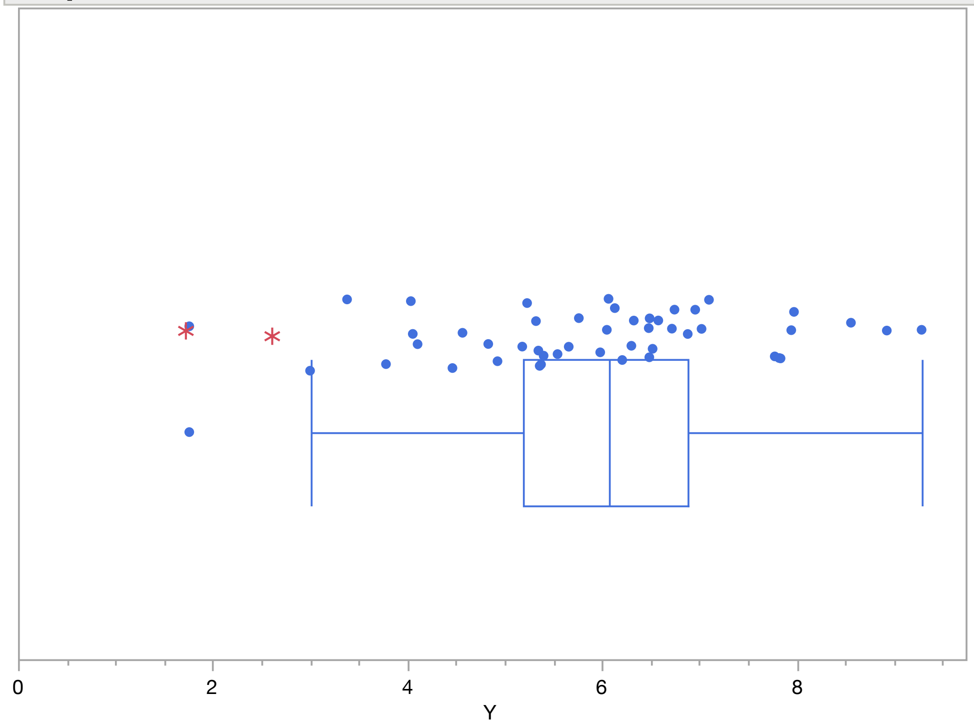


**Well = E10**

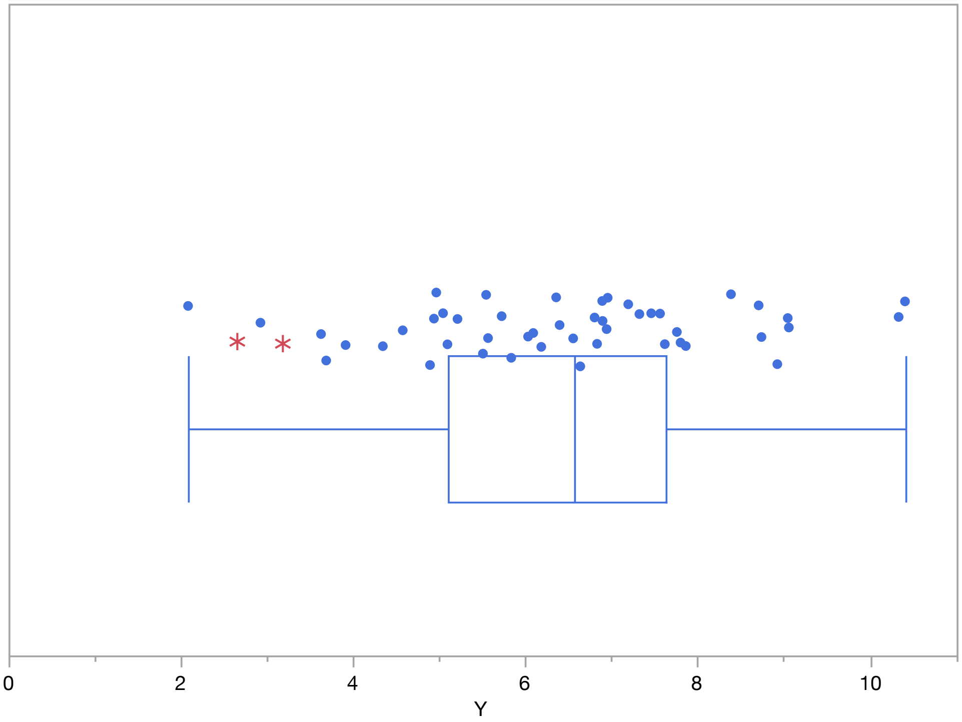


**Well = E11**

## Graph Builder

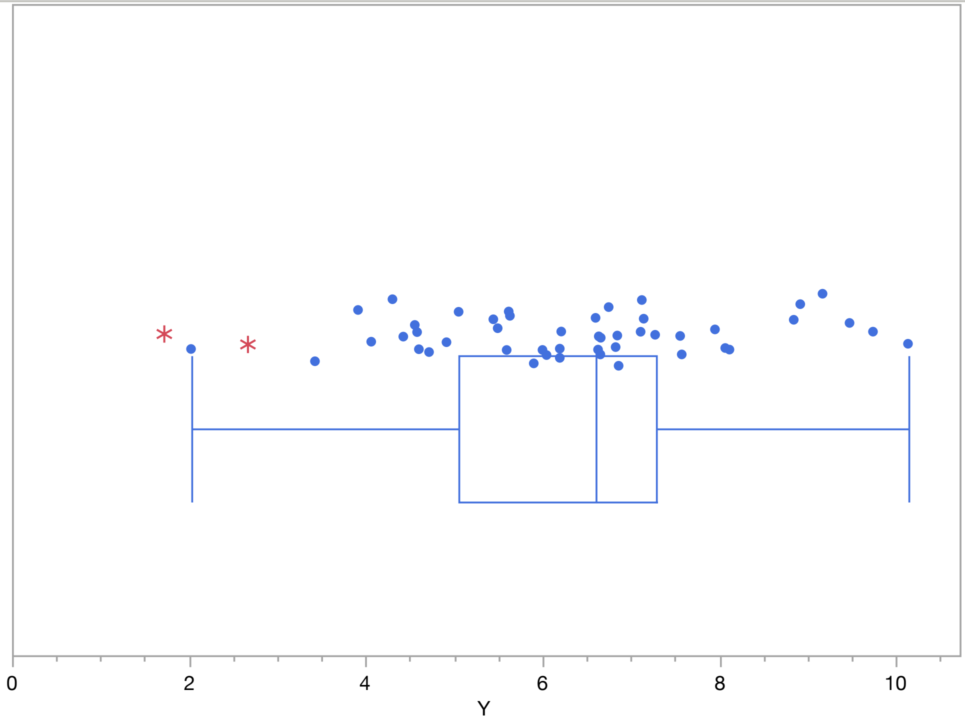


**Well = E12**

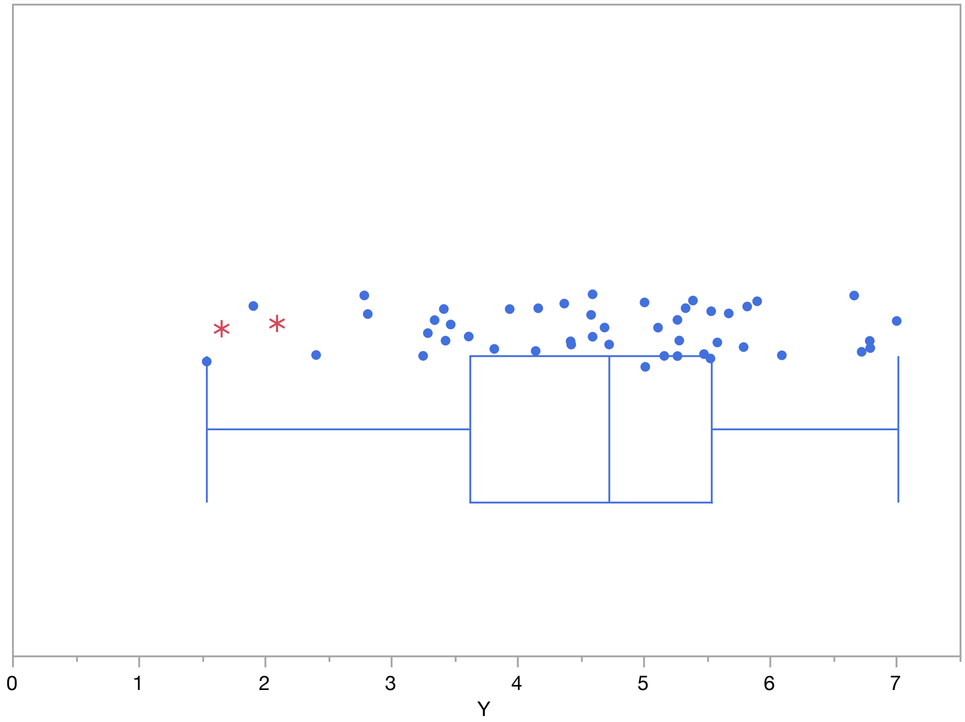


**Well = F01**

## Graph Builder

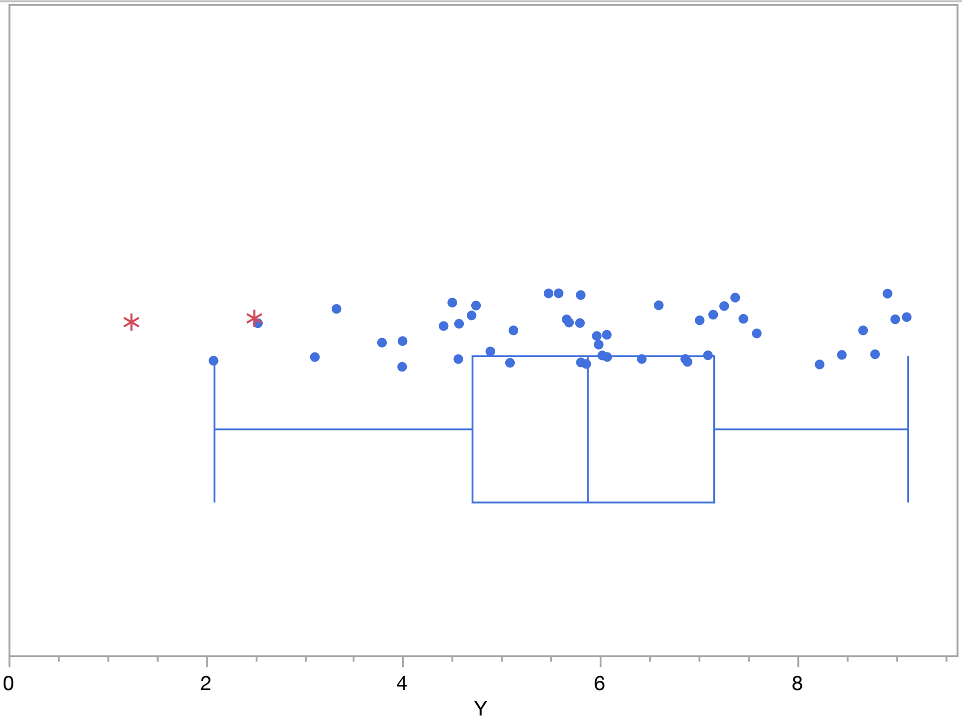


**Well = F02**

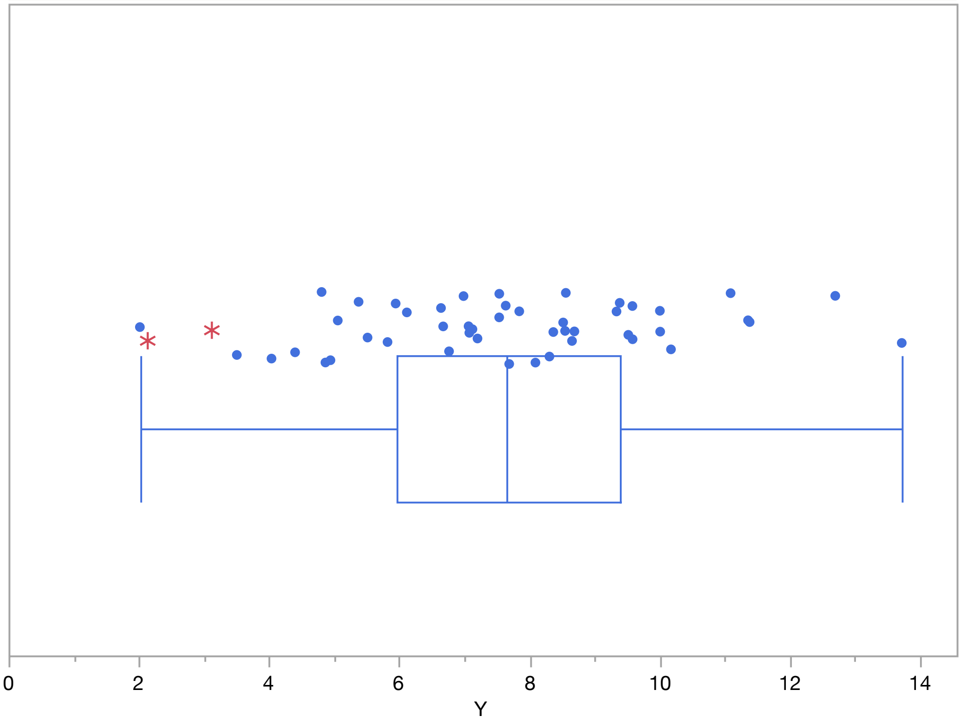


**Well = F03**

## Graph Builder

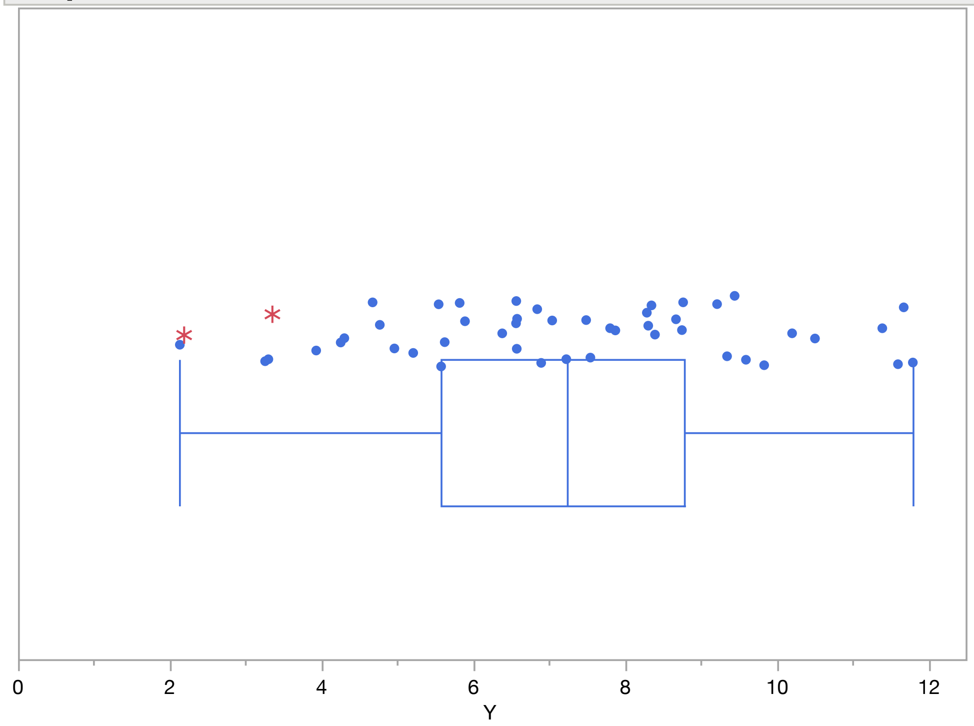


**Well = F04**

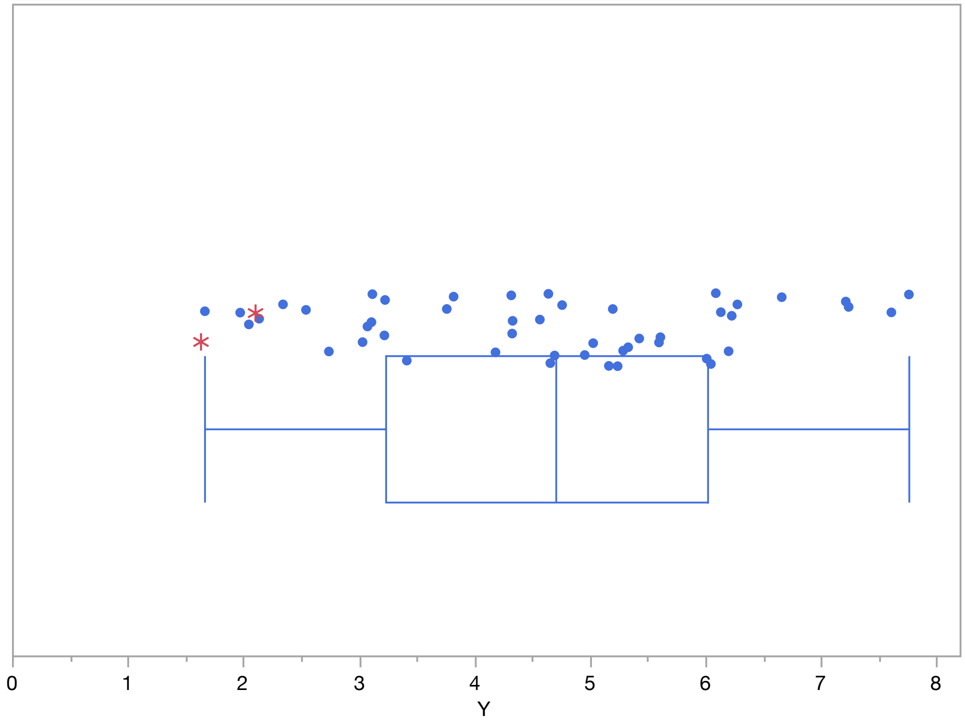


**Well = F05**

## Graph Builder

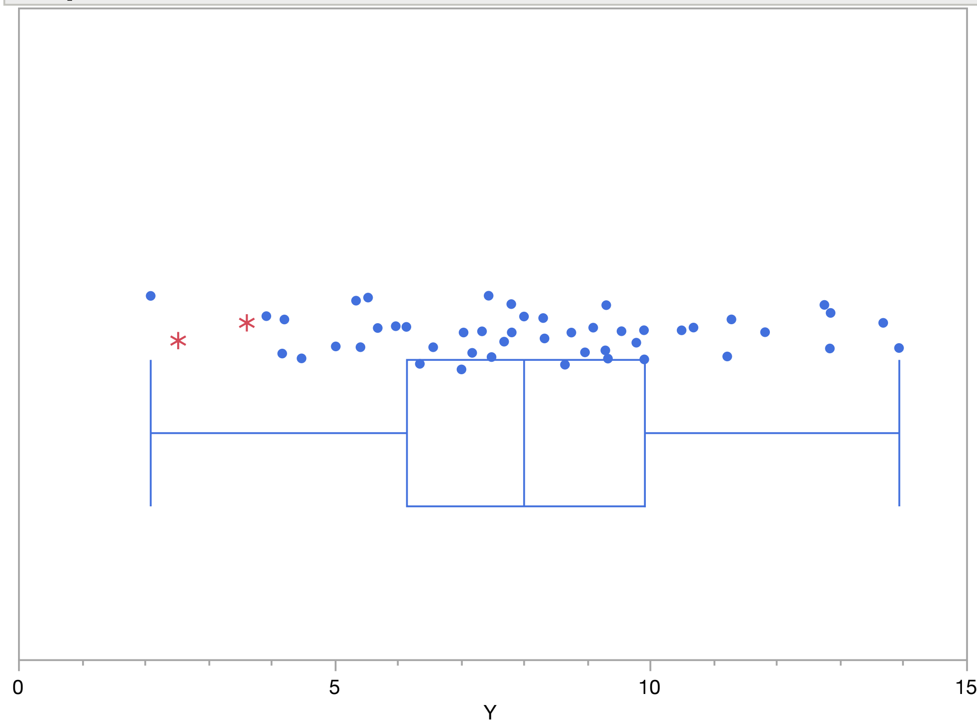


**Well = F06**

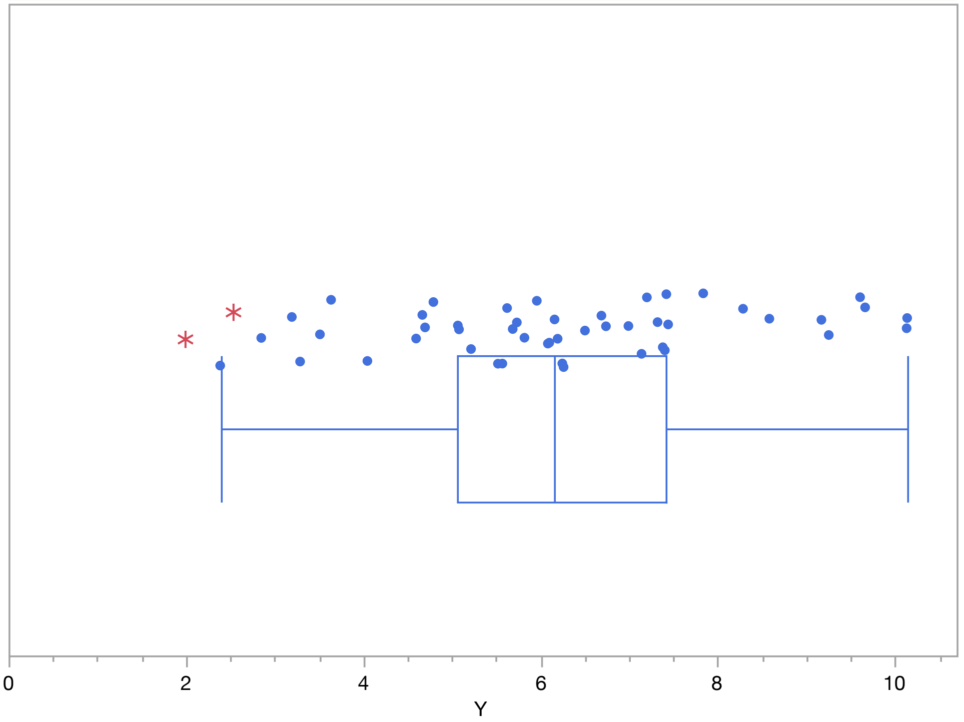


**Well = F07**

## Graph Builder

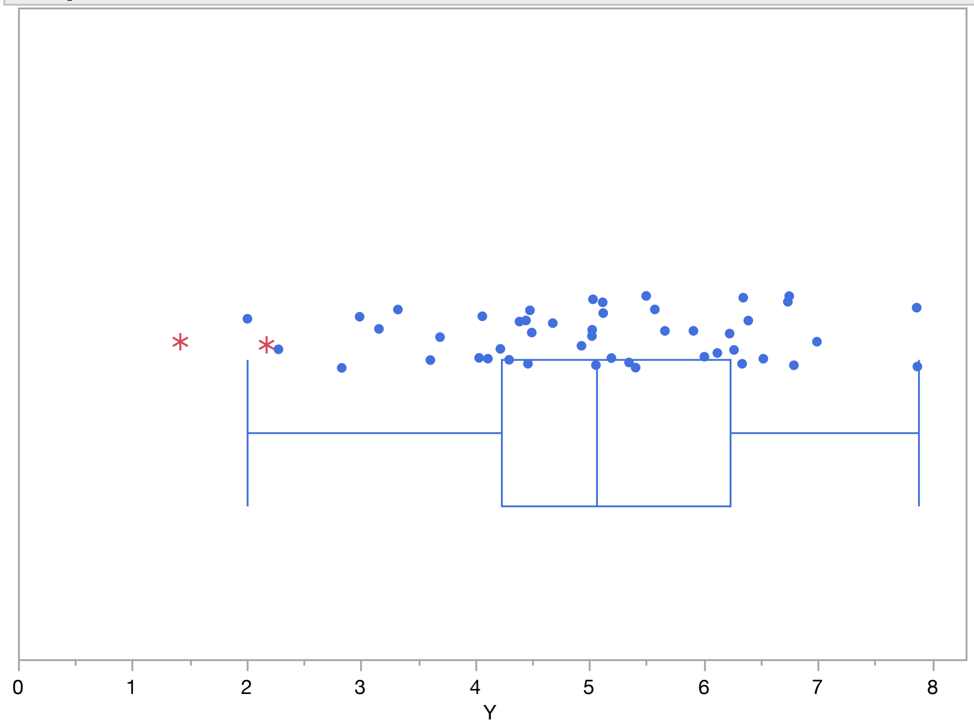


### Well = F08

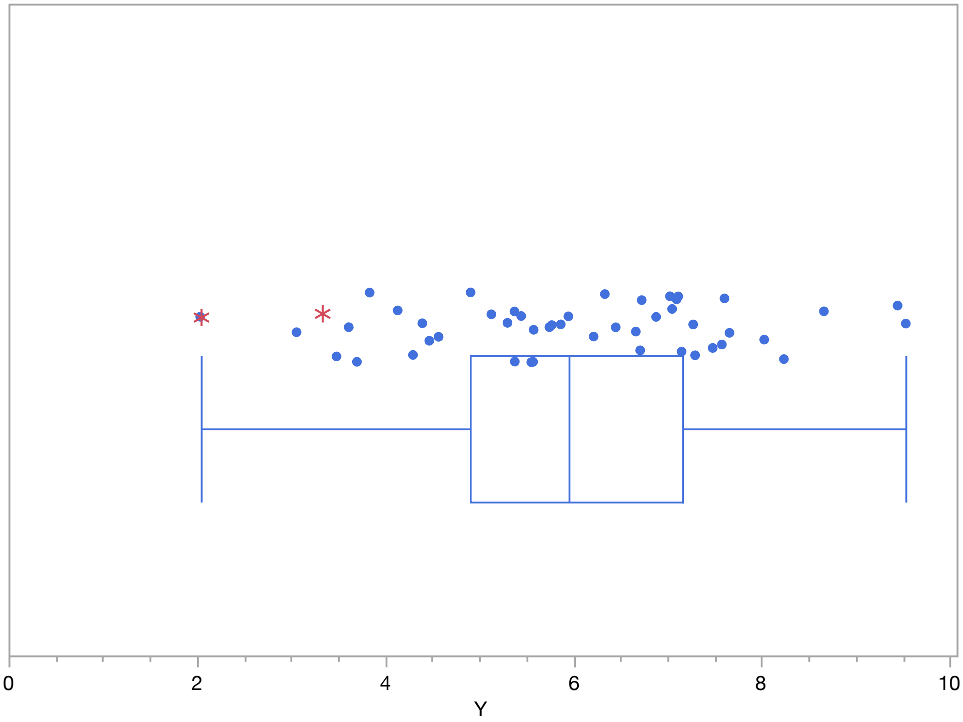


### Well = F09

## Graph Builder

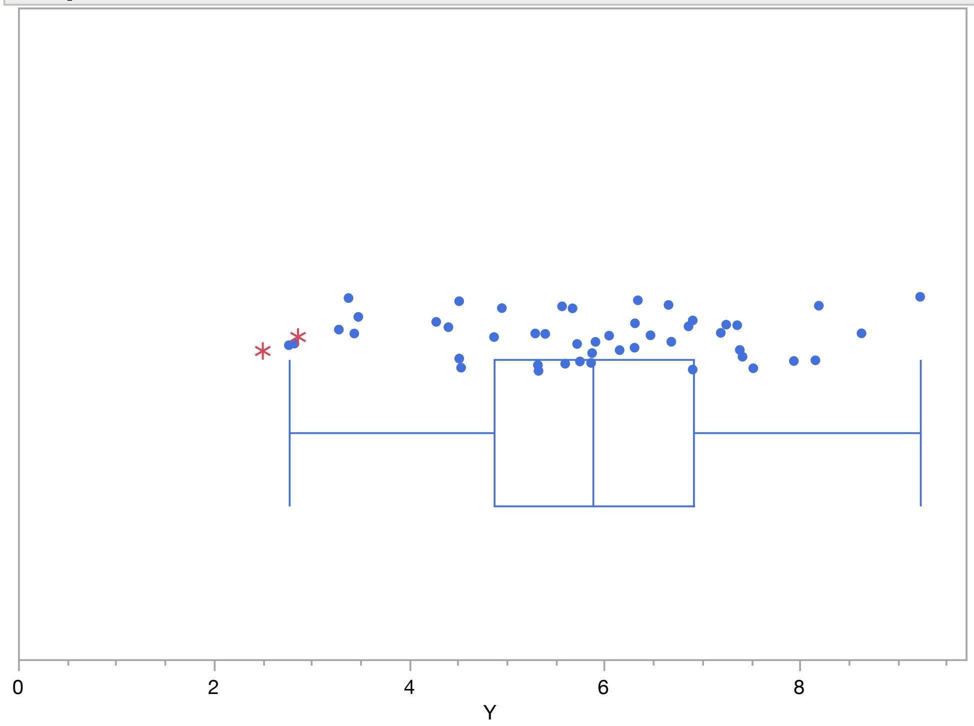


**Well = F10**

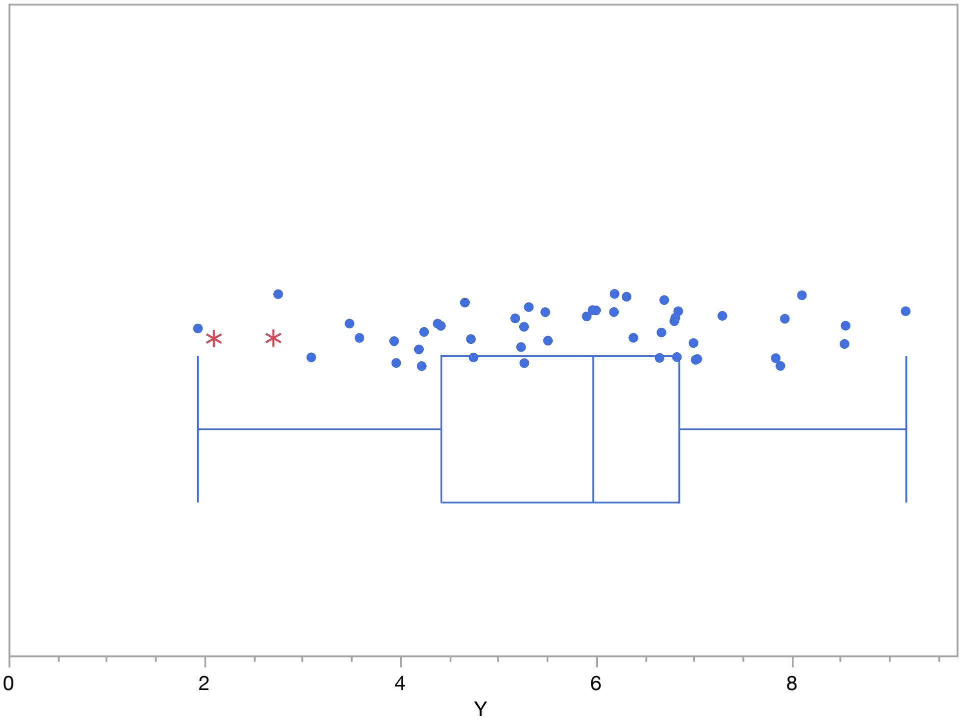


**Well = F11**

## Graph Builder

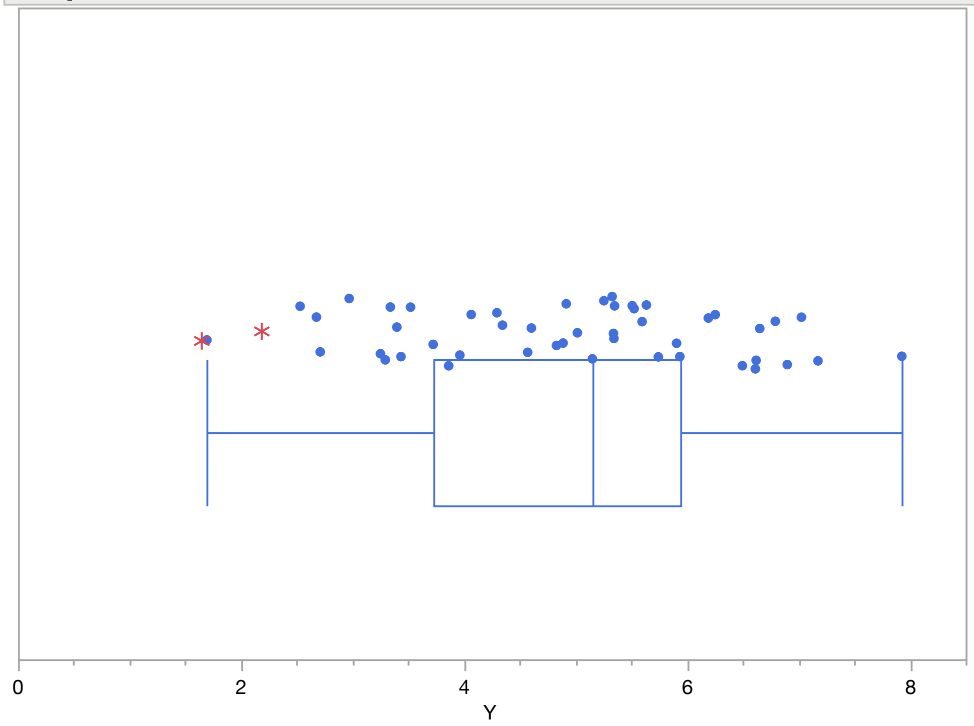


**Well = F12**

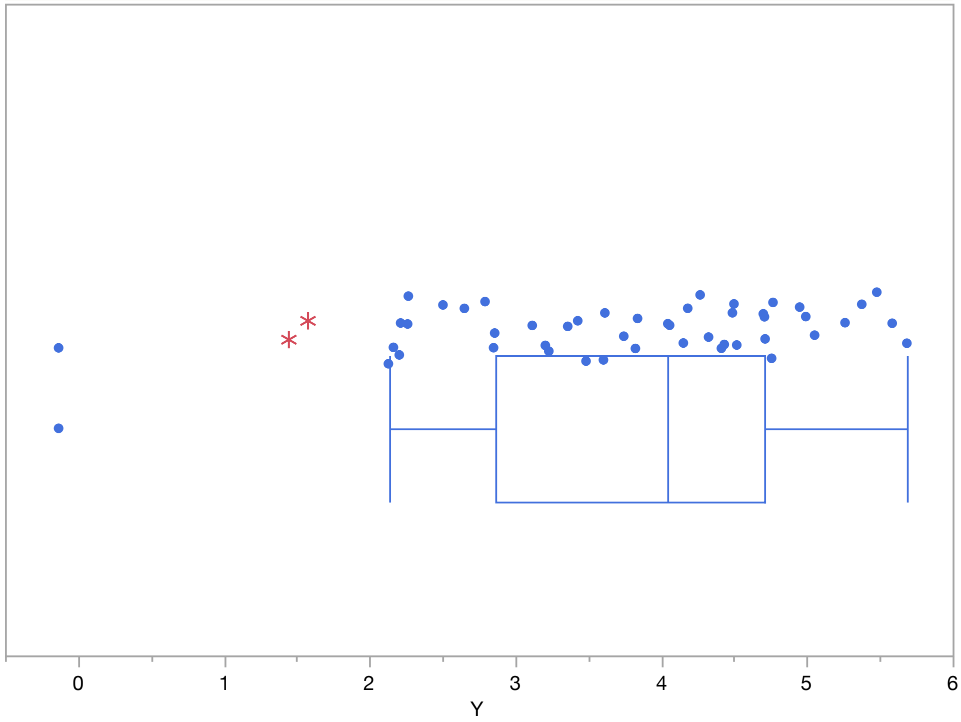


**Well = G01**

## Graph Builder

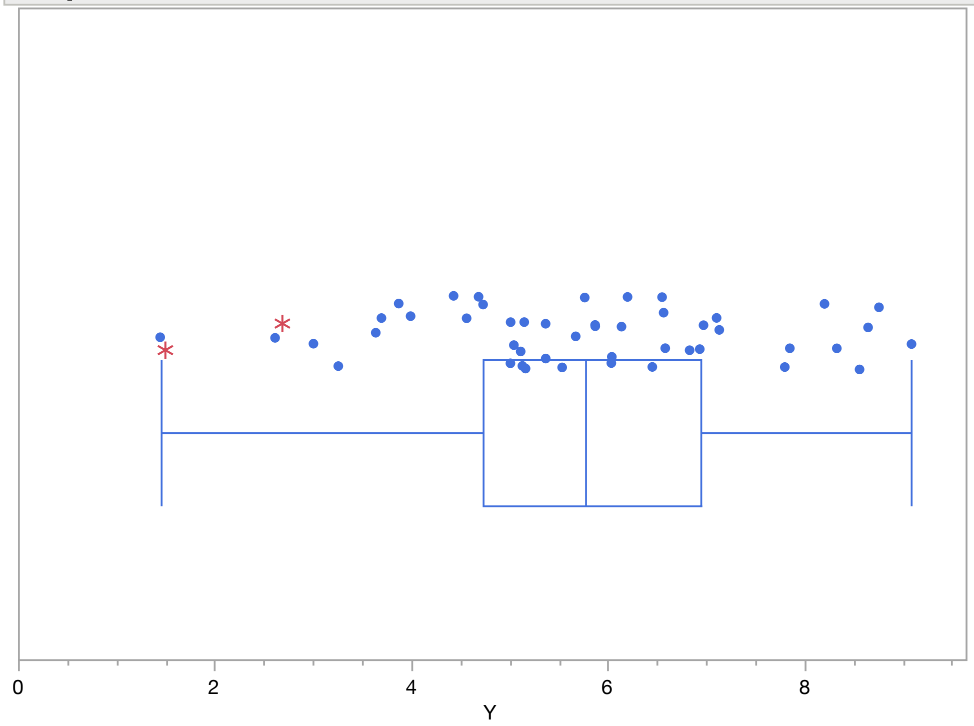


**Well = G02**

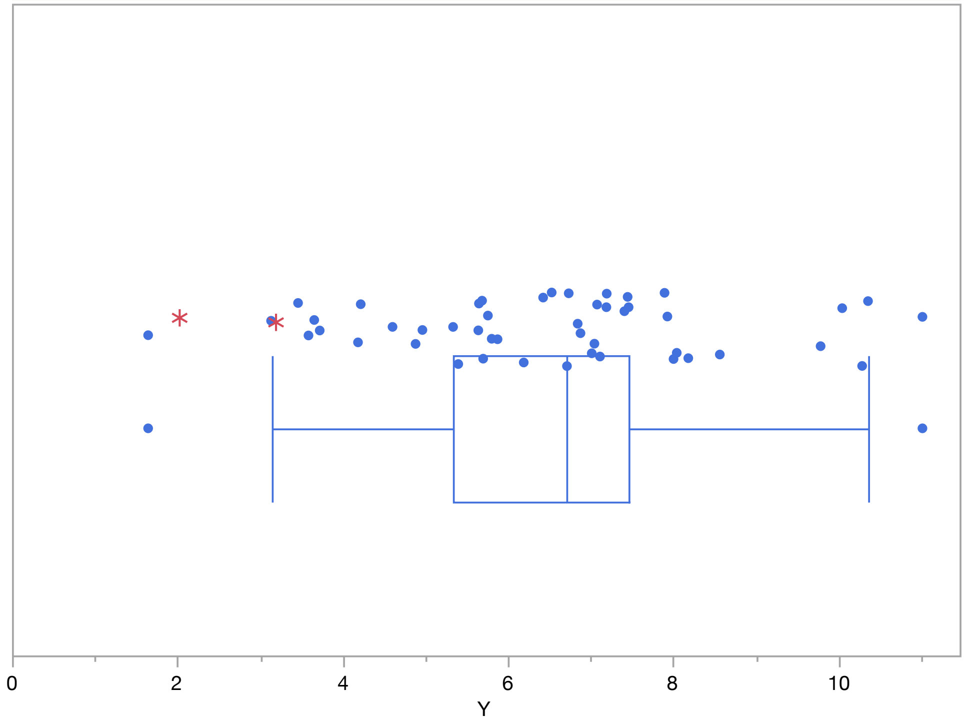


**Well = G03**

## Graph Builder

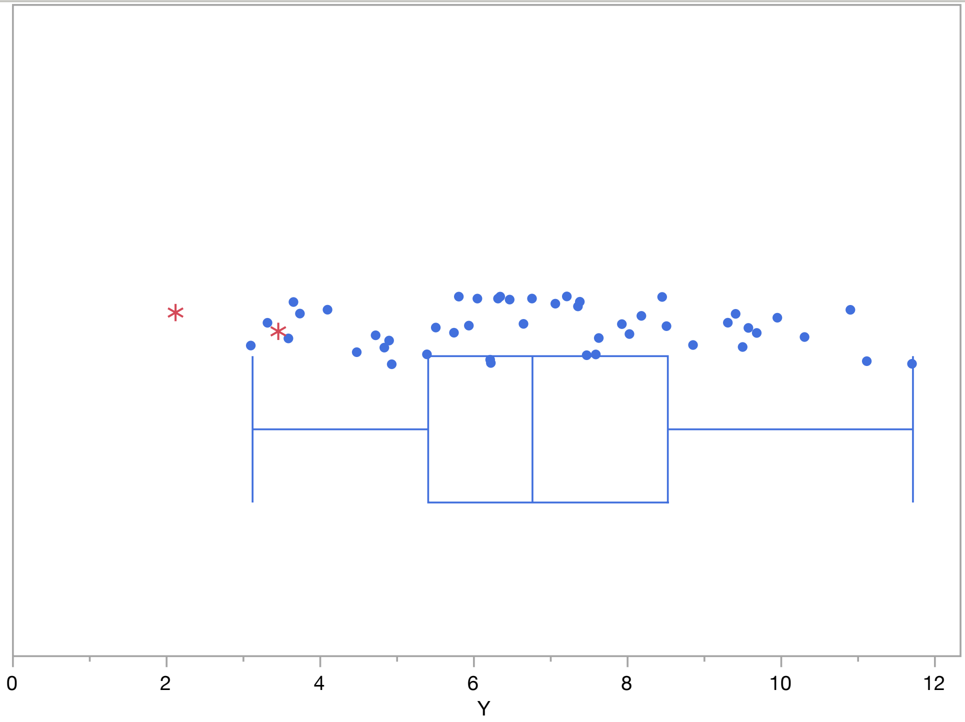


**Well = G04**

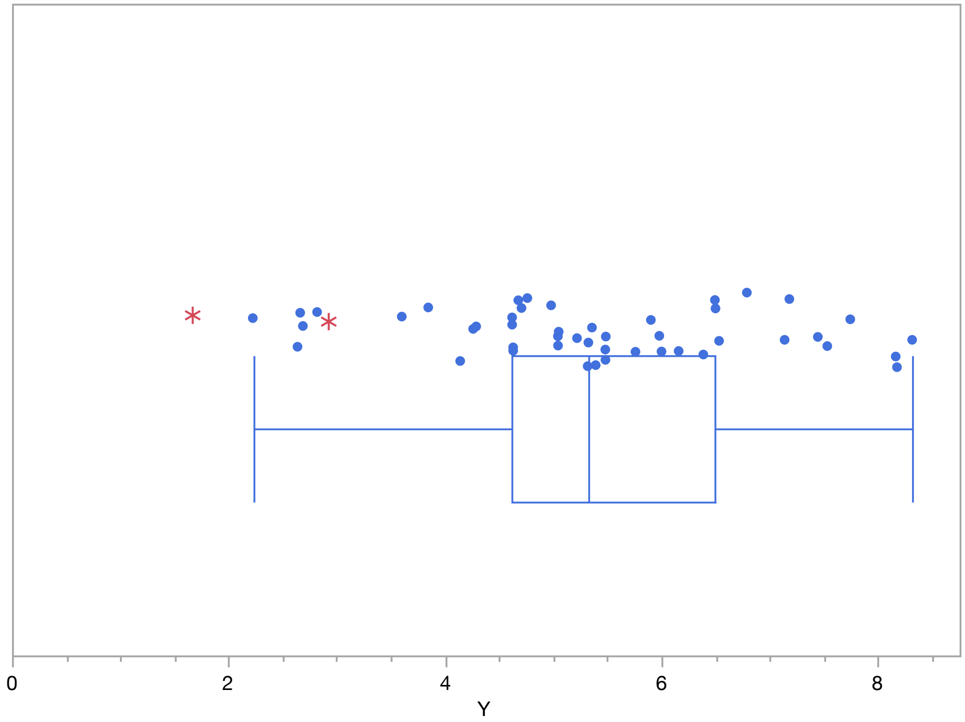


**Well = G05**

## Graph Builder

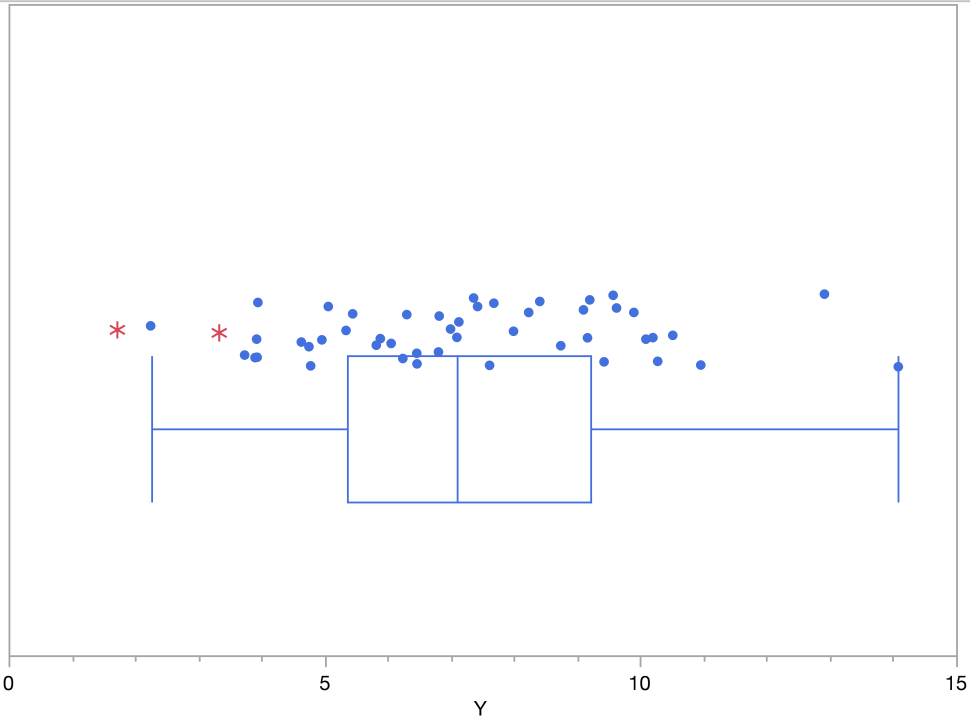


**Well = G06**

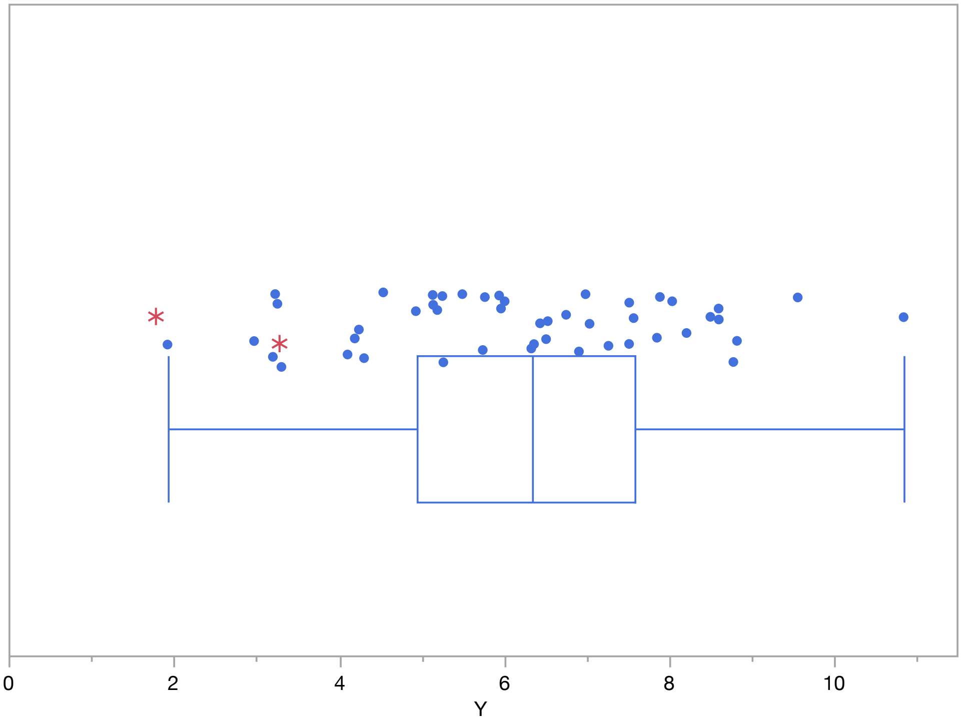


**Well = G07**

## Graph Builder

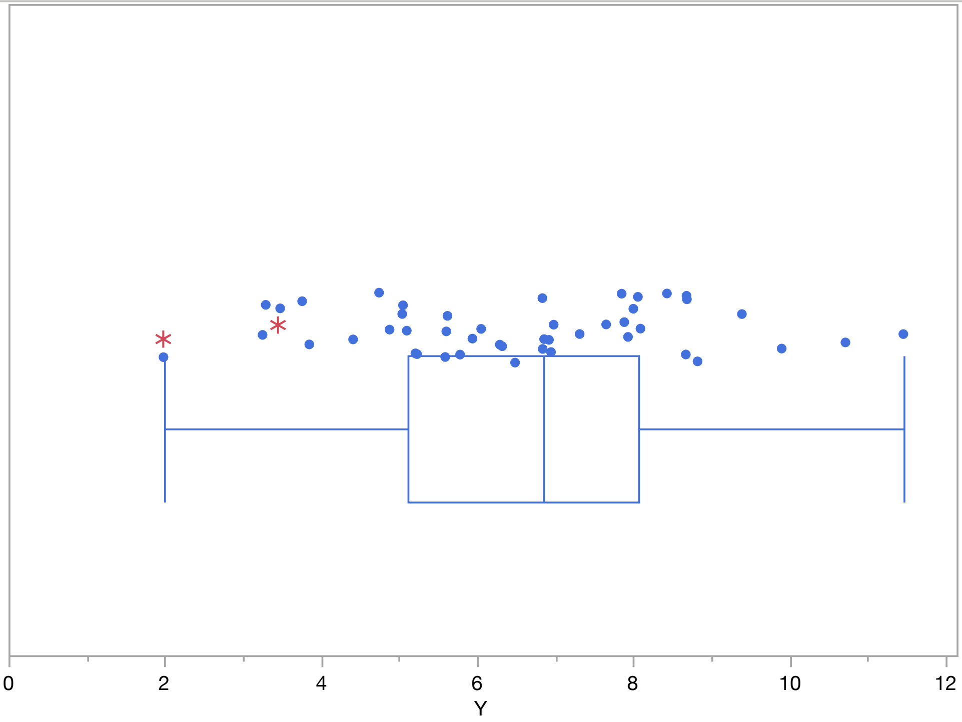


**Well = G08**

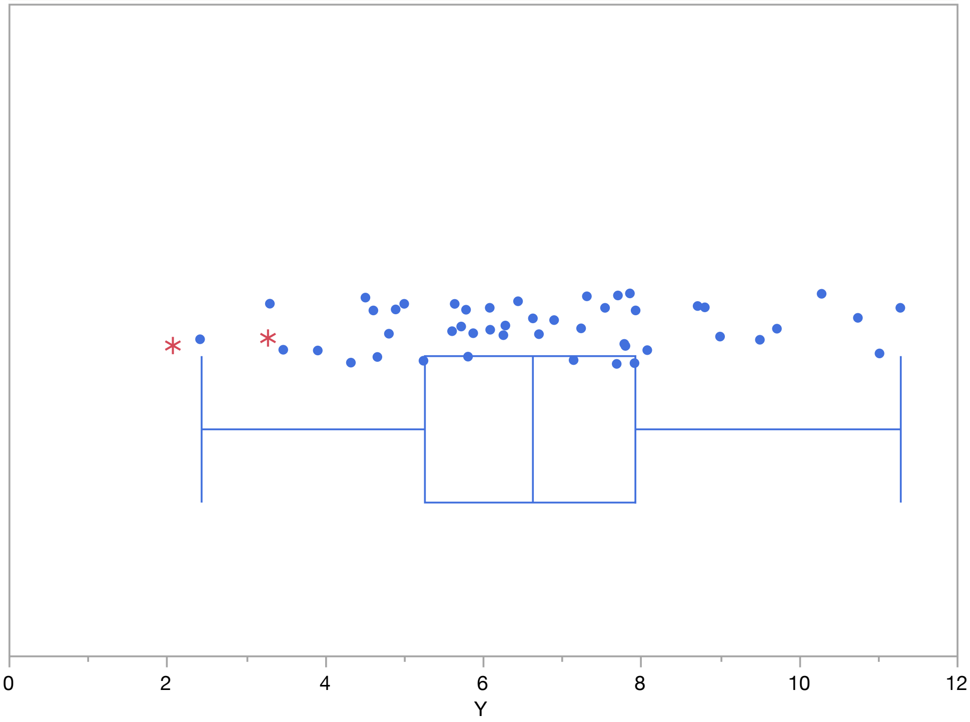


**Well = G09**

## Graph Builder

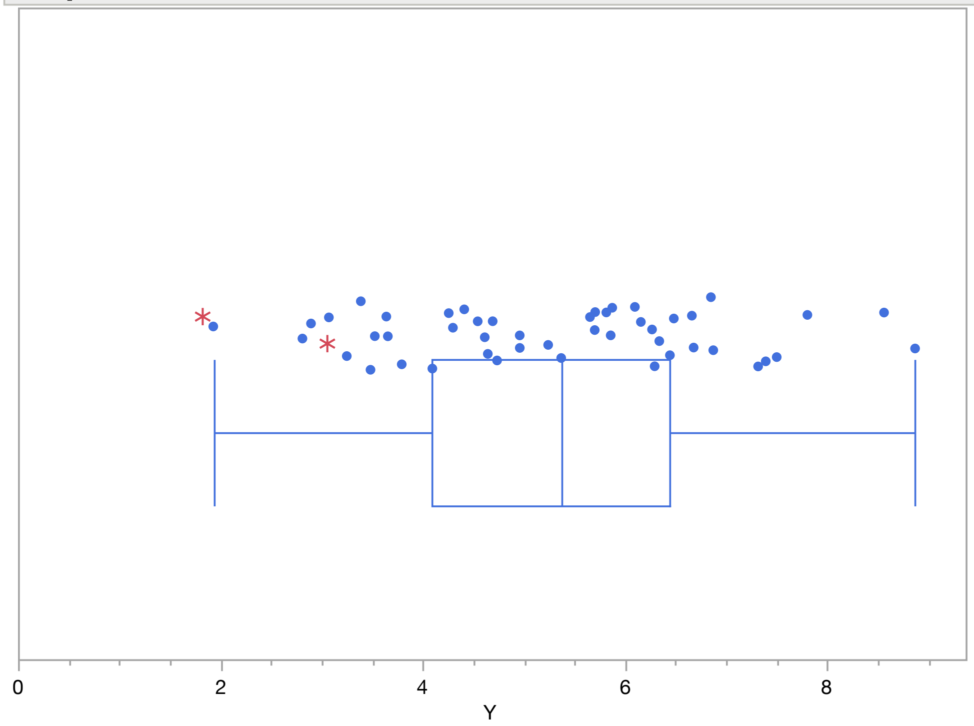


**Well = G10**

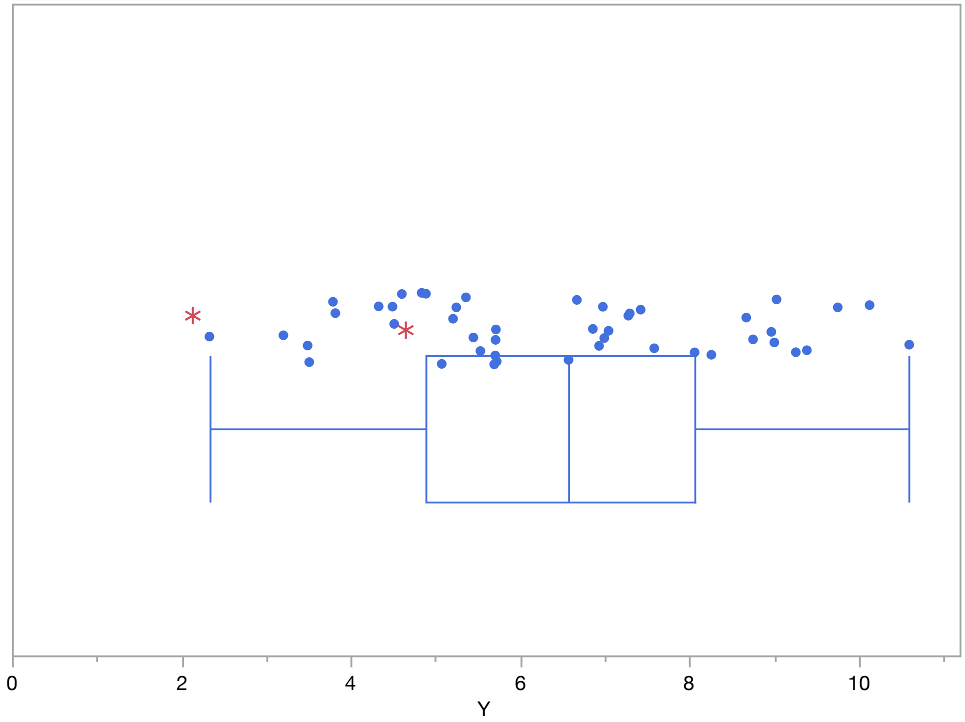


**Well = G11**

## Graph Builder

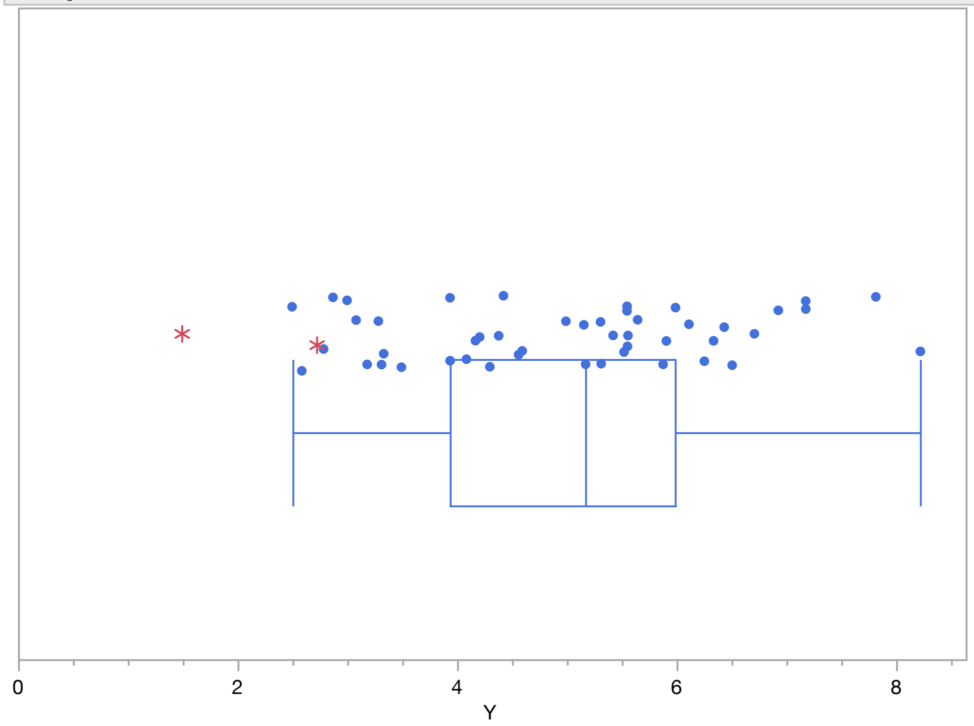


**Well = G12**

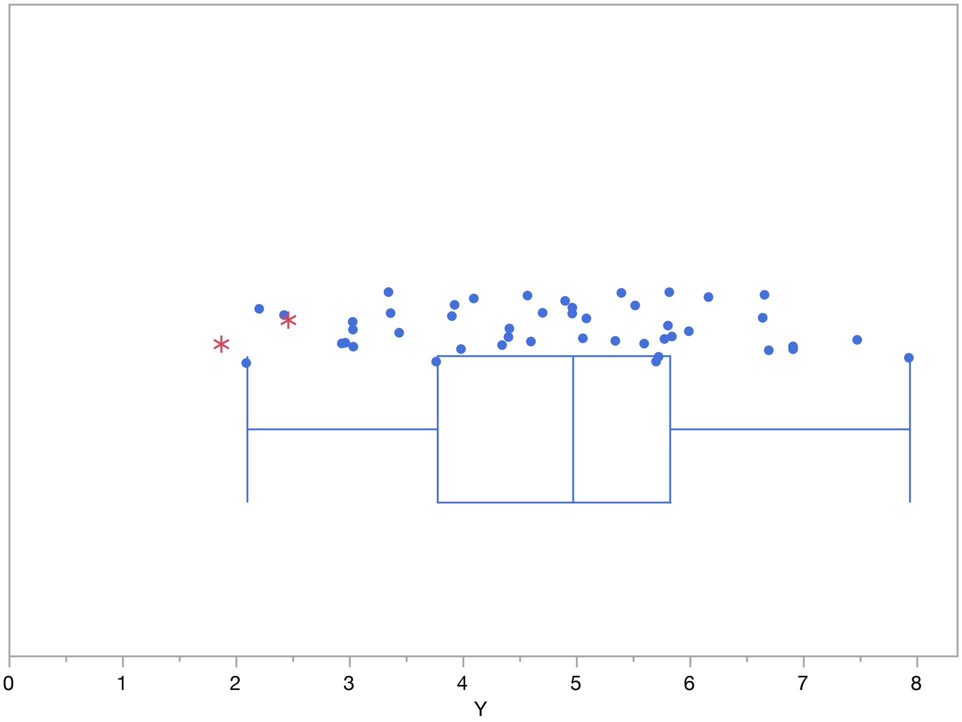


**Well = H01**

## Graph Builder

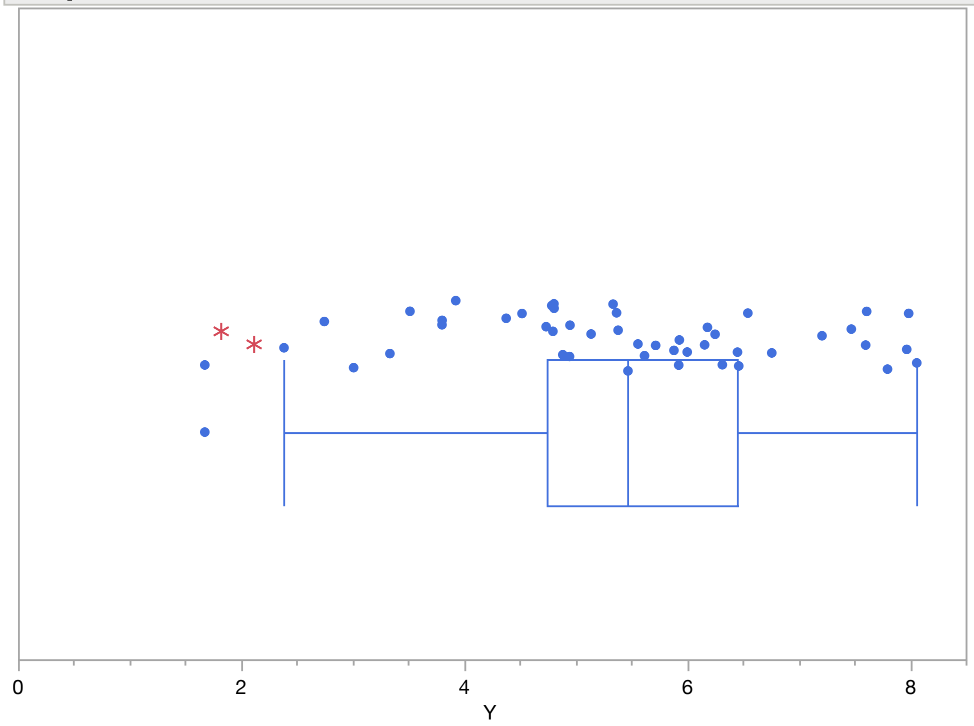


**Well = H02**

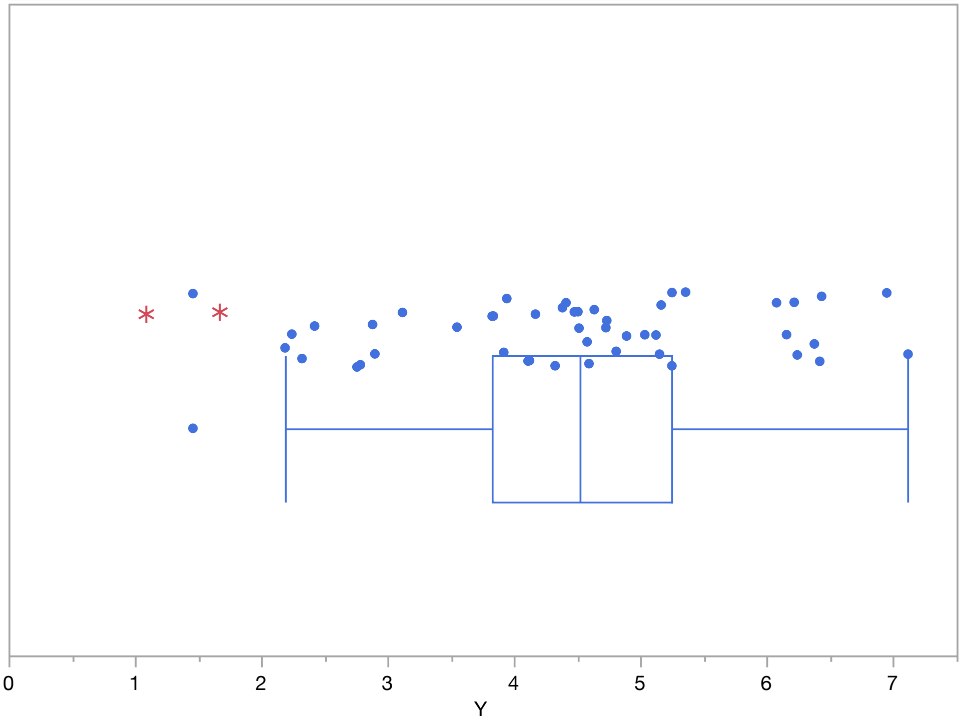


**Well = H03**

## Graph Builder

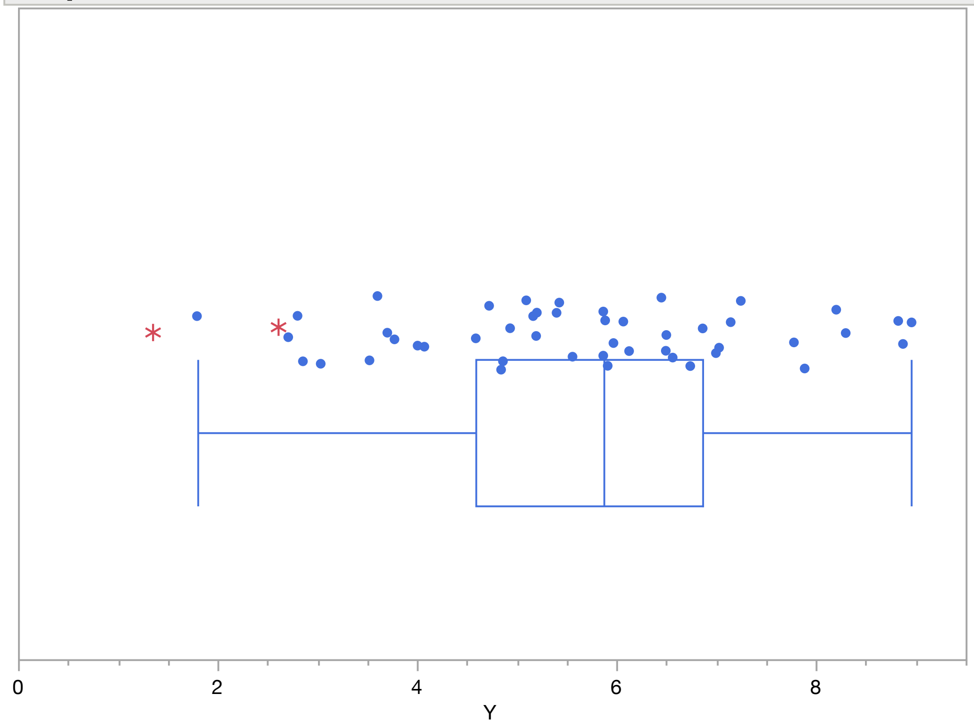


**Well = H04**

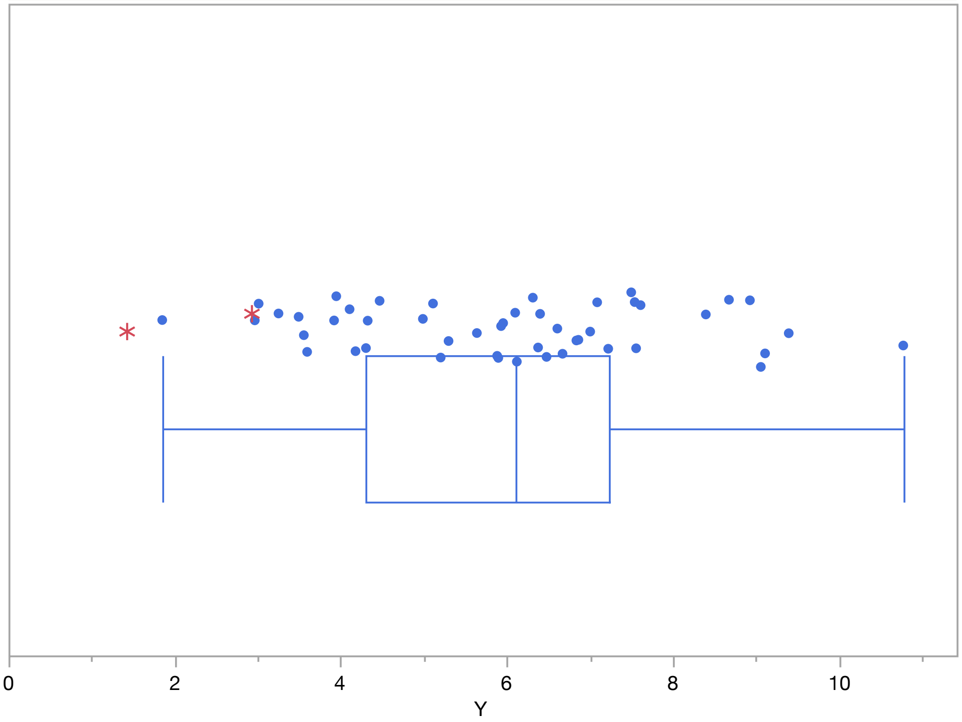


**Well = H05**

## Graph Builder

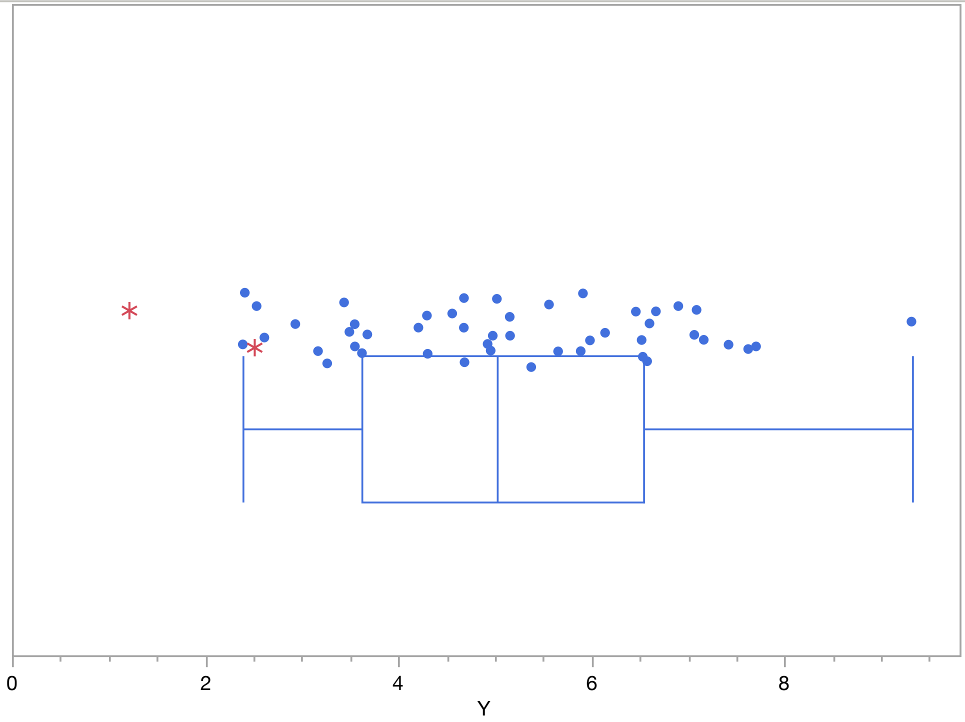


**Well = H06**

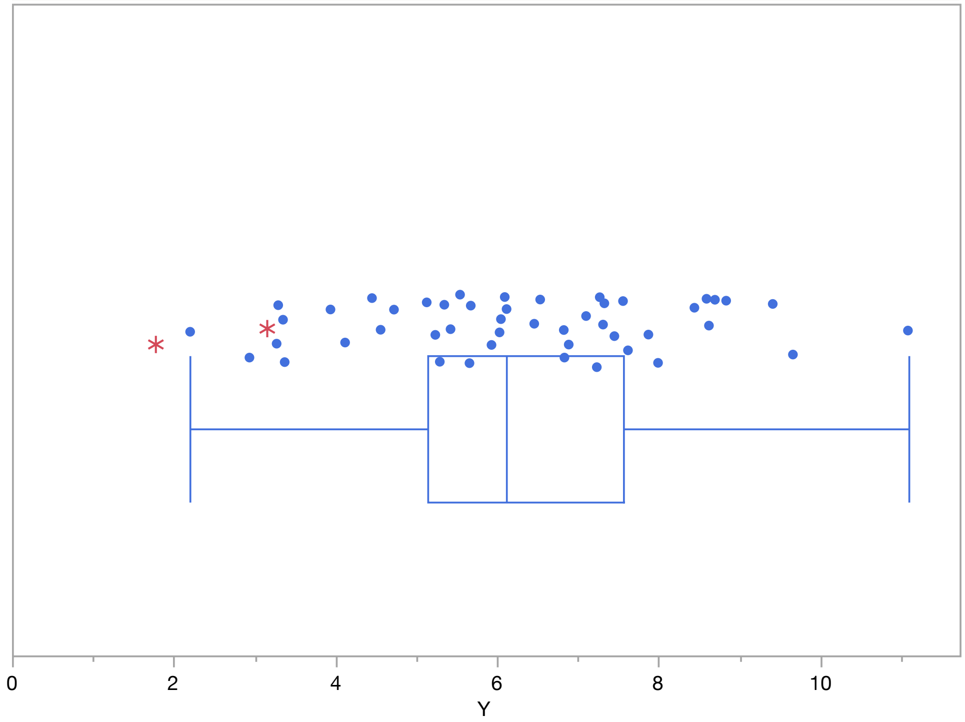


**Well = H07**

## Graph Builder

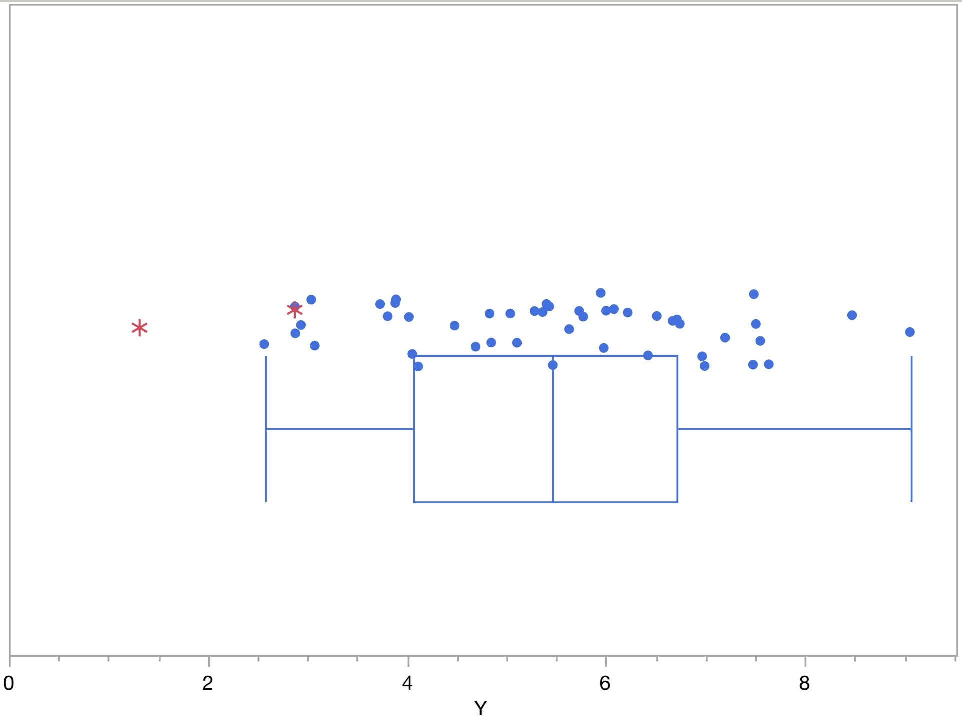


**Well = H08**

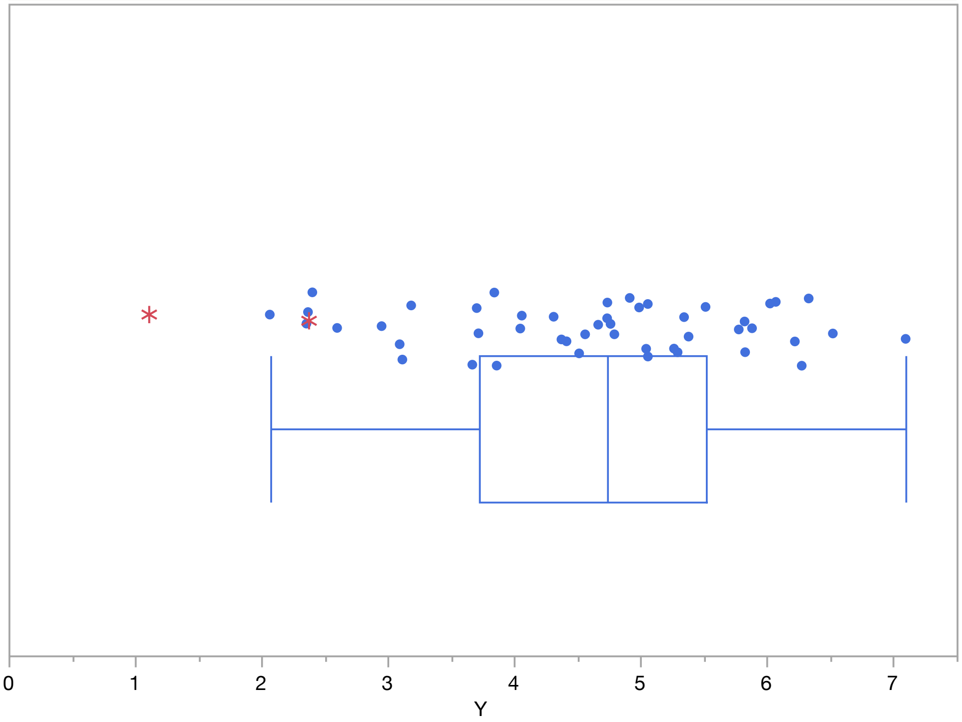


**Well = H09**

## Graph Builder

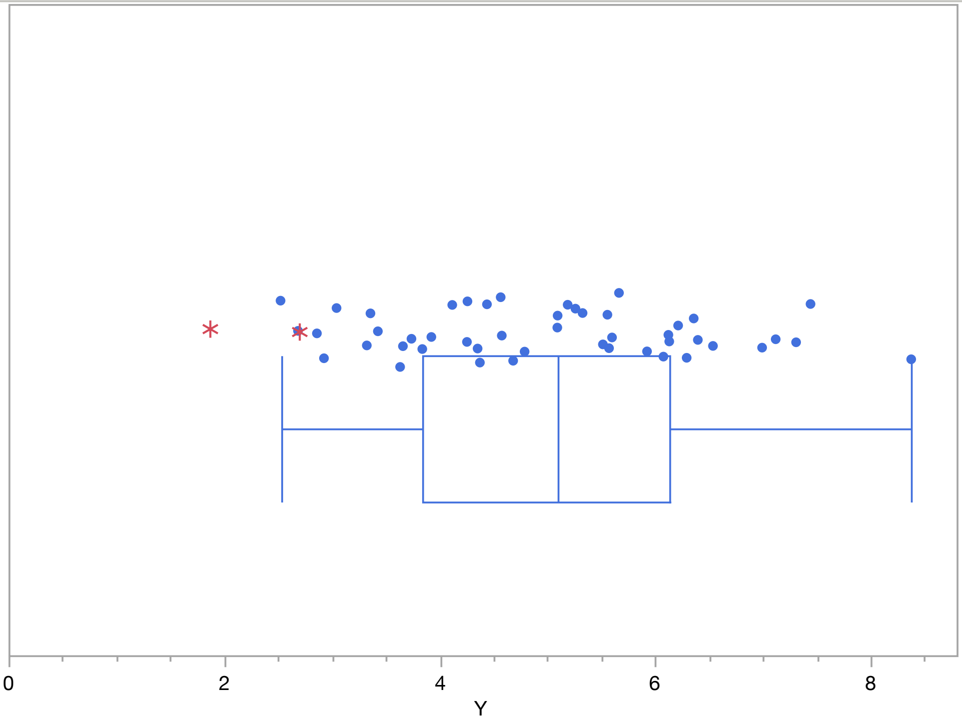


**Well = H10**

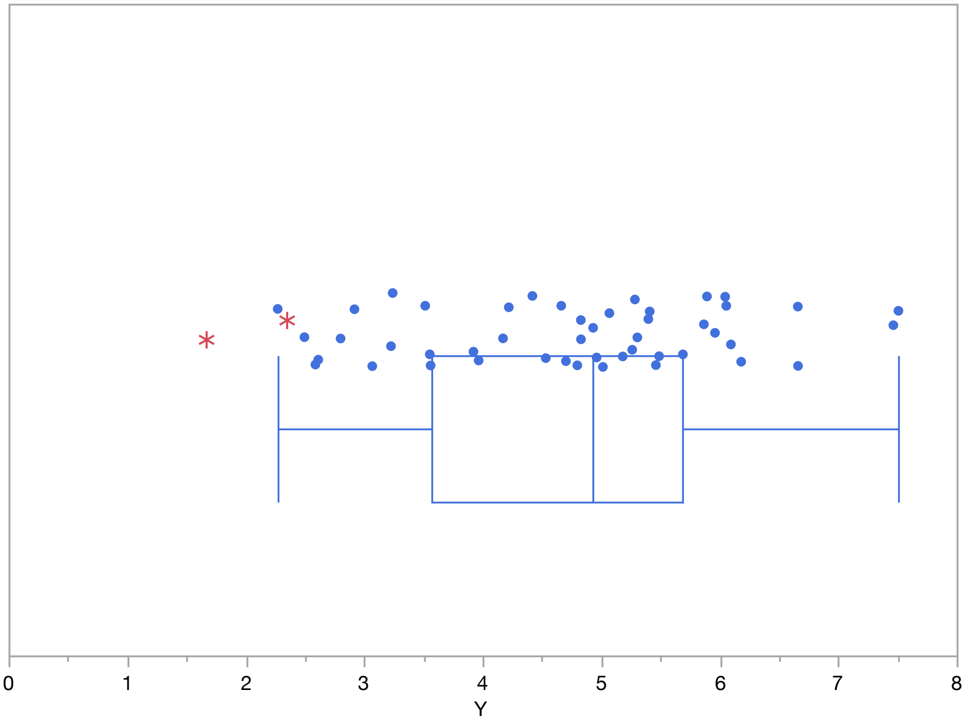


**Well = H11**

## Graph Builder

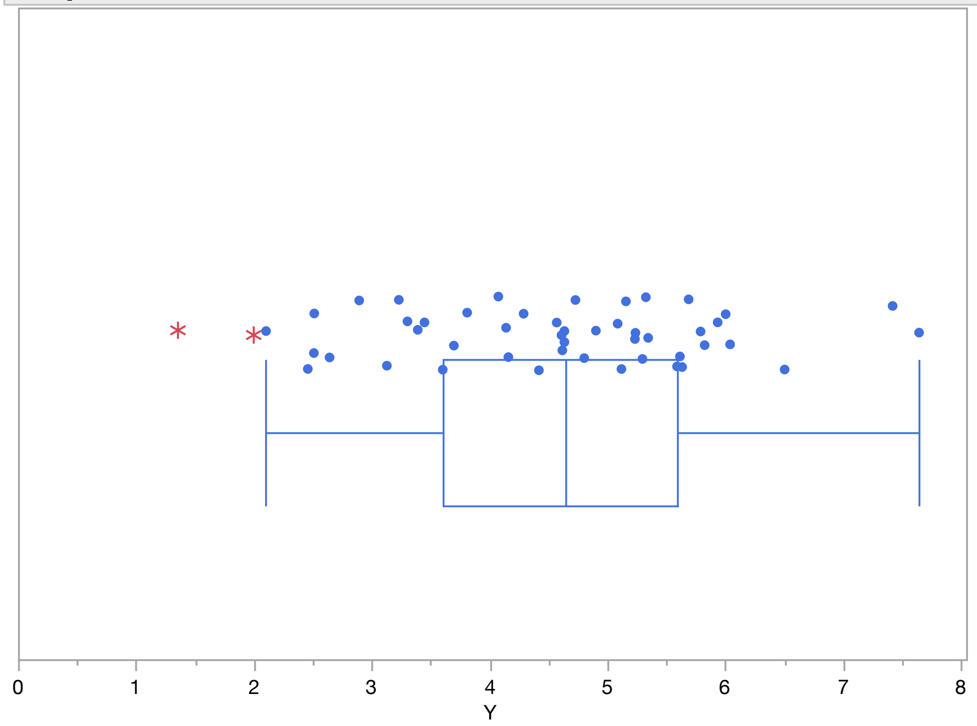


**Well = H12**

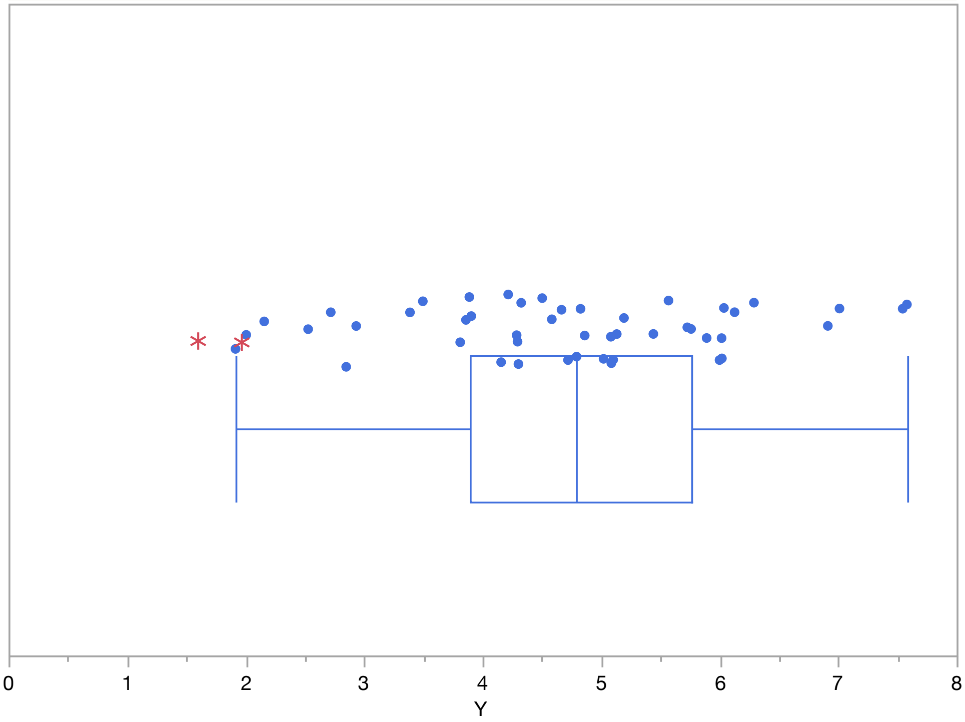

Supplement: Supplementary file 21 — Supplementary Material 21 [file 11011_2025_1546_MOESM21_ESM.pdf]
